# Phylum-wide analysis of genes/proteins related to the last steps of assembly and export of extracellular polymeric substances (EPS) in cyanobacteria

**DOI:** 10.1038/srep14835

**Published:** 2015-10-06

**Authors:** Sara B. Pereira, Rita Mota, Cristina P. Vieira, Jorge Vieira, Paula Tamagnini

**Affiliations:** 1 i3S—Instituto de Investigação e Inovação em Saúde, Universidade do Porto, Rua Júlio Amaral de Carvalho 245, 4200-135 Porto, Portugal; 2 IBMC—Instituto de Biologia Molecular e Celular, Universidade do Porto, Rua do Campo Alegre 823, 4150-180 Porto, Portugal; 3 Faculdade de Ciências, Departamento de Biologia, Universidade do Porto, Rua do Campo Alegre, Edifício FC4, 4169-007 Porto, Portugal

## Abstract

Many cyanobacteria produce extracellular polymeric substances (EPS) with particular characteristics (e.g. anionic nature and presence of sulfate) that make them suitable for industrial processes such as bioremediation of heavy metals or thickening, suspending or emulsifying agents. Nevertheless, their biosynthetic pathway(s) are still largely unknown, limiting their utilization. In this work, a phylum-wide analysis of genes/proteins putatively involved in the assembly and export of EPS in cyanobacteria was performed. Our results demonstrated that most strains harbor genes encoding proteins related to the three main pathways: Wzy-, ABC transporter-, and Synthase-dependent, but often not the complete set defining one pathway. Multiple gene copies are mainly correlated to larger genomes, and the strains with reduced genomes (e.g. the clade of marine unicellular *Synechococcus* and *Prochlorococcus*), seem to have lost most of the EPS-related genes. Overall, the distribution of the different genes/proteins within the cyanobacteria phylum raises the hypothesis that cyanobacterial EPS production may not strictly follow one of the pathways previously characterized. Moreover, for the proteins involved in EPS polymerization, amino acid patterns were defined and validated constituting a novel and robust tool to identify proteins with similar functions and giving a first insight to which polymer biosynthesis they are related to.

Cyanobacteria are a phylogenetically coherent and ancient group of prokaryotes that play key roles in the carbon and nitrogen cycles. Their long evolutionary history, autophototrophic metabolism and the diazotrophy of many strains are regarded as the main reasons for their success, with cyanobacteria thriving in different environments, from fresh to salt waters, soils, extreme environments or in symbiotic associations with different hosts^1^. These organisms show an unusual wide range of morphologies including unicellular, colonial and filamentous forms^1^,^2^. Owing to their simple and inexpensive nutritional requirements, metabolic plasticity and availability of tools for genetic manipulation, cyanobacteria are promising platforms for “green” biotechnology^3^.

Despite being generally classified as Gram-negative bacteria, cyanobacteria possess a cell envelope that combines both Gram-negative and Gram-positive features, including peptidoglycan layer thickness and cross-linking degree, presence of lipopolysaccharides (LPS), and outer membrane constituents^4^. Additionally, some strains also exhibit an S-layer^4^. In diazotrophic conditions, some filamentous strains are also able to differentiate specialized cells in N_2_-fixation, the heterocysts, which possess a thicker cell envelope that protects the nitrogenase from the O_2_ deleterious effects. The heterocysts’ envelope is composed by a glycolipid and a polysaccharide layers, with the latter being subdivided into an inner well-defined homogeneous and an external fibrous layers^4^,^5^. Many cyanobacteria also produce extracellular polymeric substances (EPS), mainly composed of polysaccharides, that can remain attached to the cell surface as sheaths, capsules or slime, or be released into the surrounding environment (released polysaccharides—RPS)^6^. These EPS serve as a boundary between the cyanobacterial cell and its immediate environment, and may play several roles depending on the strain and growth condition including protection against desiccation and UV, formation of biofilms and colonies, establishment of symbiosis, sequestration/immobilization of nutrients and metal ions^6^,^7^. Recently, it has also been shown that the bioactive compound Nostopeptolide could be found in the EPS^8^. The cyanobacterial EPS are more complex than those produced by other bacteria, usually comprising 6 to 13 different monosaccharides (including two different uronic acids and deoxysugars), ester-linked acetyl groups and peptidic moieties, and sulphate groups, which results in polymers rich in negative charged groups^6^. These characteristics make these polymers useful for several applications, including the bioremediation of heavy metals^6^,^9^. On the other hand, when growing cyanobacteria for other purposes, the production of EPS may be counterproductive by decreasing the energy available for growth, promoting biofilm formation and increasing the susceptibility to grazing^10^,^11^,^12^. As a result of this duality, the interest on cyanobacterial EPS increased and advances were made on the pathways leading to its production^6^,^7^,^11^,^12^,^13^.

Although the knowledge on EPS production in cyanobacteria is limited, their biosynthetic pathways seem to be relatively conserved throughout bacteria. It usually initiates in the cytoplasm with the conversion of monosaccharides into sugar nucleotides before being transferred by specific glycosyltransferases to carrier molecules located in the plasma membrane. Since several sugars are not specific of the EPS, many of the proteins involved in these processes are part of the general metabolism, differing according to the organism and determining the EPS composition^14^,^15^. The subsequent steps of polymerization, assembly and export are usually quite conserved, following one of three main mechanisms, namely the Wzy-, ABC transporter- or Synthase-dependent pathways, which have been described in detail elsewhere^15^,^16^,^17^,^18^,^19^,^20^. Briefly, in the Wzy-dependent pathway (Fig. 1, left), oligosaccharide lipid-linked repeating units are translocated to the periplasmatic side of the plasma membrane by Wzx, and polymerized by Wzy. Subsequently, the polymer is exported through the outer membrane by a transenvelope complex formed by Wzc and Wza, members of the polysaccharide copolymerase (PCP) and outer membrane polysaccharide export (OPX) families, respectively. Wzc phosphorylation state is controlled by the phosphatase Wzb^15^,^19^,^21^. In the ABC transporter-dependent pathway (Fig. 1, middle), the polysaccharide is fully polymerized at the inner leaflet of the plasma membrane before being translocated by an ABC transporter comprising two proteins, KpsM and KpsT. KpsC, KpsS, KpsF and KpsU are involved in the synthesis of the 3-deoxy-D-manno-oct-2-ulosonic acid (Kdo) linker (or its activated donor) that connects the polysaccharide to a terminal lipid. EPS export through the outer membrane is performed by the PCP protein KpsE and the OPX protein KpsD^15^,^17^,^19^,^22^. In the Synthase-dependent pathway (Fig. 1, right), the polysaccharide is simultaneously polymerized and exported across the plasma membrane by a synthase, Alg8 or BcsA in alginate or cellulose production, respectively. In both cases, c-di-GMP-binding is required, which is achieved by Alg44 in the former case or the synthase (BcsA) in the last. AlgI, AlgF and AlgG are involved in polymer modification at the periplasm, whereas AlgL degrades accumulated alginate. A similar hydrolytic function is predicted for BcsZ in the cellulose biosynthetic apparatus. In the last system, BcsB seems to be essential, although its specific role is unclear, whereas BscQ may be required for the polar localization of this apparatus. Alginate export depends on the scaffold protein AlgK and the AlgE porin, whereas for cellulose, this final step is performed by BcsC that combines the functions of AlgK and AlgE^16^,^23^. Some of the key proteins of these mechanisms are also involved in the assembly of other surface polysaccharides such as the O-antigen of LPS and S-layer glycans^24^,^25^. In cyanobacteria, previous studies lead to the identification of genes encoding proteins with the typical domains found in Wzx and Wzy, both characteristic of the Wzy-dependent pathway, suggesting that EPS assembly and export should, in most cases, proceed via this mechanism^6^,^13^. However, the lack of homologues of genes encoding key proteins of the Wzy- and ABC transporter-dependent pathways in some cyanobacterial strains raised the hypothesis that, in these organisms, EPS production may not exactly fit the existing models^13^. These differences are also evident in the physical organization of the EPS-related genes in cyanobacteria compared to that observed in other bacteria, with the gene copies scattered throughout the genomes, either isolated or in small clusters^6^,^13^.

This study aims to identify genes/proteins related to the last steps of EPS production in cyanobacteria and evaluate its distribution pattern among the strains. For this purpose, a phylogenomic analysis was performed focusing on the characteristic genes/proteins of the main mechanisms of bacterial EPS assembly and export: the Wzy-, ABC transporter- and Synthase-dependent pathways. The data obtained were refined taking into account available functional information for cyanobacterial EPS-related genes, by performing tblastn searches using *Synechocystis* sp. PCC 6803 sequences as query. Moreover, making use of the recently improved coverage of the cyanobacterial phylum, the relationship between the genes distribution and the strains’ position in the phylogenetic tree, morphological and ecological features was evaluated. For the proteins involved in EPS polymerization, amino acid patterns were defined and validated, providing the first indication on the polymer’s nature and highlighting the potential of cyanobacteria as a prolific source of different EPS. These motifs constitute a novel and robust tool to identify proteins with similar functions, as more bacterial genomes are becoming available.

## Results

### Cyanobacterial strains

Following the recent increase in the number of available cyanobacterial genomes, 124 strains were selected to investigate putative genes/proteins related to EPS assembly and export in cyanobacteria. Most of the selected strains are from marine or freshwater environments. Nevertheless, 19 strains are known to thrive in multiple habitats (others including symbiosis and/or combinations of the previously mentioned ones), and 12 are adapted to hot springs or soils. The reference information (metadata) comprising genomic, morphological, physiological and ecological data is compiled in Supplementary Data 1: Tables S1 and S2. A species tree generated by the maximum likelihood analysis of 31 conserved proteins concatenated is already available^26^ and, for a subset of these cyanobacteria, a consensus tree inferred from 324 single-copy proteins was also previously assembled^27^. Overall, the major phylogenetic relationships are in agreement, with only six incongruences observed (Supplementary Data 4: Fig. S1).

### Conserved domains related to EPS production

**Figure 1:**
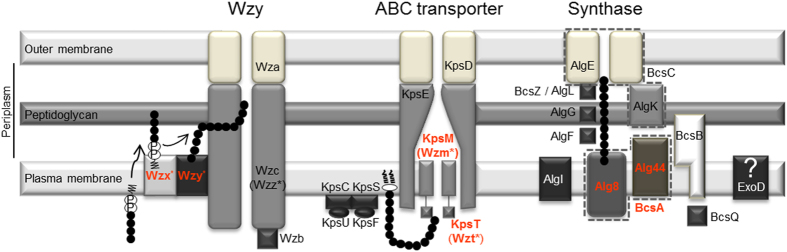
Schematic representation of the main bacterial EPS assembly and export pathways. Characteristic proteins (hallmarks) of each pathway are indicated in orange and bold. Proteins that participate in O-antigen assembly are indicated by *. Homologues exclusively involved in O-antigen assembly are indicated between parentheses. ? indicates that ExoD is involved in EPS production, but that its exact role and/or relationship with the main pathways is still unclear. The interrupted lines around Alg8 and Alg44 and AlgE and AlgK mean that BcsA and BcsC are single proteins containing domains present in Alg8 and Alg44 and in AlgE and AlgK, respectively. Updated from Pereira *et al.*^13^.

**Figure 2:**
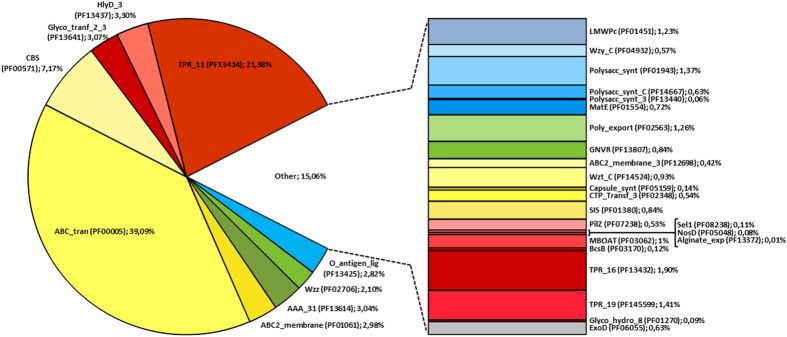
Relative abundance of sequences containing EPS-related Pfam domains in cyanobacteria. For each domain, the designation, accession number and percentage of sequences is indicated. Domains represented in blue are found in proteins involved in the Wzy-dependent pathway; domains in yellow are found in proteins involved in the ABC transporter-dependent pathway; domains in green are found in proteins involved either in the Wzy- or in the ABC transporter-dependent pathway; domains in pink-red are present in proteins involved in the Synthase-dependent pathway; the domain in grey is found in ExoD and it is not clear in which pathway this protein is involved.

Initially, the conserved domains were identified by screening bacterial proteins sequences involved in well-characterized systems of EPS assembly and export, including those following the Wzy-, ABC transporter- or Synthase-dependent pathways (Fig. 1). Additionally, other domains known to be associated to protein families involved in the transport/export of polysaccharides according to the information available in the Transporter Classification Database^28^ or in the literature were also considered. In total, 36 Pfam domains were selected (Table 1). Some of these domains are present in proteins playing similar functions in different pathways of EPS assembly and export (e.g. Poly_export domain of Wza and KpsD proteins) or in proteins involved in the EPS and/or O-antigen production (e.g. O-antigen_lig domain of the Wzy polymerase and WaaL ligase). In addition, for putative Wzx, the screening comprised not only members of the polysaccharide transporter (PST) family, but also of the evolutionary related multi-drug and toxin extrusion (MATE) family^23^,^29^. The screening of the cyanobacterial genomes/theoretical proteomes for the selected Pfam domains resulted in the identification of 18843 domain sequences (Supplementary Data 1: Table S3), present in 17745 proteins (Supplementary Data 2). The analysis of the number of domain sequences across all the selected cyanobacterial theoretical proteomes revealed an uneven distribution among the strains (Supplementary Data 1: Table S3). Overall, the ABC_tran was shown to be the most frequent domain, accounting for 39.09% of all domains identified, followed by TPR_11 that was present in 21.38% of the sequences retrieved (Fig. 2). Three other domains were shown to be essential for cyanobacteria, being present in all strains analyzed: AAA_31, ABC2_membrane, and CBS. The HlyD_3, Glyco_tranf_2_3 and O-antigen_lig domains are also abundant in cyanobacteria, being present in 113, 123 and 118 strains, respectively. In contrast, the WzyE, AlgF, Alginate_lyase and BcsC_C domains were absent in all strains analyzed, and the Alginate_exp, Polysacc_synt_3, NosD, Glyco_hydro_8, Sel1, BcsB and Capsule_synt were present in a very restricted number of organisms.

### Cyanobacterial proteins putatively involved in EPS production

To refine the dataset and strengthen the analysis, the sequences previously obtained were curated by performing tblastn searches using *Synechocystis* sp. PCC 6803 sequences as query. Due to the absence or low number of the domains identified for the majority of the proteins involved in alginate or cellulose modification and export, and since no putative AlgF, AlgG, AlgI, AlgL, AlgK, AlgE, BcsB and BcsZ were found in *Synechocystis* sp., these proteins were not selected for further analysis. The same criteria was used for BcsC, due to the absence of the BcsC_C domain in all strains analysed, being only the structural tetratricopeptide repeat domains identified. Overall, the blast searches resulted in the identification of 10164 cyanobacterial proteins putatively involved in EPS assembly and export (EPS-related proteins; Supplementary Data 1: Table S4 and Supplementary Data 3). In contrast to that observed for KpsS/KpsC, KpsF and KpsU, the majority of the EPS-related proteins have homologues in most cyanobacterial strains, with the exception of the *Cyanobacterium* sp. UCYN-A, *Thermosynechococcus elongatus* BP-1, *Synechococcus* spp. JA-3-3Ab and JA-2-3B’a(2–13), and the clades formed by *Synechococcus* spp., *Prochlorococcus marinus* and *Cyanobium* spp., or by *Synechococcus elongatus* PCC 7942 and PCC 6301. Concerning the PCP proteins, namely Wzc (PCP-2a), KpsE (PCP-3) or Wzz (PCP-1), from the 513 sequences retrieved by blast, 386 possessed the Wzz domain. Given that the Wzz domain was present in the *Synechocystis* sp. PCC 6803 sequence used as query and all 513 hits passed the significance threshold, a conservative approached was adopted by retaining all sequences until further examination. More than 7000 homologues were identified for KpsT/Wzt, suggesting that the blast filtering was not sufficient to discriminate between these proteins and other ATP-binding modules of ABC transporters. Regarding the characteristic proteins of the synthase-dependent pathway, no sequences possessing the typical multi-domain architecture of Alg44, consisting of both the PilZ and HlyD_3 domains, were found in cyanobacteria. Differently, from the 454 putative synthase sequences obtained after blast, 70 possessed the Glyco_tranf_2_3 and PilZ domains, typical of the BcsA proteins. Taking into account these results, putative Wza/KpsD, Wzb, Wzc/KpsE/Wzz, Wzy/WaaL, Wzx, KpsM/Wzm, KpsS/KpsC, KpsU, KpsF, Alg8/BcsA, and ExoD were selected for further analysis.

### Frequencies and distribution of cyanobacterial proteins putatively involved in EPS production

To evaluate possible statistical significant correlations between the frequency of the putative EPS-related proteins and the strains’ morphology, presence of persistent sheath and genome size (in ordinal scale) the Spearman’s rank correlation coefficient was calculated. The Eta coefficient test was also determined by considering the cyanobacterial morphology, presence of persistent sheath, genome size and habitat as categorical variables (Table 2). Significant positive correlations were found for most of the EPS-related proteins, with the notable exceptions of KpsS/KpsC, KpsU, KpsF for which no significant and/or low positive or negative values of correlation were obtained. For the remaining putative EPS-related proteins, the lowest correlation values were repeatedly observed for the cytoplasmic Wzb. This protein shows a very unique distribution pattern in cyanobacteria, with one to three homologues present in the strains analyzed, with the only exception of the *Cyanobacterium* sp. UCYN-A for which no Wzb was found. The frequencies of putative Wza/KpsD, Wzc/KpsE/Wzz, Wzy/WaaL, Wzx, KpsM/Wzm, Alg8/BcsA, and ExoD are positively correlated with the strains’ morphology, presence of persistent sheath and genome size. However, whereas most of the values obtained for the strains’ morphology and presence of persistent sheath are not particularly high (i. e., <0.600), those observed for the genome size indicate a strong correlation. The coefficient values obtained for the putative EPS-related proteins mentioned above and the strains’ habitat are relatively low (generally <0.500).

**Figure 3:**
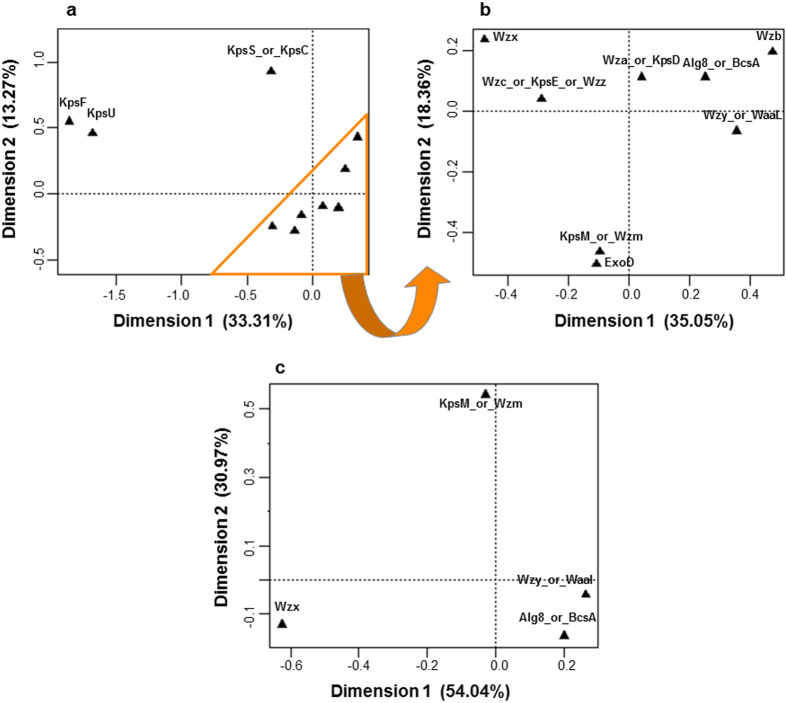
Multiple Correspondence Analyses (MCA) of the frequency of putative EPS-related proteins in cyanobacteria. For each analysis, the percentage of variance explained by the first two dimensions is indicated in parenthesis. (**a**) MCA of all putative EPS-related proteins included in this study. Two major groups, separated by the first and second components, are observed. The larger group is encircled in orange. (**b**) MCA of the putative EPS-related proteins included in the larger group defined by the first analysis. (**c**) MCA of the characteristic proteins of each of the main characterized pathways of EPS assembly and export.

For a more thorough understanding of the relationships between the different putative EPS-related proteins, Multiple Correspondence Analyses (MCA) were carried out (Fig. 3). In a first approach, a broaden analysis comprising all blast hits was performed, unveiling a division of the putative EPS-related proteins into two groups separated by the first and second components (Fig. 3a). This division clearly separates KpsS/KpsC, KpsU, KpsF from all other EPS-related proteins, supporting the results previously obtained. A second analysis focusing on the proteins comprised on the larger group defined by the first MCA, revealed that Wzb, Wzy/WaaL, and Alg8/BcsA, are all located on the right site of the plot, within the area defined by the higher values of the first component. With only four exceptions, these proteins are ubiquitously present in cyanobacteria, whereas the other putative EPS-related proteins are usually absent in *Cyanobacterium* sp. UCYN-A, *Thermosynechococcus elongatus* BP-1, *Synechococcus* spp. JA-3-3Ab and JA-2-3B’a (2–13), and strains belonging to the clades formed by *Synechococcus* spp., *Prochlorococcus marinus* and *Cyanobium* spp., or by *Synechococcus elongatus* PCC 7942 and PCC 6301 (Supplementary Data 1: Table S4 and Supplementary Data 3). Interestingly, the distribution pattern of ExoD across the cyanobacteria analyzed is more closely related to that of KpsM/Wzm than to other proteins (Fig. 3b). In addition, a MCA of the cyanobacterial homologues of characteristic proteins of the main pathways clearly demonstrated that Wzx, KpsM/Wzm and the polymerases/synthases cluster formed by Wzy/WaaL and Alg8/BcsA have a strain-dependent distribution pattern (Fig. 3c).

**Figure 4:**
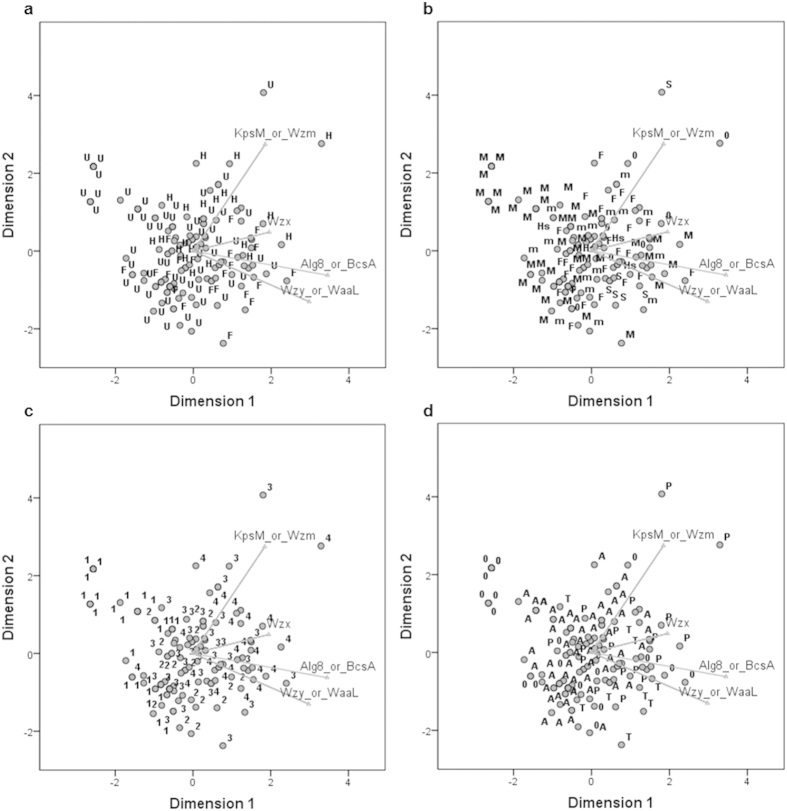
Categorical Principal Component Analysis (CATPCA) of the frequency of characteristic proteins of the main pathways of EPS assembly and export in cyanobacteria. The component loadings, displaying the correlation between the quantified variables (selected proteins) and the principal components are shown. (**a**) Distribution of the strains according to morphology: U, unicellular; F, filamentous; H, heterocystous. (**b**) Distribution of the strains according to habitat: 0, no information; m, multiple or other; F, freshwater; Hs, freshwater/hot spring; M, marine; S, soil. When the information available was discordant, the strains were considered to have multiple habitats. (**c**) Distribution of the strains according to genome size: 1, <3 Mbp; 2, 3–5 Mbp; 3, 5–7 Mbp; 4, >7 Mbp. (**d**) Distribution of the strains according to the presence of a persistent sheath, based on genus information: 0, no information; A, absence; T, present but thin; P, present. Information retrieved from the IMG database and literature data^2^,^26^,^27^,^58^.

To evaluate if the distribution of these characteristic proteins is related to certain features of the strains, a Categorical Principal Component Analysis (CATPCA) was performed (Fig. 4). The strains were plotted on the CATPCA labelled according to its morphology (Fig. 4a), habitat (Fig. 4b), genome size (Fig. 4c) and presence of persistent sheath (Fig. 4d). The results showed that the strains’ morphology and habitat do not have a strong effect on the distribution of the Wzx, KpsM/Wzm, Wzy/WaaL and Alg8/BcsA, as all categories have a broad distribution on the CATPCA plot. Despite that, a group of unicellular marine cyanobacteria, formed by strains belonging to the *Synechococcus* and *Prochlorococcus* clade, appears on the left side of the plot, well separated from all the other cyanobacteria. Concerning the genome size, the categories seem to be progressively distributed along the first component, increasing concomitantly with the number of proteins. A higher abundance of strains with a persistent sheath, either thin or developed, can be observed on the right side of the plot. Overall, the statistical analyses emphasize the unique distribution pattern of KpsS/KpsC, KpsU, KpsF and shows that the frequencies observed for the other EPS-related proteins are mainly linked with the strains’ genome size. In addition, the results reveal that most of the characteristic proteins of each pathway have a strain-dependent distribution pattern, with Wzy/WaaL and Alg8/BcsA being ubiquitous within the phylum.

### Analysis of cyanobacterial OPX and PCP proteins

**Figure 5:**
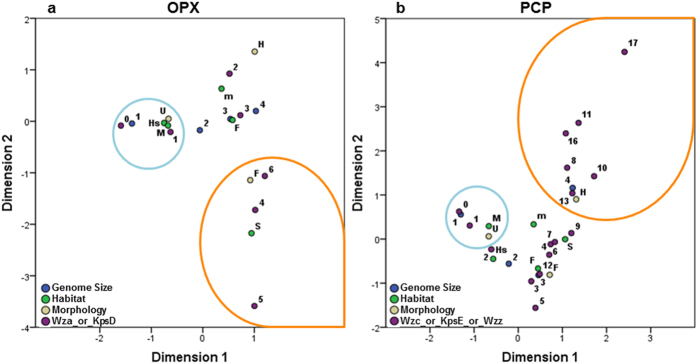
Multiple Correspondence Analyses (MCA) of putative OPX or PCP proteins and the genetic, morphological and ecological features of cyanobacteria. Joint category quantification plots of the classification of strains according to its morphology, habitat, genome size and the frequency of putative (**a**) outer membrane polysaccharide export (OPX) or (**b**) polysaccharide copolymerase (PCP) proteins. Regarding morphology, strains were classified as: U, unicellular; F, filamentous; H, heterocystous. Regarding habitat, strains were classified as: m, multiple or other; F, freshwater; Hs, freshwater/hot spring; M, marine; S, soil. When the information available was discordant strains were considered to have multiple habitats. Regarding genome size, strains were classified as: 1, <3 Mbp; 2, 3–5 Mbp; 3, 5–7 Mbp; 4, >7 Mbp. Categories with similar coordinates and, thus, more closely related to the absence/lower number or higher number of putative OPX and PCP proteins are encircled in blue and orange, respectively.

Consistent with the key role played by PCP and OPX proteins in the export of EPS by the Wzy- and ABC transporter-dependent pathways in Gram-negative bacteria, OPX and PCP homologues were identified for the majority of cyanobacteria (Supplementary Data 1: Table S4 and Supplementary Data 3). This outcome contrasts with the lack of confident identifications for their functional counterparts on the synthase-dependent pathway, namely, AlgK and AlgE (alginate) or BcsC (cellulose) (see above). To examine the diversity of the putative PCP proteins identified in cyanobacteria, a multiple sequence alignment was built and sequences were sorted by similarity (Supplementary Data 4). Twelve bacterial sequences belonging to the main groups of PCP proteins defined by Cuthbertson *et al.*^19^ were also included in this analysis. Most of the cyanobacterial PCP homologues exhibited high sequence similarity to the PCP-2a, presenting the characteristic C-terminal extension and, in many cases, the canonical Walker A and B domains. Another 122 cyanobacterial sequences, from which 99 lacked the Wzz domain, were shown to be more divergent, gathering in the bottom of the alignment. From those, 39 closely related proteins presented a smaller length (about 350 amino acids instead of 700–800) and are mostly annotated as ATPases. Possible associations between the frequency of OPX and PCP proteins and the strains’ morphology, genome size, and habitat were investigated by MCA. For this purpose, joint category plots were constructed to unveil the relationships between the different classes of each variable (Fig. 5). The results revealed that the absence or presence of only one OPX and PCP homologue is mostly associated to unicellular marine strains with genome sizes under 3 Mbp. However, while a higher abundance of OPX proteins is associated to filamentous and/or soil inhabiting strains, heterocystous cyanobacteria possess increased numbers of PCP homologues. In both plots, the classes defined for the genome size were progressively increasing along the first component, emerging as the main trait that influences the distribution of these proteins in cyanobacteria. Similar results were obtained in an MCA performed with the PCP blast hits restricted to the subset of sequences containing the Wzz domain (data not shown).

### Analysis of characteristic proteins of the main pathways of EPS production and identification of amino acid patterns for Wzy/WaaL or Alg8/BcsA

As identifiable hallmarks of the Wzy-, ABC transporter- and Synthase-dependent pathways, Wzx and Wzy/WaaL, KpsM/Wzm and Alg8/BcsA were selected for an extended analysis. Maximum Likelihood (ML) unrooted trees of the cyanobacterial sequences were generated using the sequences limited to their conserved domains (data not shown). Putative Wzx clustered in different groups, depending on the presence of the Polysacc_synt (or the closely related Polysacc_synt_3) or MatE domains. In contrast, for the putative Wzy/WaaL, the groups were heterogeneous, comprising sequences with the O-antigen_lig or the Wzy_C domains. Bootstrapped supported groups were also obtained for Alg8/BcsA, all with the Glyco_tranf_2_3 domain. Moreover, all the sequences possessing the additional PilZ domain were included in the same group.

**Figure 6:**
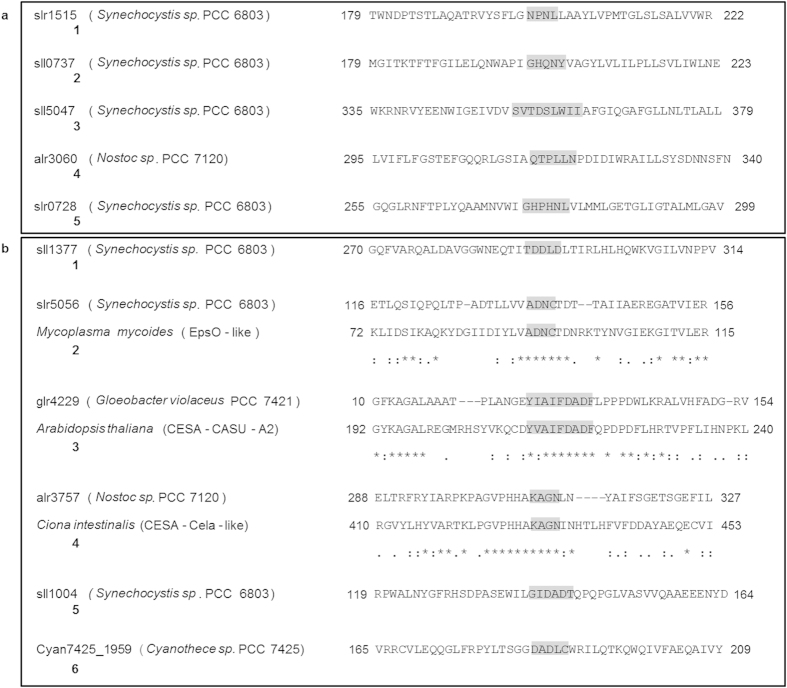
Amino acid patterns defined for cyanobacterial Wzy/WaaL and Alg8/BcsA. The relative locations of the amino acid patterns defined for (**a**) Wzy/WaaL and (**b**) Alg8/BcsA cyanobacterial phylogenetic groups are shown as shaded regions. For each pattern, only one cyanobacterial sequence, indicated by locus tag and (strain name), is shown as example. When applicable, reference sequences are shown, indicated by species name and (conserved domain designation). Numbers refer to amino acid positions within the sequences. *Positions with conserved residues; Positions with residues of strongly similar properties; Positions with residues with weakly similar properties.

Following on the ubiquitous distribution of the Wzy/WaaL and Alg8/BcsA and due to their crucial role as polymerases/synthases, specific amino acid patterns were defined for cyanobacterial sequences based on the clusters defined by the Neighbor-Joining trees (Supplementary Data 4: Figs S2 and S3). It is important to highlight the reference sequences retrieved from the conserved domain database were not used to find the patterns. Rather than allowing to establish phylogenetic relationships between sequences and/or groups of sequences, these distance matrix-based trees constitute an important tool to infer groups of closely related sequences that may share a given amino acid pattern. In total, five and six amino acid patterns were defined for putative Wzy/WaaL and Alg/BcsA, respectively (Table 3 and Fig. 6). For each strain, the frequency/distribution of the sequences within the phylogenetic groups used to define amino acid patterns can be found in Supplementary Data 1: Table S5. Regarding Wzy/WaaL, group one comprises one sequence from the majority of the cyanobacteria, with the exception of the clade formed by *Prochlorococcus marinus* NATL2A to *Prochlorococcus marinus* subsp. *marinus* CCMP 1375, for which no sequence was identified, and *Chamaesiphon minutus* PCC 6605 that possesses two sequences in this group. This group also includes all the TIGR00947 (2A73; putative bicarbonate transporter, IctB family) and one pfam04932 (Wzy_C; O-Antigen ligase) representatives, which are cyanobacterial sequences identified in this work as putative Wzy/WaaL. Groups two, three and four are bootstrap supported clusters of sequences belonging to a restricted number of organisms namely, 24, 26, 17, respectively, usually closely related. While group two includes sequences from the early and late branches of the species tree, those present in groups three and four are mostly from the late-branching organisms, mainly from heterocystous strains in the case of group four, with medium to large genomes. Similar to that observed for group one, group five also includes sequences from the majority of the strains analysed, with the exeptions of *Prochlorococcus marinus* MIT 9515, 9215 and 9312, *Cyanobium* sp. PCC 7001, *Thermosynechococcus elongatus* BP-1, and two *Acaryochloris* spp. strains. Most of the sequences comprised in group five share the same amino acid pattern and, with the only one exception, only one sequence/strain can be found. Concerning Alg8/BcsA, the bootstrapped supported group one was found to be the larger, including one (or less frequently two) sequence from most cyanobacteria, with the exceptions of *Cyanobacterium* sp. UCYN-A and *Arthrospira platensis* Paraca. The sequences clustered in group two belong to a restrict subset of 43 strains, mainly from the late branches of the species tree and with medium to large genomes. All sequences included in this group share an amino acid pattern also found in most cd06438 (EpsO-like; involved in the methanolan synthesis) reference sequences. Group three includes only eight sequences, all possessing an amino acid pattern also found in half of the cd06437 (CESA_CaSu_A2; Cellulose synthase catalytic subunit A2) and one cd06421 (CESA_CelA_like; involved in the elongation of the glucan chain of cellulose) reference sequences. The sequences comprised in group four, including those with the PilZ domain, show a widely spread distribution within the phylum, being present in 73 strains, with a number of sequences per strain varying up to four. All of these sequences possess an amino acid pattern found in more than half of the cd06421 and cd04191 (Glucan_BSP_ModH; elongation of beta-1,2 polyglucose chains of glucan) and one cd06437 reference sequences. Finally, whereas group five includes sequences from 87 cyanobacteria, group six contains sequences from only 14 strains, with no particular distribution. Overall, the results show that, although cyanobacteria possess several Wzy/WaaL and Alg8/BcsA orthologues, more or less widely distributed within the phylum, the Wzy/WaaL sequences have diverged considerably from those of other organisms, whereas Alg8/BcsA proteins may share amino acid patterns with e.g. bacteria, plants or fungi.

## Discussion

Despite the increasing interest on cyanobacterial EPS, the knowledge on the specific pathways leading to its production is still limited. Hence, we proposed to identify genes/proteins related to the last steps of EPS production and evaluate its distribution among cyanobacteria. Although the number of strains in each Order/subsection is different, with Pleurocapsales/subsection II and Stigonematales/subsection V having fewer representatives, the dataset covers the phylum and reflects its diversity in terms of phylogenetic groups, morphological types and ecological niches^26^. For this purpose, a phylogenomic analysis was performed focusing on the characteristic genes/proteins of the main pathways of bacterial EPS assembly and export (see Fig. 1)^16^,^30^. However, the involvement of proteins from each of these pathways or their functional counterparts in the assembly of other surface polysaccharides, such as the O-antigen of LPS or the S-layer glycans, increases the complexity of the analysis^24^,^25^,^31^,^32^,^33^.

Concerning the selected Pfam domains, the most abundant in cyanobacteria are not exclusively related to EPS production, but also associated with other cellular activities, including transport, energy-sensing or mediation of protein-protein interactions^34^,^35^,^36^,^37^. The ones present in Alg8/BcsA or Wzy/WaaL (also very abundant in cyanobacteria) are specifically involved in the polymerization/ligation of bacterial polysaccharides^16^,^38^. The ones with the most restricted distribution or even absent are related to the production of particular polysaccharides, such as the enterobacterial common antigen (WzyE) or the modification and export of alginate or cellulose through the outer membrane (AlgF, Alginate_lyase, Alginate_exp, BcsB and BcsC_C)^16^,^23^,^39^.

As mentioned in the Results section tblastn searches were performed using *Synechocystis* sp. PCC 6803 sequences as query. This strategy was adopted since *Synechocystis* sp. PCC 6803 is a model cyanobacterium and, therefore, the role of some of these proteins is supported by functional studies^11^,^12^. Recently, two other studies identified EPS- and LPS-related genes in *Microcystis* spp. and *Synechococcus elongatus* PCC 7942, respectively^7^,^10^. In general, the genes identified in this work were the same before data curation. The main differences are the *wzx* genes in *Microcystis* and the *wzy*/*waal* in *S. elongatus* which are the less conserved and therefore do not pass the defined threshold.

For most of the cyanobacteria included in the dataset, the theoretical proteome harbors EPS-related proteins putatively involved in each of the three main pathways of bacterial EPS assembly and export. The wide distribution within the phylum observed for Wza/KpsD, Wzb, Wzc/KpsE/Wzz, Wzx, Wzy, KpsM/Wzm, Alg8/BcsA, ExoD is consistent with vertical inheritance and subsequent losses of their encoding genes. This is further supported by the presence of proteins possessing the same Pfam domains and/or correspondent COG classifications in the list of orthologs present in the putative “most recent common cyanobacterial ancestor”^40^. The presence of several homologues of most of these EPS-related proteins was previously reported for a smaller set of strains than the one analyzed here^6^,^13^ and, not surprisingly, the higher numbers are associated to larger genomes, probably due to paralogous duplications or horizontal gene transfer (HGT) events. This hypothesis is consistent with gene duplication being the main force of genome evolution in microorganisms, including cyanobacteria, creating the potential for broadening the phenotypes and subsequently the adaptive behavior of the organisms^40^,^41^,^42^. A strong correlation was observed between the genome size and the distribution/frequency of EPS-related proteins, but this relationship was far more limited for morphology and habitat. In fact, although smaller genomes belong to unicellular cyanobacteria restricted to marine environments or hot springs, cell differentiation and genome size are not directly related^40^, with the heterocystous *Raphidiopsis brookii* D9 and *Cylindrospermopsis raciborskii* CS-505 possessing small to medium genomes and two unicellular strains from the *Acaryochloris* genus having rather large genomes (7.8 and 8.3 Mbp). Likewise, although the habitat imposes a set of forces that shapes biodiversity, cyanobacteria with larger genomes have also wider distribution^40^ occupying marine and freshwater environments, soils, or multiple habitats. Regarding the absence/presence of a persistent sheath, although it was shown to be related to the distribution/frequency of EPS-related proteins, it is necessary to consider that the information refers to the genus^2^ and is absent for many cyanobacteria and most of the strains produce a type of EPS, either in the form of a well-defined sheath, a thick capsule, mucilage or released polysaccharides (RPS), and it remains to be known if these structures are assembled following the same/different pathways. Despite all these considerations, the coherent picophytoplanktonic clade of *Prochlorococcus* and *Synechococcus* strains is particularly distinctive by lacking most of the EPS-related proteins. These unicellular cyanobacteria possess the smallest genomes and do not produce a conspicuous persistent sheath. These strains adopted an ecologically successfully genome streamlining strategy to adapt to their specific niches, with a progressive genome size reduction from *Synechococcus* to *Prochlorococcus* isolates^40^,^43^. *Synechococcus* strains are present in most marine environments, whereas those belonging to *Prochlorococcus* genus are restricted to warmer oligotrophic oceans^44^. In these environmental contexts, the energy-demanding endeavor of producing EPS provides no obvious advantage, as no immediate need of protection from desiccation, UV radiation or adherence to solid substrates is required. Hence, it is likely that many of the genes encoding the EPS-related proteins were lost during the evolutionary process.

In the three main pathways of bacterial EPS assembly and export, the transmembrane events are fundamentally different^30^. Regarding the initial steps of the process, the confined distribution of putative KpsS/KpsC, KpsF and KpsU dissociates the assembly of cyanobacterial EPS from the established ABC transporter-dependent pathways, suggesting the absence of the poly-Kdo linker in cyanobacterial EPS, or the existence of different mechanisms to transfer Kdo residues to the phospholipid acceptor^15^,^22^ . In contrast, the Wzy/WaaL (Wzy-dependent) and Alg8/BcsA (synthase-dependent) are the most ubiquitous proteins found in the cyanobacterial strains analyzed. Wzy is involved in the polymerization of both EPS and O-antigen^15^,^18^,^19^,^38^,^45^ and may share the same domains with WaaL, which mediates the ligation of the O-antigen chains to lipid A-core^24^,^31^,^38^. The absence of the predicted WaaL ligase of *Synechococcus elongatus* PCC 7942^10^ from our dataset after blast data curation, raises the hypothesis that, in cyanobacteria, the similarity between Wzy and WaaL is not as strong as in other organisms. The wide distribution observed for the Wzy/WaaL is consistent with their key role in the transfer of lipid-linked oligosaccharides to a sugar acceptor^38^, either to another oligosaccharide repeating unit in the case of Wzy (polymerization) or to the lipid A-core in the case of WaaL (ligation). In the synthase-dependent pathway, the c-di-GMP-binding PilZ domain of Alg44 is regarded as absolutely required for the simultaneous polymerization and export of alginate through the plasma membrane, although there are no experimental evidences that Alg8 and Alg44 are responsible for this process^16^,^23^. Despite the ubiquitous distribution of Alg8/BcsA, no sequences possessing the multi-domain architecture of Alg44 were identified, whereas 70 out of 454 possess both the Glyco_tranf_2_3 and PilZ domains typical of the cellulose synthase. Our results corroborate and expand previous data suggesting that cellulose synthase are widespread among cyanobacteria and that cellulose biosynthesis is a common feature in these organisms^46^,^47^. Considering the absence of the identified proteins involved in alginate or cellulose modification and export in the majority of the cyanobacteria, it is likely that their synthases interact with different cell components or that the proteins are different than those of other bacteria. In the ABC transporter-dependent pathway, the polysaccharide is fully polymerized by the sequential action of glycosyltransferases before being translocated through the plasma membrane^17^,^20^. Due to the large number of possible linkages and, consequently, of specific glycosyltransferases^14^, we did not attempted to identify these proteins.

Concerning the transport of the repeating units/polymer through the plasma membrane, a different outcome was observed, with the absence of obvious Wzx (either with the Polysacc_synt/Polysacc_synt_3 or MatE domains; Wzy-dependent) and/or KpsM/Wzm (ABC transporter-dependent) candidates in several cyanobacteria. These results may be somewhat unexpected considering that these proteins (Wzx and Wzm) may participate in the assembly of the O-antigen of LPS that is suggested to predominantly follow the ABC transporter-dependent pathway in cyanobacteria^10^. In addition, Wzm is known to participate in the transport of bacterial S-layer glycans^25^. Nevertheless, the lack of O-antigen does not impair growth and the presence of an S-layer is not a universal feature^4^,^10^. Alternatively, it is possible that these proteins were not identified due to the low degree of conservation reflecting the different sugar compositions of its substrates^20^,^29^,^48^ or due to the existence of additional transport mechanisms.

Finally, the simultaneous wide distribution observed for the putative Wzc/KpsE/Wzz and Wza/KpsD provides a strong indication that cyanobacterial EPS are exported by a process requiring both members of the PCP and OPX protein families, even if the existence of additional mechanisms cannot be ruled out. The homology between most of the cyanobacterial sequences and bacterial PCP-2a proteins, further suggests the prevalence of a Wzc/Wza complex typical of the Wzy-dependent pathway, as previously proposed for a smaller subset of strains^13^,^19^,^49^,^50^. The ubiquitous presence of the Wzc phosphatase—Wzb—in theoretical proteomes of cyanobacteria strengthens this hypothesis^19^. The different abundances observed for PCP and OPX proteins may be related with the involvement of PCP proteins in the assembly and export of both EPS (PCP-2a and PCP-3) and O-antigens (PCP-1), while OPX proteins will participate only in the export of EPS^19^,^21^,^49^,^50^. In addition, the higher abundance of PCP proteins in heterocystous strains is consistent with previous findings^13^ and points towards the involvement of these proteins in the assembly of the polysaccharidic layer of the heterocysts^5^.

Although the exact role of the predicted membrane protein ExoD is still unknown, this protein was shown to participate in the production of bacterial EPS, including those of *Synechocystis* sp. PCC 6803^51^. The broad distribution of this protein within the phylum, present in early- and late-branching lineages, is in agreement with its important role although not essential for cell survival^11^.

The analysis of the amino acid patterns of the ubiquitous Wzy/WaaL and Alg8/BcsA provided further information about these proteins, highlighting groups of closely related sequences that may play similar functions and/or be under the control of similar regulatory mechanisms. Regarding Wzy/WaaL, the lack of reference sequences from organisms other than cyanobacteria, is consistent with the low sequence conservation reported for the Wzy polymerases and WaaL ligases^18^,^38^. Why putative ictB proteins such as slr1515 and Synpcc7942_0357, possess domains known to be involved in bacterial oligosaccharide polymerization and ligation remains unclear. However, the inability to isolate fully segregated mutant on their encoding genes^52^,^53^, is consistent with the high conservation degree of the proteins included in group one (Supplementary Data 4: Fig. S2). It is also interesting that homologues from this group are only absent in *Prochlorococcus* strains, since it has been shown that this genus does not possess CO_2_ uptake systems or Cmp-dependent HCO_3_^−^ transport^53^,^54^. Likewise, the wide distribution of the sequences included in group five points out to a conserved cellular function, whereas the presence of the sequences from groups two, three and four among closely related strains suggests vertical inheritance of their encoded genes followed by occasional losses, presumably providing certain ecological benefits (Supplementary Data 4: Fig. S2). Regarding the Alg8/BcsA, the presence of sequences from group one in all cyanobacteria with the exception of one strain displaying severe loss of genome reduction^40^, emphasizes the essential role of these phylum-specific glycosyltransferases (Supplementary Data 4: Fig. S3). In addition, the different groups of Alg8/BcsA sequences, some restricted to cyanobacteria and others sharing amino acid patterns with EPS-related proteins from other organisms clearly demonstrates the potential of cyanobacteria to produce a variety of EPS, ranging from strain-dependent polymers to cellulose, one of the most abundant polymers on earth.

In conclusion, this work demonstrates that most cyanobacteria harbors genes encoding proteins related to the three main pathways of bacterial EPS assembly and export, displaying a more complex scenario than that observed for other bacteria. Multiple gene copies are correlated to larger genome, probably as a consequent of gene duplications during cyanobacteria long evolutionary history. Besides, the clade of unicellular strains from the *Synechococcus* and *Prochlorococcus* genera seems to have lost most of the EPS-related proteins during their adaptation to the marine environment. The absence of some of the proteins surveyed and the broad distribution of Wzy/WaaL and Alg8/BcsA within the phylum raises the hypothesis that, in cyanobacteria, EPS production may not follow the existing patterns. Nevertheless, it is currently unknown if all EPS-related proteins are being expressed simultaneously or if their encoding genes are under different regulatory mechanisms. The sequence analysis and amino acid patterns confirm the potential of cyanobacteria as a prolific source of EPS and provide a valid tool to identify proteins with similar functions as more genomes became available. In addition, the data generated in this work provide a robust basis for further studies that will clarify the process of EPS production in cyanobacteria.

## Material and Methods

### Identification of protein domains involved in the assembly and export of EPS

Proteins involved in well-characterized systems of bacterial assembly and export of EPS following the Wzy-, ABC transporter- or synthase-dependent pathways were screened for their Pfam domains using the Integrated Microbial Genomes (IMG) database (v4, Sep. 2013; https://img.jgi.doe.gov/cgi-bin/w/main.cgi)^55^. Regarding the Wzy-dependent pathway, the screening comprised proteins from *Escherichia coli K*12-W3110, *Burkholderia cenocepacia* PC184, *Erwinia amylovora* ATCC 49946, *Lactococcus cremoris* NIZO B40 and *Pseudomonas aeruginosa* PAO1^19^,^45^. For the ABC transporter-dependent pathway, proteins from *Neisseria meningitidis* Z2491, *Escherichia coli* O1:K1:H7 and *Pseudomonas aeruginosa* PAO1^19^,^20^ were analyzed. The domains associated to the synthase-dependent pathway were investigated in proteins from *Pseudomonas aeruginosa* PAO1, *Escherichia coli* K-12 MG1655 and W3110^16^,^23^. Other domains associated to recognized protein families involved in the transport/export of polysaccharides were also included according to the information available in the literature or by screening the sequences of members of the TC:1.B.13, TC:1.B.18, TC:2.A.50, TC:2.A.66, TC:8.A.3, TC:8.A.4, TC:9.A.41, and TC:9.B.67 transport systems available in the Transporter Classification Database (http://www.tcdb.org)^28^. For each protein(s), the domains selected for subsequent analysis are listed in Table 1.

### Screening of protein domains involved in assembly and export of EPS in cyanobacterial genomes

The presence or absence of genes encoding proteins with the selected Pfam domains was investigated in 124 cyanobacterial genomes using the available information at the IMG database (v4.510, Oct. 2014). The cyanobacterial strains selected for this study belong to different Orders/subsections, and display different physiological and ecological features. Moreover, their phylogenetic relationship has been recently assessed^26^,^27^. For the selected proteins/Pfam domains, the lists of encoding genes were filtered by using it as subject sequences in tblastn searches (NCBI, http://www.ncbi.nlm.nih.gov/) against *Synechocystis* sp. PCC 6803 sequences^11^,^12^. The query sequences used are indicated in Table 4.

### Identification of amino acid patterns

For both Wzy/WaaL and Alg8/BcsA, representative cyanobacterial sequences were blasted against the Conserved Domain Database to identify the family to which they belong to. Members of all subfamilies of the identified families, namely cl04850 (Wzy_C) and cd06423 (CESA_like), were downloaded. These reference sequences, together with the cyanobacterial sequences were aligned using ClustalW as implemented in MEGA5^56^ and Neighbor-Joining trees were generated using the same software. Cyanobacterial sequences belonging to major clusters were used as a guide to define amino acid patterns as previously described in Fonseca *et al.*^57^. The presence of the patterns was then checked in the reference sequences.

### Statistical analysis

Data for the classification of the cyanobacterial genomes was retrieved from the IMG database (v4.510, Oct. 2014), the Pasteur Culture Collection (https://www.pasteur.fr/en/research/crbip-biological-resource-center-institut-pasteur/open-collections/pasteur-culture-collection-cyanobacteria-pcc) and available literature^26^,^27^,^58^. The Multiple Correspondence Analysis (MCA) of the frequency of the EPS-related proteins was computed in R^59^. All other statistical analyses were performed using the SPSS Statistics 20 software (IBM)^60^. To calculate the Spearman Rank Order Correlations, the frequency of the different proteins were used in their original metric scale and the strains’ categorical classifications were transformed into ordinal variables according to the coding system available in Supplementary Data 1: Table S6. To calculate the Eta coefficient, the frequencies of the different proteins were defined as dependent variables and the categorical classifications, coded numerically as indicated above, as independent variables. Categorical Principal Component Analysis (CATPCA) and MCA for OPX and PCP proteins were performed selecting the variable principal normalization method and a weight of one for all variables. In CATPCA, the frequencies of the different proteins were defined as numerical (scale) variables.

## Additional Information

**How to cite this article**: Pereira, S. B. *et al.* Phylum-wide analysis of genes/proteins related to the last steps of assembly and export of extracellular polymeric substances (EPS) in cyanobacteria. *Sci. Rep.* **5**, 14835; doi: 10.1038/srep14835 (2015).

## Supplementary Material

Supplementary Data 1

Supplementary Data 2

Supplementary Data 3

Supplementary Information

## Figures and Tables

**Table 1:** Pfam domains selected for analysis.

Putative protein	Pfam accession no.	Pfam designation
Wza, KpsD	PF02563	Poly_export
Wzb	PF01451	LMWPc
Wzc, KpsE (Wzz)	PF02706	Wzz
	PF13807	GNVR
	PF13614	AAA_31
Wzy (WaaL)	PF06899	WzyE
	PF04932	Wzy_C
	PF13425	O-antigen_lig
Wzx	PF01943	Polysacc_synt
	PF14667	Polysacc_synt_C
	PF13440	Polysacc_synt_3
	PF01554	MatE
KpsM (Wzm)	PF01061	ABC2_membrane
	PF12698	ABC2_membrane_3
KpsT (Wzt)	PF00005	ABC_tran
	PF14524	Wzt_C
KpsS, KpsC	PF05159	Capsule_synth
KpsU	PF02348	CTP_transf_3
KpsF	PF01380	SIS
	PF00571	CBS
Alg8, BcsA	PF13641	Glyco_tranf_2_3
Alg44, BcsA	PF07238	PilZ
	PF13437	HlyD_3
AlgK	PF08238	Sel1
AlgE	PF13372	Alginate_exp
AlgF	PF11182	AlgF
AlgG	PF05048	NosD
AlgI	PF03062	MBOAT
AlgL	PF05426	Alginate_lyase
BcsB	PF03170	BcsB
BcsC	PF05420	BcsC_C
	PF13432	TPR_16
	PF14559	TPR_19
	PF13414	TPR_11
BcsZ	PF01270	Glycol_hydro_8
ExoD	PF06055	ExoD

The same Pfam domains may be present in proteins playing similar functions in different pathways of EPS assembly and export (e.g. Wza and KpsD), or in proteins involved in the EPS or O-antigen production (e.g. Wzy and WaaL). Proteins exclusively involved in O-antigen production are indicated within parenthesis.

**Table 2:** Correlations between the frequency of EPS-related proteins and the strains’ genetic, morphological and ecological features.

Putative protein	Wzb	Wzc, KpsE (Wzz)	Wzy (WaaL)	Wzx	KpsM (Wzm)	KpsS, KpsC	KpsU	KpsF	Alg8, BcsA	ExoD	Morphology	Persistent Sheath	Genome Size	Habitat
Wza, KpsD	0,107	**0,620**	**0,363**	**0,525**	**0,514**	0,033	−0,101	−**0,326**	**0,374**	**0,354**	**0,468**	**0,287**	**0,641**	
											0,590	0,281	0,601	0,301
Wzb		**0,253**	**0,202**	**0,189**	0,098	−0,051	−0,077	0,098	0,249	−0,017	0,093	0,129	**0,273**	
											0,147	0,130	0,336	0,126
Wzc, KpsE (Wzz)			**0,467**	**0,688**	**0,633**	0,028	−**0,179**	−0,166	**0,597**	**0,552**	**0,630**	**0,436**	**0,809**	
											0,609	0,611	0,754	0,322
Wzy (WaaL)				**0,581**	**0,574**	0,077	−0,140	−**0,181**	**0,528**	**0,459**	**0,206**	**0,342**	**0,558**	
											0,234	0,363	0,602	0,329
Wzx					**0,588**	0,125	−0,124	−0,070	**0,595**	**0,498**	**0,403**	**0,435**	**0,718**	
											0,340	0,558	0,643	0,227
KpsM (Wzm)						0,104	−0,142	−**0,208**	**0,485**	**0,600**	**0,458**	**0,428**	**0,672**	
											0,440	0,388	0,629	0,451
KpsS, KpsC							0,118	0,146	0,120	0,047	0,165	0,141	0,070	
											0,201	0,230	0,212	0,136
KpsU								**0,467**	−**0,194**	−0,091	−**0,284**	−0,005	−**0,247**	
											0,248	0,110	0,264	0,230
KpsF									0,020	−0,127	−**0,188**	−0,098	−**0,177**	
											0,198	0,097	0,278	0,209
Alg8, BcsA										**0,292**	**0,363**	**0,376**	**0,584**	
											0,414	0,426	0,601	0,391
ExoD											**0,341**	**0,366**	**0,598**	
											0,370	0,361	0,626	0,340

First line: Spearman’s rank correlation coefficients (association between ranked variables); statistical significant correlations (p value < 0.05) are highlighted in bold. Second line: Eta coefficient values (correlation between quantitative and categorical variables). Proteins exclusively involved in O-antigen production are indicated within parenthesis. Genetic, morphological and ecological features are based on the information available at the IMG database and literature data^2^,^26^,^27^,^58^.

**Table 3:** Wzy/WaaL and Alg8/BcsA amino acid patterns for cyanobacterial phylogenetic groups (see Supplementary Data 4: Figs S2 and S3).

Putative Protein	Amino acid pattern	Coverage		
		within the group	outside the group	Reference sequences
Wzy/WaaL	NPNL	114/114 (100%)	0/212 (0%)	10/10 (100%) TIGR00947: 2A73
				1/60 (1.6%) pfam04932: Wzy_C
	GH[HPQ]N[FY]	33/34 (97.1%)	0/ 292 (0%)	1/60 (1.6%) pfam04932: Wzy_C
	[ST][AITV][APT]DS[FL]W[IV][IL]	26/26 (100%)	0/300 (0%)	—
	[ADENQS]TP[IL][FIL]N	19/19 (100%)	0/307 (0%)	—
	[AFGLNPY]H[ACEPSTV]H[NS][FIL]	111/118 (94.1%)	0/208 (0%)	—
Alg8/BcsA	TDDLD	128/128 (100%)	0/326 (0%)	—
	ADNC	48/48 (100%)	0/406 (0%)	23/25 (92.0%) cd06438: EpsO_like
	[LY][IV][AMT]IFDADF	8/8 (100%)	3/446 (0.7%)	6/12 (50.0%) cd06437: CESA_CaSu_A2
				1/53 (1.9%) cd06421: CESA_CelA_like
	KAGN	121/121 (100%)	0/333 (0%)	36/53 (67.9%) cd06421: CESA_CelA_like
				1/12 (83.3%) cd06437: CESA_CaSu_A2
				10/17 (58.8%) cd04191: Glucan_BSP_ModH
	[FGL][IMTV]DAD[ITV]	102/105 (97.1%)	1/349 (0.3%)	—
	[DE][AS]D[FIL]C	15/17 (88.2%)	0/437 (0%)	—

**Table 4:** Sequences from *Synechocystis* sp. PCC 6803 used as query in tblastn searches.

Locus tag	IMG gene ID	Putative homologues
sll1581*	637010084	Wza, KpsD
slr0328	637011521	Wzb
slr0923*	637009749	Wzc, KpsE (Wzz)
sll0737	637012282	Wzy (WaaL)
slr1515	637010893	
slr0728	637009524	
slr1074	637009782	
sll5047	637471849	
sll5049	637471851	Wzx
slr1543	637010329	
slr0896	637011947	
slr0977*	637009752	KpsM (Wzm)
slr2107	637010911	
sll0574*	637012623	
slr0982*	637009756	KpsT (Wzt)
slr2108	637010912	
sll0575*	637012622	
slr2115	637010921	KpsS, KpsC
slr2122	637010928	KpsU
slr2111	637010917	KpsF
slr1566	637012477	Alg8, BcsA
sll1377	637011129	
sll1004	637009901	
slr5056	637471858	
sll1181	637011139	Alg44, BcsA
sll1481	637012497	
slr1875*	637010543	ExoD

*Synechocystis*’s sequences were identified by screening the genome/theoretical proteomes for EPS-related Pfam domains.

^*^The role of the protein in EPS is supported by functional data^11^,^12^.
