# Supplementary material for: Phylum-wide analysis of genes/proteins related to the last steps of assembly and export of extracellular polymeric substances (EPS) in cyanobacteria
Source: Sci Rep. 2015 Oct 6;5:14835. doi: 10.1038/srep14835 (PMC4594306; doi:10.1038/srep14835)
Supplement: Supplementary Information [file srep14835-s4.pdf]

Phylum-wide analysis of genes/proteins related to the last steps of assembly and export of extracellular polymeric substances (EPS) in cyanobacteria

Sara B. Pereira[1,2*], Rita Mota[1,2,3], Cristina P. Vieira[1,2], Jorge Vieira[1,2], Paula Tamagnini[1,2,3*]

[1]i3S – Instituto de Investigação e Inovação em Saúde, Universidade do Porto, Rua Júlio Amaral de Carvalho 245, 4200-135 Porto, Portugal

[2]IBMC – Instituto de Biologia Molecular e Celular, Universidade do Porto, Rua do Campo Alegre 823, 4150-180 Porto, Portugal

[3]Faculdade de Ciências, Departamento de Biologia, Universidade do Porto, Rua do Campo Alegre, Edifício FC4, 4169-007 Porto, Portugal

*Correspondence and requests for materials should be addressed to S. B. P. (email: sarap@ibmc.up.pt) or P. T. (email: pmtamagn@ibmc.up.pt)

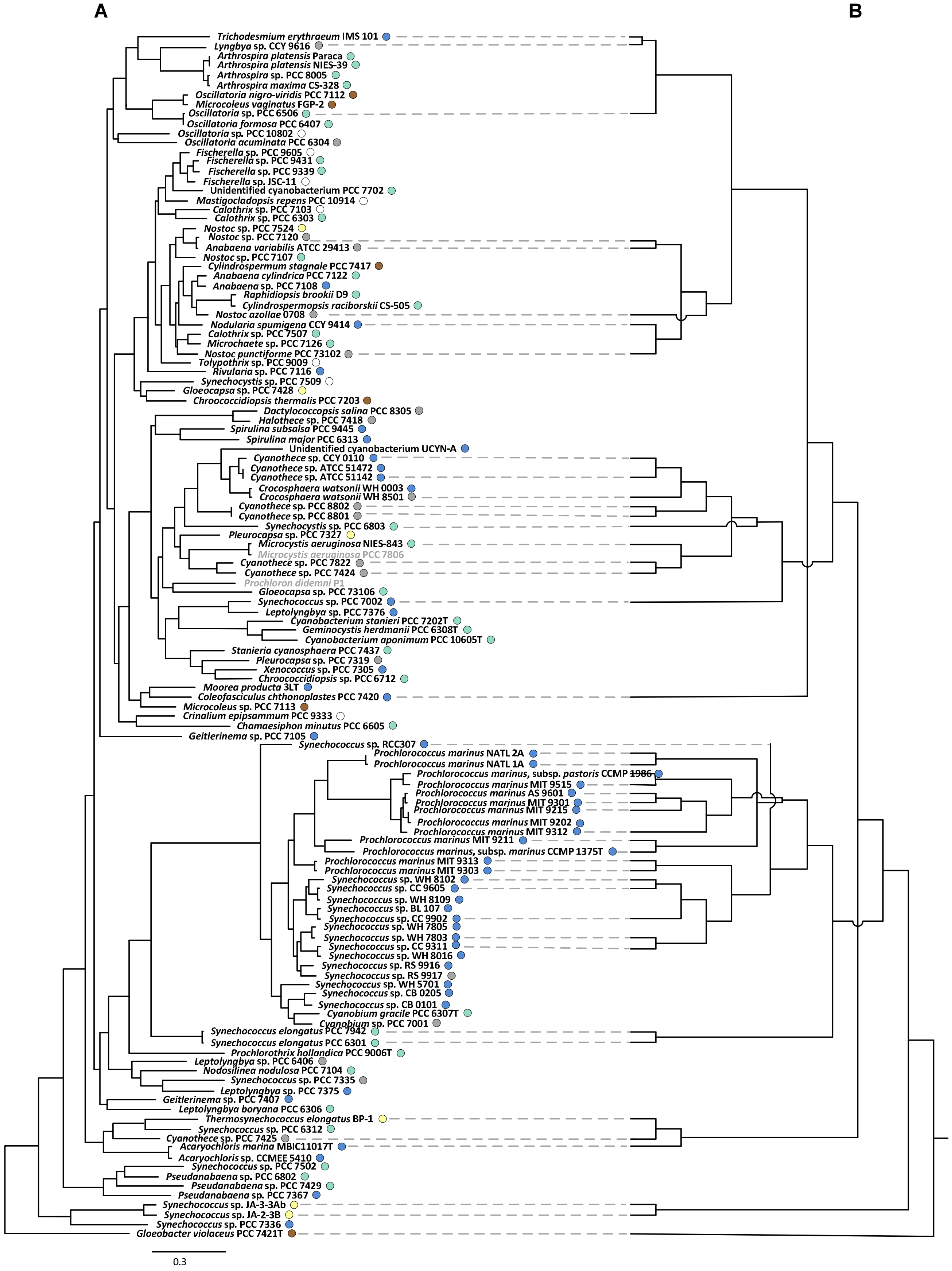

A
B
Trichodesmium erythraeum IMS 101
Lyngbya sp. CCY 9616
Arthrospira platensis Paraca
Arthrospira platensis NIES-39
Arthrospira sp. PCC 8005
Arthrospira maxima CS-328
Oscillatoria nigro-viridis PCC 7112
Microcoleus vaginatus FGP-2
Oscillatoria sp. PCC 6506
Oscillatoria formosa PCC 6407
Oscillatoria sp. PCC 10802
Oscillatoria acuminata PCC 6304
Fischerella sp. PCC 9605
Fischerella sp. PCC 9431
Fischerella sp. PCC 9339
Fischerella sp. JSC-11
Unidentified cyanobacterium PCC 7702
Mastigocladopsis repens PCC 10914
Calothrix sp. PCC 7103
Calothrix sp. PCC 6303
Nostoc sp. PCC 7524
Nostoc sp. PCC 7120
Anabaena variabilis ATCC 29413
Nostoc sp. PCC 7107
Cylindrospermum stagnale PCC 7417
Anabaena cylindrica PCC 7122
Anabaena sp. PCC 7108
Raphidiopsis brookii D9
Cylindrospermopsis raciborskii CS-505
Nostoc azollae 0708
Nodularia spumigena CCY 9414
Calothrix sp. PCC 7507
Microchaete sp. PCC 7126
Nostoc punctiforme PCC 73102
Tolypothrix sp. PCC 9009
Rivularia sp. PCC 7116
Synechocystis sp. PCC 7509
Gloeocapsa sp. PCC 7428
Chroococcidiopsis thermalis PCC 7203
Dactylococcopsis salina PCC 8305
Halothece sp. PCC 7418
Spirulina subsalsa PCC 9445
Spirulina major PCC 6313
Unidentified cyanobacterium UCYN-A
Cyanothece sp. CCY 0110
Cyanothece sp. ATCC 51472
Cyanothece sp. ATCC 51142
Crocosphaera watsonii WH 0003
Crocosphaera watsonii WH 8501
Cyanothece sp. PCC 8802
Cyanothece sp. PCC 8801
Synechocystis sp. PCC 6803
Pleurocapsa sp. PCC 7327
Microcystis aeruginosa NIES-843
Microcystis aeruginosa PCC 7806
Cyanothece sp. PCC 7822
Cyanothece sp. PCC 7424
Prochloron didemni P1
Gloeocapsa sp. PCC 73106
Synechococcus sp. PCC 7002
Leptolyngbya sp. PCC 7376
Cyanobacterium stanieri PCC 7202T
Geminocystis herdmanii PCC 6308T
Cyanobacterium aponimum PCC 10605T
Stanieria cyanosphaera PCC 7437
Pleurocapsa sp. PCC 7319
Xenococcus sp. PCC 7305
Chroococcidiopsis sp. PCC 6712
Moorea producta 3LT
Coleofasciculus chthonoplastes PCC 7420
Microcoleus sp. PCC 7113
Crinalium epipsammum PCC 9333
Chamaesiphon minutus PCC 6605
Geitlerinema sp. PCC 7105
Synechococcus sp. RCC307
Prochlorococcus marinus NATL 2A
Prochlorococcus marinus NATL 1A
Prochlorococcus marinus, subsp. pastoris CCMP 1986
Prochlorococcus marinus MIT 9515
Prochlorococcus marinus AS 9601
Prochlorococcus marinus MIT 9301
Prochlorococcus marinus MIT 9215
Prochlorococcus marinus MIT 9202
Prochlorococcus marinus MIT 9312
Prochlorococcus marinus MIT 9211
Prochlorococcus marinus, subsp. marinus CCMP 1375T
Prochlorococcus marinus MIT 9313
Prochlorococcus marinus MIT 9303
Synechococcus sp. WH 8102
Synechococcus sp. CC 9605
Synechococcus sp. WH 8109
Synechococcus sp. BL 107
Synechococcus sp. CC 9902
Synechococcus sp. WH 7805
Synechococcus sp. WH 7803
Synechococcus sp. CC 9311
Synechococcus sp. WH 8016
Synechococcus sp. RS 9916
Synechococcus sp. RS 9917
Synechococcus sp. WH 5701
Synechococcus sp. CB 0205
Synechococcus sp. CB 0101
Cyanobium gracile PCC 6307T
Cyanobium sp. PCC 7001
Synechococcus elongatus PCC 7942
Synechococcus elongatus PCC 6301
Prochlorothrix hollandica PCC 9006T
Leptolyngbya sp. PCC 6406
Nodosilinea nodulosa PCC 7104
Synechococcus sp. PCC 7335
Leptolyngbya sp. PCC 7375
Geitlerinema sp. PCC 7407
Leptolyngbya boryana PCC 6306
Thermosynechococcus elongatus BP-1
Synechococcus sp. PCC 6312
Cyanothece sp. PCC 7425
Acaryochloris marina MBIC11017T
Acaryochloris sp. CCMEE 5410
Synechococcus sp. PCC 7502
Pseudanabaena sp. PCC 6802
Pseudanabaena sp. PCC 7429
Pseudanabaena sp. PCC 7367
Synechococcus sp. JA-3-3Ab
Synechococcus sp. JA-2-3B
Synechococcus sp. PCC 7336
Gloeobacter violaceus PCC 7421T
0.3

Figure S1. Comparison of phylogenetric trees available for the cyanobacterial strains included in this work. (A) Species tree generated by the maximum likelihood analysis of 31 conserved proteins concatenated [1]. (B) Neighbor-joining consensus tree inferred from 324 single-copy protein families common the cyanobacteria analyzed by Dagan et al. [2]. Incongruences between the strains phylogenetic relationships obtained by the two studies are indicated by half circles. The strains' habitat, based on the information available at the IMG database and literature [1-3]**,** is shown by a colored circle: white, no information; grey, multiple or other; green, fresh water; yellow, fresh water, hot string; blue, marine; brown, soil. When the information available in the literature was discordant, strains were considered to have multiple habitats.

Partial CLUSTAL multiple sequence alignment of putative PCP (polysaccharide copolymerase) protein sequences. In addition to the cyanobacterial sequences (label: IMG ID and locus tag), 12 sequences from other bacteria (label: NCBI Gi and accession number, highlighted in grey) were included (Table 1). These sequences were selected in order to comprise homologues from each of the main groups of PCP proteins defined by Cuthbertson et al. [4]. Sequences are sorted by similarity. Canonical Walker A and B motifs (below) [5-9] are highlighted in yellow and blue, respectively, in the cyanobacterial sequences. For the other bacterial sequences, Walker A and B motifs, as defined by Cuthbertson et al. [4] are highlighted in grey. The labels of 39 cyanobacterial sequences with shorter length (about 350 amino acids) are underlined.

Walker A – [A/G]XXXXGK[S/T], in which X represents any amino acid

Walker B – hhhhD, in which h represents an hydrophobic amino acid

Table 1 Bacterial PCP (polysaccharide copolymerase) sequences used in alignment

Accession no.	Organism	Name	PCP
AAD21564	*Escherichia coli* K30	Wzc	PCP-2a
AAV27324	*Klebsiella pneumoniae* K1	Wzc	PCP-2a
NP_418983	*Caulobacter crescentus* CB15		PCP-2a-like
NP_437626	*Sinorhizobium meliloti* 1021	ExoP	PCP-2a-like
AAA86371	*Xanthomonas campestris* pv. *campestris*	GumC	PCP-2a-like
NP_250925	*Pseudomonas aeruginosa* PAO1	PslE	PCP-2a-like
YP_110442	*Burkholderia pseudomallei* K96243		PCP-1
CAA62141	*Vibrio cholera* O139	OtnB	PCP-1
CAA52655	*Escherichia coli* K5	KpsE	PCP-3
AAB51624	*Escherichia coli* K1	KpsE	PCP-3
YP_109401	*Burkholderia pseudomallei* K96243	WcbD	PCP-3
CAA38731	*Haemophilus influenzae* serotype b	BexC	PCP-3

PCP alignment (PARTIAL)

```
2505798636_Cal7507_0387             --L----------------TLERPIRSLVISSALPGDGKSTVAFQLAQTSAAMGKRV-LL
2509783408_Mic7126DRAFT_4160        --L----------------NFERPIRSLVVSSPLPGDGKSTVAFQLAQTATAMGKRV-LL
648052066_Aazo_4925                 --L----------------NSDQPIRSLTISSTMPGDGKTTVSFHLAQIAAALGKRV-LL
2504133443_Anacy_3769               --L----------------NSDQPIRSLTVSSTMPGDGKTTVSFHLAQIAAALGKRV-LL
2506494313_Ana7108_4449             --L----------------NSDKPIHALVVSSAMPGDGKTTVSFHIAQIAAALGKRV-LL
2512980750_Fis9431DRAFT_5079        --L----------------SSDRPIRSIVISSAMPGDGKSTIAFHLAEIACAMEQRV-LL
2517062414_PCC9339DRAFT_03271       --L----------------SSDRPIRSIVISSAMSGDGKSTVAFHLAEIACAMEQRV-LL
2505767894_FJSC11DRAFT_3537         --L----------------SSDRPIRSIVMTSAVSGDGKSTVAFHLAEIACAMEQRV-LL
2516145585_FIS9605DRAFT_02588       --L----------------SSDRPIRSIVISSAMSGDGKSTVAFHLAEIACAMGQRV-LL
```

```
2504096080_Cal6303_3081                    --L----------------SSDRPVHSVVVSSAMSGDGKSTIAFHLAQVAATMGQRV-LL
2507333046_Tol9009DRAFT_00030320           --L----------------SSDRPIRSIIISSAMSGDGKSTVAFHLAQIATAMGQRV-LL
2507476338_Cal7103DRAFT_00032380           --L----------------GSDRPIRSIVVSSAMNSDGKSTIAFHLAKIAAVMGQRV-LL
2505802134_Cal7507_3852                    --L----------------NCDRPIHSIITASAMPGDGKSTIAFHLAQIATAMGQRV-LL
2509782674_Mic7126DRAFT_3425               --L----------------ESDRSIHSLIISSAMPRDGKSTIALHLAQIATAMGRRV-LL
2509771979_CylstDRAFT_5939                 --L----------------STDRQIRSIIISSAMPGDGKSTVAFHLAQIATGMGQRV-LL
2503738747_Nos7107_0132                    --L----------------NSDHPIRSIIITSSMPSDGKSTVAFNLAQIATAMGQRV-LL
2509809495_Nos7524_1926                    --L----------------NTDRQIRSITISSAMPGDGKSTIAFYLAQIATAMGQRV-LL
640026730_N9414_07896                      --L----------------NSDSQIRSITVSSVMAGEGKSTIAFNLAQIAASIGKRV-LL
2517239228_Mas10914DRAFT_0471              --L----------------SSDQRIRSIVISSAMAGDGKSTVAFHLAQIASAMGQRV-LL
2510087477_Riv7116_2331                    --L----------------SSDEKINSLVITSAMSGDGKSTVAFHLAQAASAIGRRV-LL
2510087323_Riv7116_2177                    --L----------------SSDRQVRSIVISSALPGDGKSTVAYNLAQVAASMGQKV-LL
2507477177_Cal7103DRAFT_00040780           --L----------------SSDSPIRSIVISSAEPGDGKSLVSYHLAEVAATMGKRV-LL
637235631_all5222                          --I----------------KPKSQLRSLVVSSVMAGDGKSIVAFYLAQTATAMGLRV-LL
2506482271_LYNGBM3L_49700                  --L----------------SSDRPIRSLAISSALPGDGKSTIAIYLAQTAAAIGKKV-LL
641611510_SYNPCC7002_A1500                 --L----------------SADRPLRSVIIASALPGDGKSTIALNLAPTAAILGQKV-LL
2509842234_Lepto7375DRAFT_2251             --M----------------GADRTIRSVIISSSMPNEGKSTVARHLAQTAATMGKRV-LL
647107641_CRC_03354                        --L----------------NSDEVVKSVAITSALKGEGKSTLALHLAQMAASVGRRV-LL
647110196_CRD_02439                        --L----------------NSDKVIKSLTVSSAIKGEGKSTISSYLALVAASLGKRV-LL
2509804452_LepboDRAFT_3794                 --L----------------NTDHPVRSLVVSSPTAGDGKTTVALYLAQAAAAMGQRV-LL
647567980_MC7420_2223                      --I----------------SPDQPIRSVVISSAVPADGKSTIATFMAQAAAAMGQRV-LL
2509437593_Mic7113_5758                    --I----------------SPDQPIRSLVISSSTPADGKSTITTFLAQAAAAMGLRV-LL
2506479760_LYNGBM3L_56010                  --L----------------YSEKPISAIVVSSAEPADGKSTTAIFLALAAAAMGQKV-LL
647567242_MC7420_5618                      --L----------------GSDTPVHSIIISSATPSEGKSTVSVHLAQAAAAMGQRV-LL
2509436287_Mic7113_4452                    --L----------------GSDTPIHSIVISSATPGDGKSTVSVHLAQAAAAMGQRV-LL
2503796318_Glo7428_3737                    --L----------------GSDTPIHSLVISSASHADGKSTIAAHLAQAAAAMGQRV-LL
2517697153_SYN7509DRAFT_1270               --L----------------GSDTPINSLVVSSAQKGDGKSTVSIQLAQAAAAMGQKV-LL
2503615076_Chro_4796                       --L----------------GSDTPIHSLVISSSVHADGKSTISTHLAQAAAAMGRRV-LL
2509422734_Oscil6304_3527                  --M----------------SPDRPLKSIVISSTLPAEGKTTISMKLAEAASAMGNRV-LL
2506601543_Spi9445_4406                    --F----------------TPDKPLQVLVISSSVPLEGKSTTSINLAQAAAAMGQRV-LL
2506609271_Spi6313_1630                    --F----------------TPDKPLQVIVVSSSVPLEGKSTTAIHLAQTAAAMGQKV-LI
640014711_L8106_15385                      --L----------------SSDTPIKSFVISSPSQSDGKSTLAVHLAQAAAAMGNRV-LL
2509507761_Osc10802DRAFT_1411              --L----------------GAGSPIRSLAVSSPAPADGKSTVAAHLALAAAAMGQRV-LL
2509420889_Oscil6304_1682                  --L----------------TPGKPLRSLAIASAIPAEGKSTVAVNLAEAAASMGQRV-LL
2503798162_Sta7437_0103                    --L----------------GSDSPINSIVISSSIPSEGKSTISSHLAQAAAAMGQKV-LL
2505785430_Chr6712_1674                    --L----------------GSDSPINSIVISSSIPSEGKSTISSHLAQAAAAMGQRV-LL
2509711143_Pleur7313DRAFT_05131            --L----------------GSESAMRSFVISSSTPSEGKSTISCHLAQAAAAMGQKV-LL
2508648278_Xen7305DRAFT_00003760           --L----------------GSDANINSIVVSSSIPSEGKSTVSSHLAQAASAMGQKV-LL
2508646941_GLO73106DRAFT_00031960          --L----------------GSDSLIKSLVISSSAPSEGKSTISLNLAKAASAMGQKV-LL
2509573356_Ple7327_1473                    --L----------------GGDREFNSLVISSAQAGDGKSTVSTNLAQAAAAMGQRV-LV
641537293_MAE_32940                        --L----------------GSDSPLKSLVISSVSPGDGKTTMAINIAKAAARMGQKV-LL
643482531_PCC7424_4310                     --L----------------DSHQPLKSIAISSTTAREGKSTIALYLAKVVAMMGQRV-LL
648185689_Cyan7822_0712                    --L----------------DS-EPLKSIVISSATAGEGKSTIALYLAQVAAVMGQRV-LL
2509502225_Pro9006DRAFT_3724               --L----------------QSDTPLMSLVVSSALPEDGKSTVSLNLAQAAAAMGQRV-LL
643584098_Cyan7425_1087                    --L----------------GTDSPIRSLVLSSALPADGKSTTSYNMALAAAVMGQRV-LL
2503742029_Nos7107_3348                    --L----------------DPDKPLKSLVISSALPGEGKSTIAVYLAQVAASMGKRV-LL
2504097563_Cal6303_4545                    --Y----------------SVESQVRSITVTSTAGKEGKSTVALQLALIAAQAGQRV-LL
2507478127_Cal7103DRAFT_00050290           --M----------------SQEMPLRTISVTSTTSGEGRTTVAVNLAQIAAEAGQRV-LL
2510089327_Riv7116_4181                    --L----------------KEETPIASLVITSANPGEGKTTVAATFAKTAAEAGLRV-LL
2517239184_Mas10914DRAFT_0427              --L----------------KSETSIRSIVITSATSGDGKSTVAANLAKIAAQAGQRV-LL
2507335438_Tol9009DRAFT_00054260           --Y----------------KMEASTRSIAITSATSSEGKTTVAAYLAQMAAEAGARV-LL
2504086624_Osc7112_0134                    --L----------------NVNTPINSCVITSCQVADGKSTVAVNLARAAAAMGQQV-LL
2506346341_MicvaDRAFT_2066                 --L----------------NLDTPINSCVITSCQVADGKSTVAVNLARAAAAMGQQV-LL
648859632_OSCI_3640004                     --L----------------NVDSPIRSCAITSCQVADGKSTVAVNLAMAAAAMGQRV-LL
2508872900_Oscil6407DRAFT_00010170         --L----------------NVDSPIRSCAITSCQVADGKSTVAVNLAMAAAAMGQRV-LL
643171775_AmaxDRAFT_4132                   --L----------------ATESPINSCVISSARPADGKSTVALNLAIGAAAMGQRV-LL
648386075_APCC8_010100004460               --L----------------ATESPINSCVISSARPADGKSTVALNLAIGAAAMGQRV-LL
650384684_NIES39_C03520                    --L----------------ATESPINSCVISSARPADGKSTVALNLAIGAAAMGQRV-LL
646131598_AplaP_010100019750               --L----------------ATESPINSCVISSARPADGKSTVALNLAIGAAAMGQRV-LL
640018815_L8106_14065                      --L----------------NPESPVRSCVISSATPADGKSTVAMNLAMGAAAMGQRV-LL
638107046_Tery_1925                        --L----------------NIENPINSLVISSALPGDGKSTVAGHLASAAAAMGQRV-LL
2506483316_LYNGBM3L_63940                  --L----------------GCDKPIQSIVISSARPADGKSTVAVHLAIVAAAMGRRV-LL
648856139_OSCI_1010012                     --L----------------TPGRSMRSLVIGSAVTGDGKSTIAFYLARTAAAVGLRV-LL
2508875360_Oscil6407DRAFT_00034810         --L----------------TPGRSMRSLVIGSAVTGDGKSTIAFYLARTAAAVGLRV-LL
2509510258_Osc10802DRAFT_3909              --L----------------SPQMPVRSLAIGSAAPGDGKSTVAIHLAKTAAAIGQRV-LL
2509438066_Mic7113_6231                    --L----------------SARKPIHSLVIGSAVPGDGKSTVAIHLAQTAASIGQRV-LL
2504090796_Osc7112_4249                    --L----------------SAGTAIRSLLVGSAVAGDGKSTVALHLAATAAAVGQRV-LL
2506345448_MicvaDRAFT_4285                 --L----------------SAGTAIRSLLVGSAVAGDGKSTVALHLAATAAAVGQRV-LL
2504091687_Osc7112_5131                    --L----------------NAEHPISSLTITSTIKGEGKSTVALYLAKTASAVGKRV-LL
647568459_MC7420_6195                      --L----------------SSKQPIHSLAICSPISGDGKTTVALYLAKAAATIGKRV-LL
647572642_MC7420_919                       --L----------------KSKKPIHSLAVSSAIPGDGKTTVAVYLAKTAAAIGQRV-LL
640014779_L8106_06284                      --L----------------SSHKSLNTLTISSATLGDGKSTIAIHLAQTAAFMGQRV-LL
2509421881_Oscil6304_2674                  --L----------------SIHKPIHSLVIGGAIPNVGKSTIAIHLARTAATIGQRI-LI
641253939_AM1_4861                         --L----------------NPDLPISSLVISSCSPQEGKSTTSMHLAQAAAAMGRRV-LL
2514735482_ACCM5_010100007602              --L----------------NPDLPISSLVISSCSPQEGKSTTSMHLAQAAAAMGRRV-LL
2510441931_Cha6605_5932                    --L----------------NPDLPIRSLVVSSALPNEGKTTIAIQLAQAAAAMGQRV-LL
2503611061_Chro_0810                       --L----------------DFNGTVRSLAIVSAAPEDGKSTVALYLAQTAAAMGKRV-LL
2503613235_Chro_2969                       --L----------------GFDKPIRSLAVISAAAEEGKSTVALHLAQTAAMMGQRV-LL
2512979625_Fis9431DRAFT_3954               --L----------------GSDAPIRSLVISSAGQGDGKSTVAVHLALAAAAMGHRV-LL
2517059612_PCC9339DRAFT_00465              --L----------------GSDTPIRSLVISSAGQGDGKSTVAVHLALAAAAMGHRV-LL
2505768848_FJSC11DRAFT_1825                --L----------------GSDAPIRSLVISSAGQGDGKSTVAVHLALAAAAMGHRV-LL
2516149265_FIS9605DRAFT_06272              --L----------------GSDAPIRSLVISSAGQGDGKSTVAVHLALSAAAMGHRV-LL
2517241827_Mas10914DRAFT_3070              --L----------------GSDTSIRSLVISSAGAEDGKTTVAIHLALAAAAMGQRV-LL
2507335136_Tol9009DRAFT_00051240           --L----------------GSDTPIRSLVISSAGQGDGKSTVAIQLAQAAAAMGQRV-LL
2504095135_Cal6303_2139                    --L----------------GSDAPIRSLVISSAQHGDGKSTVAVYLALAAAAMGQRV-LL
2507476798_Cal7103DRAFT_00036990           --L----------------GSDTPIRSLVISSAGQGDGKSTVAIYLALAAAAMGHRV-LV
2510085606_Riv7116_0460                    --L----------------GSDTPIRSLVVSSATPEDGKTTVAIQLALAAAAMGQRV-LL
2503739631_Nos7107_1000                    --L----------------GSDDPIRSLVISSAGQGDGKSTVAVHLAQAAAAMGQRV-LL
```

2505801704_Cal7507_3428	--L-----------------GADAPIHSLVISSAGQGDGKSTVAVQLAQAAAAMGQRV-LL
2505804241_Cal7507_5918	--L-----------------GSDSPIRSLVIGSATQGEGKSTVAIQLAQAAAAMGQRV-LL
643168344_AmaxDRAFT_0719	--L-----------------SPDQQIRSMVISSSVPGEGKSTTAIYLALAAAEQGRKV-LL
648390241_APCC8_010100025948	--L-----------------SPDQQIRSMVISSSVPGEGKSTTAIYLALAAAEQGRKV-LL
646129996_AplaP_010100011596	--L-----------------NPDQQIRSMVISSSVPGEGKSTTAIYLALAAAEQGRKV-LL
650383923_NIES39_A06230	--L-----------------NPDQQIRSMVISSSVPGEGKSTTAIYLALAAAEQGRKV-LL
640017922_L8106_10512	--L-----------------SPDAAIGSVVISSSQPGEGKSTVAIYLAQAAAEQGRRV-LL
2510101197_Gei7105DRAFT_2452	--L-----------------NPDTPVRSLTVSSPSLGDGKSTVAIHLAQAAAAMGQRV-LL
2509573281_Ple7327_1398	--L-----------------KSNPPIQSLVVSSASSEDGKSTVALYLAQTAASMGQRV-LL
2506597130_Spi9445_0041	--L-----------------SPDHWLRSLVITSSEPGEGKSTVAVHLAIAAAAMDQRV-LL
2503636620_PCC7418_1894	--L-----------------NPDVPVRAIVISSAMPQEGKSTVATHLAKAAAAMGRRV-LL
2506607995_Spi6313_0371	--L-----------------DADDPVRAVVISSATAGEGKTTIAAYLAQAVARMNRRV-LV
643585148_Cyan7425_2153	--F-----------------NIDTPIRSLTISSVCPNDGKTTVAINLARVAASMGHRV-LL
2504681848_Pse7367_3384	--L-----------------NPDEPIQSIVITSTAIGDGKSMFSAYLGQAAAAMGRRV-VI
647577962_S7335_3828	--------------------GSQDIAKSFSVSSAVPNEGKSTISFYLAHASASLGKRT-LL
2509846600_Lepto7375DRAFT_6617	--S-----------------SVEQPLTSVVVSSVMDNEGKSIAAIHLAEAAALMGQRV-LL
2509774530_Lepto7104DRAFT_1750	-RL-----------------DADRPLRSLVISSADNQLADSTTAAYLAWAAAELGNRV-LL
2509804417_LepboDRAFT_3759	--T-----------------NLQNPLHSIVVASALASDGKSTVAMNMALAAATMGQRV-LL
2531850144_CWATWH0003_3517	FAT-----------------NSDKKLKSLTISSAIPGEGKSAVAAFLAETAAKIGQKV-LL
2509777916_Lepto7104DRAFT_5137	--L-----------------SSDTPVQVVALTSSVPGEGKSTICAHLAIAAANMGRRV-LL
648856194_OSCI_1040007	--L-----------------FSDRQIGSLTIASAAPGDGKSTVALHLAEVAALAGQRV-LL
2508875344_Oscil6407DRAFT_00034650	--L-----------------FSDRQIGSLTIASAAPGDGKSTVALHLAEVAALAGQRV-LL
2504087727_Osc7112_1201	--L-----------------SSNRTISSLAIGSATVGEGKSTVALNLAHVAALAGQRV-LL
2506348042_MicvaDRAFT_3627	--L-----------------SSNRTISSLAIGSATVGEGKSTVALNLAHVAALAGQRV-LL
2509438406_Mic7113_6573	--L-----------------FSDPPVRSLAVCSAGHEDGKSTVALNLAQTAASMGQRV-LL
647571294_MC7420_5066	--L-----------------FSDRPIRSLSVTSAALGDGKSTVALHLAQTAAQMGQRV-LL
2506481927_LYNGBM3L_44990	--R-----------------FCNPPLRSIAICSAMAGDGKSSVALNLAHMAASRGQRV-LL
640019302_L8106_03117	--L-----------------YGDSPLRSLAVCSAESGDGKSMVALHLAQTIASMGQKV-LI
2509707887_Pleur7313DRAFT_01875	--R-----------------FTEPPIRSLVLSSAIEDDEQPTIAWNLADTAAATGQKV-LL
641251898_AM1_2808	---
2514737774_ACCM5_010100019113	--L-----------------PIEPPVRSLVISSVAARDGKTTVALYLAQSAARMGQRV-LL
641251897_AM1_2807	--L-----------------PIEPPVRSLVISSVAARDGKTTVALYLAQSAARMGQRV-LL
2512634931_Chl7702DRAFT_3806	--L-----------------AGVSKTQSIVVTSPEPGDGKSTVAMYLAQAAADMGKKT-LL
2516146228_FIS9605DRAFT_03233	--L-----------------AGAPKTQSLVVTSPAPGDGKSTIALHLAQAAAYMGKKA-LL
641675948_cce_1468	--Q-----------------EQGSNLRSVMISSAESEDGCSTIAVNLATNAAQKGQQV-LL
2507501641_Cy51472_2500	--Q-----------------EQGSNLRSVMISSAESEDGCSTIAVNLATNAAQKGQQV-LL
640626055_CY0110_29394	--Q-----------------EQGTNLRSLIVCSAEPEDGCSTIAVNLAINAAQKGQQV-LL
2531850936_CWATWH0003_4939	--Q-----------------EKVSKLRSLLLCSVESQDGCSTIALNLAINAAGKGQKV-LL
643476430_PCC8801_3415	--L-----------------PSKT-IRSLLITSVESKDGQSTVAMNLAISAATAGQRV-LL
644980660_Cyan8802_2701	--L-----------------PSKT-IRSLLITSVESKDGQSTVAMNLAISAATAGQRV-LL
643478515_PCC7424_0240	--L-----------------YNHPPLHSLVVSSVQAQDGQTTIALYLAKAAAATGKRV-LL
648186851_Cyan7822_1903	--I-----------------YNSPALHSLVVSSVQAQDGQSTVALYLAKAAAATGKKV-LL
2509575480_Ple7327_3597	--Q-----------------HSGSSLNSIAVSSTEAEDGQSTVALYLAKTVARTGKRV-LL
2508645044_GLO73106DRAFT_00012970	----------------------NESIKSLIICGVEPGDGQAFVAANLARVVADSGQKV-LI
2509773763_Lepto7104DRAFT_0983	--------------------------RSLVVTSALPNEGATTVAVHLVQAAAAMGLKV-LL
2503745907_Cyan10605_1646	--L-----------------TPNLPSKSVVISSSIPREGKSTISLNLAKVATSMGQKV-LL
2509874395_Syn6308DRAFT_1102	--L-----------------NSNSVVNSLVISSPNSMEGKSTISLNLAQTAVAMGRKV-LL
644979531_Cyan8802_1534	---
2503745344_Cyan10605_1086	--L-----------------SSDTSIRSIVVGAAEASEGKSTTSLFLAKAAALLGQKV-LI
2509873750_Syn6308DRAFT_0457	--L-----------------SLDRTVSSITIGTVDAKQGQSIISIYLAKAATMIGKKV-LL
2505785711_Chr6712_1954	-EV-----------------ESNQNIHSLIITSAISGEGKSTVVLNLAQARAAMGTRV-LV
2509706287_Pleur7313DRAFT_00275	PEV-----------------SSDTSLKSIVITSAIPREGKSTVALNLARACASLGKRI-LL
640027738_N9414_07903	-------------------------------------SASTV-------------------
2506482929_LYNGBM3L_58920	--------------------SSDKALKAVTVTSSLPQEGKSTVSANLAAVIAQRGSKV-LL
637461200_glr3785	--------------------SSDRKLKVFVVSSSMPGEGKSTVATNLAKVVGGLNRKA-LL
2503887239_Lepto7376_1629	--L-----------------IEERNYKTFVVSAATVGDGKTTVAINWADTAAVMGQKV-LL
638113739_sync_0150	--L-----------------NTERPLKAVALTSSLPAEGKSLVNVLLAKTLAEMGQRV-LL
638963744_RS9917_02426	--L-----------------NSDRPLRAIALTSSLPAEGKSLVNVLLAKTLSEMGQRV-LL
2507493170_Syn8016_1895	--L-----------------NSDRPLRSIALTSSLPAEGKSLVNTLLAKTLAEMGQRV-LL
638960084_WH5701_13945	--L-----------------SSDRPVQSVDVSSSVPSEGKSLLIVLLAKTLSELGRRV-LL
650128418_SCB01_010100004274	--V-----------------RAGGNLRMLAVTSTASGEGKTTSVALLGQALADLGLKV-LL
650132168_SCB02_010100007823	--L-----------------RAGRSLRLLAVTSTASGEGKTTLVALLGQTLADLGLRV-LL
2508551611_Cyagr_1223	--L-----------------RADNNIRMVGITSSTQGEGKSTAVAVFSRTLADLGLKV-LV
640081072_P9515_13861	--L-----------------NSDNPIKVITMTSSIPKEGKSLINILLSKTLSEMDLKI-LQ
648050855_Aazo_3125	TDLK-----------------------SLMVTSALPDDGKSALTLGLAMSAARLHKKV-LL
2504129904_Anacy_0284	SDLK-----------------------SLMVTSALPDEGKSALALGLAMSAARLHKKV-LL
2506490031_Ana7108_0216	SDLR-----------------------SLMVTSALPDDGKSALALGLAMSAARLHKKV-LL
2509770736_CylstDRAFT_4696	SDLK-----------------------SLMVTSALPDDGKSALALGLAMSAARLHKRV-LL
642600344_Npun_R1070	ANLK-----------------------SLMITSALPDEGKSALALGLAMSAARLHKRV-LL
2505799741_Cal7507_1481	TNLK-----------------------SLMITSPLSDEGKSALALGLAMSAARLHKRV-LL
2509779661_Mic7126DRAFT_0411	ANLK-----------------------SLMITSALPDESKSALALGLAMSAARLHKRV-LL
2503739922_Nos7107_1290	SSLK-----------------------SLMITSALPDEAKSGMALGLAMSAARLHKRV-LL
640027761_N9414_00965	STLK-----------------------SLMITSALRDESKSALALGLAMSAARLHKRV-LL
637233217_alr2833	SSLK-----------------------SLMITSPLLDRGKSGLALGLAMSAARLHKRV-LL
646566603_Ava_1116	SSLK-----------------------SLMITSPLLDRGKSGLALGLAMSAARLHKRV-LL
2509808002_Nos7524_0433	SSLK-----------------------SLMISSPMVDEGKSALALGLAMSAARLHKRV-LL
2517241466_Mas10914DRAFT_2709	SSFK-----------------------SLMITSALSDDGKSALALGLAMSAARLHKRV-LL
647105946_CRC_01573	SDLK-----------------------SLMVTSALPDISKSSLTLGLAMSAARLHKRV-LL
647109494_CRD_02102	SDLK-----------------------SLMVTSALPDTSKSSLTLGLAMSAARLHKRV-LL
2507332201_Tol9009DRAFT_00021840	ASFK-----------------------SLMITSALPDEGKSALALGLAMSAARLHKRV-LL
2512975951_Fis9431DRAFT_0279	STFK-----------------------SLMVTSALPDEGKSGLALGLAMSAARLHKRV-LL
2517062640_PCC9339DRAFT_03498	STFK-----------------------SLMVTSALPDEGKSGLALGLAMSAARLHKRV-LL
2505767181_FJSC11DRAFT_4188	STFK-----------------------SLMVTSALPDEGKSGLALGLAMSAARLHKRV-LL
2516146836_FIS9605DRAFT_03842	STFR-----------------------SLMVTSALPDDGKSGLALGLAMSAARLHKRV-LL
2512634275_Chl7702DRAFT_3149	STYK-----------------------SLMVTSAVPDEGKSALALGLAMSAARLHKRV-LL
2510089385_Riv7116_4239	SAFK-----------------------SLMITSALPDEGKSALALGLAMSAARLHKKV-LL
2504094820_Cal6303_1825	AMLK-----------------------SLMVTSPLPDEGKSALALGLAMSAARLHKRV-LL
2507482515_Cal7103DRAFT_00094220	AALK-----------------------SLMVTSALPDDGKSALSLGLAMSAARLHKRV-LL

```
2503612562_Chro_2300                 AAFK----------------------SLVVTSAVAGEGKSTLALGLALSAARLHQRV-LL
2517695945_SYN7509DRAFT_0062         STLR----------------------SIMITSALAGEGKSTLALGLALSAARLHQRV-LL
2503608186_GEI7407_2184              SPLK----------------------SIVVTSALAGEGKSTLAMGLAMSAARLHQRV-LL
2504685208_Cri9333_2761              STLKDGEALDLIYKNVQLQKSTSVFSSLMITSALDGEGTSTIALGLAISAARLHQRV-LL
2509431928_Mic7113_0093              STLR----------------------SLMVTSAVAGEGKSTLALGLAISAARLHQRV-LL
2506479159_LYNGBM3L_28310            SELK----------------------SLMVTSALSGEGKSTLALGLALSAARLHRRV-LL
640017638_L8106_27951                KP-----------------------KSLLVTSALNGAGKSTLVVGLAISAARLHQRV-LL
2509806250_LepboDRAFT_5593           DPLK----------------------SLVVTSALAGEGKSTVALGLAISAARLHQRV-LL
2504583836_Pse7429DRAFT_1713         SPIR----------------------SLVITSALPSEGKSTIALGLATSAARLHKRV-LL
641251010_AM1_1910                   ---------------------KNPYKTLMITSAGAGEGKSTLALGLAISAARLDQRV-LV
2514735759_ACCM5_010100008979        ---------------------KNPYKTLMITSAGAGEGKSTLALGLAISAARLDQRV-LV
2509847582_Lepto7375DRAFT_7599       -----------------------PLSSLLVTSALMGEGKTTLSIGLALSAARSHQRV-LL
648051793_Aazo_4516                  -----------------------PFKSLMLTSALPGEGKTTLALGLGASAAQMHQRV-LV
2504134691_Anacy_5006                -----------------------PFKSLMLTSALPGEGKTTLTLGLGASAAHMHQRV-LV
2506494605_Ana7108_4733              -----------------------PFKSLMLTSAVSGEGKTTLALGLGASAAHMHQRV-LV
647107726_CRC_03451                  -----------------------PFKSLMLTSALPGEGKTTLAIGLGASAANMHQKV-LV
642604187_Npun_R5250                 -----------------------PFKSLMFTSALPGEGKTTLVLGLVASATRMHRRV-LV
2509767727_CylstDRAFT_1687           -----------------------RFKSLMLTSALPGEGKTTLALGLGASAAHMHRRV-LV
2507335141_Tol9009DRAFT_00051290     -----------------------AFKSLMVTSALPKEGKTTIAQGLALSAARMHRRV-LL
640028130_N9414_07219                -----------------------PFKSLMLTSALSREGKTTLALGLAASAARMHRRV-LL
637230414_all0059                    -----------------------PFKSMMVTSALPGEGRTTLAMGLAASAAHMHRRV-LL
646568157_Ava_2661                   -----------------------PFKSVMVTSALPGEGRTTLAMGLAASAAHMHRRV-LL
2509811005_Nos7524_3436              -----------------------PFNSLMITSAIPGEGKTTLALGLAASAAHMHQRV-LL
2503739635_Nos7107_1004              -----------------------HSKSLMFTSALSGEGKTTLLLGLAASAARMHRRI-LL
2509781657_Mic7126DRAFT_2408         -----------------------PVKSLLLTSTSVGEGKTTLALGLAASAAHMHQRV-LL
2517243237_Mas10914DRAFT_4480        -----------------------PYKSLMFTSALPGEGKSTLALGLAMSAARMHQRV-LL
2512634393_Chl7702DRAFT_3267         -----------------------SFKSLMITSARSQEGKTTLALGLAVSAARMHRRV-LL
2505769181_FJSC11DRAFT_2156          -------------------------KSVMLTSAQPKEGKTSIALGLAVSAARMHKRV-LL
2512977368_Fis9431DRAFT_1696         -----------------------ASKSVMLTSAQPKEGKISIALGLAVSAARMHKRV-LL
2517063601_PCC9339DRAFT_04460        -----------------------SSKSVMLTSAKPKEGKTSIALGLAVSAARMHKRV-LL
2516147062_FIS9605DRAFT_04068        -----------------------------LTSALPKEGKTTLALGVAVSAARMHRRV-LL
2507482923_Cal7103DRAFT_00098300     -----------------------PYKSLMVTSANFGEGKSTVTLGLAVSSARTHHRV-LI
2510085595_Riv7116_0449              -----------------------HCQTLMVTSALAGEGKSTATLGLAVSAARMHQRV-LV
2503614917_Chro_4637                 -----------------------SFKSLLLTSALTGEGKSTLAAGMALSVARLQRRV-LL
2504093446_Cal6303_0467              -----------------------PCKSLMVTCAGTGEGKSDLLVGFAVSAARMHQRV-LI
647578236_S7335_5395                 -------------------------KSLAITSTRSAESSSTLAVGLALSAARIGQRV-II
2503798844_Sta7437_0777              -----------------------AFKSLLLTSAVAGEGKSTLVLGLAFSIARHQKRV-LV
2509708827_Pleur7313DRAFT_02815      -----------------------PLQSLAVTSAIAGEGKSTLILGLAISAARHQQRV-LV
637009749_sll0923                    ----------------------RQCHSFVVTSAESGDGKSTTAFFLAQAAAKLGQKV-LL
2503800689_Sta7437_2597              -------------------GKI---NSLALTSSTSAEGKTTVTYNLGLVLAELGLRV-LI
2509710958_Pleur7313DRAFT_04946      -------------------GQL---KTLAVTSSTSAEGKSTISYNLSIVLAELDLRV-LI
2505786361_Chr6712_2601              -------------------GKI---KSLVLTSSTSSEGKSTTSYNLALALTDLGSRV-LL
2508650693_Xen7305DRAFT_00027920     -------------------RKI---KSLVTTSSTSSEGKTTVTYNLALALTELDAKV-LV
2506480412_LYNGBM3L_19610            -------------------NRI---KVLAFTSAMPSEGKTTITYHLGGVLAKLGQRV-LV
2509512444_Osc10802DRAFT_6095        -------------------GQV---KTLAFTSSTPSEGKSTLAYNIGRVLGELGHRV-LL
643587900_Cyan7425_4938              --------------------EL---RTIAFTSTIPSEGKSTIVYNLGRMLAELGQKV-LI
2504582150_Pse7429DRAFT_3127         --------------------MM---KVIAFTSSVPSEGKSTLTYNLAMILSSLGYRI-LL
2504679882_Pse7367_1451              --------------------SI---KSIAFTSAVPSEGKSTLVFNLASILAELGHKV-LV
2507088905_Pse6802_3551              --------------------SI---KSIAFTSALPAEGKSTLIYNLGCILAELDYRV-LV
2508687870_Syn7502_01070             --------------------TF---KTIAFTSSIPSEGKSTIIYNLSLTLAELGYKV-LL
2506748948_Syn7336_3628              -------------------GR---SKVLAITSAHPGEGKSTVACNLGKVLAEVGKRV-LM
643484053_PCC7424_5769               -------------------HSQSTKKIIAITSSVDGEGKTTITYNLGLALAELGQRV-LI
648199484_Cyan7822_5718              -------------------NPQITGKIIAVTSATTEEGKTTITYNLGLALTEIGKKV-LI
2510436294_Cha6605_0295              -------------------------KIIAMTSSLPKEGKSTIVFHTSNTLAELGYRV-LL
637314397_tll1767                    -------------------------QSLAIISALPGDGRTTVALHLALAAAGTGQRV-LL
637313820_tll1199                    -------------------------HALAVMGTSGQEGQTTIALYLALAAAAIGKRV-LL
2512980079_Fis9431DRAFT_4408         --------------------SDKQLKAITVTSSIPKEGKSEVSANLAVAMAEVERRV-LL
2517061743_PCC9339DRAFT_02599        --------------------SDKQLKAITVTSSIPKEGKSEVCANLAVAMAEVERRV-LL
2505770929_FJSC11DRAFT_1293          --------------------SYKQLKAITVTSSIPKEGKSEVSANLAVAMAQVERRV-LL
2516148164_FIS9605DRAFT_05171        --------------------SDQQLRAIAVTSSIPKEGKSEVSANLAVAMAQVGRRV-LL
2512634416_Chl7702DRAFT_3290         --------------------SDKQLKAIAVTSSVAKEGKSEVSANLAVVMAQVGRKV-LL
2505800360_Cal7507_2092              -------------------GSDEELKAIAVTSSVAKEGKSEVAANLAVAIAQLGRRV-LL
2509780096_Mic7126DRAFT_0847         -------------------NSDQEIKAIAVTSSVAKEGKSEVAANLAVAMAQLGRRV-LL
2505800523_Cal7507_2254              -------------------SSDQELKAIVVTSSVAKEGKSQVAANLAVAIAQLGRRV-LL
2507478259_Cal7103DRAFT_00051610     -------------------SSDHELRAFTITSSISKEGKSEVAANLALAMTQVGRRV-LI
637234831_all4432                    -------------------CSDKPLKSIVVTSSVPKEGKSEVSANLAVTMAQAGRRV-LL
646566875_Ava_1386                   -------------------CSDKPLKSIVVTSSVPKEGKSEVSANLAVTMAQAGRRV-LL
642600743_Npun_R1496                 -------------------CSDKPLKSVVITSSVGKEGKSDVSANLAVTMAQAGRRV-LL
2507334816_Tol9009DRAFT_00048030     -------------------CSDKPLKSIVVSSSVAKEGKSEVAANLAVAMAQLGRRV-LL
2507476704_Cal7103DRAFT_00036050     -------------------CSDKPLKSIVVTSAVAKEGKSEVAANLAVAMAEVGRRV-LL
2510090628_Riv7116_5482              -------------------CSDTQLRSIVVTSSVPKEGKSEVAANLAIAMASVGRRV-LL
2506483552_LYNGBM3L_64290            -------------------SSDRPLQSIVVTSCVPKEGKSHVCANLAAAMAQVGRRV-LL
2517697529_SYN7509DRAFT_1646         -------------------SSDKELKVVVVTSSIPQEGKSTLCANLAVTMAQSERRV-LL
2503608035_GEI7407_2035              -------------------SPDRELKVIVVTSPVPQEGKSTVSANLAAAMAQLGRRV-LL
2509433692_Mic7113_1857              -------------------SPDKELKVVVVTSSVSKEGKSTVSANLAVAMAQLGHRV-LL
640015133_L8106_09871                -------------------SPDHELNFIVVTSAVSQEGKSTISANLAAAMSQVGSRV-LL
2503795055_Glo7428_2485              -------------------SSDKPLKVIVVTSAVPKEGKSTVSANLAAAMAQLGRKV-LL
2509506717_Osc10802DRAFT_0363        -------------------SSDKPLKVIVVTSSVAKEGKSAVSANLAAAVAQLGRRV-LL
2503614965_Chro_4685                 -------------------SSDK-LKVILVTSSVPGEGKSTVSANLGAAIAQLGNRV-LI
2510091904_Riv7116_6758              -------------------SADKPLQVIVVTSSVPKEGKSTVSANLAAAIAQLGQRV-LL
2503612629_Chro_2367                 -------------------NLDRPLKAVVVTSSVPKEGKSTVCANLAVAIAQLGRRV-LL
2503612419_Chro_2158                 AKRKIKPSLSIGTFFSSLKNSNKAFKTIVVTSSISKEGKSTISANLAATMAEIGRKV-LL
2506493946_Ana7108_4087              -------------------SSEPPLKAIVVTSTVPKEGKSTVSANLAAAIAQLGRKV-LL
2509803997_LepboDRAFT_3339           -------------------SLDEKLKSVVITSSVPREGKSTIAANLAATMATLGRRT-LL
2512979777_Fis9431DRAFT_4106         -------------------S-HRKVRKIVVTSSVSAEGKSEVSANLAAVIAQSGKRV-LL
2517062202_PCC9339DRAFT_03059        -------------------S-HRKVRKIVVTSSVSAEGKSEVSANLAAVIAQSGKRV-LL
2505770300_FJSC11DRAFT_0671          -------------------S-HRKVRKIVVTSSVTAEGKSEVSANLAAVIAQSGKRV-LL
2507334440_Tol9009DRAFT_00044270     -------------------S-HKKVRTIVVTSSLTEEGKSEVCANLCGAIAQAGRRV-LL
```

2510088639_Riv7116_3493	-------------------S-HKEVRTIAVTSSVVGEGKSEVTANLGAILAQGGQRV-LI
2505803639_Cal7507_5320	-------------------SLDRKARTIVVTSSVSGEGKSEVTANLATVMAQAGRRV-LL
2509782388_Mic7126DRAFT_3139	-------------------SLDRKARTIVVTSSVSGEGKSEVSANLATVMAQAGRRV-LL
2507335607_Tol9009DRAFT_00055950	-------------------SLDRKVSAIVVTSSMSGEGKSEVAANLAAVMALAGRRV-LL
2510088989_Riv7116_3843	-------------------SLDKKVRSIAVTSSVAKEGKTEVAANLAVVTAQVGRKV-LL
2504096650_Cal6303_3648	-------------------S-HKQVRTIVVTSSVAGEGKSEVSANLAAAIAQTGKRV-LL
2507481772_Cal7103DRAFT_00086780	-------------------S-HKTVRTIVVTSSVPGEGKSEVSANLAAVIAQAGKRV-LL
2509803885_LepboDRAFT_3227	-------------------SLDKKVKTIVISSAVPREGKSEVAANLAATMAQSGRKV-LL
647579433_S7335_2031	-------------------TSRQELKTIVITSAAAGEGKSEVSVNLATTIAQSGQRV-LL
2509846113_Lepto7375DRAFT_6130	---------------------DTHVKTVTVTSAVGGEGKSEVAANLALTLAHLGHTV-LL
2512978854_Fis9431DRAFT_3183	-------------------SANKELKVIVVTSSVPREGKSTVAANLAMAMAQMERKV-LL
2516143706_FIS9605DRAFT_00708	-------------------SADKELKVIVVTSSVPKEGKSTVAANLAIAMAQMEHKV-LL
642600641_Npun_F1381	-------------------GGDKQLKVIVVTSSVPREGKSTVAANLAIVMAQMDHQV-LL
2509770219_CylstDRAFT_4179	-------------------SADKELKVIVVTSSVPREGKTTVATNLAVAIAQMECKV-LL
2504094091_Cal6303_1107	-------------------SADKELKVIVVTSSVPKEGKSTVAANLAMAIAQMERKV-LI
2507479320_Cal7103DRAFT_00062230	-------------------SADKELKVIVVTSSVAKEGKSTVAANLAMAIAQMERRV-LL
2510085390_Riv7116_0244	-------------------SADKELKVIVVTSSVPQEGKSTVAANLATSMAQMERRV-LL
642601572_Npun_F2453	--
2503611448_Chro_1193	-------------------NSDKQLKAIVVTSSVPKEGKSTVAANLAVTIAQMGRKV-LL
2503796978_Glo7428_4384	-------------------SSDKQIKVVVVTSSVPQEGKSTVSANLAASMAQLGRRV-LL
2504087786_Osc7112_1259	-------------------SSDKELKVIVVSSSLPQEGKSTVSANLAVAIAQLGRKV-LL
643584136_Cyan7425_1127	-------------------NSDGDLKVIAVTSSIPGEGKSTTCANLAIAMAQRGCRV-LL
647105865_CRC_01492	--------------------TDKELRVILLTSSSPGEGKSFISANLALAASHLGKRV-LI
647108284_CRD_00419	--------------------RDRELQVMLVTSSSQGEGKSFVSANLAAALSYLGKRV-LI
2505800344_Cal7507_2076	--------------------SDKKLKVIVVSSSIPGEGKSFVTANLAVAIAQLGRRV-LL
2509781889_Mic7126DRAFT_2640	--------------------SDRKLKVIVLSSSIPGEGKSFVAANLAVAATQMGRRV-LL
2507481279_Cal7103DRAFT_00081850	--------------------SDQQLRVIAVTSSVAGEGKSFVAANLAVAKVQMGYRV-LL
637233240_alr2856	--------------------SDKKLKVIVVSSCVMNEGKSFIAANLAVATAQMGRRV-LL
646566532_Ava_1045	--------------------SDKKLKVIVVSSCVMNEGKSFIAANLAVATAQMGRRV-LL
640028261_N9414_00005	--------------------SDKELKVIVVSSCLPGEGKSFVAANLSVATAQMGRRV-LL
642603803_Npun_R4851	--------------------SDKPLKVIAVVSSTPGEGRSFVAANLAVAKAHMGRRI-LL
2509770699_CylstDRAFT_4659	--------------------SDKPLKVIAVVSSTPGEGRSFVAANLAVAKAHMGRRI-LL
2507478691_Cal7103DRAFT_00055940	--------------------PDQVLKSIVITSAVPSEGKSFVSANLALAMAQLGRRV-LI
641249527_AM1_0407	-------------------SSDHHVQVFVVTSSIPKEGKSTVVANLALALGELGHRV-LV
2514736728_ACCM5_010100013856	-------------------SSDHHVQVFVVTSSIPKEGKSTVVANLALALGELGHRV-LV
643586770_Cyan7425_3796	------------------SSPDHPIRTIVITSTRPQEGKSTVSANLALAIAELGHKV-LL
2509555626_Dacsa_3240	------------------SSVDQKMKVIVLTSSIPSEGKSTISANLGMALAELGNKV-LI
2509775597_Lepto7104DRAFT_2817	-------------------SSDTSHRIIAITSSVSEEGKSEVSANLAATLAHSGKQV-LL
2506492920_Ana7108_3067	-------------------NSEQSIKVIVVSSSVPKEGKSTISANLAFSISQLGRNV-LL
2512631781_Chl7702DRAFT_0655	-------------------NLEQP-RVIVVSSSVPKEGKSTIAANLAFSMSQIGRKV-LL
2504685956_Cri9333_3503	-------------------SLDNQLKVMVISSSVPKEGKSTTAANLAVAISQLGQRV-LL
2510436704_Cha6605_0705	-------------------NADDSLKVVVVSSSQSGEGKSTVAANLALAVAELGKRV-LL
2507482822_Cal7103DRAFT_00097290	-------------------DSDQKPKVIVISSSIPQEGKSTVAANLATVFAELKKRV-LL
2510087184_Riv7116_2038	-------------------DSTQKSNVIVISSSVPQEGKSTVAANLAAVFAETKKRV-LL
2506745596_Syn7336_0315	------------------QTAIDRPTTVTLASSVPGEGKSTIAANLAMAVAELGRRV-LL
2503796286_Glo7428_3705	-----------------PPSLERSSRVILVTSSAPKEGKSTTCANLAAVMALLNYKV-LI
642604427_Npun_F5505	-------------------SSDKVLKTIVVTSAVPKEGKSTVSANLATAIAQLGRQV-LL
2503612394_Chro_2133	-------------------SPDRALRTIVITSAVPKEGKSTVSANLAAAIAQLGRKV-LL
2512632720_Chl7702DRAFT_1594	-------------------SSERVLKTIVVTSAVPKEGKSTVAANLAAAIAQLGRKV-LL
2509807906_Nos7524_0337	-------------------SSDKELKTIVVTSSVPKEGKSSVSANLALAISQMGRKV-LL
2509770373_CylstDRAFT_4333	-------------------SSDKVLKTIVVTSAVPKEGKSTVSANLAAAIAQLGRRV-LL
646570355_Ava_4846	-------------------SSDQVLKNLVITSALPREGKSTVSANLAAAIAQLGRRV-LL
2510438837_Cha6605_2838	-------------------SADKPLKIIAITSSVANEGKSKVAANLAAAIAGMGQKV-LL
2506477923_LYNGBM3L_10200	-------------------RSDHPLKVIVVTSSVSKEGKSTVSANLAAAMAQLGRRV-LL
2509574529_Ple7327_2646	-------------------SSDRQQKIIVVTSSVSKEGKSTVAANLAAAMAQVRHKV-LL
643170532_AmaxDRAFT_2895	-------------------GSDRKVRVIVVSSSVPQEGKSTVSANLAAAIAQLGHRV-IL
648389308_APCC8_010100021105	-------------------GSDRKVRVIVVSSSVPQEGKSTVSANLAAAIAQLGHRV-IL
646129623_AplaP_010100009700	-------------------GSDRKVRVIVVTSSVPQEGKSTVSANLAAAIAQLGHRV-IL
650384826_NIES39_C04940	-------------------GSDRKVRVIVVTSSVPQEGKSTVSANLAAAIAQLGHRV-IL
2503801641_Sta7437_3540	-------------------SLEHPPQVILVTSSVPEEGKSTVSANLATAIAQLGKRV-LL
2509711085_Pleur7313DRAFT_05073	-------------------TSDKPPKVILITSSVPEEGKSTVAANLAAAIAQLGRSV-LL
2503801821_Sta7437_3719	-------------------NQDNPPQVVLVTSSVPGEGKSTIAANLATAIAQLGRRV-LL
2505786500_Chr6712_2739	-------------------TAQRPPKVILITSSVPGEGKSSIAANLAAAISQLGRRV-LL
2508650623_Xen7305DRAFT_00027220	-------------------TSQRPPKVILVTSSIPGEGKSTVTANLAAAISQLGRKV-LL
2509706990_Pleur7313DRAFT_00978	-------------------SIKRQPKVILMTSSVPGEGKSTVTANLAAAMAQLGRKV-MI
2505787175_Chr6712_3399	-------------------SSQRSPKVILVTSSVPGEGKSTITANLAAAIAQLGRHV-LL
2508650743_Xen7305DRAFT_00028420	-------------------SSQVLPKVILVTSSVPGEGKSTVTANLAAAISQLGSRV-LL
2509711668_Pleur7313DRAFT_05656	-------------------DADAKKQVIAITSSVPQEGKSSVSANLAISRAQCGQKI-LL
2509712372_Pleur7313DRAFT_06363	-------------------DNEVANKVIVVTSAVSGEGKSFVSANLAVAQAQCDQRV-LL
2505785156_Chr6712_1404	-------------------QGQEAIKSIAVTSAVAKEGKTSVSANYASAQAQCGKRI-LL
2503799529_Sta7437_1457	-------------------SLSQERKVIAVTSCVPQEGKSHVSANLAIAQNQVGKRI-LL
2506749054_Syn7336_3731	-------------------QLDNAPQIIAVTSALPQEGKTTVAANLASVMAQNRYRT-LS
2503799932_Sta7437_1855	-------------------EHQEDLKSIVITSSIPQEGKSTVSSNLATAVAQLGKKV-LL
2505786145_Chr6712_2386	--------------------DDRDIKVVTVTSSIPKEGKSTVSANLAAALAQIKKKV-LI
641538163_MAE_41520	-------------------NPDKNPITVIVTSSIFGEGKSEVAANLALAKAELGSQV-LI
2507085801_Pse6802_0485	----------------NANCP---LKVILITSSVPSEGKSTVSANLAAIAAQLGKHV-LL
2507089189_Pse6802_3805	----------------KSDSSAKGVKVIVVTSSIAAEGKSTVAANLAATTAQLGRRA-LL
2508689585_Syn7502_02785	----------------NSD---RQIKAIVITSSVPGEGKSTVSANLAVAIAQLGKRV-LL
2504679565_Pse7367_1141	-------------------SLKKRLKVIVVTSSVPREGKSTIAANLAATIAELGQRV-LL
2503802282_Sta7437_4176	-------------------SCDQKIKTIVVTSSVSQEGKSTISANLAAAMAQVGHRV-LL
2509709361_Pleur7313DRAFT_03349	-------------------NSDATIKSVVVTSSSSQEGKSTVAANLAAAMAQVGNKV-LL
2508651492_Xen7305DRAFT_00035910	-------------------CSDKKINTVVITSSTSGEGKSSIAANLASAMAQVGKKV-LL
2505784713_Chr6712_0967	---------------------DRQIQTIVVTSSGSGEGKSTVAANLAAAMAQVGHKV-LL
2514739634_ACCM5_010100028418	-------------------SDDHSLKSLVVTSIDVQEGKSTVAANLAIAMAQLEQRV-LL
637461727_glr4310	-------------------SSERGLRAFVLTSASPQEGKSTTAANLAAVSAQAGARV-LL
641253915_AM1_4837	-------------------QTGRHCQTLVVTSTSPQEGKSTVVANLAMALAQAGNQV-LV
2514739407_ACCM5_010100027286	-------------------QTGRHCQTLVVTSTSPQEGKSTVVANLAMALAQAGNYV-LV
2509712141_Pleur7313DRAFT_06132	------------------NKANSEQKMIVVSSSLRGEGKSLVSANLALTLSQLGHKV-LI

```
2506482932_LYNGBM3L_58950                      -------------------------YCIKTTSKHWNNAE---------------------
2506608339_Spi6313_0712                        -------------------NREQKARILAVTSSIPGEGKSVTAANLAISLSMLSHRV-LL
641249511_AM1_0391                             -------------------GLEHSPRVLMMTSAINQEGKSTTCANLAVSLAQMNHRV-LL
2514736711_ACCM5_010100013771                  -------------------SLELSPRIIMVTSAITQEGKSTTCANLAASLSQMNHRV-LL
2509500847_Pro9006DRAFT_2342                   ------------------KGWGNQLRSLAVLSTIGQEGRSEIVANLAATTAQGRRRV-LI
2504681110_Pse7367_2660                        -------------------EPGKAPKVIAITSSIAKEGKSTFTANLAIAAAQMGAKV-LL
2509874159_Syn6308DRAFT_0866                   ---------------------EKPLKVITISSTVAKEGKSTISANIALAAAGIGKKV-LL
2509428796_Syn6312_0755                        ------------------SNSDGKLKVITITSSTQGEGKSTVTANLGLAMTEIGYRV-AI
2508653159_Xen7305DRAFT_00052600               --------------------SQNRIRTIVITSCTAQEGKSSIAANLAHIMAQKGENV-LL
2512978631_Fis9431DRAFT_2960                   --------------RSTEPL-----RSIVVSSTVTGEGKSVVASHLAVISAMLSRRT-LL
2517061062_PCC9339DRAFT_01917                  --------------RNPESL-----RSIVVSSTITGEGKSVVASHLAVISAMLSRRT-LL
2505768802_FJSC11DRAFT_1780                    --------------RSSEPL-----RSIVVSSTITGEGKSVVASHLAAISAMLSRRT-LL
2516146713_FIS9605DRAFT_03719                  --------------RSTESL-----QLIVVSSTISGEGKSVVASHLAAVSAMLSRRT-LL
2507336787_Tol9009DRAFT_00067760               --------------RSNDKV-----QIIVVSSTLSGEGKSIVASHLAAISAMLSRRT-LI
2517242598_Mas10914DRAFT_3841                  --------------RSLEQL-----RLIVVSSPISGEGKSVVASHLAAVAAMLSWRT-LI
2507476104_Cal7103DRAFT_00030040               --------------RTSE-L-----QLLVVSSTISGEGKSIVASHLAAVSAMLSRRT-LI
2510090822_Riv7116_5676                        --------------RSDDEL-----QKIVVSSTISGEGKSVVVSHLAAVSAMLSRKT-LV
2509766470_CylstDRAFT_0429                     --------------RHTQKL-----RSIVISSTIPGEGKSIVVSHLAAVSAMLSRRT-LI
637233445_alr3059                              --------------RNVDNL-----QVIVVSSPLSGEGKSVIVSHLAAVAAMLSRRT-LI
646566337_Ava_0852                             --------------RNVDNL-----QVIVVSSPLAGEGKSVIVSHLAAVSAMLSRRT-LI
2509812587_Nos7524_5019                        ------------------------------------------------------------
2507480867_Cal7103DRAFT_00077730               --------------RSASKL-----RSIVVSSALSGEGKSVVVSHLAAVSAMLSRRT-LL
2504094392_Cal6303_1407                        --------------STAENL-----HLFVVSSTISGEGKSIVTSHLGAISAMLSRRT-LL
2506481239_LYNGBM3L_38240                      --------------RAQKRL-----QLIVVSSAVSGEGKSVVASHLGAVSVRFSRRT-LI
2503613794_Chro_3525                           --------------AANEDI-----KVIVVSSALSGEGKSLVSSYLAAVAATLSRRT-LL
2510437494_Cha6605_1495                        --------------DRGTQI-----RRLVIGSALASEGKSMVAAHLAMVAAMQSRRT-LL
2509773567_Lepto7104DRAFT_0787                 --------------QCSQRRLDGAGRTVVFTSMSPGEGKSATAIYLGAIAAMLSRRS-VI
641254791_AM1_5726                             -------------------KNSTESKVLVITSAISGEGKSFVATNLAVLSAMLSKRT-LL
2514737653_ACCM5_010100018486                  -------------------KNSTESKVLVITSAISGEGKSFVATNLAVLSAMLSKRT-LL
637459597_gll2202                              -----------------------KAAVIVVSSAVANEGKSTVATNLARAMARAGRRT-LV
gi|4512007|gb|AAD21564.1|                      -------------------------NVLMISGASPSAGKTFISSNLAATIAITGKKV-LF
gi|53987110|gb|AAV27324.1|                     -------------------------NILMISGPSPEIGKTFVSTNLAGVVAQAGQKV-LL
637230853_all0493                              -------------RIGGR----NL-KVVLVTSTSSQEGKTTSAYNLGIASARAGKRT-LI
646568404_Ava_2908                             -------------RIGGR----NL-KVVLVTSTSSQEGKTTSAYNLGIASARAGKRT-LI
2509812367_Nos7524_4799                        -------------RIGGK----NL-KVVLMTSTSSQEGKTTSAYNLGVAAARAGKRT-LI
642599777_Npun_F0459                           -------------RIGGK----NL-KVVLITSTSSLEGKTASAYNLGIASARAGKRT-LI
2509772030_CylstDRAFT_5990                     -------------RIGGK----NL-KVLLITSVSSQEGKTASVYNLGIASARAGKRT-LI
2503740929_Nos7107_2283                        -------------RLAGK----DV-KILLIASTGAQEGKTVSAYNLGVACARAGKRT-LI
640027950_N9414_23213                          -------------RIGGK----KF-KMLLITSTSSQEGKTVTAYNLGIASALAGKRT-LI
2505798270_Cal7507_0021                        -------------RIGGK----NL-KVVLIASTVSSEGKTATAYNLGIASARAGKRT-LI
2509781264_Mic7126DRAFT_2015                   -------------RIGGK----QL-KVILITSVSSTEGKTASAYNLGIASARAGKRT-LI
2507337082_Tol9009DRAFT_00070720               -------------RIGGK----NL-KVVLITSTSSSEGKTVSAYNLGIASARAGKRT-LI
2517243673_Mas10914DRAFT_4916                  -------------RIGGK----NL-KVVLITSTSSSEGKTVSAYNLGIASARAGKRT-LI
2504133191_Anacy_3523                          -------------RIGGN----DV-KVILITSVSSQEGKTVSAYNLGIASALAGKRT-LI
2506492757_Ana7108_2905                        -------------RIGGQ----NL-KVILITSVSSQEGKTVSAYNLGIASALAGKRT-LI
648049213_Aazo_0828                            -------------RIGGK----DA-KVILITSVSSQEGKTLSAYNLGIASARAGKRT-LI
2512978956_Fis9431DRAFT_3285                   -------------RFGSK----NL-KVILVTSISSSEGKTTTAFNLGIASARAGKRT-LI
2517059344_PCC9339DRAFT_00197                  -------------RFGSK----KL-KVILITSVSSSEGKTTSAYNLGIASARAGKRT-LI
2505768976_FJSC11DRAFT_1951                    -------------RLGGK----NL-KVILITSVSSSEGKTVSAYNLGIASARAGKRT-LI
2516145655_FIS9605DRAFT_02658                  -------------RLGGK----DL-KVILIASVSSDEGKTVSAYNLGIAFARAGKRT-LI
2512633064_Chl7702DRAFT_1938                   -------------RIGGQ----DL-KVVLIVSTASCEGKTVSAFNLGIASARAGKRT-LI
2510085904_Riv7116_0758                        -------------RIGGR----KV-KVVLITSTSSLEGKTTSAYNLGIASARAGKRT-LI
2504093110_Cal6303_0131                        -------------RIGGR----NF-KVVLITSTGAAEGKTTCAYNLGIASARAGKRT-LV
2507480421_Cal7103DRAFT_00073250               -------------RVGGS----NI-KVVLVTSTGASEGKTVSAYNLGIACARAGKRT-LI
2503609163_GEI7407_3152                        -------------RAGET----PA-KLVLFTSALDGEGKTVTAYNLAIASAHAGKRT-LI
2509421114_Oscil6304_1907                      -------------RIGEK----AP-KVLLITSVAPKEGKTFCAYNLAIAAARAGKRT-LL
2509805590_LepboDRAFT_4932                     -------------LTETK----NL-RVLMMISIVDGEGKTVTAYNLAIASARAGKRT-LL
646129274_AplaP_010100007922                   -------------SNGQK----PP-KVVLMTSTKGPEGKTLTAYNLAIASARAGKRT-LI
650387413_NIES39_K02750                        -------------SNGQK----PP-KVVLMTSTKGPEGKTLTAYNLAIASARAGKRT-LI
643168837_AmaxDRAFT_1210                       -------------SNGQK----PP-KVVLMTSTKGPEGKTLTAYNLAIASARAGKRT-LI
648390350_APCC8_010100026493                   -------------SNGQK----PP-KVVLMTSTKGPEGKTLTAYNLAIASARAGKRT-LI
640015518_L8106_06195                          -------------RSGDK----PA-RVVLITSAGDLEGKTFCAYNLAIATARAGKRT-LL
648859640_OSCI_3640012                         -------------RVGEK----PP-KLVILTSATALEGKTFSAYNLAIASARASKRT-LL
2508873356_Oscil6407DRAFT_00014730             -------------RVGEK----PP-KLVILTSATALEGKTFSAYNLAIASARASKRT-LL
2504092741_Osc7112_6177                        -------------RIGNK----PV-KVVLLTSAAPLEGKSFSAYNLAIASARSGKRT-LL
2506346135_MicvaDRAFT_1862                     -------------RIGNK----PV-KVVLLTSAAPLEGKSFSAYNLAIASARSGKRT-LL
638107751_Tery_2688                            -------------LQGSQ----AP-KVVLLSSGVKGEGKTFCAYNLAIAAARAGKRT-LL
2506480833_LYNGBM3L_31450                      -------------LREEP----AV-KVVLVVTSTAGNEGKTIVAYNLAIASAQAGMRT-LL
647565613_MC7420_2957                          -------------S-GEH----KSLKVVLIASTVKGEGKSTTAYNLAIASAHAGKRT-LL
2510102323_Gei7105DRAFT_3578                   -------------RVEG-----GAPKVVLITSAKAGEGKTFTAYNLAIASARAGKRT-LL
2509498590_Pro9006DRAFT_0077                   -------------RLGERG-----ARVILVSSPGVQEGKTLTAYNLAMATAQVGKRV-LV
2509773034_Lepto7104DRAFT_0253                 -------------RVGSLSEAGVAPRVVLVVSSRGGEGKTTSAFNLGIAAARAGRRT-LV
2517693907_LEP6406DRAFT_3365                   -------------LAGAQLQAGKPPQMIMVTSTRDQEGKTITAFNLAIAAARAGRRT-LI
2509844778_Lepto7375DRAFT_4795                 -------------LTGFE-DSVIGPRVVLVTSTISNEGKSITAYNLAIAAAQAGRRT-LL
641676481_cce_1994                             -------------RLGS-----DMSKVVMITSVSNEEGKSATAYNLAIASAQAGKRT-LL
2507502153_Cy51472_3013                        -------------RLGS-----DMSKVVMITSVSNEEGKSATAYNLAIASAQAGKRT-LL
640626984_CY0110_27525                         -------------RLGS-----DMSKVVMITSVSNEEGKSATAYNLAIASAQAGKRT-LL
2503335332_CWat_WH8501_draft2_00062360         -------------RLGS-----DVSKVVMITSVSNEEGKSATAYNLAIASAQAGKRT-LL
2531849533_CWATWH0003_2441                     -----------------------------ITSVSNEEGKSATAYNLAIASAQAGKRT-LL
643475556_PCC8801_2514                         -------------RLTP-----DTSNVILITSISNEEGKSVTAYNLAIASAHAGKRT-LL
644981523_Cyan8802_3592                        -------------RLTP-----DTSNVILITSISNEEGKSVTAYNLAIASAHAGKRT-LL
2509576278_Ple7327_4396                        -------------RLAS-----ESCKVILITSIAGDEGKTVTAYNLAIASALAGKRT-LL
641537721_MAE_37160                            -------------LVGGG----ETVKVVVVTSITGGEGKTATAYNLAIAASLAGRRT-LL
648188704_Cyan7822_3790                        -------------RFGS-----ASSKVIIVTSIINEEGKTANAYNLAIASAQAGKRT-LL
2503801406_Sta7437_3306                        -------------RFAP-----QSAKVILITSVSSDEGKSVTAYNLAIASAHAGKRT-LL
2505785267_Chr6712_1514                        -------------RYSS-----SATKVILVASVSNREGKSVTAYNLAIASAHAGKRT-LI
2506598905_Spi9445_1800                        -------------RLSP-----KSTKVILVTSVSQGEGKSVVAYNLAIASAHAGKRT-LL
2506609325_Spi6313_1684                        -------------RVAG-----PQGKVVLMTSVEENEGKSLTSYNLAIASAHAGQRT-LL
```

```
2503367585_Cyast_2162                   -------------RLGGEN-----AKVILISSVIADEGRTVTAYNLAIASANAGKRT-LL
gi|16124419|ref|NP_418983.1|            ------------------------VQVIAVTSSLPGEGKTTTTFSLARTLATSGAKV-IV
gi|16264834|ref|NP_437626.1|            -------------------------RVIAIASALPDEGKSIIAANFAALLAASGKRT-LL
2508652600_Xen7305DRAFT_00047000        -----------------QISSNQVIKTITVTSSNTAEGKSTFIANLAALTTQLGKKV-LI
2506480821_LYNGBM3L_31350               -------------------------RRILITSAMVSEGKTTVTLGLATALVDLGFRV-LV
2509432410_Mic7113_0575                 -------------------------HRLLITSAIMGEGKTTVTLGLARALADLGFRV-LL
2507482189_Cal7103DRAFT_00090960        -------------------------RRLLVTSAIMGEGKTTVAIGLANALVDLGFKV-LL
2509801296_LepboDRAFT_0638              -------------------------IRLLIASAITGEGKTTVALQLAHALTELGFRV-LL
2505802696_Cal7507_4407                 -------------------------RRLLITSAMEGEGKTTVTLGLARALVDLGFRV-LV
2507332342_Tol9009DRAFT_00023250        -------------------------RHLLITSAMEGEGKTTVTLGLARALVDLGFRV-LM
2510087202_Riv7116_2056                 -------------------------RHILITSAMESEGKTTVTLGLGKALVELGFRV-LV
2510089901_Riv7116_4755                 -------------------------GRLMIASANTEEGKTTTTIGLANALLVLGFKV-LL
2506745681_Syn7336_0399                 -------------------------RYLMVASATAGEGKTTTVLGLATALSSLGFRV-LL
637457826_glr0454                       -------------------------RRLMITSSGVGEGKTTVTLGLAKALRELGFRV-LV
637459188_gll1796                       -------------------------RRLMITSANTGEGKTTVTLGLAKALRELGFRV-LV
637461130_gll3716                       -------------------------RRLMITSSTAGEGKTTLTLGLANALAEMGFRV-LL
2503610992_Chro_0741                    -------------------------PCLMVTSATAGEGKTTVTLGLALALVNFGFRV-LV
2503793842_Glo7428_1287                 -------------------------QRILITSPTAGEGKTTMTLGLALALVNCGFRV-LM
2505800320_Cal7507_2052                 -------------------------RRLMVSSSTPGEGKTTVTLGLAAALSFLGFRV-LI
643588131_Cyan7425_5174                 -------------------------PRLMITSAMYGEGKTTIAIGLAAALARLGFKI-LL
643167924_AmaxDRAFT_0301                ---------------------------FAISSGKGGVGKSTVAVNVAVALASTGAKVGLI
648389440_APCC8_010100021805            ---------------------------FAISSGKGGVGKSTVAVNVAVALASTGAKVGLI
646130866_AplaP_010100015993            ---------------------------FAISSGKGGVGKSTVAVNVAVALASTGAKVGLI
650384994_NIES39_D01080                 ---------------------------FAISSGKGGVGKSTVAVNVAVALASTGAKVGLI
640015631_L8106_17697                   ---------------------------LAISSGKGGVGKSTVAVNVAVALAQMGAKVGLI
647567948_MC7420_2316                   ---------------------------VAISSGKGGVGKSTVAVNVAVALAQTGAKVGLI
638106387_Tery_1165                     ---------------------------IAISSGKGGVGKSTVAVNVAVALAQMGAKVGLI
648857672_OSCI_2870002                  ---------------------------LAISSGKGGVGKSTIAVNVAVALAQLGAKVGLL
2508875395_Oscil6407DRAFT_00035160      ---------------------------LAISSGKGGVGKSTIAVNVAVALAQLGAKVGLL
2509801138_LepboDRAFT_0480              ---------------------------IAISSGKGGVGKSSVAVNVAVALAQAGAKVGLI
2512979571_Fis9431DRAFT_3900            ---------------------------IAISSGKGGVGKSTVAVNIAVALAQAGAKVGLL
2517061293_PCC9339DRAFT_02148           ---------------------------IAISSGKGGVGKSTVAVNIAVALAQAGAKVGLL
2505768399_FJSC11DRAFT_3149             ---------------------------IAISSGKGGVGKSTVAVNVAVALAQAGAKVGLL
2509782632_Mic7126DRAFT_3383            ---------------------------IAVSSGKGGVGKSTVAVNIAVALAQTGAKVGLL
2504683355_Cri9333_0934                 ---------------------------LAVSSGKGGVGKSTVAVNIAVALAQLGAKVGLI
2503606101_GEI7407_0138                 ---------------------------IAVSSGKGGVGKSTVAVNLAVALAKAGSKVGLI
2509502622_Pro9006DRAFT_4121            ---------------------------IAVSSGKGGVGKSTIAVNLAVALAQAGAKVGLV
2509778293_Lepto7104DRAFT_5514          ---------------------------IAVSSGKGGVGKSTVAVNLAVSLAQMGSAVGLI
2517694920_LEP6406DRAFT_4378            ---------------------------FAVSSGKGGVGKSTVAVNIAVALAQAGAKVGLI
647577822_S7335_3227                    ---------------------------LAVTSGKGGVGKSTVAVNLAVALAKTGAAVGLI
2503637469_PCC7418_2732                 ---------------------------VAVSSGKGGVGKSSVAVNVAVSLAQTGAKVGLL
2509552467_Dacsa_0081                   ---------------------------IAVSSGKGGVGKSSVAVNLAVSLAQSGAKVGLL
2503367528_Cyast_2106                   ---------------------------IAISSGKGGVGKSTVAVNVAIALAKAGSKVGLL
2503887475_Lepto7376_1861               ---------------------------IAISSGKGGVGKSSVAVNVAIALAQSGAKVGLL
637688026_PMN2A_1153                    ---------------------------IAISSGKGGVGKSTVAVNLACALSQKGFKVGLL
640085320_NATL1_20271                   ---------------------------IAISSGKGGVGKSTVAVNLACALSQKGFKVGLL
641286083_P9211_17011                   ---------------------------VAVTSGKGGVGKSTVAVNLACALAQKGLKVGLL
640547039_SynRCC307_2084                ---------------------------IAVSSGKGGVGKSTVAVNLACALAQQGLRVGLL
637798215_PMT9312_1673                  ---------------------------IAVSSGKGGVGKSTIAVNLACSLAKLGSKTGLL
640161373_P9301_17721                   ---------------------------IAVSSGKGGVGKSTIAVNLACSLAKLGLKTGLL
640944474_P9215_18531                   ---------------------------IAVSSGKGGVGKSTIAVNLACSLAKLGLKTGLL
647672827_P9202_63                      ---------------------------IAVSSGKGGVGKSTIAVNLACSLAKLGLKTGLL
640079518_A9601_17881                   ---------------------------IAVSSGKGGVGKSTIAVNLACSLAKLGLKTGLL
637450610_PMM1581                       ---------------------------IAISSGKGGVGKSTIAVNIACSLAKLGLKTGLL
640081464_P9515_17681                   ---------------------------IAISSGKGGVGKSTIAVNIACSLAKLGLKTGLL
646569576_Ava_4074                      ---------------------------LGISSGKGGVGKSTTAVNIAAALSLQGAKVGLL
2505803290_Cal7507_4972                 ---------------------------LAVSSGKGGVGKSTTAVNIAAALRLQGAKVGIL
2517700475_SYN7509DRAFT_4594            ---------------------------LAISSGKGGVGKSTTSVNLAVALSLQGFKVGLL
2506747473_Syn7336_2171                 ---------------------------LAISSGKGGVGKSTTAVNLAVALSLKGAKVGLL
gi|1172092|gb|AAA86371.1|               ------------------------------------------------------------
gi|15597431|ref|NP_250925.1|            --------------------------TLGLTSARHGEGVTFIIEQLRRLLEENGVRVRI-
637450246_PMM1225                       NFVVFQKSIEEYCIGIKNLNESKNFKTFFLASPFSQEVKTLVNVLSSKILSSTNERV-IL
gi|53721457|ref|YP_110442.1|            -------------------------------------GRQAV------------ERARRS
638961258_RS9917_04770                  ------------------------------GELAPQAWSGPIQLLADGPLAASGLGS-GG
639888778_RS9916_38577                  ------------------------------DQTIQKSWIEPIKLIAQGPLLA--------
637444523_SYNW0419                      --------------------------------MAPEQWSSTAQLLAEGPLA---------
640545089_SynRCC307_0169                ------------------------------------------------------------
640084152_NATL1_08771                   --------------------------------VQKQSWTESIDLVISGPLSEI-------
647672433_P9202_965                     ------------------------------DGNFKKYTKD------------IFFEE-II
637797888_PMT9312_1355                  -------------------------------------SKTNI------------KKI-SL
640944066_P9215_14561                   ------------------------------TFSLDDKGKWEESINLILKEESFKKNIPIL
gi|397405|emb|CAA52655.1|               -----------------------------------------IACWLLVCCLLFGTLKLLL
gi|455023|gb|AAB51624.1|                -----------------------------------------IACWLLVCCLLFGTLKLLL
gi|1107924|emb|CAA62141.1|              ---------------------------VLVRFAFRKAGKNH-------------------
gi|53720415|ref|YP_109401.1|            -----------------------EYQRLALEQSFGEKLLASALTSLEQARADAERKQIYL
gi|45297|emb|CAA38731.1|                -----------------------DYQRLMLANELAQQQLAAAMTSLQNTRGEADRQQLYL


2505798636_Cal7507_0387                 VD-ADLRRPQLH--NLSDLNN-------------VQGLSSVISTNL-----------PVD
2509783408_Mic7126DRAFT_4160            VD-ADLRRPQLH--NLSELNN-------------VQGLSSVISTNL-----------PVD
648052066_Aazo_4925                     VD-GDLRRAQVH--KLSNLQN-------------LSGLSNVLTSNM-----------PVE
2504133443_Anacy_3769                   VD-ADLRRAQVH--RLSNLHN-------------LSGLSNVIASNM-----------PVE
2506494313_Ana7108_4449                 ID-ADLRRPQVH--KVSNLHN-------------LSGLSNVISSNM-----------PVE
2512980750_Fis9431DRAFT_5079            VD-ANLRQPVIH--ELANLSN-------------FWGLSNLITTNL-----------PIT
2517062414_PCC9339DRAFT_03271           VD-ANLRQPVIH--ELANLSN-------------FWGLSNLITTNL-----------PIT
2505767894_FJSC11DRAFT_3537             VD-ANLRQPVIH--ELANLSN-------------FWGLSNLITTNL-----------PVT
2516145585_FIS9605DRAFT_02588           VD-ANLRQPVIH--SLSDLNN-------------SWGLSNLISTNL-----------PVM
2504096080_Cal6303_3081                 VD-ADLRQPQIH--QLANLNN-------------LWGLSNLISTNL-----------PLN
```

```
2507333046_Tol9009DRAFT_00030320        VD-ADLRQPTIH--TLSNLNN-------------LWGLSNLISTNL-----------PVG
2507476338_Cal7103DRAFT_00032380        VD-ANLRQPAIH--TLANLNN-------------LWGLSNLISTNS-----------PIE
2505802134_Cal7507_3852                 VD-ANLRQPVIH--TLSDLNN-------------LWGLSNLISSNL-----------PIG
2509782674_Mic7126DRAFT_3425            VD-ANLRQPIIH--KLLGLNN-------------SWGLSNLITSNL-----------PVG
2509771979_CylstDRAFT_5939              VD-ADLRQPKIH--TLSDLNN-------------LWGLSNLITTNL-----------PVG
2503738747_Nos7107_0132                 VD-ADLRRPTIH--TLSNLSN-------------LWGLSNLISTNL-----------PVG
2509809495_Nos7524_1926                 VD-GDLRQPKIH--ELSNLNN-------------LWGLSSLITSNL-----------PVE
640026730_N9414_07896                   VD-GDMRRPTIH--TLSNLKN-------------LWGLSSLITSNL-----------PFE
2517239228_Mas10914DRAFT_0471           VD-ADLRRPRIH--ALSNINN-------------LWGLSSVISGSM-----------PVE
2510087477_Riv7116_2331                 VD-TDMRRPDIH--NLASLNN-------------HYGLSNLISGNI-----------NLE
2510087323_Riv7116_2177                 ID-TDLRRPQVH--ARTGLPN-------------LWGLSNLISGNM-----------PAE
2507477177_Cal7103DRAFT_00040780        VD-GDMRRSQVH--NQAGLNNS------------LWGLSSLISTNM-----------EAE
637235631_all5222                       VD-VNLRQPMIH--NLANLKN-------------LWGLSSLLSTNL-----------PVS
2506482271_LYNGBM3L_49700               VD-ADLRKPQVH--PRLQLSNQ------------L-GLSNLILQTLSP----------E
641611510_SYNPCC7002_A1500              VD-SDMRRPQVA--QRLNL-NQ------------KQGLSTVITNNSNETE--------ID
2509842234_Lepto7375DRAFT_2251          VD-ADLRRPTVH--HRLGLSNK-------------TGLSDLLSGNVTRG----------S
647107641_CRC_03354                     VD-TDLRLPQIH--KRLNLSN-------------LVGLSNAITSNL-----------TPD
647110196_CRD_02439                     VD-ADLRVPKQH--KRFNLPN-------------VVGLSNMITSNL-----------AVQ
2509804452_LepboDRAFT_3794              VD-TDLRSPQIA--DRLEVSNE------------QGLTELIARNLNP-----------Q
647567980_MC7420_2223                   VD-ADLRRPQLH--VRTDLPNV-------------WGLSHVISSD-----------INVK
2509437593_Mic7113_5758                 VD-ADLRRPRIH--ETTDLPNV-------------WGLSNVISSD-----------IHVD
2506479760_LYNGBM3L_56010               VD-ANLRFPQIH--QKMDLPNL-------------WGLSDVLSSE-----------IKVD
647567242_MC7420_5618                   VD-ADLRRPQVH--HYLNLDNQ-------------VGLSNVIATG-----------LTAK
2509436287_Mic7113_4452                 VD-ADLRLPQVH--KVLGLENQ-------------IGLSNVIATG-----------CTAK
2503796318_Glo7428_3737                 VD-ADLRLPQVH--NKLNLPNE-------------AGLSNVITTS-----------LPVD
2517697153_SYN7509DRAFT_1270            VD-ADMRLPQIH--TRLGLPNE-------------QGLSNIISTN-----------LPIY
2503615076_Chro_4796                    VD-ADLRLPQIH--HILHLSND-------------TGLSNAITTN-----------VPVA
2509422734_Oscil6304_3527               VD-ADLRRPQID--QRMDLPNV-------------WGLSNLISTDS----------LNAQ
2506601543_Spi9445_4406                 VD-ADLRRPQVH--VMTDLPNV-------------WGLSHAISMDIE-----------VE
2506609271_Spi6313_1630                 VD-ADLRRPQVH--AMTDLPNV-------------WGLSHVISMDLE-----------VD
640014711_L8106_15385                   VD-ANLRLPQVH--DQFNLANE------------F-GLSNLINTDVDP-----------D
2509507761_Osc10802DRAFT_1411           VD-ADLRWPYLH--DLLGLPNK-------------RGLSDVVATALAP-----------E
2509420889_Oscil6304_1682               VD-ADLRWPQVH--KRLGLPNR-------------QGLTHLIATEMD-----------MN
2503798162_Sta7437_0103                 ID-ADLRRPQVH--RWMGLENK-------------DGLSNVLATGLD-----------VN
2505785430_Chr6712_1674                 ID-ADLRRPQVH--RWTGLNNE-------------KGLSNLLSAPLRGEAEEQELKNLIE
2509711143_Pleur7313DRAFT_05131         VD-ADLRRPQVH--RWIGVENQ-------------EGLSNILATGLD-----------VE
2508648278_Xen7305DRAFT_00003760        ID-ADLRRPQVH--LWTGLENK-------------SGLSNILATGLP-----------VE
2508646941_GLO73106DRAFT_00031960       ID-ADLRRPQVH--HRLGVENQ-------------KGLSNVIAMNSDW-----------R
2509573356_Ple7327_1473                 ID-ADLRRPQVH--KRLGIAND-------------RGLSNVLATGLS-----------TQ
641537293_MAE_32940                     VD-ADLRRPQIH--LRLNLDND-------------HGLSNVLAEGLDW-----------N
643482531_PCC7424_4310                  IE-SDLRHPQYY--QWIDIPYQ-------------QGLSEVLSLNLD-----------VA
648185689_Cyan7822_0712                 VE-ANLRKPQYY--QWVDVPQP-------------EGLNEVLTLGLE-----------VA
2509502225_Pro9006DRAFT_3724            VD-ADMRRPQVH--SRLGLRN-------------LQGLSNILARNIDP-----------D
643584098_Cyan7425_1087                 VD-ADLRRPTVH--TRLELPN-------------LQGLSNAITANL-----------PLT
2503742029_Nos7107_3348                 ID-ADLRRPTIH--TRVHSLTN------------VWGLSNLISGSF-----------PIE
2504097563_Cal6303_4545                 VD-ANFRHPQLH--ESVGLVNT-------------KGLSEILGDGL-----------DLN
2507478127_Cal7103DRAFT_00050290        VD-ADLRHPQVH--TQLGIPNT-------------EGLSEVLSQGL-----------DIK
2510089327_Riv7116_4181                 VD-ANLQNPQVH--HSLGLINT-------------QGLSEILSQGL-----------DLN
2517239184_Mas10914DRAFT_0427           VD-ANLRHPQVH--HILGLVNA-------------KGLSEVLCEGL-----------DFS
2507335438_Tol9009DRAFT_00054260        VD-GDLRHPQIH--LRFGLLNN-------------QGLSEVLSEGI-----------DIS
2504086624_Osc7112_0134                 VD-ADLRRPQVH--EMLGLPN-------------WQGLHNVISEDVD-----------VQ
2506346341_MicvaDRAFT_2066              VD-ADLRRPQVH--EMLGLPN-------------WQGLHNVIAEDVD-----------VE
648859632_OSCI_3640004                  VD-ADLRRPQVH--EMLCLPN-------------WKGLSSVITEDLE-----------VE
2508872900_Oscil6407DRAFT_00010170      VD-ADLRRPQVH--EMLCLPN-------------WKGLSSVITEDLE-----------VE
643171775_AmaxDRAFT_4132                VD-ADLRNPRIH--SMLGVSN-------------LQGLSDAIAGNLE-----------IE
648386075_APCC8_010100004460            VD-ADLRNPRIH--SMLGVSN-------------LQGLSDAIAGNLE-----------IE
650384684_NIES39_C03520                 VD-ADLRNPRIH--SMLGVSN-------------LQGLSDAIAGNME-----------IE
646131598_AplaP_010100019750            VD-ADLRNPRIH--SMLGVSN-------------LQGLSDAIAGNME-----------IE
640018815_L8106_14065                   VD-ADLRQPKVH--RMLQLSN-------------EKGLSDVLIQNLE-----------IQ
638107046_Tery_1925                     ID-ADVRCPQIH--NMMGFSNQ-------------EGLSSVITDGL-----------LIE
2506483316_LYNGBM3L_63940               VD-TDLRRPRVH--KIFGLNNQ------------L-GLSDVLSKGIK-----------AN
648856139_OSCI_1010012                  VD-ADLRLPQLH--LRLGLPNV-------------RGLSDAIATDLS-----------LN
2508875360_Oscil6407DRAFT_00034810      VD-ADLRLPQLH--LRLGLPNV-------------RGLSDAIATDLS-----------LN
2509510258_Osc10802DRAFT_3909           VD-ADLRRPQLH--TRLGIPNV-------------RGLSDAIISDIG-----------LN
2509438066_Mic7113_6231                 ID-ADLRRPQLH--LRLGLPNE-------------QGLSDVITTDLS-----------LN
2504090796_Osc7112_4249                 VD-ADLRCPQLH--AKLGLPNV-------------RGLGEAISTDLS-----------LN
2506345448_MicvaDRAFT_4285              VD-ADLRCPQLH--AKLGLPNV-------------RGLGDAISTDLS-----------LN
2504091687_Osc7112_5131                 VD-ANMRLPQLH--TYLGLSNM-------------KGLSNVLSSDLS-----------LN
647568459_MC7420_6195                   ID-TNLRSPQLH--IRLELSNE-------------RGLSEIIAADST-----------IQ
647572642_MC7420_919                    VD-TDLRVPQLH--KRLDLPNS-------------QGLSDIITTNVA-----------IN
640014779_L8106_06284                   VD-ADLRNPQIH--QKLGLPN-------------LRGFRDAITSDLS-----------LN
2509421881_Oscil6304_2674               VD-ADLRSPKIH--TKLDVPN-------------LRGLSDAISTDIS-----------LN
641253939_AM1_4861                      VD-ADLRAPSLSR-VL----NLPNSP----------GLSDLMATE-----------GNLQ
2514735482_ACCM5_010100007602           VD-ADLRAPSLSR-VL----NLPNSP----------GLSDLMATE-----------GNLQ
2510441931_Cha6605_5932                 VN-ADLRKPSLQN-LVDRHHNRETLE----------GLTDVIAGT-----------TKLM
2503611061_Chro_0810                    VD-ANLRTPTIH--KMLNLSNA-------------QGLSNAIAEGLNFQYAIQQVRNSSE
2503613235_Chro_2969                    VD-ADLRNPNIH--KRLGLSNT-------------VGLSNVIVNDINFQDVLHRV-NCEP
2512979625_Fis9431DRAFT_3954            VD-ANLRSPNLH--QRVGLMNI-------------QGLTDVISSD-----------LDWS
2517059612_PCC9339DRAFT_00465           VD-ANLRSPNLH--QRVGLMNI-------------QGLTDVISSD-----------LDWS
2505768848_FJSC11DRAFT_1825             VD-ANLRSPSLH--QRVGLMNI-------------QGLTDVISSD-----------LDWS
2516149265_FIS9605DRAFT_06272           VD-ANLRSPSLH--QRVGLMNI-------------QGLTDVISAD-----------LDWS
2517241827_Mas10914DRAFT_3070           VD-ANLRFPTVH--TRVGLMNI-------------QGLTDVISQD-----------LDWN
2507335136_Tol9009DRAFT_00051240        VD-ANLRCPTLH--NRVGLMNI-------------QGLTDVISQD-----------LEWS
2504095135_Cal6303_2139                 VD-ANLRCPTLH--QRLGLMNI-------------QGLTDVISSD-----------LEWS
2507476798_Cal7103DRAFT_00036990        VD-ANLRCPTLH--KRVGVMNI-------------QGLTDIISSD-----------LDWS
2510085606_Riv7116_0460                 VD-ANLRCPTIH--KRVGLMNI-------------QGLTDIISSE-----------LEWS
2503739631_Nos7107_1000                 VD-SNLRCPTLH--QRLGILNV-------------QGLTDVIAQD-----------LDWE
2505801704_Cal7507_3428                 VD-ANLRAPSLH--KGLGAMNM-------------QGLTDVILQD-----------LDWN
```

```
2505804241_Cal7507_5918                     VD-ADLRFPTLH--KLVGSMNI-------------QGLTDVISHN-----------LDWE
643168344_AmaxDRAFT_0719                    VD-SDLRCPSLH--QQLGLINM-------------QGLTDLISSDA--------LDIDIE
648390241_APCC8_010100025948                VD-SDLRCPSLH--QQLGLINM-------------QGLTDLISSDA--------LDIDIE
646129996_AplaP_010100011596                VD-SDLRCPTLH--QHLGLINM-------------MGLTDLISSDL--------IDIDIE
650383923_NIES39_A06230                     VD-SDLRCPTLH--QHLGLINM-------------MGLTDLISSDL--------IDIDIE
640017922_L8106_10512                       VD-TDLRHPSLH--YFLGLANM-------------QGLTDVISTD-----------IDFN
2510101197_Gei7105DRAFT_2452                VD-TDLRHPSLH--IRLGLANT-------------RGLTDAIADS----------HLDVW
2509573281_Ple7327_1398                     VD-VDLRYPSLH--DRLMLSNQ-------------QGVSDILSSET----------LDFN
2506597130_Spi9445_0041                     VD-TDLRRPRVH--THLGLINA-------------YGLTNVLAGE-----------LEVN
2503636620_PCC7418_1894                     VD-ADLRRPQFE--HSSEDNTA-------------LGLTDLI--DR--------QELNLT
2506607995_Spi6313_0371                     VD-TDLRRPQIH--QRLGLVNG-------------DGLTDVLSGDL--------LAED--
643585148_Cyan7425_2153                     VD-ADLRKPDIH--KRLNLSNE-------------QGLSDLMASNIH-----------YR
2504681848_Pse7367_3384                     VD-TDMRQAYLYT-DGNGFVNSNMDSGMAITPSKQKGLTDIISAN-----------LKVA
647577962_S7335_3828                        VD-ADLRHPTLH--RLCNLPNE-------------KGLSDYIAGD--------------
2509846600_Lepto7375DRAFT_6617              VD-TNLRNPKIH--SYLKLPHDA-------------GLTDLASIDPSSITKNTFLHEPI-
2509774530_Lepto7104DRAFT_1750              ID-ADLRFPHLH--DFLDLPND-------------QGFSNILAGEL-----------DLK
2509804417_LepboDRAFT_3759                  VD-ADLYRSKVHV--YARVPNE-------------QGLSQYLTSDA-----------PLD
2531850144_CWATWH0003_3517                  VE-GDLRRPQVQKHKRLKVSSE-------------YSLEDILDNQISP-----------E
2509777916_Lepto7104DRAFT_5137              ID-GDLRKPTLHL--IFGQPNR-------------QGLSDLITQ----------ATTDPN
648856194_OSCI_1040007                      VD-ANLHSPTLH--TLLDLPN-------------FRGLSDLL-CN----------KLEPN
2508875344_Oscil6407DRAFT_00034650          VD-ANLHSPTLH--TLLDLPN-------------FRGLSDLL-CN----------KLEPN
2504087727_Osc7112_1201                     VD-ANLHSPSLH--LLLELPN-------------VKGLSNLL-DN----------KNEPD
2506348042_MicvaDRAFT_3627                  VD-ANLHSPTLH--LLLELPN-------------VKGLSNLL-DN----------KNEPH
2509438406_Mic7113_6573                     VD-ANLRQPQIH--ERLGIPN-------------QKGLCDLL-AK----------KLAPN
647571294_MC7420_5066                       VD-ANLIHPQIH--QQLNLPN-------------VKGLSDLL-AD----------NLMPH
2506481927_LYNGBM3L_44990                   VD-GNLHSPQLH--TNLNLQN-------------LPGLSDLL-SN----------DLEV-
640019302_L8106_03117                       VD-ANLRHPQLH--QSLGLPN-------------QKGLSNLLTDS----------RIAPS
2509707887_Pleur7313DRAFT_01875             VD-ATFSHPQLQ--VKSYSAN-------------KVGLSELLT-N----------QTDLN
641251898_AM1_2808                          ------------------------------------------------------------
2514737774_ACCM5_010100019113               VD-ANMAFPQLH--TRLGVPN-------------AEGLSDVI-DR----------NLDPN
641251897_AM1_2807                          VD-ANMAFPQLH--TRLGVPN-------------AEGLSDVI-DR----------NLDPN
2512634931_Chl7702DRAFT_3806                VD-ANFRTPCLH--TILGLEN-------------VQGLSNLISQE----------FSPQA
2516146228_FIS9605DRAFT_03233               VD-ANFRLPYLH--TKLGLEN-------------VHGLSNMISED----------LAPRP
641675948_cce_1468                          VD-TNFSNPQLH--NLLNVSN-------------HKGLIDVL-GG----------QVSPQ
2507501641_Cy51472_2500                     VD-TNFSNPQLH--NLLNVSN-------------HKGLIDVL-GG----------QVSPQ
640626055_CY0110_29394                      VD-TNFSHPQLH--NLLNVDN-------------HKGLIDVL-EG----------QISPQ
2531850936_CWATWH0003_4939                  VD-ANFFNPQLH--NLLNISN-------------NRGLIDIL-GG----------DVSPQ
643476430_PCC8801_3415                      VD-VNWHKPQLH--TLLEVSN-------------DAGLCQVI-NE----------DISPK
644980660_Cyan8802_2701                     VD-VNWHKPQLH--SLLEVSN-------------DAGLCQVI-NE----------DISPK
643478515_PCC7424_0240                      VD-TNLASPQLH--SWFNLPN-------------YKGLSDLLMDE----------ELASY
648186851_Cyan7822_1903                     VD-SNLANPQLH--LWLNLPN-------------YKGLSDLLVDD----------ELASY
2509575480_Ple7327_3597                     VD-ANFINPQLH--EWLNLPN-------------YRGLSHLLA-E----------SIPSE
2508645044_GLO73106DRAFT_00012970           VD-ANLNDPAIH--KYFSLSNE-------------KGLKDLLTYSLNEETIIQKTEL---
2509773763_Lepto7104DRAFT_0983              VD-SQFRRGGTRL-SSL------------LGIKADPGLSDYISNRAS-----------LN
2503745907_Cyan10605_1646                   ID-TDFRLPQLH--ILVNVDNN-------------QGLTHILTGKI-----------PWQ
2509874395_Syn6308DRAFT_1102                ID-ADLRLPQLH--RLLGIENN-------------QGLSEVLSSEL-----------KLE
644979531_Cyan8802_1534                     ------------------IKNE--------------------------------------
2503745344_Cyan10605_1086                   VD-ADMRKPKIH--TYLGIKNKI-------------GLSNLIAEDI-----------LPE
2509873750_Syn6308DRAFT_0457                VD-MNLREPKLH--SYLGLENKA-------------GISDIIFNDI-----------PLN
2505785711_Chr6712_1954                     VE-ADLRSPNKLS-SNNSIFRKSNR-----------GLSDLLMAK--------N--LAVE
2509706287_Pleur7313DRAFT_00275             VD-TDLRSTDCLT-KSFGL--ESEI-----------GLSDILNQD--------NTNLGLE
640027738_N9414_07903                       ------------------------------------------------------------
2506482929_LYNGBM3L_58920                   ID-ADLHKPVQH--RIWDLSNEV-------------GLSNMLVNQ---------------
637461200_glr3785                           VD-ADLRRPTVS--NKLELSNGP-------------GLSTVLAGE---------------
2503887239_Lepto7376_1629                   ID-ANFRKPEI-P-EFLNLDNDA-------------GLVDLLREPTQ----------DIT
638113739_sync_0150                         VD-ADLRKPQLH--IRLGINN-------------LQGLSNLITED----------ALHWR
638963744_RS9917_02426                      ID-ADLRKPQMH--TRLGLNN-------------LSGLSNLLADD----------GFHWR
2507493170_Syn8016_1895                     ID-ADLRKPQLH--TRLGLNN-------------LRGLSNVLTDD----------SSHWR
638960084_WH5701_13945                      VD-ADLRQPQVH--HRLGLNN-------------LTGLSNLLTEE----------EQDWR
650128418_SCB01_010100004274                VD-GDLRQMQLH--QRFAADG-------------ARGWSELFTEH----------PPELE
650132168_SCB02_010100007823                VD-GDFRQAQLH--QRLGVDG-------------ARGWSEMFTES----------PPALS
2508551611_Cyagr_1223                       ID-SDMRLPMQA--RYLGVEQ-------------GDGLSTLLSDS----------TRKPT
640081072_P9515_13861                       ID-ADLRRPQIH--SRLGLNN-------------LTGLSNILTNP----------SLTLE
648050855_Aazo_3125                         ID-ANLRDPSLH--KQLNLPNE-------------QGLSTLLASD---------------
2504129904_Anacy_0284                       ID-ANLRDPSLH--KQLNLPNE-------------QGLSTLLGSD---------------
2506490031_Ana7108_0216                     ID-ANLRAPSLH--QQLNLPNE-------------QGLSTLLASD---------------
2509770736_CylstDRAFT_4696                  ID-ANLRVPSLH--TQLNLPNE-------------QGLSTLLASD---------------
642600344_Npun_R1070                        ID-ANLRDPSLH--HQLNLPNE-------------QGLSTLLASD---------------
2505799741_Cal7507_1481                     ID-ANLREPSLH--KQLNLPNE-------------QGLSTLLASD---------------
2509779661_Mic7126DRAFT_0411                ID-ANLREPSLH--KQLNLPNE-------------QGLSTLLASD---------------
2503739922_Nos7107_1290                     ID-ANLRDPSLH--EQLNLPNE-------------QGLSTLLSSE---------------
640027761_N9414_00965                       ID-ANLREPSLH--EQLNLPNE-------------QGLSTLLASD---------------
637233217_alr2833                           ID-ANLRDPSLH--EHLNLPND-------------QGLSTLLASE---------------
646566603_Ava_1116                          ID-ANLRDPSLH--EHLNLPND-------------QGLSTLLASE---------------
2509808002_Nos7524_0433                     ID-ANLRNPSLH--QQLNIPND-------------QGLSTLLASE---------------
2517241466_Mas10914DRAFT_2709               ID-ANLRDPSLH--KQLNLPNE-------------QGLSTLLASD---------------
647105946_CRC_01573                         ID-ANLRDPNLH--KQLNLPNE-------------QGLSTLLAND---------------
647109494_CRD_02102                         ID-ANLRDPNLH--KQLNLPNE-------------QGLSTLLAND---------------
2507332201_Tol9009DRAFT_00021840            ID-ANLRDPSLH--KQLNLPNE-------------QGLSSLLASD---------------
2512975951_Fis9431DRAFT_0279                ID-ANLRSPNLH--QQLNLPNE-------------QGLSTLLVSE---------------
2517062640_PCC9339DRAFT_03498               ID-ANLRSPNLH--KQLNLPNE-------------QGLSTLLVSE---------------
2505767181_FJSC11DRAFT_4188                 ID-ANLRAPNLH--KQLNLPNE-------------QGLSTLLVSE---------------
2516146836_FIS9605DRAFT_03842               ID-ANLRAPSLH--KQLNLPNE-------------QGLSTLLVSE---------------
2512634275_Chl7702DRAFT_3149                ID-ANLRDPSLH--KQLNLPNE-------------QGLSTLLVSE---------------
2510089385_Riv7116_4239                     ID-ANLRDPSVH--KHLNLPND-------------QGLSTLLTSD---------------
2504094820_Cal6303_1825                     ID-ANLREPNLH--KQLNLPNE-------------QGLSSLLVSD---------------
2507482515_Cal7103DRAFT_00094220            ID-ANLREPTLH--KQLNLPNE-------------QGLSSLLASD---------------
2503612562_Chro_2300                        ID-TDLRRPSLH--QILNLPND-------------YGLSTLLSSD---------------
```

```
2517695945_SYN7509DRAFT_0062          ID-ADLRHPNLH--QMLNLPNE-------------QGLSTLLESD---------------
2503608186_GEI7407_2184               ID-ADLRRPSLH--KQLNLPND-------------RGLSSLLSGD---------------
2504685208_Cri9333_2761               ID-TDFRQPMLH--QLLYLSNE-------------YGLSSLLESN---------------
2509431928_Mic7113_0093               ID-ADLRNPTLH--EKLNLPND-------------YGLSTLLSSD---------------
2506479159_LYNGBM3L_28310             ID-VDMRRPRLH--QQLGLSHQ-------------EGLSTLLEDD---------------
640017638_L8106_27951                 ID-ANLRSPALH--EQLNLSNE-------------QGLSTFLSNE---------------
2509806250_LepboDRAFT_5593            ID-ADLRRPGLH--KQLQLPND-------------HGLSTLLTSD---------------
2504583836_Pse7429DRAFT_1713          ID-ADMRSPALH--KQLNLPNE-------------RGLSTLLASK---------------
641251010_AM1_1910                    ID-ADLRNPSLH--YMFNLNNH-------------QGLSTLLNGN---------------
2514735759_ACCM5_010100008979         ID-ADLRNPSLH--YMFNLNNH-------------QGLSTLLNGN---------------
2509847582_Lepto7375DRAFT_7599        ID-ANFRNPSLH--QYLGLLNV-------------QGLSTLLHPN---------------
648051793_Aazo_4516                   ID-ANLRAPSLH--KILAISND-------------WGLSLLLLDD---------------
2504134691_Anacy_5006                 ID-ANLRSPSLH--KILNISND-------------WGLSLLLLDD---------------
2506494605_Ana7108_4733               ID-ANLRSPSLH--KILKISND-------------WGLSLLLVDD---------------
647107726_CRC_03451                   ID-GNLRSPQLH--KVLSIHND-------------WGLSLLLLDD---------------
642604187_Npun_R5250                  ID-ANLHNPSLH--KILGLSND-------------WGLSLLLVDE---------------
2509767727_CylstDRAFT_1687            ID-ANLRYPRLH--KILELSND-------------WGLSLLLVDG---------------
2507335141_Tol9009DRAFT_00051290      ID-ANMRDPSLH--NILELSND-------------WGLSLLLLDE---------------
640028130_N9414_07219                 ID-ANLRCPNLH--KNLQLSND-------------WGLSLLLVDE---------------
637230414_all0059                     ID-ANLRSPKLH--KILQLSND-------------WGLSLLLLDE---------------
646568157_Ava_2661                    ID-ANLRSPKLH--KILQLSND-------------WGLSLLLLDE---------------
2509811005_Nos7524_3436               ID-ANLRSPKLH--KVLQLSNE-------------WGLSLLLVDE---------------
2503739635_Nos7107_1004               ID-ANFQYPNLH--KILQLSND-------------WGLSLLLLDE---------------
2509781657_Mic7126DRAFT_2408          ID-ANLQRPSLH--NILEISND-------------WGLSLLLVDE---------------
2517243237_Mas10914DRAFT_4480         ID-ASLRQPNLH--KILGLTND-------------WGLSLLLVEE---------------
2512634393_Chl7702DRAFT_3267          ID-ANLHQPKLH--KVLALSND-------------WGLSLLLVDE---------------
2505769181_FJSC11DRAFT_2156           ID-ANLREPSLH--KTLELAND-------------WGLSLLLVDE---------------
2512977368_Fis9431DRAFT_1696          ID-ANLREPSLH--KTLEISND-------------WGLSLLLLDE---------------
2517063601_PCC9339DRAFT_04460         ID-ANLREPSLH--KTLELSND-------------WGLSLLLLDE---------------
2516147062_FIS9605DRAFT_04068         ID-ANLRQPSLH--KVLELSND-------------WGLSLLLVDE---------------
2507482923_Cal7103DRAFT_00098300      ID-ANLREPSLH--KYLDLQNE-------------WGLSLLLVDE---------------
2510085595_Riv7116_0449               ID-ANLRSPCLH--SILELSND-------------WGLSLLLLEE---------------
2503614917_Chro_4637                  ID-ADLRRSNIH--KLLNLSND-------------RGLSTLLTEE---------------
2504093446_Cal6303_0467               ID-ANWHNPTLH--KTLDLSNE-------------WGLSLLLVDE---------------
647578236_S7335_5395                  VD-ANLRSSTLQ--ERFNFPEG-------------RGLNALLDQP---------------
2503798844_Sta7437_0777               ID-ADLRCPSLH--EKLGVENN-------------SGLSDLLQGT---------------
2509708827_Pleur7313DRAFT_02815       ID-ADLRRPSLH--KAFNLANH-------------TGLADFLAGE---------------
637009749_sll0923                     VD-GDRYFPQKE--SWLKLAEITGFGGENTTPDGNGVLGSLVANSNGHNGDLP-------
2503800689_Sta7437_2597               VD-ADMRKPKIH--KLAKLAND-------------EGLSEAIANERP--WTDYVRLGVLE
2509710958_Pleur7313DRAFT_04946       VD-ADLRKPKLH--KLARIPNE-------------SGLMQAITKDQP--WTNYIQSGSIK
2505786361_Chr6712_2601               VD-ADLRKPRIH--KLTKQSNE-------------KGLSEAIVSDEP--WSENCLPSIVE
2508650693_Xen7305DRAFT_00027920      VD-ADLRKPKIH--KMAKISNE-------------QGLSE-VISDNKHSWTE-VVQNVTG
2506480412_LYNGBM3L_19610             VD-GDLRQPTLH--LLAGLPNH-------------GGLSTALTSDRP--WYSLVQTAETE
2509512444_Osc10802DRAFT_6095         VD-ADMRKPTIH--QFLKRPNM-------------FGLSSAIATDTP--WRQLVYSADGG
643587900_Cyan7425_4938               VD-ADTRNPTIH--QIIEQPNL-------------RGLTTAIASDKS--WQEMLIPGGVT
2504582150_Pse7429DRAFT_3127          VD-ADLRKPTVH--KLARLSNK-------------FGLSTALATPSP--WQELTQVVDEK
2504679882_Pse7367_1451               VD-ADMRKPNVH--HLAKLRNN-------------YGLSTVIATNRS--WRQVVQSANVK
2507088905_Pse6802_3551               VD-ADLRRPTIH--KLAGLSNG-------------LGLSTAIATNHA--WREIVRTLSLQ
2508687870_Syn7502_01070              VD-ADMRKPTIH--SLAGVSNK-------------LGLSQAIATKLP--WQQIIHTGDHS
2506748948_Syn7336_3628               VD-ADFMHSTVH--QNFEIGNR-------------VGLST-VFRHPELDWRDMVSSFQQG
643484053_PCC7424_5769                VD-ANLNQPAIH--QVFQLPNT-------------SGLSTAIATDIP--WLELIQSHSPK
648199484_Cyan7822_5718               VD-GNFSNPMLH--EVFSLPNT-------------QGLTTTIATELS--WSKIVQSFEEE
2510436294_Cha6605_0295               ID-ADM-----HKSSIAQLCANSPLFKREDCDIED-GLSDAIVGSSNW------------
637314397_tll1767                     ID-GDLRQPKIH--RYLGLTNE-------------QGLADWLIHRRHWHNIAQT------
637313820_tll1199                     VD-ANLHQPSLH--QVLGIPN-------------VNGLGELLQGTMPLPHWPSLLANL--
2512980079_Fis9431DRAFT_4408          VD-ADMRHPIQH--HIWQLSNKR-------------GLSNIIVD----------------
2517061743_PCC9339DRAFT_02599         VD-ADMRHPIQH--HIWQLSNKK-------------GLSNLIVD----------------
2505770929_FJSC11DRAFT_1293           VD-ADMRHPIQH--HIWQLSNNQ-------------GLSNIIVD----------------
2516148164_FIS9605DRAFT_05171         ID-ADMRHPVQH--HIWGLTNAQ-------------GLSNVIVD----------------
2512634416_Chl7702DRAFT_3290          VD-ADMRRPCQH--HIWELTNAQ-------------GLSNAIVDD---------------
2505800360_Cal7507_2092               VD-ADMRHPVQH--HIWGMTNAV-------------GLSNVIVD----------------
2509780096_Mic7126DRAFT_0847          VD-ADMRHPVQH--QIWGMTNVV-------------GLSDAIID----------------
2505800523_Cal7507_2254               VD-ADMRHPVQH--HIWGMINAV-------------GLSNVMVD----------------
2507478259_Cal7103DRAFT_00051610      VD-ADMRHPIQH--HIWYLNNNI-------------GLSNLLVE----------------
637234831_all4432                     VD-ADMRHPIQH--HIWDLSNAI-------------GLTNVLIG----------------
646566875_Ava_1386                    VD-ADMRHPIQH--HIWDLSNAI-------------GLTNVLIG----------------
642600743_Npun_R1496                  VD-ADMRNPIQH--HIWGLNNTV-------------GLSNVIVG----------------
2507334816_Tol9009DRAFT_00048030      VD-ADMRHPIQH--HIWELTNAV-------------GLSNIIVD----------------
2507476704_Cal7103DRAFT_00036050      VD-ADMRQPIQH--HIWNLTNAI-------------GLTNVIVD----------------
2510090628_Riv7116_5482               VD-ADMRHPIQH--HTWGLTNAV-------------GLSNVIVD----------------
2506483552_LYNGBM3L_64290             ID-ADLRYPSQH--HLWELTNSV-------------GLSNILVG----------------
2517697529_SYN7509DRAFT_1646          ID-ADTRQPSQH--KIWNLVNQV-------------GLSNVLVN----------------
2503608035_GEI7407_2035               VD-ADMRHPMQH--HVWHLTNLA-------------GLSNVIMK----------------
2509433692_Mic7113_1857               ID-ADLHHPMQH--HIWHLANAV-------------GLSDVIVN----------------
640015133_L8106_09871                 ID-ADLHHPQQH--HIWNLTNEV-------------GLSDVIVN----------------
2503795055_Glo7428_2485               VD-ADMRHPLQH--HIWELTNAA-------------GLSDVIVS----------------
2509506717_Osc10802DRAFT_0363         VD-ADMRHPLQH--HIWELINAV-------------GLSNAIVG----------------
2503614965_Chro_4685                  VD-ADMRRPRQH--HIWEKANTE-------------GLSDAIVG----------------
2510091904_Riv7116_6758               VD-ADMQSPLQH--NLWGVNNVK-------------GLSDIIVG----------------
2503612629_Chro_2367                  ID-ADLRSPIQH--HIWQQTNVE-------------GLSHVLVG----------------
2503612419_Chro_2158                  VD-ANMHHPIQH--QIWRLSNTS-------------GLSNILSG----------------
2506493946_Ana7108_4087               VD-ANMRHPLQH--QIWNITNTV-------------GLSDVIVD----------------
2509803997_LepboDRAFT_3339            ID-ADLHHPSQH--HIWNLTNVG-------------GLSHVIVE----------------
2512979777_Fis9431DRAFT_4106          VD-ADMRKPTQH--HLWSLINSV-------------GLSHLLVG----------------
2517062202_PCC9339DRAFT_03059         VD-ADMRKPTQH--HLWSLINSV-------------GLSHLLVG----------------
2505770300_FJSC11DRAFT_0671           VD-ADMRKPTQH--HLWGLINSV-------------GLSHLLVD----------------
2507334440_Tol9009DRAFT_00044270      VD-ADMHSPSQH--HLWGLINSV-------------GLSNIIVG----------------
2510088639_Riv7116_3493               VD-ADMRQPMQH--HLWGLINSG-------------GLSNVIVG----------------
```

```
2505803639_Cal7507_5320                 VD-ADMRQPSQH--HLWGLINSV-------------GFSNVIAG----------------
2509782388_Mic7126DRAFT_3139            VD-ADMRQPSQH--HLWGLINSI-------------GFSNLIAG----------------
2507335607_Tol9009DRAFT_00055950        VD-ADMRQPSQH--HLWGLINSV-------------GLSNVMVG----------------
2510088989_Riv7116_3843                 VD-ADMYNPSQH--HLWGLINSI-------------GLSNVMVG----------------
2504096650_Cal6303_3648                 VD-ADMRQPSQH--HLWGLMNVK-------------GLSNLLVD----------------
2507481772_Cal7103DRAFT_00086780        VD-GDMRNPSQH--HLWGVMNLT-------------GLSNVIVG----------------
2509803885_LepboDRAFT_3227              VD-ADMRNPSQH--HLWGIVNSV-------------GLSNVVVD----------------
647579433_S7335_2031                    ID-ADMRHPSLH--KTWNISNRV-------------GLSSFLVG----------------
2509846113_Lepto7375DRAFT_6130          VD-ADLRKPTQH--HIWDLPNVQ-------------GLSNFASG----------------
2512978854_Fis9431DRAFT_3183            VD-GDLEHPIQH--QIWKLTNNE-------------GLSDVICG----------------
2516143706_FIS9605DRAFT_00708           VD-GDLHRPVQH--KIWELNNTQ-------------GLSNVIVG----------------
642600641_Npun_F1381                    ID-GNLHRPVQH--QIWELNNNQ-------------GLSNLILE----------------
2509770219_CylstDRAFT_4179              ID-GDLHRPVQH--KIWDLTNSQ-------------GLTNLIVG----------------
2504094091_Cal6303_1107                 VD-GDLHRPSQH--QIWDLPNSE-------------GLSNVIVG----------------
2507479320_Cal7103DRAFT_00062230        VD-GDLHRPVQH--NIWDLPNSE-------------GLSNVIVG----------------
2510085390_Riv7116_0244                 ID-GDLHRPSQH--HIWELPNAQ-------------GLSNVIVG----------------
642601572_Npun_F2453                    ---------------CWD------------------------------------------
2503611448_Chro_1193                    VD-ADMYRPLQH--EIWELPNHL-------------GLSNIIVG----------------
2503796978_Glo7428_4384                 VD-ADMLRPTQH--QIWELPNQS-------------GLSNLLVG----------------
2504087786_Osc7112_1259                 VD-ADMHCPVQH--RIWELPNQV-------------GLSNVIVG----------------
643584136_Cyan7425_1127                 ID-ADMRVPTQH--HFWQLTNAI-------------GLSEVIVG----------------
647105865_CRC_01492                     VD-GDLRRPRQH--KVWGLPNFV-------------GLSNVLVA----------------
647108284_CRD_00419                     VD-GDLRRPCQH--RVWKLPNFV-------------GLSEILAG----------------
2505800344_Cal7507_2076                 ID-ADMRRPRQH--KIWEQSNLI-------------GLSNILVG----------------
2509781889_Mic7126DRAFT_2640            ID-ADMRRPRQH--KIWEQSNLI-------------GLSNVLVN----------------
2507481279_Cal7103DRAFT_00081850        ID-ADMRRPRQH--KVWEKANFM-------------GLSEVLVG----------------
637233240_alr2856                       ID-ADMRRPRQH--EMWQQPNLM-------------GLSNVLVG----------------
646566532_Ava_1045                      ID-ADMRRPRQH--EMWQQPNLM-------------GLSNVLVG----------------
640028261_N9414_00005                   ID-ADMRRPRQR--EVWQVRNLM-------------GLSNVLAG----------------
642603803_Npun_R4851                    VD-ADMRHSCQQ--EIWKLSNPA-------------GLSNVLVG----------------
2509770699_CylstDRAFT_4659              VD-ADMRHSCQQ--EIWKLSDPV-------------GLSNVLVG----------------
2507478691_Cal7103DRAFT_00055940        ID-ADMRCSRQH--EVWKVENSQ-------------GLNEILEN----------------
641249527_AM1_0407                      ID-ADMRRPSQH--QVWELPNAL-------------GLSNVLVE----------------
2514736728_ACCM5_010100013856           ID-ADMRRPSQH--QVWELPNTL-------------GLSNVLVE----------------
643586770_Cyan7425_3796                 ID-ADLRLPLQH--QIWELPNAT-------------GLTHLLADN---------------
2509555626_Dacsa_3240                   ID-GDLRRPSQH--QVWEEPNAV-------------GLSNVLVED---------------
2509775597_Lepto7104DRAFT_2817          ID-ADLRSPSQH--HIWDVVNRA-------------GLSQVLIRE---------------
2506492920_Ana7108_3067                 VD-ADLRNPSQH--KIWEIPNEV-------------GLSNVLKR----------------
2512631781_Chl7702DRAFT_0655            VD-ADLRNPSQD--KIWDIPNRI-------------GLSNVIKR----------------
2504685956_Cri9333_3503                 ID-ADMRKPSQH--KIWQLPNET-------------GLSTVLTG----------------
2510436704_Cha6605_0705                 VD-ADMRKPAQH--LIWRQGNYE-------------GLSNVLSGQ---------------
2507482822_Cal7103DRAFT_00097290        ID-ADMRHPSQH--DIWGQTNEL-------------GLSNVIVG----------------
2510087184_Riv7116_2038                 ID-ADVRNPFQH--HAWKLTNQL-------------GLSNAIVG----------------
2506745596_Syn7336_0315                 VD-ADLRNPSQA--ALWELSAGP-------------GLVEAISGK---------------
2503796286_Glo7428_3705                 ID-ADLRHPSQH--QIWEIANHI-------------GLMNTIAKR---------------
642604427_Npun_F5505                    ID-ADMRVPSQH--HLWEVSNAD-------------GLSEVLVG----------------
2503612394_Chro_2133                    VD-ADMRDSSQH--HLWQLTNAA-------------GLSEVLVG----------------
2512632720_Chl7702DRAFT_1594            ID-ADMRVPSQH--EFWKLENYP-------------GLSEVLVG----------------
2509807906_Nos7524_0337                 ID-ADMRVPSQH--KIWELSHTS-------------GLSDVLVG----------------
2509770373_CylstDRAFT_4333              ID-ADMRVPFQH--TLWQLTNTA-------------GLSDFLVG----------------
646570355_Ava_4846                      ID-GDMRSPSQH--QFWQLNNLV-------------GLSEVLVG----------------
2510438837_Cha6605_2838                 ID-ADMRVPYQH--RFWKLPLRK-------------GLSELIER----------------
2506477923_LYNGBM3L_10200               ID-SDMRHPCQH--YIWQVMNVA-------------GLSQVLIG----------------
2509574529_Ple7327_2646                 VD-ANLHHPIQH--RIWELYPNV-------------GLSNLLVEQ---------------
643170532_AmaxDRAFT_2895                ID-GDMRQPIQH--HIWGLTNAV-------------GLSDVLVE----------------
648389308_APCC8_010100021105            ID-GDMRQPIQH--HIWGLTNAV-------------GLSDVLVE----------------
646129623_AplaP_010100009700            ID-GDMRQPIQH--HIWGLTNAV-------------GLSDVLVE----------------
650384826_NIES39_C04940                 ID-GDMRQPIQH--HIWGLTNAV-------------GLSDVLVE----------------
2503801641_Sta7437_3540                 ID-GDLRKPSQH--HLWNLDNFV-------------GLKDVISA----------------
2509711085_Pleur7313DRAFT_05073         ID-GDLRRSSQH--HLWGVDNRK-------------GLRDIITE----------------
2503801821_Sta7437_3719                 ID-GDLRRANQH--HLWNMSNHL-------------GLKDVLTH----------------
2505786500_Chr6712_2739                 ID-GDLRRASQH--QIWGTSNHI-------------GLKDVLTN----------------
2508650623_Xen7305DRAFT_00027220        ID-GDLRKASQH--QLWGVSNQK-------------GVKELLTE----------------
2509706990_Pleur7313DRAFT_00978         ID-GDLRKASQH--ELWQVSNRA-------------GVKDIVAH----------------
2505787175_Chr6712_3399                 ID-GDLRKPSQS--TLWKVADIP-------------GIAEVIRD----------------
2508650743_Xen7305DRAFT_00028420        VD-GDLRRPSQH--LLWNRNSHQ-------------GIKEVIQG----------------
2509711668_Pleur7313DRAFT_05656         ID-ADMRRPTQH--NIWEIPNQV-------------GLSNVL-NQ---------------
2509712372_Pleur7313DRAFT_06363         ID-GDLRRPTQH--NLWEVSNDE-------------GLTNVL-RE---------------
2505785156_Chr6712_1404                 ID-GDLRCPSQH--RRWQISNQV-------------GLSNVL-HQ---------------
2503799529_Sta7437_1457                 ID-ADMRRPTQH--KIWSIPNYL-------------GLSNVL-KG---------------
2506749054_Syn7336_3731                 ID-GDLFRPAQH--EIWQLDGGI-------------GLRQVLQGS---------------
2503799932_Sta7437_1855                 ID-ADVHHPQQQ--EIWELDNSV-------------GLLDVLQN----------------
2505786145_Chr6712_2386                 ID-ADLHRPQQH--KIWGTFNHQ-------------GLAEILRD----------------
641538163_MAE_41520                     ID-ADLPNPQQQ--RIWSLDNDQ-------------GLSDILA-R---------------
2507085801_Pse6802_0485                 ID-TDMRHPSQH--AIWDIPHGL-------------GLTDLLAGQ---------------
2507089189_Pse6802_3805                 ID-ADMRRPSQQ--LIWNIANTQ-------------GLSDLIAGK---------------
2508689585_Syn7502_02785                VD-VDMRRPSQH--KIWGLGNAS-------------GLSNILVGE---------------
2504679565_Pse7367_1141                 VD-ADMRHPAQH--RIWQLPNEN-------------GLSDLLTG----------------
2503802282_Sta7437_4176                 ID-AHLHHPIQH--RIWDLYNNC-------------GLSNLIAEQ---------------
2509709361_Pleur7313DRAFT_03349         VD-ANLHSPIQH--RIWDIYNNN-------------GLSNVIAEQ---------------
2508651492_Xen7305DRAFT_00035910        ID-ANLHSPIQH--RIWDIYAEP-------------GLSNFIAEQ---------------
2505784713_Chr6712_0967                 VD-ANLHSPVQQ--YIWNTYSDY-------------GLSNLLIE----------------
2514739634_ACCM5_010100028418           ID-ANLQSPKQH--HLFNLPNEL-------------GLSTAIKDT---------------
637461727_glr4310                       VD-GDLRKPRQH--ALWDLDNRQ-------------GLSTLLVGE---------------
641253915_AM1_4837                      VD-GNLRNPFQD--RMWDVLNEF-------------GLSNVLFGQ---------------
2514739407_ACCM5_010100027286           VD-GNLRNPFQD--RMWDVSNEF-------------GLSNVLFGQ---------------
2509712141_Pleur7313DRAFT_06132         ID-GDLYKSSQS--MLWNISNSV-------------GLSNVIEDE---------------
2506482932_LYNGBM3L_58950               ------------------------------------------------------------
```

```
2506608339_Spi6313_0712                     LE-ADLAAPQQH--EIWRLTNVW-------------GLGNVLQEQ---------------
641249511_AM1_0391                          ID-ADIRLASQH--QIWELGNQE-------------GLTHILLNK---------------
2514736711_ACCM5_010100013771               ID-ADVRLASQH--HIWELKNQE-------------GLTHILLRN---------------
2509500847_Pro9006DRAFT_2342                ID-ADLRSPSQH--LLWKTPNTQ-------------GLSTLLDQE---------------
2504681110_Pse7367_2660                     ID-ADLRKPTQH--QLWQVPNSQ-------------GLSNVLAGS---------------
2509874159_Syn6308DRAFT_0866                IE-ADLRQPGQKKIWK-NIDNGL-------------GLSNLLQKENNALISDSVVSV---
2509428796_Syn6312_0755                     ID-CDLRRPAQH--HCWQIPNQI-------------GLSNILVED---------------
2508653159_Xen7305DRAFT_00052600            ID-ANFHSPIQH--KVWNVYHNI-------------GISN-LIGE---------------
2512978631_Fis9431DRAFT_2960                ID-ADLRRPVLH--TLFNLVPQP-------------GITDVIDSE---------------
2517061062_PCC9339DRAFT_01917               ID-ADLRRPMLH--TLFNLAPQP-------------GITDVIDSQ---------------
2505768802_FJSC11DRAFT_1780                 ID-ADLRRPILH--TLFNLAPRP-------------GITDVIDSE---------------
2516146713_FIS9605DRAFT_03719               ID-ADLRHPMLH--TLFNLAPQP-------------GITDAIDKE---------------
2507336787_Tol9009DRAFT_00067760            ID-ADLRRPMQH--TLFNLAPKP-------------GITDVIEKR---------------
2517242598_Mas10914DRAFT_3841               ID-ADLRRPVQH--TLFNLDPRP-------------GITDVLEGN---------------
2507476104_Cal7103DRAFT_00030040            ID-ADLRRPVQH--TIFNLPAKP-------------GITDIVEGN---------------
2510090822_Riv7116_5676                     ID-ADLRRPVQH--TLFNLPPKP-------------GITEIIEGR---------------
2509766470_CylstDRAFT_0429                  VD-ADLYRPMQH--NLFNSAPYP-------------GITDVLNGD---------------
637233445_alr3059                           ID-ADLRKPSQH--TLFNLPPRP-------------GITDVIDGT---------------
646566337_Ava_0852                          ID-ADLRKPSQH--TLFNLPPRP-------------GITDVIDGT---------------
2509812587_Nos7524_5019                     ----NFEMPFQA--NF--------------------------------------------
2507480867_Cal7103DRAFT_00077730            ID-ADLRRPKLH--RVFNLRSSP-------------GLSDAITER---------------
2504094392_Cal6303_1407                     ID-ADLRKPVQH--KLFNLNPQP-------------GIAEVFDGT---------------
2506481239_LYNGBM3L_38240                   ID-ADWRHPMQH--NLFGLPPKP-------------KVTDVIDSN---------------
2503613794_Chro_3525                        ID-ADLRCPTQH--KLFKLAAKP-------------GLAEVAQGK---------------
2510437494_Cha6605_1495                     ID-ANLWQPEQH--RLFNLPDSP-------------GLAEVLEGK---------------
2509773567_Lepto7104DRAFT_0787              LD-ADLRQPSPT--RFFGAATRP-------------GFGDLVTQS---------------
641254791_AM1_5726                          ID-ADMRRPSIH--KIFQLPQDP-------------GLQDYLRGSVKEG-----------
2514737653_ACCM5_010100018486               ID-ADMRRPSIH--KIFQLPQDP-------------GLQDYLRDSVKED-----------
637459597_gll2202                           VD-ADLVRPSLS--ETFGLEGRGGLAEWLFHRPSAGSVGNYVQQ----------------
gi|4512007|gb|AAD21564.1|                   ID-ADLRKGYAHKM---------------FGHKNDKGLSEFLSGQ---------------
gi|53987110|gb|AAV27324.1|                  ID-ADMRRGYIYRY---------------FDGLPNNGLSEILTGR---------------
637230853_all0493                           IE-TDLR--SPSRCSSLRVSSDEDATLEPLRYY-------GSLSECIRLV----------
646568404_Ava_2908                          IE-TDLR--SPSRSTSLRVSPDEDATLEPLRYY-------GSLSECIRLV----------
2509812367_Nos7524_4799                     IE-TDLR--SPSRCLSLKVNPDPDATIEPLRYY-------GSLSECIRLV----------
642599777_Npun_F0459                        IE-TDLR--SPSRSTSLKVIPDPDATIEPLRYY-------ANLSECIRLV----------
2509772030_CylstDRAFT_5990                  IE-TDLR--SPSGASSLNVTPDPDATIEPLRYY-------SSLSECIRLV----------
2503740929_Nos7107_2283                     IE-TDLR--SPSRAASLKVNLDPDATIEPLRYY-------ASLSDCIRLV----------
640027950_N9414_23213                       IE-TDLR--SPSHASSLNVSPDTDVSVEPLRYY-------ARLNECIRLV----------
2505798270_Cal7507_0021                     IE-TDVR--SPSCCSSLKVTSDPDATIEPLRYY-------ARLSECIHLV----------
2509781264_Mic7126DRAFT_2015                IE-TDLR--SPSCCSSLKLAADPDATIEPLRYY-------ASLSECIRLV----------
2507337082_Tol9009DRAFT_00070720            IE-TDLR--SHSRCQSLNVNLDPDATLEPLRYY-------GSLSECIYLV----------
2517243673_Mas10914DRAFT_4916               VE-TDLR--SPSHSSSLRVTSDPDATVEPLRYY-------ASLSECIRIV----------
2504133191_Anacy_3523                       IE-TDLR--SPSQAASLKIAPDSYATVEPLLYY-------SSLSDCIRLV----------
2506492757_Ana7108_2905                     IE-TDLR--SPSRASALKVSPDSYATIEPLLYY-------SSLSDCIRLV----------
648049213_Aazo_0828                         IE-TDLR--SPSRASSLNVA--SYATLEPLLYY-------SSLSDCIRLV----------
2512978956_Fis9431DRAFT_3285                ME-TDLR--SPSRSPSLKVSIDPNANIEPLRYY-------GSLSECIRLV----------
2517059344_PCC9339DRAFT_00197               IE-TDLR--SPSGCPSLKVSIDPNAKIEPLRYY-------GSLSECIRLV----------
2505768976_FJSC11DRAFT_1951                 IE-TDLR--SPSRSPSLKVTIDPNANIEPLRYY-------GSLSECIRLV----------
2516145655_FIS9605DRAFT_02658               IE-TDLR--SPSCSPSLKVTIEPEANLEPLRYY-------GSLSECIRLV----------
2512633064_Chl7702DRAFT_1938                VE-TDLR--SPSCCPSLQVTLNPDVNLEPLRYY-------ASLNECIHLV----------
2510085904_Riv7116_0758                     IE-TDLR--SQSRCESLNVNLDPDANVEPLRYY-------GS-SECIRLV----------
2504093110_Cal6303_0131                     IE-TDLR--SPSRCKSLNVSINPDATIEPLRYY-------GSFSECIRLV----------
2507480421_Cal7103DRAFT_00073250            IE-ADLR--SPSYCESLGVTIDPNATTEPLRYY-------ASLGECIRLV----------
2503609163_GEI7407_3152                     IE-ADLR--SPSEAKALKVAPDPDSTIEPLRYY-------GQIGDCIRLV----------
2509421114_Oscil6304_1907                   IE-ADLR--SPSQAQSLQVAIEPDANLEPLRYY-------GQFHQCIRLA----------
2509805590_LepboDRAFT_4932                  IE-TDLR--SPSSAQGVNLTADPDSQIEPLRYF-------GQISDCIRLA----------
646129274_AplaP_010100007922                IE-ADLR--SPSQVERMRLAIDSQDRWEPLRYY-------GDLNNCIRMV----------
650387413_NIES39_K02750                     IE-ADLR--SPSQVERMRLAIDSQDRWEPLRYY-------GDLNNCIRMV----------
643168837_AmaxDRAFT_1210                    IE-ADLR--SPSQAERMRLAIDSQDRWEPLRYY-------GDLNNCIRMV----------
648390350_APCC8_010100026493                IE-ADLR--SPSQAERMRLAIDSQDRWEPLRYY-------GDLNNCIRMV----------
640015518_L8106_06195                       IE-GDLR--SPSRVESLKLAPEPLSGTEPLHYY-------GDANNCIRLV----------
648859640_OSCI_3640012                      IE-ADLR--SPSWVQFLKVAPDPASSIEPLRYY-------GDLCECIRLV----------
2508873356_Oscil6407DRAFT_00014730          IE-ADLR--SPSWVQFLKVAPDPASSIEPLRYY-------GDLCECIRLV----------
2504092741_Osc7112_6177                     IE-ADLR--SPSNVESLKIAADPLATVEPLHYY-------GNISECVLLV----------
2506346135_MicvaDRAFT_1862                  IE-ADLR--SPSNVESLKIAADPLATVEPLHYY-------GNISECVLLV----------
638107751_Tery_2688                         IE-TDLR--SPSKAESLKIFPDPDSCVEPLRYY-------GDINSCIRQV----------
2506480833_LYNGBM3L_31450                   VE-GDLR--SHSVAQAQEVIHLMDAPLEPLRFY-------GSKSECIHLA----------
647565613_MC7420_2957                       IE-ADLR--SPSQAHFHHVTPEPEAKTQPLPYY-------GSKSACIQLA----------
2510102323_Gei7105DRAFT_3578                VE-ADLR--SPSKAESLKVALDPHSLIEPLRHY------R-DFQSGMRFV----------
2509498590_Pro9006DRAFT_0077                LE-ADLR--HATGSQQLGLVPNLDSATEPLTYY-------GNVSDCLVPV----------
2509773034_Lepto7104DRAFT_0253              IE-ADLR--QPSCGDRLGVKLDPAAMAEPLHYY------AGRQQSPIQLA----------
2517693907_LEP6406DRAFT_3365                LE-ADLR--SHSQARLLGITPDEQAILEPLRYY------GGYLSDPVQMV----------
2509844778_Lepto7375DRAFT_4795              VE-ADLR--SGSKAQYLGLVNDDQVLMEPLRYY------GGSIGDNVRMV----------
641676481_cce_1994                          VE-GDLR--SPSKAQWLEVTPDPDSNLEPLRFY-------HNRTDAVNLV----------
2507502153_Cy51472_3013                     VE-GDLR--SPSKAQWLEVTPDPDSNLEPLRFY-------HNRTDAVNLV----------
640626984_CY0110_27525                      IE-GDLR--SSSKARWLEITPDPNSSLEPLRFY-------QNRADAVVLV----------
2503335332_CWat_WH8501_draft2_00062360      VE-ADLR--SPSKAQWLEVTPNPNNHLEPLRFY-------NNRTDAVSLV----------
2531849533_CWATWH0003_2441                  VE-ADLR--SPSKAQWLEVTPNPNNHLEPLRFY-------NNRTDAVSLV----------
643475556_PCC8801_2514                      VE-ADLR--APSKAKWLEVTPDSEASVEPLRYY-------GDRTGSIQLV----------
644981523_Cyan8802_3592                     VE-ADLR--APSKAKWLEVTPDSEASVEPLRYY-------GDRTGSIQLV----------
2509576278_Ple7327_4396                     VE-ADLR--SRSKASVLQVTLDLEAALEPLRYY-------ANRGEAINLV----------
641537721_MAE_37160                         VE-GDLR--SPSKAEEIGVTPDPNSFREPLLYY-------GAKSKSIRLA----------
648188704_Cyan7822_3790                     VE-ADLR--SPSKSSILQVNPDPESGLEPLRYY-------AARTEAISLV----------
2503801406_Sta7437_3306                     LE-ADLR--FSSNADLIQVELDREATGEPLNYY-------GNRNDAIRLV----------
2505785267_Chr6712_1514                     IE-ADLR--SPSNSEWVQIRHDENANQEPLKYF-------AESNDAIRLV----------
2506598905_Spi9445_1800                     VE-GDLR--SPSNAQAVKLAVDHDAQAEPLSYY-------GSRNDSVRLV----------
2506609325_Spi6313_1684                     IE-ADLR--SRSRVSSLNLAVDLDAVAEPLSYY-------GAAQSCTVLA----------
2503367585_Cyast_2162                       VE-LDLRDKNPGSRKYFQLQPDPALSSNPVSYYDFQGVSNGDLSSLIQRV----------
```

```
gi|16124419|ref|NP_418983.1|                 VD-CDLRQSAIS-----QFLKEPAPVGLLEVLNGVATLDQAIIND---------------
gi|16264834|ref|NP_437626.1|                 ID-ADIRKPGL---------------TQMITPAPRTGLVETLIGEASWPAGIKVDQR---
2508652600_Xen7305DRAFT_00047000             ID-TNLHTPKQK--AIWRIDNNL-------------GLTDILKKDVEFDNVVQAPSL---
2506480821_LYNGBM3L_31350                    VD-GDFRQATLSQRLGHH-HD------PL---KPGQPVK---------------------
2509432410_Mic7113_0575                      VD-GDFRKAELSRRLGYG-REVELADAPRFANEVQQPVS---------------------
2507482189_Cal7103DRAFT_00090960             VD-ADFRQAALSRRLGYT-QTSVTEDLS---------IS---------------------
2509801296_LepboDRAFT_0638                   VD-GDFRKAQLSHQLGYL-SDRSVGD----------QVIS---------------------
2505802696_Cal7507_4407                      VD-GDFFQAELSSKLGYI-PQ---------SHSAPTPIV---------------------
2507332342_Tol9009DRAFT_00023250             VD-GDFFQAELTHKLGYI-KE---------LNSTLTPIS---------------------
2510087202_Riv7116_2056                      VD-GDFIRAELTQDLGCT-QE---------LNITNEVVS---------------------
2510089901_Riv7116_4755                      VD-GDFRRGELSESLGYQ-PQ---------AKSRFVHNQ---------------------
2506745681_Syn7336_0399                      VD-GDFHKGELSQKLGCAWQTSNRTEASSLVDTNVVTVP---------------------
637457826_glr0454                            VD-ADFRQAGLGRRL--------QIEGQS-----SEPVA---------------------
637459188_gll1796                            VD-ADFRQAGLGRKL--------QV-GPG-----SEPVA---------------------
637461130_gll3716                            VD-ADFRQASLSRLLGHG----PQVEGTQ-----AEPVS---------------------
2503610992_Chro_0741                         ID-GDLRKAEMSRRLGKN-RIKSAANASP-----PMPVS---------------------
2503793842_Glo7428_1287                      VD-GDLRQAELSRRLGHS-KTETTMNAKQ-----S-PVS---------------------
2505800320_Cal7507_2052                      VD-GDFHQAKLSHYFGYV-LPKETV--VL-----PTPVQ---------------------
643588131_Cyan7425_5174                      VD-GDLRRARLTEQLSDRYQ-------GSWNPRETAVVP---------------------
643167924_AmaxDRAFT_0301                     --DADIYGP--NDPTMLGLADTQVMVQQTPEG----------------------------
648389440_APCC8_010100021805                 --DADIYGP--NDPTMLGLADTQVMVQQTPEG----------------------------
646130866_AplaP_010100015993                 --DADIYGP--NDPTMLGLADTQVMVQQTPEG----------------------------
650384994_NIES39_D01080                      --DADIYGP--NDPTMLGLADTQVMVQQTPEG----------------------------
640015631_L8106_17697                        --DADIYGP--NDPTMMGLSDYQVIVHQGPQG----------------------------
647567948_MC7420_2316                        --DADIYGP--NTAAMLGLADAKVMVQQGPQG----------------------------
638106387_Tery_1165                          --DADIYGP--NDPTMLGLEDAQVMVQQGESG----------------------------
648857672_OSCI_2870002                       --DADIYGP--NDPTMLGLAEAKVMVTKGPQG----------------------------
2508875395_Oscil6407DRAFT_00035160           --DADIYGP--NDPTMLGLAEAKVMVTKGPQG----------------------------
2509801138_LepboDRAFT_0480                   --DADIYGP--NAPTMLGLEGSGVAVTQTEQG----------------------------
2512979571_Fis9431DRAFT_3900                 --DADIYGP--NDPTMLGLADAQIVVRSSEKG----------------------------
2517061293_PCC9339DRAFT_02148                --DADIYGP--NDPTMLGLADAQIVVRSSEKG----------------------------
2505768399_FJSC11DRAFT_3149                  --DADIYGP--NDPTMLGLADAQIVVRTSDQG----------------------------
2509782632_Mic7126DRAFT_3383                 --DADIYGP--NDPTMLGLAEAEIVVRPSATG----------------------------
2504683355_Cri9333_0934                      --DADIYGP--NAPTMLGLANAQIAVRQGANG----------------------------
2503606101_GEI7407_0138                      --DADIYGP--NAPTMLGLTEAQVSVQKSDQG----------------------------
2509502622_Pro9006DRAFT_4121                 --DADIYGP--NAPNMLQLADAHLTVL---EG----------------------------
2509778293_Lepto7104DRAFT_5514               --DADIYGP--NAPMMMGLSEAQVVVK---QG----------------------------
2517694920_LEP6406DRAFT_4378                 --DADIYGP--NVPTMLGLEGAGMTVRPGPQG----------------------------
647577822_S7335_3227                         --DADIYGP--NAPTMMGLGESNVIVREDKGE----------------------------
2503637469_PCC7418_2732                      --DADIYGP--NAPSMLGLAEAKVRVQESPQG----------------------------
2509552467_Dacsa_0081                        --DADIYGP--NAPSMLGLAEAKVRVQESPKG----------------------------
2503367528_Cyast_2106                        --DADIYGP--NAPTMLGLLDAPINVTKSPTG----------------------------
2503887475_Lepto7376_1861                    --DADIYGP--NVPNMMGVGEVDIKVDKTDGK----------------------------
637688026_PMN2A_1153                         --DADIYGP--NTPYMLGVSEITPEVSGSGAE----------------------------
640085320_NATL1_20271                        --DADIYGP--NTPYMLGVSEITPEVSGSGAE----------------------------
641286083_P9211_17011                        --DADIYGP--NTPTMLGVAQKTPEVFGQGAE----------------------------
640547039_SynRCC307_2084                     --DADIYGP--NAPTMLGVADRTPEVEGEGES----------------------------
637798215_PMT9312_1673                       --DADIYGP--NTPSMMGVAEQNPKVTEGSGS----------------------------
640161373_P9301_17721                        --DADIYGP--NTPSMMGVAEQNPKVTEGSGS----------------------------
640944474_P9215_18531                        --DADIYGP--NTPSMMGVAEQNPKVTEGTGT----------------------------
647672827_P9202_63                           --DADIYGP--NTPSMMGVAEQNPKVTEGTGT----------------------------
640079518_A9601_17881                        --DADIYGP--NTPSMMGVAEQNPKVTEGSGS----------------------------
637450610_PMM1581                            --DADIYGP--NTPSMLGVTEENPKVTDGSGN----------------------------
640081464_P9515_17681                        --DADIYGP--NTPAMLGVTEENPTVTDGSGN----------------------------
646569576_Ava_4074                           --DADVYGP--NVPQMLGLGQADVEVIQTPTG----------------------------
2505803290_Cal7507_4972                      --DADIYGP--NVPQMLGLGQADIQVINTPRG----------------------------
2517700475_SYN7509DRAFT_4594                 --DADVYGP--NVPQMLGLGRAEVKVIETSNG----------------------------
2506747473_Syn7336_2171                      --DADIYGP--NVPQMLGLGRSDVTVTETPEG----------------------------
gi|1172092|gb|AAA86371.1|                    -----LRGPS--------------------------------------------------
gi|15597431|ref|NP_250925.1|                 --GGEPAQPG--------------------------------------------------
637450246_PMM1225                            ID-TNFGSPILD--KFFNLNSSI-------------GVLDYLANEKIQISQIIN------
gi|53721457|ref|YP_110442.1|                 GDLARLRH----------------------------------------------------
638961258_RS9917_04770                       ------------------------------------------------------------
639888778_RS9916_38577                       ------------------------------------------------------------
637444523_SYNW0419                           ------------------------------------------------------------
640545089_SynRCC307_0169                     ------------------------------------------------------------
640084152_NATL1_08771                        ------------------------------------------------------------
647672433_P9202_965                          ------------------------------------------------------------
637797888_PMT9312_1355                       VPLGDIENISLNKIENYIQTND--------------------------------------
640944066_P9215_14561                        IDFT--------------------------------------------------------
gi|397405|emb|CAA52655.1|                    AVIEDHRD----------------------------------------------------
gi|455023|gb|AAB51624.1|                     AVIEDHRD----------------------------------------------------
gi|1107924|emb|CAA62141.1|                   -EHADVRETA--------------------------------------------------
gi|53720415|ref|YP_109401.1|                 ERVAEPNEPDV-------------------------------------------------
gi|45297|emb|CAA38731.1|                     EVISQPSKPDW-------------------------------------------------

2505798636_Cal7507_0387                      QV------------------------------------------------IQQM------
2509783408_Mic7126DRAFT_4160                 QV------------------------------------------------IQEV------
648052066_Aazo_4925                          QV------------------------------------------------IQQL------
2504133443_Anacy_3769                        QV------------------------------------------------IQQL------
2506494313_Ana7108_4449                      QV------------------------------------------------IQQI------
2512980750_Fis9431DRAFT_5079                 EA------------------------------------------------IRPA------
2517062414_PCC9339DRAFT_03271                EA------------------------------------------------IRPA------
2505767894_FJSC11DRAFT_3537                  EA------------------------------------------------IRPA------
2516145585_FIS9605DRAFT_02588                EA------------------------------------------------VRPA------
2504096080_Cal6303_3081                      EV------------------------------------------------IQKI------
2507333046_Tol9009DRAFT_00030320             EV------------------------------------------------IRKV------
```

```
2507476338_Cal7103DRAFT_00032380      EV-----------------------------------------------IRQY------
2505802134_Cal7507_3852               EV-----------------------------------------------IQQL------
2509782674_Mic7126DRAFT_3425          ET-----------------------------------------------IQQL------
2509771979_CylstDRAFT_5939            EV-----------------------------------------------IRQL------
2503738747_Nos7107_0132               EA-----------------------------------------------IQEI------
2509809495_Nos7524_1926               EV-----------------------------------------------VRKL------
640026730_N9414_07896                 KV-----------------------------------------------VKQL------
2517239228_Mas10914DRAFT_0471         TV-----------------------------------------------IRQA------
2510087477_Riv7116_2331               SA-----------------------------------------------IQKI------
2510087323_Riv7116_2177               EV-----------------------------------------------MQDL------
2507477177_Cal7103DRAFT_00040780      QI-----------------------------------------------IQEA------
637235631_all5222                     EV-----------------------------------------------IHKL------
2506482271_LYNGBM3L_49700             DV-----------------------------------------------IEQKLP----
641611510_SYNPCC7002_A1500            NY-----------------------------------------------LQKPMP----
2509842234_Lepto7375DRAFT_2251        GI-----------------------------------------------LQKAMP----
647107641_CRC_03354                   EV-----------------------------------------------MQKL------
647110196_CRD_02439                   EV-----------------------------------------------IQHV------
2509804452_LepboDRAFT_3794            DV-----------------------------------------------VQRINALATA
647567980_MC7420_2223                 DV-----------------------------------------------IQP-------
2509437593_Mic7113_5758               DV-----------------------------------------------IQRCSF----
2506479760_LYNGBM3L_56010             DV-----------------------------------------------IQR-------
647567242_MC7420_5618                 QA-----------------------------------------------IQRL------
2509436287_Mic7113_4452               QA-----------------------------------------------IQRL------
2503796318_Glo7428_3737               DA-----------------------------------------------IQR-------
2517697153_SYN7509DRAFT_1270          EA-----------------------------------------------IQR-------
2503615076_Chro_4796                  DL-----------------------------------------------IQR-------
2509422734_Oscil6304_3527             DS-----------------------------------------------IQQ-------
2506601543_Spi9445_4406               DI-----------------------------------------------IQP-FP----
2506609271_Spi6313_1630               DV-----------------------------------------------IQR-SP----
640014711_L8106_15385                 EV-----------------------------------------------IQTV------
2509507761_Osc10802DRAFT_1411         EA-----------------------------------------------IQK-------
2509420889_Oscil6304_1682             EA-----------------------------------------------IQR-------
2503798162_Sta7437_0103               AA-----------------------------------------------IQKV------
2505785430_Chr6712_1674               TV-----------------------------------------------TQQP------
2509711143_Pleur7313DRAFT_05131       SA-----------------------------------------------IIKV------
2508648278_Xen7305DRAFT_00003760      AA-----------------------------------------------IKQV------
2508646941_GLO73106DRAFT_00031960     SV-----------------------------------------------VKSV------
2509573356_Ple7327_1473               EA-----------------------------------------------IQQV------
641537293_MAE_32940                   EA-----------------------------------------------IQSL------
643482531_PCC7424_4310                DV-----------------------------------------------IQKV------
648185689_Cyan7822_0712               KA-----------------------------------------------IQKV------
2509502225_Pro9006DRAFT_3724          QV-----------------------------------------------IQVVGL----
643584098_Cyan7425_1087               QV-----------------------------------------------IQQ-------
2503742029_Nos7107_3348               NI-----------------------------------------------IQ--------
2504097563_Cal6303_4545               DV-----------------------------------------------IGQV------
2507478127_Cal7103DRAFT_00050290      DS-----------------------------------------------VQQL------
2510089327_Riv7116_4181               DA-----------------------------------------------LGQS------
2517239184_Mas10914DRAFT_0427         AV-----------------------------------------------VGQL------
2507335438_Tol9009DRAFT_00054260      KV-----------------------------------------------IQQS------
2504086624_Osc7112_0134               QV-----------------------------------------------ILR-------
2506346341_MicvaDRAFT_2066            QV-----------------------------------------------ILR-------
648859632_OSCI_3640004                EV-----------------------------------------------MVQ-------
2508872900_Oscil6407DRAFT_00010170    EV-----------------------------------------------MVQ-------
643171775_AmaxDRAFT_4132              TL-----------------------------------------------IRR-------
648386075_APCC8_010100004460          TL-----------------------------------------------IRR-------
650384684_NIES39_C03520               SL-----------------------------------------------IRR-------
646131598_AplaP_010100019750          SL-----------------------------------------------IRR-------
640018815_L8106_14065                 DV-----------------------------------------------VQQ-------
638107046_Tery_1925                   AA-----------------------------------------------IQR-------
2506483316_LYNGBM3L_63940             RA-----------------------------------------------IQQV------
648856139_OSCI_1010012                DA-----------------------------------------------IQRS------
2508875360_Oscil6407DRAFT_00034810    DA-----------------------------------------------IQRS------
2509510258_Osc10802DRAFT_3909         DV-----------------------------------------------IQRSHS----
2509438066_Mic7113_6231               DA-----------------------------------------------IVRSSV----
2504090796_Osc7112_4249               DA-----------------------------------------------IQRA------
2506345448_MicvaDRAFT_4285            DA-----------------------------------------------IQRA------
2504091687_Osc7112_5131               EA-----------------------------------------------IQR-------
647568459_MC7420_6195                 EA-----------------------------------------------IQKS------
647572642_MC7420_919                  EA-----------------------------------------------IHKS------
640014779_L8106_06284                 DA-----------------------------------------------IQR-------
2509421881_Oscil6304_2674             EV-----------------------------------------------IQRL------
641253939_AM1_4861                    ST-----------------------------------------------IHPL------
2514735482_ACCM5_010100007602         ST-----------------------------------------------IHPL------
2510441931_Cha6605_5932               DT-----------------------------------------------VQLL------
2503611061_Chro_0810                  EA-----------------------------------------------EDN--GAFHE
2503613235_Chro_2969                  VA-----------------------------------------------IKN--SDRAE
2512979625_Fis9431DRAFT_3954          NV-----------------------------------------------IER-------
2517059612_PCC9339DRAFT_00465         NV-----------------------------------------------IER-------
2505768848_FJSC11DRAFT_1825           NV-----------------------------------------------IER-------
2516149265_FIS9605DRAFT_06272         NV-----------------------------------------------IER-------
2517241827_Mas10914DRAFT_3070         NV-----------------------------------------------IER-------
2507335136_Tol9009DRAFT_00051240      NV-----------------------------------------------IER-------
2504095135_Cal6303_2139               NV-----------------------------------------------IER-------
2507476798_Cal7103DRAFT_00036990      NV-----------------------------------------------IER-------
2510085606_Riv7116_0460               NV-----------------------------------------------IEK-------
2503739631_Nos7107_1000               NV-----------------------------------------------IER-------
2505801704_Cal7507_3428               HV-----------------------------------------------IER-------
2505804241_Cal7507_5918               NV-----------------------------------------------IEK-------
```

```
643168344_AmaxDRAFT_0719                    RV------------------------------------------------IQP-------
648390241_APCC8_010100025948                RV------------------------------------------------IQP-------
646129996_AplaP_010100011596                RV------------------------------------------------IQP-------
650383923_NIES39_A06230                     RV------------------------------------------------IQP-------
640017922_L8106_10512                       HV------------------------------------------------IQQ-------
2510101197_Gei7105DRAFT_2452                EA------------------------------------------------IEQ-------
2509573281_Ple7327_1398                     HA------------------------------------------------IQR-------
2506597130_Spi9445_0041                     AA------------------------------------------------IQR-------
2503636620_PCC7418_1894                     QV------------------------------------------------IQP-------
2506607995_Spi6313_0371                     -A------------------------------------------------IQA-------
643585148_Cyan7425_2153                     QA------------------------------------------------IQQI------
2504681848_Pse7367_3384                     PL------------------------------------------------LQR-------
647577962_S7335_3828                        ---------------------------------------------------------ALL
2509846600_Lepto7375DRAFT_6617              ------------------------------------------------------------
2509774530_Lepto7104DRAFT_1750              NV------------------------------------------------IKR-------
2509804417_LepboDRAFT_3759                  TL------------------------------------------------IQQ-------
2531850144_CWATWH0003_3517                  QA------------------------------------------------IE--------
2509777916_Lepto7104DRAFT_5137              DL------------------------------------------------VQTL------
648856194_OSCI_1040007                      DI------------------------------------------------IQR-------
2508875344_Oscil6407DRAFT_00034650          DI------------------------------------------------IQR-------
2504087727_Osc7112_1201                     EM------------------------------------------------IQR-------
2506348042_MicvaDRAFT_3627                  EI------------------------------------------------IQP-------
2509438406_Mic7113_6573                     EF------------------------------------------------IQK-------
647571294_MC7420_5066                       DF------------------------------------------------IER-------
2506481927_LYNGBM3L_44990                   -V------------------------------------------------MER-------
640019302_L8106_03117                       IL------------------------------------------------IQQ-------
2509707887_Pleur7313DRAFT_01875             QV------------------------------------------------IQP-------
641251898_AM1_2808                          ------------------------------------------------------------
2514737774_ACCM5_010100019113               EL------------------------------------------------IQR-------
641251897_AM1_2807                          EL------------------------------------------------IQR-------
2512634931_Chl7702DRAFT_3806                SL------------------------------------------------IQV-------
2516146228_FIS9605DRAFT_03233               SN------------------------------------------------TQL-------
641675948_cce_1468                          AI------------------------------------------------IES-------
2507501641_Cy51472_2500                     AI------------------------------------------------IES-------
640626055_CY0110_29394                      AI------------------------------------------------IES-------
2531850936_CWATWH0003_4939                  AV------------------------------------------------IET-------
643476430_PCC8801_3415                      EV------------------------------------------------IQS-------
644980660_Cyan8802_2701                     EV------------------------------------------------IQS-------
643478515_PCC7424_0240                      DV------------------------------------------------IQP-------
648186851_Cyan7822_1903                     DV------------------------------------------------IES-------
2509575480_Ple7327_3597                     KV------------------------------------------------IQS-------
2508645044_GLO73106DRAFT_00012970           ------------------------------------------------------------
2509773763_Lepto7104DRAFT_0983              DI------------------------------------------------TQRLSW----
2503745907_Cyan10605_1646                   NA------------------------------------------------IQSP------
2509874395_Syn6308DRAFT_1102                SV------------------------------------------------MQQV------
644979531_Cyan8802_1534                     ------------------------------------------------------------
2503745344_Cyan10605_1086                   DV------------------------------------------------IQSF------
2509873750_Syn6308DRAFT_0457                EV------------------------------------------------IQTS------
2505785711_Chr6712_1954                     KV------------------------------------------------IQA-------
2509706287_Pleur7313DRAFT_00275             -Y------------------------------------------------IQQ-------
640027738_N9414_07903                       ------------------------------------------------------------
2506482929_LYNGBM3L_58920                   ----------------------------------------------------------VN
637461200_glr3785                           ----------------------------------------------------------MH
2503887239_Lepto7376_1629                   SY------------------------------------------------VKKVF-----
638113739_sync_0150                         DL------------------------------------------------CKQV------
638963744_RS9917_02426                      DV------------------------------------------------VQTV------
2507493170_Syn8016_1895                     QA------------------------------------------------LQPI------
638960084_WH5701_13945                      SL------------------------------------------------LQTV------
650128418_SCB01_010100004274                AL------------------------------------------------LQWP------
650132168_SCB02_010100007823                EL------------------------------------------------IQWP------
2508551611_Cyagr_1223                       DF------------------------------------------------IHSI------
640081072_P9515_13861                       DV------------------------------------------------IQSV------
648050855_Aazo_3125                         -------------------------------------ATL------PNQI----------
2504129904_Anacy_0284                       -------------------------------------VDL------PQQI----------
2506490031_Ana7108_0216                     -------------------------------------VNI------PNQI----------
2509770736_CylstDRAFT_4696                  -------------------------------------VTL------PHQI----------
642600344_Npun_R1070                        -------------------------------------ASL------PSQI----------
2505799741_Cal7507_1481                     -------------------------------------ASL------PNQI----------
2509779661_Mic7126DRAFT_0411                -------------------------------------ATL------PNQI----------
2503739922_Nos7107_1290                     -------------------------------------VTL------PNEI----------
640027761_N9414_00965                       -------------------------------------VSL------PNQI----------
637233217_alr2833                           -------------------------------------ITL------PEQI----------
646566603_Ava_1116                          -------------------------------------ITL------PEQI----------
2509808002_Nos7524_0433                     -------------------------------------ITL------PHQI----------
2517241466_Mas10914DRAFT_2709               -------------------------------------VTL------PNQI----------
647105946_CRC_01573                         -------------------------------------ANI------PDQI----------
647109494_CRD_02102                         -------------------------------------ANI------PDQI----------
2507332201_Tol9009DRAFT_00021840            -------------------------------------IAL------PNQI----------
2512975951_Fis9431DRAFT_0279                -------------------------------------STL------PNQI----------
2517062640_PCC9339DRAFT_03498               -------------------------------------STL------PNQI----------
2505767181_FJSC11DRAFT_4188                 -------------------------------------STL------PNQI----------
2516146836_FIS9605DRAFT_03842               -------------------------------------STL------PNQI----------
2512634275_Chl7702DRAFT_3149                -------------------------------------TAL------PNQI----------
2510089385_Riv7116_4239                     -------------------------------------VAL------PNQI----------
2504094820_Cal6303_1825                     -------------------------------------TPL------PEHI----------
2507482515_Cal7103DRAFT_00094220            -------------------------------------VDL------PSQI----------
2503612562_Chro_2300                        -------------------------------------ATL------PIHN----------
2517695945_SYN7509DRAFT_0062                -------------------------------------DTA------PNHA----------
```

```
2503608186_GEI7407_2184                    -------------------------------------A-L------FQHG----------
2504685208_Cri9333_2761                    -------------------------------------ATV------PIHS----------
2509431928_Mic7113_0093                    -------------------------------------APL------PIQT----------
2506479159_LYNGBM3L_28310                  -------------------------------------TAT------PSPV----------
640017638_L8106_27951                      -------------------------------------VSMPHL-----------------
2509806250_LepboDRAFT_5593                 -------------------------------------RAITQGAIQP-------------
2504583836_Pse7429DRAFT_1713               -------------------------------------GSIDSRDV---------------
641251010_AM1_1910                         -------------------------------------VTLAELQTTPQWVYMRWDESELD
2514735759_ACCM5_010100008979              -------------------------------------VTLAELQTTPQWVYMRWDESELD
2509847582_Lepto7375DRAFT_7599             -------------------------------------LA---------------RWNKTK
648051793_Aazo_4516                        -------------------------------------IKTQFQNY---------------
2504134691_Anacy_5006                      -------------------------------------VKTQVHNY---------------
2506494605_Ana7108_4733                    -------------------------------------IKNQVNNY---------------
647107726_CRC_03451                        -------------------------------------IKTQFPNY---------------
642604187_Npun_R5250                       -------------------------------------TTTHFQDY---------------
2509767727_CylstDRAFT_1687                 -------------------------------------TDANFDDY---------------
2507335141_Tol9009DRAFT_00051290           -------------------------------------TNNGVPDY---------------
640028130_N9414_07219                      -------------------------------------TNTQVQDY---------------
637230414_all0059                          -------------------------------------TNTEVQDY---------------
646568157_Ava_2661                         -------------------------------------TNTEVQDY---------------
2509811005_Nos7524_3436                    -------------------------------------TDTTIQDY---------------
2503739635_Nos7107_1004                    -------------------------------------TSNHIHNY---------------
2509781657_Mic7126DRAFT_2408               -------------------------------------TNSQIHEY---------------
2517243237_Mas10914DRAFT_4480              -------------------------------------TNSSVKEY---------------
2512634393_Chl7702DRAFT_3267               -------------------------------------ANTCFRDY---------------
2505769181_FJSC11DRAFT_2156                -------------------------------------SNTSDHNY---------------
2512977368_Fis9431DRAFT_1696               -------------------------------------TDTSDQRY---------------
2517063601_PCC9339DRAFT_04460              -------------------------------------TDTSDQDY---------------
2516147062_FIS9605DRAFT_04068              -------------------------------------ANTSIQDY---------------
2507482923_Cal7103DRAFT_00098300           -------------------------------------AHSTFKDY---------------
2510085595_Riv7116_0449                    -------------------------------------RNSEVNDY---------------
2503614917_Chro_4637                       -------------------------------------ADVRERDC---------------
2504093446_Cal6303_0467                    -------------------------------------ENPSFQDY---------------
647578236_S7335_5395                       -------------------------------------AT--------------------R
2503798844_Sta7437_0777                    -------------------------------------TASLNIQ----------------
2509708827_Pleur7313DRAFT_02815            -------------------------------------VTYPVIE----------------
637009749_sll0923                          ------------------------------------------------------------
2503800689_Sta7437_2597                    -----N------------------------------------------------------
2509710958_Pleur7313DRAFT_04946            -----N------------------------------------------------------
2505786361_Chr6712_2601                    -----N------------------------------------------------------
2508650693_Xen7305DRAFT_00027920           -----N------------------------------------------------------
2506480412_LYNGBM3L_19610                  -----S------------------------------------------------------
2509512444_Osc10802DRAFT_6095              -----H------------------------------------------------------
643587900_Cyan7425_4938                    -----N------------------------------------------------------
2504582150_Pse7429DRAFT_3127               ----GN------------------------------------------------------
2504679882_Pse7367_1451                    ----GC------------------------------------------------------
2507088905_Pse6802_3551                    ----NS------------------------------------------------------
2508687870_Syn7502_01070                   QFSAGY------------------------------------------------------
2506748948_Syn7336_3628                    ------------------------------------------------------------
643484053_PCC7424_5769                     NLRIVSDCQENI-----------NSLQSSSQTIAVNGTKSIFARV-LTSPTAPINQESGT
648199484_Cyan7822_5718                    TATNGINPQQELTFLSSVNGMKTNKIKNSNHKIT-NNLNSIFAKVSLKANLMPNSSLSES
2510436294_Cha6605_0295                    ------------------------------------------------------------
637314397_tll1767                          ------------------------------------------------------------
637313820_tll1199                          ------------------------------------------------------------
2512980079_Fis9431DRAFT_4408               ------------------------------------------------------Q---VP
2517061743_PCC9339DRAFT_02599              ------------------------------------------------------K---VA
2505770929_FJSC11DRAFT_1293                ------------------------------------------------------Q---VP
2516148164_FIS9605DRAFT_05171              ------------------------------------------------------K---VP
2512634416_Chl7702DRAFT_3290               ------------------------------------------------------Q---IP
2505800360_Cal7507_2092                    ------------------------------------------------------Q---VP
2509780096_Mic7126DRAFT_0847               ------------------------------------------------------Q---IA
2505800523_Cal7507_2254                    ------------------------------------------------------Q---VG
2507478259_Cal7103DRAFT_00051610           ------------------------------------------------------Q---ME
637234831_all4432                          ------------------------------------------------------Q---VT
646566875_Ava_1386                         ------------------------------------------------------Q---VT
642600743_Npun_R1496                       ------------------------------------------------------Q---VS
2507334816_Tol9009DRAFT_00048030           ------------------------------------------------------Q---VS
2507476704_Cal7103DRAFT_00036050           ------------------------------------------------------Q---IA
2510090628_Riv7116_5482                    ------------------------------------------------------H---VN
2506483552_LYNGBM3L_64290                  ------------------------------------------------------E---AQ
2517697529_SYN7509DRAFT_1646               ------------------------------------------------------K---AD
2503608035_GEI7407_2035                    ------------------------------------------------------Q---TD
2509433692_Mic7113_1857                    ------------------------------------------------------R---SE
640015133_L8106_09871                      ------------------------------------------------------K---VE
2503795055_Glo7428_2485                    ------------------------------------------------------Q---AE
2509506717_Osc10802DRAFT_0363              ------------------------------------------------------Q---AD
2503614965_Chro_4685                       ------------------------------------------------------Q---VD
2510091904_Riv7116_6758                    ------------------------------------------------------Q---VE
2503612629_Chro_2367                       ------------------------------------------------------G---TD
2503612419_Chro_2158                       ------------------------------------------------------Q---AG
2506493946_Ana7108_4087                    ------------------------------------------------------Q---IE
2509803997_LepboDRAFT_3339                 ------------------------------------------------------Q---TN
2512979777_Fis9431DRAFT_4106               ------------------------------------------------------Q---AV
2517062202_PCC9339DRAFT_03059              ------------------------------------------------------Q---AV
2505770300_FJSC11DRAFT_0671                ------------------------------------------------------Q---GV
2507334440_Tol9009DRAFT_00044270           ------------------------------------------------------Q---DE
2510088639_Riv7116_3493                    ------------------------------------------------------Q---DE
2505803639_Cal7507_5320                    ------------------------------------------------------E---NE
```

```
2509782388_Mic7126DRAFT_3139              ------------------------------------------------------E---NE
2507335607_Tol9009DRAFT_00055950          ------------------------------------------------------E---NE
2510088989_Riv7116_3843                   ------------------------------------------------------E---ND
2504096650_Cal6303_3648                   ------------------------------------------------------R---VK
2507481772_Cal7103DRAFT_00086780          ------------------------------------------------------Q---DR
2509803885_LepboDRAFT_3227                ------------------------------------------------------Q---SE
647579433_S7335_2031                      ------------------------------------------------------Q---SS
2509846113_Lepto7375DRAFT_6130            ------------------------------------------------------Q---VP
2512978854_Fis9431DRAFT_3183              ------------------------------------------------------Q---VE
2516143706_FIS9605DRAFT_00708             ------------------------------------------------------Q---VE
642600641_Npun_F1381                      ------------------------------------------------------E---AK
2509770219_CylstDRAFT_4179                ------------------------------------------------------Q---TE
2504094091_Cal6303_1107                   ------------------------------------------------------Q---SD
2507479320_Cal7103DRAFT_00062230          ------------------------------------------------------Q---SD
2510085390_Riv7116_0244                   ------------------------------------------------------Q---VE
642601572_Npun_F2453                      ------------------------------------------------------------
2503611448_Chro_1193                      ------------------------------------------------------Q---TE
2503796978_Glo7428_4384                   ------------------------------------------------------Q---VE
2504087786_Osc7112_1259                   ------------------------------------------------------Q---AG
643584136_Cyan7425_1127                   ------------------------------------------------------Q---TD
647105865_CRC_01492                       ------------------------------------------------------Q---TQ
647108284_CRD_00419                       ------------------------------------------------------R---TQ
2505800344_Cal7507_2076                   ------------------------------------------------------Q---AE
2509781889_Mic7126DRAFT_2640              ------------------------------------------------------Q---AE
2507481279_Cal7103DRAFT_00081850          ------------------------------------------------------Q---AE
637233240_alr2856                         ------------------------------------------------------Q---AT
646566532_Ava_1045                        ------------------------------------------------------Q---AT
640028261_N9414_00005                     ------------------------------------------------------Q---TN
642603803_Npun_R4851                      ------------------------------------------------------Q---AE
2509770699_CylstDRAFT_4659                ------------------------------------------------------Q---AE
2507478691_Cal7103DRAFT_00055940          ------------------------------------------------------K---ID
641249527_AM1_0407                        ------------------------------------------------------Q---KD
2514736728_ACCM5_010100013856             ------------------------------------------------------Q---KD
643586770_Cyan7425_3796                   ------------------------------------------------------H---SS
2509555626_Dacsa_3240                     ------------------------------------------------------K----E
2509775597_Lepto7104DRAFT_2817            ------------------------------------------------------E---TH
2506492920_Ana7108_3067                   ------------------------------------------------------Q---LD
2512631781_Chl7702DRAFT_0655              ------------------------------------------------------H---LD
2504685956_Cri9333_3503                   ------------------------------------------------------Q---SD
2510436704_Cha6605_0705                   ----------------------------------------------------------CD
2507482822_Cal7103DRAFT_00097290          ------------------------------------------------------R---CD
2510087184_Riv7116_2038                   ------------------------------------------------------K---AN
2506745596_Syn7336_0315                   ----------------------------------------------------------CH
2503796286_Glo7428_3705                   ------------------------------------------------------I---GV
642604427_Npun_F5505                      ------------------------------------------------------Q---TE
2503612394_Chro_2133                      ------------------------------------------------------Q---AQ
2512632720_Chl7702DRAFT_1594              ------------------------------------------------------E---GQ
2509807906_Nos7524_0337                   ------------------------------------------------------Q---SQ
2509770373_CylstDRAFT_4333                ------------------------------------------------------E---VE
646570355_Ava_4846                        ------------------------------------------------------Q---VE
2510438837_Cha6605_2838                   ------------------------------------------------------K---SK
2506477923_LYNGBM3L_10200                 ------------------------------------------------------E---VP
2509574529_Ple7327_2646                   ------------------------------------------------------I---TL
643170532_AmaxDRAFT_2895                  ------------------------------------------------------E---AT
648389308_APCC8_010100021105              ------------------------------------------------------E---AT
646129623_AplaP_010100009700              ------------------------------------------------------E---AT
650384826_NIES39_C04940                   ------------------------------------------------------E---AT
2503801641_Sta7437_3540                   ------------------------------------------------------Q---KP
2509711085_Pleur7313DRAFT_05073           ------------------------------------------------------Q---LS
2503801821_Sta7437_3719                   ------------------------------------------------------K---TS
2505786500_Chr6712_2739                   ------------------------------------------------------Q---VS
2508650623_Xen7305DRAFT_00027220          ------------------------------------------------------K---IA
2509706990_Pleur7313DRAFT_00978           ------------------------------------------------------K---KS
2505787175_Chr6712_3399                   ------------------------------------------------------Q---KP
2508650743_Xen7305DRAFT_00028420          ------------------------------------------------------E---AE
2509711668_Pleur7313DRAFT_05656           ------------------------------------------------------K---IK
2509712372_Pleur7313DRAFT_06363           ------------------------------------------------------E---IE
2505785156_Chr6712_1404                   ------------------------------------------------------E---VE
2503799529_Sta7437_1457                   ------------------------------------------------------E---VE
2506749054_Syn7336_3731                   ----------------------------------------------------------AQ
2503799932_Sta7437_1855                   ------------------------------------------------------Q---NL
2505786145_Chr6712_2386                   ------------------------------------------------------E---AT
641538163_MAE_41520                       ------------------------------------------------------K---TQ
2507085801_Pse6802_0485                   -------------------------------------------------------TPEG-
2507089189_Pse6802_3805                   ------------------------------------------------------ISEEV-
2508689585_Syn7502_02785                  ------------------------------------------------------TQLE--
2504679565_Pse7367_1141                   ------------------------------------------------------Q---SP
2503802282_Sta7437_4176                   ------------------------------------------------------I---NP
2509709361_Pleur7313DRAFT_03349           ------------------------------------------------------I---DP
2508651492_Xen7305DRAFT_00035910          ------------------------------------------------------Q---AL
2505784713_Chr6712_0967                   ------------------------------------------------------N---LD
2514739634_ACCM5_010100028418             ------------------------------------------------------SIRPAE
637461727_glr4310                         ----------------------------------------------------------AD
641253915_AM1_4837                        ----------------------------------------------------------AD
2514739407_ACCM5_010100027286             ----------------------------------------------------------AD
2509712141_Pleur7313DRAFT_06132           ------------------------------------------------------GI----
2506482932_LYNGBM3L_58950                 ------------------------------------------------------------
2506608339_Spi6313_0712                   ----------------------------------------------------------II
```

```
641249511_AM1_0391                            ------------------------------------------------------HFNYHQ
2514736711_ACCM5_010100013771                 ------------------------------------------------------NVVEQH
2509500847_Pro9006DRAFT_2342                  ------------------------------------------------------EPVTWE
2504681110_Pse7367_2660                       ----------------------------------------------------------AN
2509874159_Syn6308DRAFT_0866                  ------------------------------------------------------------
2509428796_Syn6312_0755                       ------------------------------------------------------LPYQKF
2508653159_Xen7305DRAFT_00052600              ------------------------------------------------------R---FA
2512978631_Fis9431DRAFT_2960                  ------------------------------------------------------R----S
2517061062_PCC9339DRAFT_01917                 ------------------------------------------------------R----S
2505768802_FJSC11DRAFT_1780                   ------------------------------------------------------R----S
2516146713_FIS9605DRAFT_03719                 ------------------------------------------------------I----S
2507336787_Tol9009DRAFT_00067760              ------------------------------------------------------R----S
2517242598_Mas10914DRAFT_3841                 ------------------------------------------------------I----S
2507476104_Cal7103DRAFT_00030040              ------------------------------------------------------I----S
2510090822_Riv7116_5676                       ------------------------------------------------------R----N
2509766470_CylstDRAFT_0429                    ------------------------------------------------------L----S
637233445_alr3059                             ------------------------------------------------------R----P
646566337_Ava_0852                            ------------------------------------------------------R----P
2509812587_Nos7524_5019                       ------------------------------------------------------------
2507480867_Cal7103DRAFT_00077730              ------------------------------------------------------R----H
2504094392_Cal6303_1407                       ------------------------------------------------------K----T
2506481239_LYNGBM3L_38240                     ------------------------------------------------------Q----T
2503613794_Chro_3525                          ------------------------------------------------------L----A
2510437494_Cha6605_1495                       ------------------------------------------------------L----T
2509773567_Lepto7104DRAFT_0787                ------------------------------------------------------H----D
641254791_AM1_5726                            ------------------------------------------------------------
2514737653_ACCM5_010100018486                 ------------------------------------------------------------
637459597_gll2202                             ------------------------------------------------------------
gi|4512007|gb|AAD21564.1|                     ----------------------------------------------------------AA
gi|53987110|gb|AAV27324.1|                    ----------------------------------------------------------VD
637230853_all0493                             ------------------------------------------------------------
646568404_Ava_2908                            ------------------------------------------------------------
2509812367_Nos7524_4799                       ------------------------------------------------------------
642599777_Npun_F0459                          ------------------------------------------------------------
2509772030_CylstDRAFT_5990                    ------------------------------------------------------------
2503740929_Nos7107_2283                       ------------------------------------------------------------
640027950_N9414_23213                         ------------------------------------------------------------
2505798270_Cal7507_0021                       ------------------------------------------------------------
2509781264_Mic7126DRAFT_2015                  ------------------------------------------------------------
2507337082_Tol9009DRAFT_00070720              ------------------------------------------------------------
2517243673_Mas10914DRAFT_4916                 ------------------------------------------------------------
2504133191_Anacy_3523                         ------------------------------------------------------------
2506492757_Ana7108_2905                       ------------------------------------------------------------
648049213_Aazo_0828                           ------------------------------------------------------------
2512978956_Fis9431DRAFT_3285                  ------------------------------------------------------------
2517059344_PCC9339DRAFT_00197                 ------------------------------------------------------------
2505768976_FJSC11DRAFT_1951                   ------------------------------------------------------------
2516145655_FIS9605DRAFT_02658                 ------------------------------------------------------------
2512633064_Chl7702DRAFT_1938                  ------------------------------------------------------------
2510085904_Riv7116_0758                       ------------------------------------------------------------
2504093110_Cal6303_0131                       ------------------------------------------------------------
2507480421_Cal7103DRAFT_00073250              ------------------------------------------------------------
2503609163_GEI7407_3152                       ------------------------------------------------------------
2509421114_Oscil6304_1907                     ------------------------------------------------------------
2509805590_LepboDRAFT_4932                    ------------------------------------------------------------
646129274_AplaP_010100007922                  ------------------------------------------------------------
650387413_NIES39_K02750                       ------------------------------------------------------------
643168837_AmaxDRAFT_1210                      ------------------------------------------------------------
648390350_APCC8_010100026493                  ------------------------------------------------------------
640015518_L8106_06195                         ------------------------------------------------------------
648859640_OSCI_3640012                        ------------------------------------------------------------
2508873356_Oscil6407DRAFT_00014730            ------------------------------------------------------------
2504092741_Osc7112_6177                       ------------------------------------------------------------
2506346135_MicvaDRAFT_1862                    ------------------------------------------------------------
638107751_Tery_2688                           ------------------------------------------------------------
2506480833_LYNGBM3L_31450                     ------------------------------------------------------------
647565613_MC7420_2957                         ------------------------------------------------------------
2510102323_Gei7105DRAFT_3578                  ------------------------------------------------------------
2509498590_Pro9006DRAFT_0077                  ------------------------------------------------------------
2509773034_Lepto7104DRAFT_0253                ------------------------------------------------------------
2517693907_LEP6406DRAFT_3365                  ------------------------------------------------------------
2509844778_Lepto7375DRAFT_4795                ------------------------------------------------------------
641676481_cce_1994                            ------------------------------------------------------------
2507502153_Cy51472_3013                       ------------------------------------------------------------
640626984_CY0110_27525                        ------------------------------------------------------------
2503335332_CWat_WH8501_draft2_00062360        ------------------------------------------------------------
2531849533_CWATWH0003_2441                    ------------------------------------------------------------
643475556_PCC8801_2514                        ------------------------------------------------------------
644981523_Cyan8802_3592                       ------------------------------------------------------------
2509576278_Ple7327_4396                       ------------------------------------------------------------
641537721_MAE_37160                           ------------------------------------------------------------
648188704_Cyan7822_3790                       ------------------------------------------------------------
2503801406_Sta7437_3306                       ------------------------------------------------------------
2505785267_Chr6712_1514                       ------------------------------------------------------------
2506598905_Spi9445_1800                       ------------------------------------------------------------
2506609325_Spi6313_1684                       ------------------------------------------------------------
2503367585_Cyast_2162                         ------------------------------------------------------------
gi|16124419|ref|NP_418983.1|                  ------------------------------------------------------------
```

```
gi|16264834|ref|NP_437626.1|              ------------------------------------------------------------
2508652600_Xen7305DRAFT_00047000          ------------------------------------------------------------
2506480821_LYNGBM3L_31350                 ------------------------------------------------------------
2509432410_Mic7113_0575                   ------------------------------------------------------------
2507482189_Cal7103DRAFT_00090960          ------------------------------------------------------------
2509801296_LepboDRAFT_0638                ------------------------------------------------------------
2505802696_Cal7507_4407                   ------------------------------------------------------------
2507332342_Tol9009DRAFT_00023250          ------------------------------------------------------------
2510087202_Riv7116_2056                   ------------------------------------------------------------
2510089901_Riv7116_4755                   ------------------------------------------------------------
2506745681_Syn7336_0399                   ------------------------------------------------------------
637457826_glr0454                         ------------------------------------------------------------
637459188_gll1796                         ------------------------------------------------------------
637461130_gll3716                         ------------------------------------------------------------
2503610992_Chro_0741                      ------------------------------------------------------------
2503793842_Glo7428_1287                   ------------------------------------------------------------
2505800320_Cal7507_2052                   ------------------------------------------------------------
643588131_Cyan7425_5174                   ------------------------------------------------------------
643167924_AmaxDRAFT_0301                  ------------------------------------------------------------
648389440_APCC8_010100021805              ------------------------------------------------------------
646130866_AplaP_010100015993              ------------------------------------------------------------
650384994_NIES39_D01080                   ------------------------------------------------------------
640015631_L8106_17697                     ------------------------------------------------------------
647567948_MC7420_2316                     ------------------------------------------------------------
638106387_Tery_1165                       ------------------------------------------------------------
648857672_OSCI_2870002                    ------------------------------------------------------------
2508875395_Oscil6407DRAFT_00035160        ------------------------------------------------------------
2509801138_LepboDRAFT_0480                ------------------------------------------------------------
2512979571_Fis9431DRAFT_3900              ------------------------------------------------------------
2517061293_PCC9339DRAFT_02148             ------------------------------------------------------------
2505768399_FJSC11DRAFT_3149               ------------------------------------------------------------
2509782632_Mic7126DRAFT_3383              ------------------------------------------------------------
2504683355_Cri9333_0934                   ------------------------------------------------------------
2503606101_GEI7407_0138                   ------------------------------------------------------------
2509502622_Pro9006DRAFT_4121              ------------------------------------------------------------
2509778293_Lepto7104DRAFT_5514            ------------------------------------------------------------
2517694920_LEP6406DRAFT_4378              ------------------------------------------------------------
647577822_S7335_3227                      ------------------------------------------------------------
2503637469_PCC7418_2732                   ------------------------------------------------------------
2509552467_Dacsa_0081                     ------------------------------------------------------------
2503367528_Cyast_2106                     ------------------------------------------------------------
2503887475_Lepto7376_1861                 ------------------------------------------------------------
637688026_PMN2A_1153                      ------------------------------------------------------------
640085320_NATL1_20271                     ------------------------------------------------------------
641286083_P9211_17011                     ------------------------------------------------------------
640547039_SynRCC307_2084                  ------------------------------------------------------------
637798215_PMT9312_1673                    ------------------------------------------------------------
640161373_P9301_17721                     ------------------------------------------------------------
640944474_P9215_18531                     ------------------------------------------------------------
647672827_P9202_63                        ------------------------------------------------------------
640079518_A9601_17881                     ------------------------------------------------------------
637450610_PMM1581                         ------------------------------------------------------------
640081464_P9515_17681                     ------------------------------------------------------------
646569576_Ava_4074                        ------------------------------------------------------------
2505803290_Cal7507_4972                   ------------------------------------------------------------
2517700475_SYN7509DRAFT_4594              ------------------------------------------------------------
2506747473_Syn7336_2171                   ------------------------------------------------------------
gi|1172092|gb|AAA86371.1|                 ------------------------------------------------------------
gi|15597431|ref|NP_250925.1|              ------------------------------------------------------------
637450246_PMM1225                         ------------------------------------------------------------
gi|53721457|ref|YP_110442.1|              ------------------------------------------------------------
638961258_RS9917_04770                    ------------------------------------------------------------
639888778_RS9916_38577                    ------------------------------------------------------------
637444523_SYNW0419                        ------------------------------------------------------------
640545089_SynRCC307_0169                  ------------------------------------------------------------
640084152_NATL1_08771                     ------------------------------------------------------------
647672433_P9202_965                       ------------------------------------------------------------
637797888_PMT9312_1355                    ------------------------------------------------------------
640944066_P9215_14561                     ------------------------------------------------------------
gi|397405|emb|CAA52655.1|                 ------------------------------------------------------------
gi|455023|gb|AAB51624.1|                  ------------------------------------------------------------
gi|1107924|emb|CAA62141.1|                ------------------------------------------------------------
gi|53720415|ref|YP_109401.1|              ------------------------------------------------------------
gi|45297|emb|CAA38731.1|                  ------------------------------------------------------------

2505798636_Cal7507_0387                   -------PGMNNLSVMTAG------PI-LPDPTKLLSSEKMKQLMAYFH--QSF---DLV
2509783408_Mic7126DRAFT_4160              -------PGTSGLSVITAG------PI-LPDPARLLASEKMKQLMAHFH--QNF---DLV
648052066_Aazo_4925                       -------PEMSSLSVITAG------SV-PPDPARLLASDKMKQLMEYFN--ENF---DLV
2504133443_Anacy_3769                     -------PAMNSLSVITAG------PV-PPDPARLLSSDKMKQLMEYFN--QNF---DLV
2506494313_Ana7108_4449                   -------PSMDLLSVITAG------PT-PPDPARLLSSDKMKQLMEYFK--KNF---DLV
2512980750_Fis9431DRAFT_5079              -------PFMKQLSILTSG------PI-PPDPTKLLSSEKMKRLMEDFQ--NTY---DLV
2517062414_PCC9339DRAFT_03271             -------PFMKQLSILTSG------PI-PPDPTKLLSSEKMKRLMEDFQ--NTY---DLV
2505767894_FJSC11DRAFT_3537               -------PFMKQLSILTSG------PI-PPDPTKLLSSEKMKRLMEDFQ--NSY---DLV
2516145585_FIS9605DRAFT_02588             -------SFMSQLSILTAG------PI-PPDPTKLLSSEKVKRLMEDFH--QTY---DLV
2504096080_Cal6303_3081                   -------LWMKQLSVITCG------PI-PPDPAKLLSSQKMRLLMEEFH--KNF---DLV
2507333046_Tol9009DRAFT_00030320          -------PSMNKLSVITSG------PI-PPDPTKLLSSQKMKRLMEEFH--NRF---DLV
2507476338_Cal7103DRAFT_00032380          -------PQIKELSIITSG------PT-PPDPIKLLSSEKIKRLMAEFQ--NTF---DLV
```

```
2505802134_Cal7507_3852                 -------PSMNQLSVITAG------AI-PLDPTKLLSSDKMKQLMADFH--DTF---DLV
2509782674_Mic7126DRAFT_3425            -------PTLNQLSVITAG------AI-TSEPIKLLASSNMKQIMADFH--DNF---DLV
2509771979_CylstDRAFT_5939              -------PSMNQLSVITAG------PI-PPDPTKLLSSEKMKRLIADFQ--NTF---DLV
2503738747_Nos7107_0132                 -------STLSQLSVITSG------PV-PPDATKLLSSEKMKRLMTEFH--NSF---DLV
2509809495_Nos7524_1926                 -------PAMNTLSVITAG------PI-PPDATKLLSSEKMKRLMTDFY--NTF---DLV
640026730_N9414_07896                   -------PEMDKLSIITSGPL-------PPDCTKLLSSEKMKQLMREFY--NEF---DLV
2517239228_Mas10914DRAFT_0471           -------PSMRELSIITAG------PI-PPDPTKLLSSQKMKQLMADFH--QAF---DLV
2510087477_Riv7116_2331                 -------SSMAGLSVITAG------PI-PPDPIKLLSSQKMRLITEELH--SQF---DLV
2510087323_Riv7116_2177                 -------PDLYGCSVITSG------AI-PPDATRLLSSAKMKQLVAEFD--QKF---DLV
2507477177_Cal7103DRAFT_00040780        -------PSVSNLSVLTAG------PL-PPDPAKLLSSEKMKRIMANLH--QKF---DLV
637235631_all5222                       -------PSMEQLSLITAG------PV-PEDPIKLLASEKMRQLMVDFE--NNY---DLV
2506482271_LYNGBM3L_49700               ---------VAGLSVLTSG------QL-VPDPTKLIYSHKMTQLMAGFH--ETF---DLV
641611510_SYNPCC7002_A1500              -LV--------DFSVLPAG------DL-PPDPAKLLASQKMQHLVKDFE--RTF---DLV
2509842234_Lepto7375DRAFT_2251          -FL--------DFYAITAG------TA-PPDPVKLLASKRMRLLMNQLE--KQF---DLV
647107641_CRC_03354                     -------PDLDTLSVITSG------TQ-PPDPMRLISSEKMKQMMAYFR--EKF---DLV
647110196_CRD_02439                     -------PDLPTLSVITSG------ST-PPDPMRLISSEKMKQIMTSLH--KNF---DLV
2509804452_LepboDRAFT_3794              GNVDRAAVEPTNFSVLVGG------KI-PLDPTRLLSSQKMQRLNDYFK--AMY---DLV
647567980_MC7420_2223                   ------SPLEENLFVLTAG------QI-PPDPTRLLCSQKMQHLVESFE--EMF---DLV
2509437593_Mic7113_5758                 -GSTALTPTDDNLFVLTAG------QI-PPDPTRLLASKKMRHLVERFQ--EQF---DLV
2506479760_LYNGBM3L_56010               ------SPLEENLCVLTAG------QI-STDPTRLLNSPKMQNLVQYLK--ERF---ELV
647567242_MC7420_5618                   -------PMWDRLYVLTAG------QV-PPDPSRLLCSKKMQHLMEQFH--AVF---DLV
2509436287_Mic7113_4452                 -------PMWDHLYVLTAG------QL-PPDPTRLLSSKKMLYLMEQFH--AVF---DLV
2503796318_Glo7428_3737                 ------SPLWENLYVLTSG------PI-PPDPVKLLSSKKMQQIMAQLR--QSF---DLV
2517697153_SYN7509DRAFT_1270            ------SPLWENLFAITSG------QI-PPDPIKLLSSKKMQNIMAQLR--TEF---DLV
2503615076_Chro_4796                    ------SPAWENLFVLTAG------QI-PPDPTKLLSSHKMRNIMEQLR--QQY---DLV
2509422734_Oscil6304_3527               ------SALNQNLYVLTAG------QM-PPDPARLLSSQRMKQLIEEFK--TSF---DLV
2506601543_Spi9445_4406                 -SNNGGTGGEDNLYVLTSG------QI-PPDPTRLLSSQKMRSLIDYFR--QSF---DLV
2506609271_Spi6313_1630                 -N-------DDNLSILTSG------QI-PPDPTRLLSSQKMRGLVELFR--DRY---DLI
640014711_L8106_15385                   -------PLWDNLFVLTAG------QI-PPDPTKLLSSNRMRSLMSLF--CSNY---DWV
2509507761_Osc10802DRAFT_1411           ------APHEENLFVLTAG------SV-PPDPLRLLSSKKMHNLMAQWQ--ADY---DLI
2509420889_Oscil6304_1682               ------SPVEPNLFILTAG------AT-PPDPIRLLSSEKMHNLMQQWH--SLF---DLV
2503798162_Sta7437_0103                 -------PHWDNLSVMTAG------DI-PPDPTRLLSSKRMQDLMEQLKNEGQY---DFI
2505785430_Chr6712_1674                 -------DEWEGLSVITAG------DI-PPDPTRLLSSQRMQKLMELLKRSGQY---DFI
2509711143_Pleur7313DRAFT_05131         -------PQWENLSVITAG------DI-PPDPTRLLASQKMYSLMDHLKGIRKY---DLI
2508648278_Xen7305DRAFT_00003760        -------PHWENLSVITAG------DI-PPDPTRLLSSVKMQKLMERLKCDRNY---DLI
2508646941_GLO73106DRAFT_00031960       -------PHWENISVITAG------DI-PPDPTRLLSSQRMQEVMTELENCGDF---DLV
2509573356_Ple7327_1473                 -------PQWENLFVMTAG------DI-PPDPLRLLASKRMKQVIEELHHKSTF---DLI
641537293_MAE_32940                     -------PRHENLSILTAG------SI-PPDPTRLLSSQRMQEMISQLQQDHAF---DLI
643482531_PCC7424_4310                  -------PQWDNLSVLMAG------DI-PSHPARLLGGEKMQDIMIKLEQENDY---DLI
648185689_Cyan7822_0712                 -------PQWENLSVLMAG------DE-AANPTGLLASEKMGQLIEQFHQDSQY---DLV
2509502225_Pro9006DRAFT_3724            -----------NLSVLTAG------QI-PPDPTNLLSSNRMQFLIERFE--ASY---DLV
643584098_Cyan7425_1087                 ------SSLEENLFIMTAG------QI-PPDPTRLLASGRMRELMKELE--ANF---DLV
2503742029_Nos7107_3348                 -----ISPENENLHILTAG------KT-PPNPAQLLSSSKMRDLLVQFQ--KSY---DLV
2504097563_Cal6303_4545                 -------PREENLFIVTAG------QL-TQNSSKLLASEKMREFIERSH--NEF---NLV
2507478127_Cal7103DRAFT_00050290        -------PREDNLYVLTAG------KA-KANPNKLFTSSQMDNFVELSR--THF---DLV
2510089327_Riv7116_4181                 -------HEEENLFVITAG------EI-PRNSAKLFSSPRMRNFVMRSQ--SDF---DLV
2517239184_Mas10914DRAFT_0427           -------SAEENLFVVTAG------ET-PQNPTKLFSSQTMEDFVERTH--TNY---DLV
2507335438_Tol9009DRAFT_00054260        -------PSDDNLFILTGG------RI-PANPTKLLSSLRMQTFIDRTQ--TNY---DLV
2504086624_Osc7112_0134                 ------SPRDENLFVLTSG------PV-PLDPTKVLSSRKMQHLMAALS--SHF---DLV
2506346341_MicvaDRAFT_2066              ------SPRDENLFVLTSG------PV-PLDPTKVLSSRKMPHLMAALS--SHF---DLV
648859632_OSCI_3640004                  ------SPREDNLFVLTSG------PF-FPDPTKLLSSRKMQHLMENLS--SKF---DLI
2508872900_Oscil6407DRAFT_00010170      ------SPREDNLFVLTSG------PF-FPDPTKLLSSRKMQHLMENLS--SKF---DLI
643171775_AmaxDRAFT_4132                ------SPSDENLFILTSG------PL-PVDPTRMLASARMQEVMAQLR--QQF---DLI
648386075_APCC8_010100004460            ------SPSDENLFILTSG------PL-PVDPTRMLASARMQEVMAQLR--QQF---DLI
650384684_NIES39_C03520                 ------SPSDENLFVLTSG------PL-PVDPTRMLASARMQQVMAELR--QQF---DLV
646131598_AplaP_010100019750            ------SPSDE-------------------------------------------------
640018815_L8106_14065                   ------TLSDENLFVLTAG------QP-IADPTRLLSSASMQALMGHLA--EIF---DLV
638107046_Tery_1925                     ------SPVDENLFVLTSG------PI-PSDPTKILSSRRMSQLIQE--SVNTF---DLV
2506483316_LYNGBM3L_63940               -------PLWDHLHVLTAG------SP-AHDPIRLLSAPKMGTLMEHFN--AVF---DLV
648856139_OSCI_1010012                  -------PDEDNLFILTAG------YT-PPDPIKLLSSKKMLYLMEQFQ--AFF---DLV
2508875360_Oscil6407DRAFT_00034810      -------PDEDNLFILTAG------YT-PPDPIKLLSSKKMLYLMEQFQ--AFF---DLV
2509510258_Osc10802DRAFT_3909           -VWSEELPGEDNLFVLTAG------PI-PNDPIKLLSSKKMLYLMEQFQ--AFF---DLV
2509438066_Mic7113_6231                 ---------EENLFVLTAG------QP-TSDSIKLLSSAKMQYLMEQFQ--AFF---DLV
2504090796_Osc7112_4249                 -------PNQENLFVLTAG------QI-PPDPIKLLSSKKMQYLMEQFQ--AFF---DLV
2506345448_MicvaDRAFT_4285              -------PNQENLFVLTAG------QI-PPDPIKLLSSKKMQYLMEQFQ--AFF---DLV
2504091687_Osc7112_5131                 ------LPDDDNLFVLTAG------TI-PSDPIKLLSSRKMHYLMEQFL--ALF---DLV
647568459_MC7420_6195                   -------PLDENCFVLTAG------QS-LSDPIKLISSDKMQYLMEQFS--SQF---DFV
647572642_MC7420_919                    -------PLDDNFFVLTAG------LT-LSDPIKLLASDKMQYLMEQFS--TQF---DLV
640014779_L8106_06284                   ------SPQDDNLFVLTAG------QI-PPDPVQVLSAKKRQYLMEQFR--AFF---DLV
2509421881_Oscil6304_2674               -------PNEDNLFVLTAG------SL-PSDPIKLLSSKKMHSLMEQFQ--DFF---DLV
641253939_AM1_4861                      -------PGTENLFVLTAG------ML-PSDPTRILASQKMQKLMGVFA--EQF---DLV
2514735482_ACCM5_010100007602           -------PGTENLFILTAG------ML-PSDPTRILASQKMQKLMGVFA--EQF---DLV
2510441931_Cha6605_5932                 -------PGETNLFVLLAG------SV-TLDPTSILSSKKMQELVQNCE--HNF---DLV
2503611061_Chro_0810                    ATAHSREISKDNLFILTAG------SI-PPNPSILLSSPQMQSLAEQLQ--QTF---DLV
2503613235_Chro_2969                    LSVSKTSLEVESLYVLTAG------QI-PPNPAHLLSSHKMQNLAKQFE--SAF---DLI
2512979625_Fis9431DRAFT_3954            ------SPIEDNLYVLSAG------PI-PPDSIRLLASQKMQDLMQDLH--SSF---DLV
2517059612_PCC9339DRAFT_00465           ------SPIEDNLYVLSAG------PI-PPDSIRLLASQKMQDLMQDLH--SSF---DLV
2505768848_FJSC11DRAFT_1825             ------SPLEDNLYVLSAG------PV-PPDSIRLLASQKMQDLMQDLH--SSF---DLV
2516149265_FIS9605DRAFT_06272           ------SPLEDNLYVLSAG------PI-PPDSIRLLASQKMQDLMQDLH--SSF---DLV
2517241827_Mas10914DRAFT_3070           ------SPLEDNLFVLTAG------PI-PPDSIRLLASQKMQDLMNELQ--ASF---DLV
2507335136_Tol9009DRAFT_00051240        ------SHDEENLFVMSAG------PI-PPDSVRLLASGKMQDLMDDLQ--ASF---DLV
2504095135_Cal6303_2139                 ------SPIEDNLYVLSAG------PI-PPDSVRLLASQKMQDLMNDLQ--TSF---DLV
2507476798_Cal7103DRAFT_00036990        ------SPIEDNLYVLSAG------LI-PPDPIRLLASQKMQDLMSDLQ--SSF---DFV
2510085606_Riv7116_0460                 ------SPLEDNLYVMAAG------PI-PPDSTRLLASVKMQDLMSELH--SSF---DLV
2503739631_Nos7107_1000                 ------SPIEENLFVMTAG------PT-PPDSVRLLASKKMQDLMNELQ--SSF---DLV
2505801704_Cal7507_3428                 ------SPLEENLFLMSAG------PI-PPDSVRLLVSHKMRDLMNAMQ--AAF---DLV
2505804241_Cal7507_5918                 ------SRQEENLYIISAG------QV-PPDPIRLLASQKMQDLMNELQ--TSF---DLV
643168344_AmaxDRAFT_0719                ------YFLESNLFVLTSG------ST-PPDPIRILSSHRMINLIRKLE--QDF---DLV
```

```
648390241_APCC8_010100025948             ------YFLESNLFVLTSG------ST-PPDPIRILSSHRMINLIRKLE--QDF---DLV
646129996_AplaP_010100011596             ------YFLESNLFVLTSG------ST-PPDPIRILSSNRMVNLIRKLE--HDF---DLV
650383923_NIES39_A06230                  ------YFLESNLFVLTSG------ST-PPDPIRILSSNRMVNLIRKLE--HDF---DLV
640017922_L8106_10512                    ------SPQENNLFVLTAG------SI-PPDPIRVLASQKMLDLMSKLR--SAF---DLI
2510101197_Gei7105DRAFT_2452             ------SSLETNLFVLPAG------PI-PIDPPRLLASQKMQVLMEQLQ--SAF---ELV
2509573281_Ple7327_1398                  ------SPLEDNLYILTSG------SI-PADPTRFLASVKMQELMERLQ--AAF---DLV
2506597130_Spi9445_0041                  ------SPLEENLSILTTG------SM-PPDPIRLIASQKMEDLMHQLS--EAF---DLV
2503636620_PCC7418_1894                  ------SPLEENLFFLGSG------QI-PPDPSKLIASEKMQRLMIQLQ--SEF---DLV
2506607995_Spi6313_0371                  ------SVLDENLFVLTAG------ML-PPDPIRLISSQKMQELMRSLR--EQF---DLV
643585148_Cyan7425_2153                  -------SEESFLYVLTPG------QL-PPDPVNLLSSERMAVLMQQFE--NAF---DLV
2504681848_Pse7367_3384                  ------SSLEENLYVLAAG------TANSPDPTRLLASQKMEALMAYLE--SKF---DLV
647577962_S7335_3828                     DESFVNLAVDENLFFMSSGSIAV-------DPAKVLASKKIEEFFQQIY--KTF---DVI
2509846600_Lepto7375DRAFT_6617           ----------DGLKVLCAG-------TKGHDAGHILTTKRVQGLLQLK--TKF---DLV
2509774530_Lepto7104DRAFT_1750           ------SPSEPNLFVLTTGTTEI-------DPARLLSSVKMTQFVAKTE--SYF---DLV
2509804417_LepboDRAFT_3759               ------SAMEQNLFVLTAG------KA-QMDASRLLSSSKMKTLMEGLK--EEF---DLI
2531850144_CWATWH0003_3517               -----ISSLDQNLHVIAGKPHGRNAPV-------LLRSEKFSQLMEQWK--EKF---DLV
2509777916_Lepto7104DRAFT_5137           -------PGNPNLHLLTAG-------ARPPAPGRLLSSRKMQQITEQYR--RHY---DLI
648856194_OSCI_1040007                   ------SPLADNMFVLTAG------IPLPG-AARRMSSSFMAHLMEEFQ--TKF---DLV
2508875344_Oscil6407DRAFT_00034650       ------SPLADNMFVLTAG------IPLPG-AARRMSSSFMAHLMEEFQ--TKF---DLV
2504087727_Osc7112_1201                  ------SHLADNLFVLTAG------LPGSG-TARRLASSQMQYLMEELQ--AQF---DLV
2506348042_MicvaDRAFT_3627               ------SHLADNLFVLTAG------LPEPG-TARRLASSQMQYLMEELQ--AKF---DLV
2509438406_Mic7113_6573                  ------SAI-DNLFVLTTG------QLQPN-STKLLGSAQMQYLMEEFQ--ATF---DLV
647571294_MC7420_5066                    ------SPISDNLFVLTAG------QPRPH-LTRRFASAQMQELMAEFK--TKF---DLV
2506481927_LYNGBM3L_44990                ------SPFSDNLFVLTSG------QALVE-SAPDLASPRMEKIMGHFQ--EKF---DLV
640019302_L8106_03117                    ------SAMADNLFALTAG------TPTAG-AIKLLGSAQMRHIVDELQ--KTF---DLV
2509707887_Pleur7313DRAFT_01875          ------SSHRDNLFILASG------QIPAD-SSKLLSSNQMQQLIEEFR--QSF---DLI
641251898_AM1_2808                       ----------------------------------------KYL-------SKI---NYI
2514737774_ACCM5_010100019113            ------VPYQKNLSLLTAG------QTGSK-ANKSSASNQMKYLMEQLQ--STF---DLV
641251897_AM1_2807                       ------VPYQKNLSLLTAG------QTGSK-ANKSSASNQMKYLMEQLQ--STF---DLV
2512634931_Chl7702DRAFT_3806             ------SPLQKNLFVLTSG------KILPS-SPRMLGSNQMQHFMEKFQ--QAF---DLV
2516146228_FIS9605DRAFT_03233            ------SRIQNNLFVLTSG------KILPS-STRMLGSTQMQHVMEQFH--QAF---DLV
641675948_cce_1468                       ------VRDIENLFVLPMG------EHLQP-SLKHLWSPKFNSLMEELG--KTY---DLV
2507501641_Cy51472_2500                  ------VRDIENLFVLPMG------EHLQP-SLKHLWSPKFNSLMEELG--KTY---DLV
640626055_CY0110_29394                   ------VRDIDNLSVLPMG------ENLKP-SRKYLWSPKFNSLIEELG--KTY---DLV
2531850936_CWATWH0003_4939               ------APDIENLSVLPIG------GGSEP-SVKHIWSPKFNGLMEELG--KTY---DLV
643476430_PCC8801_3415                   ------VPNTENLFILTTG------KATPH-PPKRLWSARMQYLIEELP--MLY---DLV
644980660_Cyan8802_2701                  ------VPNTENLFILTTG------KATPH-PPKRLWSARMQYLIEELP--MLY---DLV
643478515_PCC7424_0240                   ------SQEIDNLFVLSAG------SAQAP-ASKLLWFPRMQSLMRELK--VRY---DLI
648186851_Cyan7822_1903                  ------SKEVENLYLLSAG------CAESP-STKLLWFPRMRSLMREFK--VRY---DLI
2509575480_Ple7327_3597                  ------VPDTENLFVLTAG------ISHPD-PSMRVWSSRMQNLMEELH--SKY---DLV
2508645044_GLO73106DRAFT_00012970        ---------NPNLSVLPGGNTSELNQIN-------LSSNQIKYLMDSLAK--KY---DLV
2509773763_Lepto7104DRAFT_0983           ---------ESNLFAISSG-------TESMDPTRLLSSVRMKDLMARME--KNF---DLV
2503745907_Cyan10605_1646                -------SQWDNLSIITVG------EVL-SDPSSLLYSQAMSTMMQQIQASQQF---DLI
2509874395_Syn6308DRAFT_1102             -------PSWDNLSVITAG------NVS-DDPTKLLYSQKMQEIVKLLNNCQTF---DLI
644979531_Cyan8802_1534                  ------------------------------------------------------------
2503745344_Cyan10605_1086                --------D-DGLSVITAGSK-------PPNPTRLIASRKWQTLMQKFK--QDF---DFV
2509873750_Syn6308DRAFT_0457             --------KLDNLFLLTAGSQ-------INNPVGILSSPKMANLTKTFK--QDF---DFV
2505785711_Chr6712_1954                  ------SRLDENLFILSSG--KINDD---LDSSKLLASPKMRSLMEELE--QNF---DLV
2509706287_Pleur7313DRAFT_00275          ------IPSEENLFILTSGFNALDTDSTKLDPSRLLVSGKMHHLMEEIK--THF---DLV
640027738_N9414_07903                    ------------------------------------------------------------
2506482929_LYNGBM3L_58920                LGDAIKPV-IETLDVLTSGVVP-----PN--PAVLLDSQRMASLINHVAS--RY---DFV
637461200_glr3785                        WSNCVNQID-ENVHVLTSGPLP-----PD--PVALLDSLRMKELVKQWGE--RY---DLV
2503887239_Lepto7376_1629                --------EDRELFVLSPG------KS-EDEIAKLLNNERLDSVMAKLE--QEF---DLI
638113739_sync_0150                      -------PGYENWSVMTAGLR-------PPDPTRLLSSQRMHDLVQEITQSNDF---DLV
638963744_RS9917_02426                   -------PGYDQWSLITAGRR-------PPDPTRLLSSQRMHSLVQELANGGEF---DLV
2507493170_Syn8016_1895                  -------NGYEGWSVMTAGRR-------PPDPTRLLSSKRMHQLVEDLGKSEEF---DLI
638960084_WH5701_13945                   -------PDHPNWQVLTAGRR-------VPDPPRLLSSERMGHLVKEISSSGAY---DLI
650128418_SCB01_010100004274             -------EA--NLALLPVGRR-------PPDAAKLLSSARCGEVIQQLRALPQF---DLV
650132168_SCB02_010100007823             -------ES--NLAVLPVGRR-------PPDAVKLLNSSRSQEVIQTLREMPGF---DVV
2508551611_Cyagr_1223                    -------A--ENMDVLPAGPK-------PPDPAKLLNSSRCKEIMEEIRALPGY---DII
640081072_P9515_13861                    -------PGFKNWDVITSGTK-------PPDPTRLLNSEKMRTFIKDIKESDKY---DLI
648050855_Aazo_3125                      --GLQYSG-SSYIDILTAG------PIPV-DPAHLLSSPRMMELMAAFE--ENY---DLV
2504129904_Anacy_0284                    --GVQYSG-SSYIDILTAG------PIPI-DPAHLLSSPRMMQLMAAFE--ENY---DLV
2506490031_Ana7108_0216                  --GVQYSG-SSYIDILTAG------PIPL-DSANLLSSPRMIQLMAAFE--ENY---DLV
2509770736_CylstDRAFT_4696               --GVPYSG-SSYIDILTAG------PTPT-DPANLLSSPRMMQLMAAFE--DNY---DLV
642600344_Npun_R1070                     --SLQYAG-SAYIDILTAG------PKPA-DPANLLSSPRMMQLMAAFE--ENY---DLV
2505799741_Cal7507_1481                  --SIQYSG-SSYIDIMTAG------PIPA-DAANLLSSPRMMQLMAAFE--ENY---DLV
2509779661_Mic7126DRAFT_0411             --SIQYSG-SSYIDIMTAG------PIPA-DAANLLSSPRMMQLMATFE--ENY---DLV
2503739922_Nos7107_1290                  --GIQSLG-SAYIDILTAG------PKPT-DPAHLLSSPRMIQLMRTFE--ENY---DLV
640027761_N9414_00965                    --GVPSLG-SAYIDILTAG------PTPI-DPANLLSSPRMMQLMAVFE--DNY---DLV
637233217_alr2833                        --GIHNVG-SAYIDILTAG------PSPS-DSANLLSSPRMKQLMAAFE--ENY---DLV
646566603_Ava_1116                       --GIHNVG-SAYIDILTAG------PSPS-DSANLLSSPRMKQLMAAFE--ENY---DLV
2509808002_Nos7524_0433                  --GTHSVG-STYIDILTAG------PRPT-DPANLLSSPRMKELMNAFE--ENY---DLV
2517241466_Mas10914DRAFT_2709            --SIQSSG-SSYIDILTAG------PTPA-DPANLLSSPRMQQLMATFE--ENY---DLV
647105946_CRC_01573                      --GISYSG-SAYIDILTAG------PIPA-DPAHLLSSPRMREIITMFE--ENY---DLV
647109494_CRD_02102                      --GISYSG-SAYIDILTAG------PIPA-DPAHLLSSPRMREIITMFE--ENY---DLV
2507332201_Tol9009DRAFT_00021840         --SIQSSG-SSYIDILTAG------PSPG-DPANLLSSPRMMQLMAAFE--ENY---DLV
2512975951_Fis9431DRAFT_0279             --SIPSAG-SSYIDILTAG------PAPA-DPANLLSSPRMEELMAAFE--ESY---DLV
2517062640_PCC9339DRAFT_03498            --SIPSAG-SSYIDILTAG------PAPA-DPANLLSSPRMEELMAAFE--ENY---DLV
2505767181_FJSC11DRAFT_4188              --SIPSAG-SSYIDILTAG------PAPA-DPANLLSSPRMEELMAAFE--ENY---DLV
2516146836_FIS9605DRAFT_03842            --SIPSSG-SSYIDILTAG------PTPA-DPANLLSSPRMEELMAAFE--ENY---DLV
2512634275_Chl7702DRAFT_3149             --NLQSSG-SSYIDILTAG------PIPA-DPAHLLSSPRMQELMRAFE--ENY---DLV
2510089385_Riv7116_4239                  --TLQSSG-SSYIDILTAG------PVPE-DPANLLSSPRMEELMSAFE--ENY---DLV
2504094820_Cal6303_1825                  --GVQSSG-SSYIDILTAG------PEPL-DPANLLSSPRMEQLMATFE--GNY---DLV
2507482515_Cal7103DRAFT_00094220         --SIQSSG-SSYIDILTAG------PAPA-DPAHLLSSPRMKQLMAAFE--ANY---DLV
2503612562_Chro_2300                     --AIQSSG-S-YIDILTAG------PQPE-DPANLLSSQRMRELMAGFE--QNY---DLI
2517695945_SYN7509DRAFT_0062             --GIQSSG-A-YIDILTAG------PVPT-DSASLLSSQRMRELMAKFE--DTY---DLI
2503608186_GEI7407_2184                  --DIQPSGHDSDISVLTAG------PTPT-DPAKLLSSRRMAELMAMFE--QTY---DLV
```

```
2504685208_Cri9333_2761              --HIPVL--DIHIDILTSG------PIPT-DPVNLLSSPQMKKLITAFE--QSY---DLV
2509431928_Mic7113_0093              --SLHAS--SAYIDVITSG------PISP-DPANLLSSPRMGELMAEFE--QSY---DLV
2506479159_LYNGBM3L_28310            --SISPLG--STIDVLTAG------PTPI-DPVKLLGSKRMKNLMAEFQ--QTY---DLV
640017638_L8106_27951                -NQISALGLS--IDVLTGG------SITA-DPVKLLSSHKMRQLMTIFE--RNY---DLI
2509806250_LepboDRAFT_5593           ----ASTYSDLPISVLTAG------SSPS-DSVKLLSSKRMRDLMTLFE--QHY---DLV
2504583836_Pse7429DRAFT_1713         ---IQSS--NSAIDILTGG------PIPS-DSVTLLSSEWMQNLISKFE--QEY---DLV
641251010_AM1_1910                   PGTLPPSDLS--IDVLTAG------PLSA-DPVKLLSLERMQDVLKAFE--NNY---DLI
2514735759_ACCM5_010100008979        PGTLPPSDLS--IDVLTAG------PLSA-DPVKLLSLERMQDVLKAFE--NNY---DLI
2509847582_Lepto7375DRAFT_7599       ISPVSLSHSGTSVDVLLAG------AVP-EDPMRLLSSQQMRQVLEKAE--ANY---DLV
648051793_Aazo_4516                  ---IQPIHPS--IDVLTAG------PTP-DDVVNLLTSGRMKELIESFE--KIY---DLV
2504134691_Anacy_5006                ---IQPIHPS--IDILTAG------PTP-EDVVNLLSSEKLKELIESCE--QMY---DLV
2506494605_Ana7108_4733              ---VQPIHPS--IDILTAG------PTP-DDVVNLLSSARMKELIQSFE--QIY---DLV
647107726_CRC_03451                  ---VQPIHPL--IDVLTAG------PQP-DNAVSLLTSGKFQELLEVLD--GIY---DLV
642604187_Npun_R5250                 ---IQPIHPS--IDILTAG------PEP-EDTVKLLSSQRMKELIELFE--QSY---DLV
2509767727_CylstDRAFT_1687           ---IQPIHPY--IDILTAG------PTP-DDAVNLLSSQRMKELIELFE--QTY---DLV
2507335141_Tol9009DRAFT_00051290     ---IQPIHPA--IDILTAG------PTP-EDTVKLLSSRRMKELIQVFE--QTY---DLV
640028130_N9414_07219                ---IQPIHPS--IDILTAG------PTA-EDTINLLSSQRMQELIELFE--QTY---DLV
637230414_all0059                    ---IQPIHPS--IDILTAG------PTP-EDAVELLSSQRLKDLIELFE--ETY---DLV
646568157_Ava_2661                   ---IQPIHPS--IDILTAG------PTP-EDAVELLSSQRLKDLIELFE--ETY---DLV
2509811005_Nos7524_3436              ---IQPIHPS--IDILTAG------PVP-EDPVKLLSSHRLKELIEIFE--QSY---DLV
2503739635_Nos7107_1004              ---IQPVHPL--IDVLTTG------PQP-EDTIRLLSSQRMKELIKQFE--QTY---DLV
2509781657_Mic7126DRAFT_2408         ---IQPIHPS--IDVLTAG------PFP-EDTVTLFSSQRMKQLIAYFG--QNY---DLV
2517243237_Mas10914DRAFT_4480        ---IQPIHPS--IDVLTAG------PTP-EDTVKLLTSARMKELLKFFE--QTY---DVV
2512634393_Chl7702DRAFT_3267         ---IQPIHPS--IDVLTAG-------SAKEDTVKLLSSQRMKELLKTFE--CTY---DLV
2505769181_FJSC11DRAFT_2156          ---IQPVHPS--IDVLTAG-------SPIEDAVRLLSSGRMKELLESFE--QTY---DLV
2512977368_Fis9431DRAFT_1696         ---IQPVHPS--IDVLTAG-------SPAEDPVKLLSSGRMKELLESFE--QTY---DLV
2517063601_PCC9339DRAFT_04460        ---IQPVHPS--IDVLTAG-------SPAEDPVKLLSSGRMKELLESFE--QTY---DLV
2516147062_FIS9605DRAFT_04068        ---IQPIHPS--IDVLTAG------STP-EDTVKLLSSQRMKELLESFE--QTY---DLV
2507482923_Cal7103DRAFT_00098300     ---IQPIHPS--IDVLTAG------PPELEDTVKLLSSRRMKELLKEFE--QNY---DLI
2510085595_Riv7116_0449              ---VQPVHPA--IDVLTAG------PIP-DDTVNLLSSGRMKELLEIFT--RSY---DLV
2503614917_Chro_4637                 ---IQSVQPS--LDVLTAG------PTP-TDIVKLLSSHRMKHLIESFE--QSY---DLV
2504093446_Cal6303_0467              ---IQPIHPS--IDVLTAG------VMP-EDSLKLLSSNRMKELLEYFE--QIY---DVV
647578236_S7335_5395                 LIPVSTTLLGTSIDVLPA-------TLEAHDSIKILSSPRFRVLIEALE--NRY---DLV
2503798844_Sta7437_0777              ----QVTLAGENIDLVTCG------SRIT-DPVKFLSSPKFKQSIDQLQ--ANY---DLI
2509708827_Pleur7313DRAFT_02815      ----QVSFSGETIDLIPSG------SIPL-DPVKLLNSSILLDLIDQQK--QNY---DLI
637009749_sll0923                    ------EPLGKNLFYFKV-------QDDTMTPEQLVSASQ-NFVVKMNQWKETF---DLI
2503800689_Sta7437_2597              ------------LHLITAGST-------SPNPIALLNSDKMKQLMQQWQ--EAY---DYV
2509710958_Pleur7313DRAFT_04946      ------------LHLITAGAT-------SPNPIALLNSDKMKQLIQAWE--AAY---DYV
2505786361_Chr6712_2601              ------------LDVMTAGSN-------VPNPIALLKSQKMSQLIQEWE--AAY---DYV
2508650693_Xen7305DRAFT_00027920     ------------LDVITAGAN-------VANPIALLRSQIMSELLKEWE--AAY---DYV
2506480412_LYNGBM3L_19610            ------------LDFISSGPT-------PANPVALLNSQKMKQLIDEWR--QTY---DYV
2509512444_Osc10802DRAFT_6095        ------------LDVITSGPT-------PPNPVALLDSAKMTQLLSEWR--QAY---DYV
643587900_Cyan7425_4938              ------------LDILTAGFR-------PPNPIALLDSYKMVGLIDEWR--QHY---DYI
2504582150_Pse7429DRAFT_3127         ------------LHILTSGSI-------PPNPMLLLESVKMTALVQEWR--QEY---DYV
2504679882_Pse7367_1451              ------------LNVITSGPL-------PPNPMVLLESTKMTKLLQLWR--QTY---DYV
2507088905_Pse6802_3551              ------------LDVLTSGPI-------PPNPLVLLESVKMTNLMQAWE--DEY---DYV
2508687870_Syn7502_01070             ------------LHIITAGAT-------PPNPMILLESNKFQKLLGEWD--QTY---DYV
2506748948_Syn7336_3628              -----------HLDVLPAGPR-------VRNPLVWFLSQRMSTLLASWQ--EVY---DYI
643484053_PCC7424_5769               LEQRYQKLVLSQPDILTSGPV-------PATPLAWLASEKMMQMLDQWR--KAY---NYV
648199484_Cyan7822_5718              LIEKY-------PDVLTSGPV-------GKNSFSWLVAPKMNELLNSWR--QAY---DYV
2510436294_Cha6605_0295              -KDLIKVSPESQLHVLFSGK-------QTVSSIVLFNSPRLIRLMEEWRK--EY---DYV
637314397_tll1767                    ---------QRGLAILTAGEL-------QQQPMRLLSQDTLKPFMAHLKNY--F---HLV
637313820_tll1199                    ---------TEGLWVLTGGQA-------QQEPMALFSSYRWFDFLDSTK--ETF---DVV
2512980079_Fis9431DRAFT_4408         IETVVHEV-MPNLHVLTSGVMP-----PN--PVALLDSERMASLVANFTK--DY---DCV
2517061743_PCC9339DRAFT_02599        LETVVHEV-MPNLHVLTSGVIP-----PN--PVALLDSDRMATLVANFTE--DY---DCV
2505770929_FJSC11DRAFT_1293          LETIVHEV-MPNLHVLTSGVIP-----PN--PVALLDSDRMATLVASFTQ--KY---DCV
2516148164_FIS9605DRAFT_05171        LDSVVQEV-MPNLYVLTSGVRP-----PN--PVALLDSQRMAALVTTFTR--DY---DCV
2512634416_Chl7702DRAFT_3290         LSTIVEEV-IPNLYVLTSGVIP-----PN--PLALLDSQRMAALVTVFAR--NY---DCV
2505800360_Cal7507_2092              LNKAVQEF-IPNLYVLTSGVIP-----PN--SIALLGSKRMDSLVKIFAK--EY---DFV
2509780096_Mic7126DRAFT_0847         LGIAVQEV-IPNLYVLPSGVVP-----PN--TVALLGSKKMEALVTIFSK--EY---DFV
2505800523_Cal7507_2254              LDVAIQEV-MPNLYVLPSGVLP-----PN--PVALLDSQRMAALVGSFAK--HY---DFV
2507478259_Cal7103DRAFT_00051610     SDKVIQEV-MPNLFVLPSGVLP-----PN--PLALLDSMRMSALVDEFVR--DY---DFV
637234831_all4432                    LDEAVQEV-MPNLEVLPCGILP-----PN--PVAMLDSQRMATLMSNFAR--DY---DFI
646566875_Ava_1386                   LDEAVQEV-MPNLEVLPCGILP-----PN--PVAMLDSQRMATLMSNFAR--DY---DFI
642600743_Npun_R1496                 LDAVVQEV-MPNLEVLTSGILP-----PN--PVALLDSQRMATLISNFSK--DY---DLV
2507334816_Tol9009DRAFT_00048030     LNAAVQEV-LPNLYVLSSGVLP-----PN--PMALLDSQRMAILLNSFAR--DY---DFV
2507476704_Cal7103DRAFT_00036050     LSSAAQKV-LPNLDVLPCGVLP-----PN--PMALLDSQRMTSLVEDFNE--RY---DFV
2510090628_Riv7116_5482              IESAVQEV-APNLDVLSSGVLP-----PN--PMALLDSQRMATLVKQFTH--KY---DFV
2506483552_LYNGBM3L_64290            FDQAKQEV-MTGLDVLMAGVIP-----PN--PVALLDSQKMASLMEKFSQ--EY---DCV
2517697529_SYN7509DRAFT_1646         LKTAIKKV-MPNLSVLPAGAIP-----PN--PMPLLESKRMTSLIDHFSD--SY---DFV
2503608035_GEI7407_2035              LSEAVNPV-MDNLDVLTCGAIP-----PN--PLALLDSQRMQSLVQEFGQ--QY---DFV
2509433692_Mic7113_1857              FGIAVREV-MDKLDVLPSGVIP-----PN--PLALLDSKRMNSLIEEFSG--TY---DFV
640015133_L8106_09871                LNSAIKRI-SYNLDVLPSGVIP-----PN--SLALLESKRMNSLMKDLRA--IY---DFI
2503795055_Glo7428_2485              FESVVTEV-IPKLDVLSAGVIP-----PN--PMALLDSKRMASLVQYFSD--RY---DFV
2509506717_Osc10802DRAFT_0363        FKTAATEV-MPNLDVLTAGVVP-----PN--PLALLDSKRMASLIEYFSE--NY---DFV
2503614965_Chro_4685                 YQSAIKEV-LPKLHILTAGATP-----PN--PVALLDSVQITSLIDNFAK--SF---DFV
2510091904_Riv7116_6758              FETVVSGG-MLSLDILTAGATT-----PN--PLFLLDSRKMETLIEDFKN--TY---DFV
2503612629_Chro_2367                 FQAAVEQV-MPNLHVLTAGVVP-----PN--PMALIDSQRMAALVSSLSD--RY---DFV
2503612419_Chro_2158                 FEVIEKSD-VANLDILTAGVIP-----PK--PIALLDSPRMALLIDYFAA--RY---DFV
2506493946_Ana7108_4087              FEAAVQRG-IDNLDVLTSGDIP-----FN--PLSLVDSKRMVSLIEDFYQ--NY---DFV
2509803997_LepboDRAFT_3339           ARDAIIPV-MQNLDVLTCGVIP-----PN--PLALLDSKRMASLIETFEQ--QY---DFV
2512979777_Fis9431DRAFT_4106         FSEAVHKV-TEFLSVLTAGVIP-----PN--PLALIDSESMTSLVDIFAK--QY---DYV
2517062202_PCC9339DRAFT_03059        FSEAVHKV-TEFLSVLTAGVIP-----PN--PLALIDSEGMTSLVDVFAE--NY---DYV
2505770300_FJSC11DRAFT_0671          FPQAVQKV-TEFLAVLTAGVIP-----PN--PLALIDSEGMTSLVDIFAE--NY---DYV
2507334440_Tol9009DRAFT_00044270     FPQAIRVV-TKYLSVLTAGVQP-----PN--PLALIDSDRMTSLIDKFSQ--SY---DYI
2510088639_Riv7116_3493              LYRCHHQV-TENLSVLTAGVQP-----PN--PSALIDSAGMDSLIQQLLP--SY---DYI
2505803639_Cal7507_5320              FSEAVQSV-TNNLSVLTAGVMP-----PN--PLALIDSERMTSLMEMFAQ--RY---DYV
2509782388_Mic7126DRAFT_3139         FSEAVQSV-TDNLFVLTAGVMP-----PN--PLALIDSERMIALMEMFAQ--KY---DYV
```

2507335607_Tol9009DRAFT_00055950	FSRSVQNI-TDNLSVLTAGVMP-----PN--PLALIDSERMTNFIDMLSK--TY---NYV
2510088989_Riv7116_3843	FESAVQKV-TDNLSVLTSGVMP-----PN--PLALIDSERMTNFIEMLSQ--TY---DCI
2504096650_Cal6303_3648	LNRVVQKV-STNLSLLTAGVIP-----PN--PVALIDSESMMNLIQTMAG--EY---EYI
2507481772_Cal7103DRAFT_00086780	IRSCIQSV-TRNLSVLTAGVIP-----PN--PQALLDSEGMSALIQALSQ--HY---DYI
2509803885_LepboDRAFT_3227	LRQAVKPI-TNNLSILTAGVIP-----PN--PLAILDSEAMTTLLETIAK--DY---DYV
647579433_S7335_2031	AQDTIQSV-MPRLHVLPSGWIS-----SN--PVDLLDASSMERLIAELSEY--Y---DFI
2509846113_Lepto7375DRAFT_6130	LKDVIVSQ-EPNLHILTAGVIP-----FN--PLAVLESQLITSLLQ--ASEKAY---DYI
2512978854_Fis9431DRAFT_3183	SKTAVKTV-MNDLDVMTAGILS-----SS--SVSLLDSQKMAGLIDSFAA--NY---DFV
2516143706_FIS9605DRAFT_00708	VGTAINNV-MNNLDVLTAGVVP-----PS--PASLLDSRKMAGLIESFAA--NY---DFV
642600641_Npun_F1381	ISTVIKTV-MNNLDVLTAGLVR-----PS--PASLLDSKCMDSLIQNFAI--NY---DFV
2509770219_CylstDRAFT_4179	IKTAIKKV-MDNLDVLTSGVVP-----PS--PGSLLDSKRMASLIEIFSA--NY---DFV
2504094091_Cal6303_1107	MWGATTQV-MDNLYVLTSGLVP-----PS--PASLLDSKRMATLIQRFAA--NF---DFV
2507479320_Cal7103DRAFT_00062230	ARTAITRV-MDNLYVLPSGLVP-----PS--PASLLDSKRMATLIDSFRN--NY---DCV
2510085390_Riv7116_0244	VMAAIKKG-MDNLDVMTCGAVP-----PS--PASLLDSKRMASLVESFRS--YY---DYV
642601572_Npun_F2453	--
2503611448_Chro_1193	PKTAIKKI-TANLHILTSGVIP-----PN--PMALLDSQRMASLVTVFSA--NY---DYT
2503796978_Glo7428_4384	FPAVVKQV-IDNIHVLTAGSTP-----PN--PVALLESQRMANLATMFSE--NY---DFT
2504087786_Osc7112_1259	FGDAIAQV-MDNLDVLTCGVIP-----PN--PMALLDSQRLTSLIKQFSA--TY---DAV
643584136_Cyan7425_1127	LPVALNPV-LENLDVLTAGVIP-----PN--PGALLDSKRMATLVQALRE--RY---DFI
647105865_CRC_01492	LENCLQD--VSSVHVLTAGKVP-----PN--PVKLLDSQSMASLIAAARR--DY---DFV
647108284_CRD_00419	LGASIQE--VSSISVLTAGNTP-----PN--ALQLLDSESMVNFVQKVRN--EY---DFI
2505800344_Cal7507_2076	LQSSTKEA-LMAMDLLTAGTIP-----PN--PAALLDSQRLATLIKQATG--EY---DFV
2509781889_Mic7126DRAFT_2640	LQSSTKEA-LMAMDLLTAGTIP-----PN--PAALLDSQRLTTLIKEATG--EY---DFV
2507481279_Cal7103DRAFT_00081850	YVAIAQEA-IHNLDILTAGTIP-----PN--PAALLDSQRMRALIEEASK--DY---DFV
637233240_alr2856	LAEAAKEV-VINLELLTSGTIP-----PN--PAALLDSQRMNALLQQAAK--DY---DCV
646566532_Ava_1045	LAEAAKEV-VINLELLTSGTIP-----PN--PAALLDSQRMNGLLQQAAE--DY---DYV
640028261_N9414_00005	LTQSSQEA-LVNLDLLTAGTIP-----PN--PAALLDSQRM-------------------
642603803_Npun_R4851	FRRTTQEV-LVNLDLLTAGTIP-----PN--PAALLDSQRMALLIQEAAK--DY---DCV
2509770699_CylstDRAFT_4659	FRSTSQEV-LVNLDLLTAGTIP-----PN--PAALLDSQRMALLIQEAAK--DY---DCV
2507478691_Cal7103DRAFT_00055940	LQTASRKI-ANNVYLLTAGATP-----SN--PFATLASPQMNALIATAQQ--EY---DFV
641249527_AM1_0407	WHEAIRPE-DDQLDILTAGVTP-----PN--PVPLIDSHHMAVLLEEFHQA--Y---DYV
2514736728_ACCM5_010100013856	WHESIRPE-DDQLDILTAGVTP-----PN--PVPLIDSHHMAVLLEEFHQA--Y---DYV
643586770_Cyan7425_3796	-TKFIHQE-SDNLDVLTAGPRA-----PN--PLLLLDSQHMASLIDKFRTV--Y---DYV
2509555626_Dacsa_3240	INSVVREL-MPNLDVLTAGAPP-----PN--STALLDSQRMKDVLNQCS--LDY---DYV
2509775597_Lepto7104DRAFT_2817	-QDVTHKV-FENLTLMTAGALP-----PN--PLAILDSERMADMLQQLKR--QY---DYI
2506492920_Ana7108_3067	LDRAVTQI-SPNLEVLTAGELN-----QN--PASLLDSSQMAVFVAQIAQ--KY---DFV
2512631781_Chl7702DRAFT_0655	LERAITEV-APNLQVMTAGERT-----SN--PSALINSSQIAVFIARVAQ--KY---DFV
2504685956_Cri9333_3503	FNDAVVEV-MENLEVLTAGTPP-----PN--PLILIDSSQMAVLVGQSAQ--TY---DFV
2510436704_Cha6605_0705	RKTATTEI-QPNLFLVTAGVVP-----PN--PVVLLDSVQMSTSIAQWSQ--EY---DLV
2507482822_Cal7103DRAFT_00097290	TSMATKEI-MPNLHLLTSGVIP-----PN--PLTLLKSQAMATLINNESR--NY---DYI
2510087184_Riv7116_2038	LSTAIKII-QPNLHVLTSGVTP-----PN--PVTLLKSQAMAELIKHASE--NY---DYV
2506745596_Syn7336_0315	LADALQTV-QPYLDILPAGQLA-----AN--PTSLLQSRSWSALLQQAAE--QY---DFI
2503796286_Glo7428_3705	NKKAIQPV-AKNIDVLTTGTSE-----SD-LSI-LIDSRSMSALITQYAK--QY---DYL
642604427_Npun_F5505	FDVALSKV-MDNLDVLTAGSRP-----PN--PLALLDSKRMASLIESFSSQYKY---DFV
2503612394_Chro_2133	LSVATSKV-MNNLDVLTAGVTP-----PN--PLALLDSQQMAALVEGFSS--RY---DFV
2512632720_Chl7702DRAFT_1594	FPQVTKKG-MDCLDVLTAGSRP-----PN--PLALLDSVAMASLVD--YSCKHY---DYV
2509807906_Nos7524_0337	FNETVSQV-THYLDVLTAGVRP-----PN--PLALLDSKRMALLIQDFSD--QY---DVV
2509770373_CylstDRAFT_4333	FHKIAHPV-MDNLDVLPSGVKP-----PN--PLALLDSKRMASVIADLSS--QY---DFV
646570355_Ava_4846	FITAISKV-MDNLDVLTAGVRP-----PN--PLALLDSKRMAALIADFSS--QY---DFV
2510438837_Cha6605_2838	LQQISWTV-MENLDVLTAGARP-----SN--PLSALESKQMKLLIQEVSK--SY---DFV
2506477923_LYNGBM3L_10200	LELAICKA-MENLDVLTAGVMP-----PN--PLALLDSKQMASLIQECST--QY---DFV
2509574529_Ple7327_2646	SKALKQAV-IPNLDILSSGVIP-----PS--PATILDSQRMTLLLREFTR--KY---DFV
643170532_AmaxDRAFT_2895	LSEAVKPG-IDQLDILTAGVTP-----PN--PLALLDSRRMTSLIRNFSE--EY---DFV
648389308_APCC8_010100021105	LSEAVKPG-IDQLDILTAGVTP-----PN--PLALLDSRRMTSLIRNFSE--EY---DFV
646129623_AplaP_010100009700	LSEAVKPG-IDQLDILTAGVTP-----PN--PLALLDSRRMTSLIRNFSE--EY---DFV
650384826_NIES39_C04940	LSEAVKPG-IDQLDILTAGVTP-----PN--PLALLDSRRMTSLIRNFSE--EY---DFV
2503801641_Sta7437_3540	LSLAVSRP-LEQLDLLTAGEVQ-----SN--PLALLDSEAMSELINQSRH--KY---DLI
2509711085_Pleur7313DRAFT_05073	PKSVIERP-MPKLDILTSGLVR-----TN--PLALIDSQAMSDLVGRSRR--EY---DLI
2503801821_Sta7437_3719	LHEVVARP-IERLDLLTVGVVP-----PN--PLALLDSIEMNELIAQAKK--DY---DFV
2505786500_Chr6712_2739	LREVAIQP-MAKLDLLSSGLIP-----PN--PLALLDSNEMGELVAKARK--EY---DIV
2508650623_Xen7305DRAFT_00027220	LNQITQQP-MPNMDLLTSGEII-----SN--PLSLLDSLEMSELVAKARR--EY---DLI
2509706990_Pleur7313DRAFT_00978	ISQVVVQP-MKQLDLLTSGTIA-----PN--PLALLDSLEMSELVAMARK--EY---DLV
2505787175_Chr6712_3399	LANAVYQP-MAKLDLLLSTSRV-----AN--PLALLDSPEMGELIAQGRK--TY---DLV
2508650743_Xen7305DRAFT_00028420	LADAVTQP-MLNLKLLASGEVV-----SN--PLAILDSSAMNELIAESRK--GY---DLV
2509711668_Pleur7313DRAFT_05656	WEDSIQNV-MPNLDVLTAGSIS-----DN--PIALLDSSFIKAFIDNVSK--HY---DQV
2509712372_Pleur7313DRAFT_06363	WHHAIQKV-MPNLDLITSGTTS-----KH--PISLLNSLLIKTFIADLSD--YY---DCI
2505785156_Chr6712_1404	WYDVVQNV-MPNLDVLTAGTIP-----DN--PVVLLDSLSMGLLVMKILN--HY---DLV
2503799529_Sta7437_1457	WQEAIKTI-KPGLDILTAGEPP-----DN--AVPLIDSERMKHLIGTTAL--NY---DHV
2506749054_Syn7336_3731	LSQVIQPV-SPHLDVLAMGDRP-----SP----PTLDYRKFRSMLAVLSR--HY---DRI
2503799932_Sta7437_1855	LDQAIKSV-IPNLDVLTAGKAL-----PN--ALALLNSPQMRSLFEELQQ--DY---DLI
2505786145_Chr6712_2386	ISETIQQI-TPYIHLIPSGLAQ-----QG-ESLMLLEPKKLATLIERVK--YDY---DLV
641538163_MAE_41520	LSLAIKNI-SKNLDLILAGHQV-----IN--PLALFNSDKMESLL--YESLETY---DFI
2507085801_Pse6802_0485	--SVIHTV-IPNLNLLLAGKLP-----SNTLSLSLLKSQRMSALMEKWAD--IY---DYV
2507089189_Pse6802_3805	--ATQKSV-MPNLDLLLSGTIP-----SNPLSL--LDSNRMTALMKNWSE--DY---DFV
2508689585_Syn7502_02785	--TVLESV-VPNLDILSSGVLP-----PDPLTL--IDSQHMTNLIGDWSR--AY---DYV
2504679565_Pse7367_1141	LSEVAHHV-MPTLEVMPAGSKP-----AN--PVALLNSQATSNLIDYGSS--NY---DVM
2503802282_Sta7437_4176	SSAIKE-V-MLNLDVLPSGVIP-----PS--PVTLLDSQKMINLIDFFAT--RY---DFV
2509709361_Pleur7313DRAFT_03349	RTVTEK-V-MPNLDLLTSGVIA-----PS--PAALLDSQRMRMLMDYWTE--RY---DFV
2508651492_Xen7305DRAFT_00035910	DSVKQQ-V-ITNLDLIPSGAIP-----PS--PVTLLDSRRMKSLVDHCAT--IY---DFI
2505784713_Chr6712_0967	SRLAIETV-MTNLSVITSGAIP-----SS--PGNLLDSYRMKDLMHYWSRV--Y---DFI
2514739634_ACCM5_010100028418	VKMIIHRV-PGNLDVLTSGSKP-----AS--PMSLLSSERMHLLMQYFA--WNY---DFV
637461727_glr4310	WQTAVRPTPQPGLVVLTAGPPP-----PN--PVVLLESRRLGELLGLWSE--QF---DLV
641253915_AM1_4837	LDSATQSV-TAHVDVMTAGNLH-----AN--AMGTLESKHMTVMLNAAKSI--Y---NYI
2514739407_ACCM5_010100027286	LKSATQSV-TAHVDVMTAGNLH-----AN--AMGILESKHMTVMLNAAKST--Y---HYV
2509712141_Pleur7313DRAFT_06132	LDSAITSV-SDNLDVLSSGSLE-----LGFNPLTIIDSTKMKFLLWKLVE--KY---NFV
2506482932_LYNGBM3L_58950	--
2506608339_Spi6313_0712	SEQAIQSI-TPNLDIMSAGIVT-----PG-L-VALLDSERMEMLVRRMSEV--Y---DYV
641249511_AM1_0391	-ELPTHKV-ADNLEIITAGRIS-----SN--PLPMLESEQFANFVQ--VQIEHY---DYI

```
2514736711_ACCM5_010100013771          -SLPTHRV-SKGLDVITAGRIS-----SN--PLPMLESEEFSAFVQ--GQLDTY---DYV
2509500847_Pro9006DRAFT_2342           NLQTLVQPLTPYLSLLPSGPLP-----LN--PLALLDSQYTMDFIRQVSRW--Y---DCV
2504681110_Pse7367_2660                FYTTTQQV-EPRLELLTAGTLP-----TN--PAALLDSRPMNSLLSVAAR--NY---DLV
2509874159_Syn6308DRAFT_0866           ---------MDNLDILFAGNN-------QSNPVALIGSSQMLYLIEEIKK--EY---DLI
2509428796_Syn6312_0755                MHH-----ENSNLDIITSGLTP-----PN--PITIINSSSFLSFLNKLRVI--Y---DYV
2508653159_Xen7305DRAFT_00052600       PQSIIQRA-RPNLDIISAGET-------D-SPTPILDSPRMQSFLANYSNI--Y---DFI
2512978631_Fis9431DRAFT_2960           LEETVQRTHIANLSVLSCGDLY-----GR--PSHLLESEAMKSLITEATQ--RY---DCV
2517061062_PCC9339DRAFT_01917          LEETVQRTHIANLSVLTCGDLY-----GR--PSHLLESEAMRSVIIEATR--LY---DFV
2505768802_FJSC11DRAFT_1780            LEETVQRTNIPNLSVLTCGELH-----GR--PSHLLESEAMKSLLIEAVR--QY---DFV
2516146713_FIS9605DRAFT_03719          LDRAVQRTKIPNLSVLTCGELH-----GR--PSQLLESDAMKSLLAEAAG--KY---DYV
2507336787_Tol9009DRAFT_00067760       LKQAVQPTEIDNLHVLTGGEFH-----GR--PSQLLESLAMKSLVAEAAA--NY---DMV
2517242598_Mas10914DRAFT_3841          LLDAVQPTDIENLDVLTCGELH-----GR--PSQLLESIAMKSLIAEASE--NY---DLV
2507476104_Cal7103DRAFT_00030040       LKEAVQSTEIENLSVLTCGGIY-----GR--ASQILESAAMKKLIAEAAA--NY---DFV
2510090822_Riv7116_5676                FANAIQKTDVDNLDVLTCGELH-----GR--PSQLLESVEMKLILEEAAK--EY---DLV
2509766470_CylstDRAFT_0429             LLDAVQTRGMENLSVLTCGKTH-----SR--PSQLLESTAMKSLLADAIA--HY---DFV
637233445_alr3059                      LLSAVQSTTIENLSVLTCGELR-----GR--PSQILESAAMKSLVAEAAQ--RY---DLV
646566337_Ava_0852                     LLSAVQSTTIENLSVLTCGELR-----GR--PSQILESAAMKALVAEAAQ--RY---DLV
2509812587_Nos7524_5019                ------------------------------------------------------------
2507480867_Cal7103DRAFT_00077730       LADFVQPTAIENLSVLTCGSMS-----TR--PSQLLESTYMKALMMEAAE--NY---DLV
2504094392_Cal6303_1407                LTQAIQPTEIENLWVLTCGDFY-----GS--ASQMLESAAMRHIITEASE--HF---DLV
2506481239_LYNGBM3L_38240              WRSAVQPTAIANLSILTCSDQP-----SG--SATFLQSQMIKSILAAAAD--HY---DLV
2503613794_Chro_3525                   LAEAVQTTGIDKLKVLCSGRPR-----SH--PSELFESRQIQQVIAAAAS--QY---DLI
2510437494_Cha6605_1495                LEQSVQPTRIANLSLLTCGNLV------H--SSFGLDSARMKSLLAEAER--QF---DLV
2509773567_Lepto7104DRAFT_0787         LETAIQPTLLEHLFLLPCGRVK-----GH--PATLSESPRTEDLLETLRD--RY---AWT
641254791_AM1_5726                     LPAAIHRTSVKNLSVMTSGKF-------SYDNVFSFETQRAKELFTEVQ--QHY---DVI
2514737653_ACCM5_010100018486          LPAAIHRTSVKNLSVMTSGKF-------SYDNVFSFETQRAKELFTEVQ--QHY---DVI
637459597_gll2202                      -------TAVSNLDVLGAGSLQL------PDSGSLLDEEAVDALIGALEP--HY---EQI
gi|4512007|gb|AAD21564.1|              AEMIIDKVEGGGFDYIGRGQI-------PPNPAELLMHPRFEQLLNWAS--QNY---DLI
gi|53987110|gb|AAV27324.1|             YEKAVVHTDIAGLDYIGCGEI-------PPNPAELLMGSRIEKFLEWAS--GKY---DLV
637230853_all0493                      -------PEVENLYIIPS-------PGPVRQSAAILESSEMRRLMEDVRE--RY---DLV
646568404_Ava_2908                     -------PEVENLYIIPS-------PGPVRQSAAILESSEMRRLMEDVRE--RY---DLV
2509812367_Nos7524_4799                -------PDVENLYIIPS-------PGPVRQSAAILESSEIRRLMEDVRE--RY---DLV
642599777_Npun_F0459                   -------PDVENLYILPS-------PGPVRQSAAILESSEMRRLIEDVRQ--RF---DLV
2509772030_CylstDRAFT_5990             -------PEVENLYILPS-------PGPVRQSAAILESSEMRRLIEDARQ--RY---DLV
2503740929_Nos7107_2283                -------PDVENLYIIPS-------PGPVLQSPAIIESSEMRRLMEDARE--RY---DLV
640027950_N9414_23213                  -------PEVENLYIIPS-------PGPVSQSSAILESSEIKRLMEDVRE--RF---DLV
2505798270_Cal7507_0021                -------PDVENLYIIPS-------PGPVQQAPAILESSEMRRLMEDARE--RY---DLV
2509781264_Mic7126DRAFT_2015           -------PDVENLYIIPS-------PGPVRQSASVLESSEMHSLIKDARA--RY---DLV
2507337082_Tol9009DRAFT_00070720       -------PDVENLYIVPS-------PGPVRQSAAILESSEMRKLMEDARQ--RY---DLV
2517243673_Mas10914DRAFT_4916          -------PDIENLYILPS-------PGPVRQSAAILESSEMRRLMEDVRE--RF---DLV
2504133191_Anacy_3523                  -------PDVENLYIIPS-------VGPVRQSAAIIESSEMRRLIEDARE--RY---DLV
2506492757_Ana7108_2905                -------PDVENLYIIPS-------AGPVRQSAAIIESGEMRRLIEDARE--RY---DLV
648049213_Aazo_0828                    -------PEIENLYIIPS-------AGPALQSAAIIESSEMVRVIEDARQ--RY---DLV
2512978956_Fis9431DRAFT_3285           -------PDVENLYIVPS-------AGAVPQPAAILESSEFRRLIEDARE--RY---DLV
2517059344_PCC9339DRAFT_00197          -------PDVENLYIVPS-------VGSVHQPASILESSEFRCLIEDARE--RY---DLV
2505768976_FJSC11DRAFT_1951            -------PDIENLYIVPS-------VGPVSQPAAILESSELRRLIEDARE--RY---DLV
2516145655_FIS9605DRAFT_02658          -------PDVENLYIVPS-------PGPVRQPAAILESSELRHLIEDARE--RY---DSV
2512633064_Chl7702DRAFT_1938           -------PKVENLYIIPS-------PGPVRRPTAVLESSELRRLIADARE--RY---DLV
2510085904_Riv7116_0758                -------PDIENLYIVPS-------PGPLRQSAAVLESSELRRLMEDARE--RF---DLV
2504093110_Cal6303_0131                -------PDIENLYIVPS-------AGPVRQSAAIIESSEMRRLLEDARE--RF---DLV
2507480421_Cal7103DRAFT_00073250       -------PEVENLYIVPS-------PGPVRNSAAILESSEMRRLMEDVRA--RF---DFV
2503609163_GEI7407_3152                -------PEVENLYVIPS-------PGPQRQAAAILESSEMRRLIDDVRG--RF---DLV
2509421114_Oscil6304_1907              -------PEIENLYVVPS-------AGPLKNSAAAIESNEFRRLLEDAKA--RF---DLV
2509805590_LepboDRAFT_4932             -------PDVENLYIVAS-------PGPQASAASLLESSELRRLLEDARA--RF---DLV
646129274_AplaP_010100007922           -------PDVENLYVIPS-------PGPLKQVTAVLESSEMRRLLKEVRH--RF---DVV
650387413_NIES39_K02750                -------PDVENLYVIPS-------PGPLKQVTAVLESSEMRRLLKEVRH--RF---DVV
643168837_AmaxDRAFT_1210               -------PDVENLYVIPS-------PGPLKQVTAVLESSEMRRLLKEVRH--RF---DVV
648390350_APCC8_010100026493           -------PDVENLYVIPS-------PGPLKQVTAVLESSEMRRLLKEVRH--RF---DVV
640015518_L8106_06195                  -------PDVQNLYVIPS-------PGPVRQAATVLESSEMKRLLNEVRY--RF---DMV
648859640_OSCI_3640012                 -------PAVENLFVIPS-------PGPLRQAAAVLESSEIRRLLTEVRH--RF---DFI
2508873356_Oscil6407DRAFT_00014730     -------PAVENLFVIPS-------PGPLRQAAAVLESSEIRRLLTEVRH--RF---DFI
2504092741_Osc7112_6177                -------PEVENLYVVPS-------PGFLRQASAVLESSEMRRLFEDVRN--RF---DFV
2506346135_MicvaDRAFT_1862             -------PEVENLYVVPS-------PGFLRQASAVLESSEMRRLFEDVRN--RF---DFV
638107751_Tery_2688                    -------LEVENLYIIPS-------PAVVKQPGRIIESSEMQKLLSDSRH--RF---DFV
2506480833_LYNGBM3L_31450              -------QDFENLYILPS-------LGPLRKAAGILESSEMKHLLEDARG--RF---DFV
647565613_MC7420_2957                  -------PNIQNLYIVPS-------PGPQKQAAAILESREFQQLIRDARN--RF---DFV
2510102323_Gei7105DRAFT_3578           -------PQVENLYTIPS-------PGPQQHAAAIIESSELRRLLSQARV--QF---DFV
2509498590_Pro9006DRAFT_0077           -------PWVDNLYLIPS-------VGPQAHPAAVLESSEMRALIKDCKG--RF---DVV
2509773034_Lepto7104DRAFT_0253         -------PWVENLYLAPS-------PGPQPHPAAILESSEMGQFLVDARA--RF---DLV
2517693907_LEP6406DRAFT_3365           -------PGVENLYIAPS-------AGPQRNAPMVIDSSEMIRFLADAQA--RF---DLV
2509844778_Lepto7375DRAFT_4795         -------PFVENLYVSPS-------PGPQRQAAAILESSEMRRFLDDARE--RF---DMV
641676481_cce_1994                     -------PGIANLYVLPS-------PGPQRQAAAIIESSELRLLLKDARG--RY---DMV
2507502153_Cy51472_3013                -------PGIANLYVLPS-------PGPQRQAAAIIESSELRLLLKDARG--RY---DMV
640626984_CY0110_27525                 -------PGVPYLSILPS-------PGPQSQAAAIIESSELRLLLDNVKG--NY---DMV
2503335332_CWat_WH8501_draft2_00062360 -------PGVANLYLLPS-------PGPQRQAAAIMESSELQLLLKDARG--RY---DMV
2531849533_CWATWH0003_2441             -------PGVANLYLLPS-------PGPQRQAAAIMESSELQLLLKDARG--RY---DMV
643475556_PCC8801_2514                 -------PGVTNLYILPS-------PGSQRQAAAIIESSELQLVLKDVRG--RF---DLV
644981523_Cyan8802_3592                -------PGVTNLYILPS-------PGPQRQAAAIIESSELQLVLKDVRG--RF---DLV
2509576278_Ple7327_4396                -------PTIENLYILPG-------PGPQRRAAAIIESSELQLLLKDAKG--RY---DMV
641537721_MAE_37160                    -------PNIENLSILPS-------PGPQKQAAAIIESSELQLILKDSRG--RF---DLV
648188704_Cyan7822_3790                -------PAVENLYILPS-------AGPQKRAAAIIESSELQLLLKDARG--RF---DMV
2503801406_Sta7437_3306                -------PAIANLYIVPS-------LGPLRQVPAIIESNEFRRLIEDARG--RF---DVV
2505785267_Chr6712_1514                -------PAIANLYLVPS-------PGPSRQVAAILESNELQRLIEDARG--RF---DVV
2506598905_Spi9445_1800                -------PDVMNLYISPS-------AGPQRKAAAIVESSELKSLLEDARQ--RF---DLV
2506609325_Spi6313_1684                -------PDVANLYVIPS-------AGPQRNAAMLLESSELLRLLQDARV--RY---DLV
2503367585_Cyast_2162                  -------PEIPNFSVVPS-------PGFQQQAPAILESSEIKSFLRYARA--QY---DFV
gi|16124419|ref|NP_418983.1|           ----------------ESGAHILPLAKSSYTPRDVLGSSAMHRLLGELRG--RY---EIV
gi|16264834|ref|NP_437626.1|           ----------TKLAILPAGGASHQ----RHQSNELLASPAMANLIENAR--NAF---DYV
```

```
2508652600_Xen7305DRAFT_00047000        -----------NLNVITAGSM-------VEDYLSLWQSESMKEFMLHVR--EKY---DLI
2506480821_LYNGBM3L_31350               --------IQPSLDLLPT--SHKKGKI-----VELVTQGKFEQSLATAEESGDY---DYV
2509432410_Mic7113_0575                 --------LEGGLDLLPT--SPKKGKI-----VELVRRGRFEQSLAVAESANKY---DYV
2507482189_Cal7103DRAFT_00090960        --------IQPNLDLLPT--LPRQGKI-----VEIIRRGNFEKTLATCEANNKY---DYI
2509801296_LepboDRAFT_0638              --------LQPNLDFVPT--MPQTGKI-----VDLVKRGRFERYLAVAESQKDY---DYV
2505802696_Cal7507_4407                 --------IEPQLDLLPA--IAQQGKI-----LKLVSRGQFKKALADAEAHSVY---DYV
2507332342_Tol9009DRAFT_00023250        --------IEPNLDLLPA--IPQHGKI-----LEMISGGRFQQALADAKSHAEY---DYV
2510087202_Riv7116_2056                 --------VEPNLDLLPA--TPRSSKI-----VQMVSQGRFQQALADAESHTKY---DYV
2510089901_Riv7116_4755                 --------VRFNLDLLSVNIAED--KI-----SEFVARGEFEQYLNLIQAQGNY---DYV
2506745681_Syn7336_0399                 --------VKPGIDLVPS--LPLGSDT-----AKFLARGGFAEYVQQARATGNY---DYV
637457826_glr0454                       --------LAPSLNFLPACLPEAGLSV-----GEYIAKGQFTRYLAELERTGNY---DYV
637459188_gll1796                       --------LAPSLSFLPAGRPDERTSV-----GEYIARGHFSRYLSELESAGGY---DYV
637461130_gll3716                       --------VIADLDLVPALAIEDTSTA-------FFVQGGFERYLDDVQTDG-Y---DYV
2503610992_Chro_0741                    --------VSPGFDLLPALSIP-KQKI-----PEFFARGSFERNIKALRESGGY---DYV
2503793842_Glo7428_1287                 --------VSLGLDLIPAPAVR-REKI-----AEFFARGSFERNLNQIQSSGGY---DFV
2505800320_Cal7507_2052                 --------INYRWDLLPAMQISNGRAV------EFVARGGFERHLDAIQKAGKY---DYV
643588131_Cyan7425_5174                 --------LSPGLDLMPAPAIPN-DRI-----VNYVAKGGFEQQLQTLQQSGEY---DYV
643167924_AmaxDRAFT_0301                -D-RLEPAFNYGVKLVSMAFLI------DRDQPVIWRGPMLNGIIRQFLYQVYWGELDYL
648389440_APCC8_010100021805            -D-RLEPAFNYGVKLVSMAFLI------DRDQPVIWRGPMLNGIIRQFLYQVYWGELDYL
646130866_AplaP_010100015993            -D-RLEPAFNYGVKLVSMAFLI------DRDQPVIWRGPMLNGIIRQFLYQVYWGELDYL
650384994_NIES39_D01080                 -D-RLEPAFNYGVKLVSMAFLI------DRDQPVIWRGPMLNGIIRQFLYQVYWGELDYL
640015631_L8106_17697                   -E-VLEPAFNYGVKLVSMAFLI------DRDQPVIWRGPMLNGIIRQFLYQVQWGELDYL
647567948_MC7420_2316                   -E-VLEPAFNHGIKLVSMAFLI------DPDQPVIWRGPMLNGIIRQFLYQVNWGELDYL
638106387_Tery_1165                     -E-VLQPAFNHGVKLVSMAFLI------DKDQPVIWRGPMLNGIIRQFLYQVQWGELDYL
648857672_OSCI_2870002                  -D-ILEPAFNYGVKLVSMAFLI------DKDQPVIWRGPMLNGIIRQFLYQVQWGDLDYL
2508875395_Oscil6407DRAFT_00035160      -D-ILEPAFNYGVKLVSMAFLI------DKDQPVIWRGPMLNGIIRQFLYQVQWGDLDYL
2509801138_LepboDRAFT_0480              -E-ILEPAFNHGVKLVSMGFLI------DKDQPVIWRGPMLNGVIRQFLYQVRWGDLDYL
2512979571_Fis9431DRAFT_3900            -E-ILEPAFNHGVKLVSMGFLI------DRDQPVIWRGPMLNGVIRQFLYQVQWGELDYL
2517061293_PCC9339DRAFT_02148           -E-ILEPAFNHGVKLVSMGFLI------DRDQPVIWRGPMLNGVIRQFLYQVQWGELDYL
2505768399_FJSC11DRAFT_3149             -E-ILEPAFNHGVKLVSMGFLI------DRDQPVIWRGPMLNGVIRQFLYQVQWGELDYL
2509782632_Mic7126DRAFT_3383            -D-VLIPAFNHGVKLVSMGFLI------DRDQPVIWRGPMLNGVIRQFLYQVEWGELDYL
2504683355_Cri9333_0934                 -E-ILEPAFNHGVKLVSMGFLI------DPDQPVIWRGPMLNGVIRQFLYQVEWGELDYL
2503606101_GEI7407_0138                 -D-ILQPVFNHGVKLVSMGFLI------DPDQPVIWRGPMLNGIIRQFLYQVAWGELDYL
2509502622_Pro9006DRAFT_4121            -D-ILEPAFNHGVKLVSMAFLI------DKDQPVIWRGPMLNGIIRRFLYDVQWGDLDYL
2509778293_Lepto7104DRAFT_5514          -E-VLEPAFNHGVKMVSMGFLI------DRDQPVIWRGPMLNGIIRQFLYQVEWGELDYL
2517694920_LEP6406DRAFT_4378            -E-ELEPAFNHGVKMVSMGFLI------DRDQPVIWRGPMLNGIIRQFLYQVQWGDLDYL
647577822_S7335_3227                    -Q-VLEPAFNHGVKMVSMGFLI------DRDQPVVWRGPMLNGVIRQFLYQVQWGDLDYL
2503637469_PCC7418_2732                 -E-VLEPAFNHGVKLVSMGFLI------DPDQPVVWRGPMLNGIIRQFLYQVNWGELDYL
2509552467_Dacsa_0081                   -E-VLEPAFNHGVKLVSMGFLI------DTDQPVVWRGPMLNGIIRQFLYQVNWGELDYM
2503367528_Cyast_2106                   -D-ILEPLFNHGIKMVSMGFLI------DPDQPVMWRGPMLNGIIRQFLYQVNWGDLDYL
2503887475_Lepto7376_1861               -D-ILQPEFNHGVKIVSMAFLI------DPDQPVMWRGPMINSMVRQFLYQVNWGELDYL
637688026_PMN2A_1153                    -Q-KIIPIETCGIGMVSMGLLI------DQNQPVIWRGPMLNGIIRQFLYQASWGERDFL
640085320_NATL1_20271                   -Q-KIIPIETCGIGMVSMGLLI------DQNQPVIWRGPMLNGIIRQFLYQASWGERDFL
641286083_P9211_17011                   -Q-KIIPIESAGIAMVSMGFLI------DEDQPVIWRGPMLNGIIRQFLYQTSWGERDVL
640547039_SynRCC307_2084                -Q-RLTPIESCGLAMVSMGLLI------GPDQPVIWRGPMLNGIIRQFLYQVDWGERDVL
637798215_PMT9312_1673                  -DQRLIPINKYGISLVSMGFLI------EEGQPVIWRGPMLNSIIRQFLYQVEWNNLDFL
640161373_P9301_17721                   -DQRLIPINKYGISLVSMGFLI------EEGQPVIWRGPMLNSIIRQFLYQVEWNNLDFL
640944474_P9215_18531                   -DQRLIPINKYGISLVSMGFLI------EEGQPVIWRGPMLNSIIRQFLYQVEWNNLDFL
647672827_P9202_63                      -DQRLIPINKYGISLVSMGFLI------EEGQPVIWRGPMLNSIIRQFLYQVEWNNLDFL
640079518_A9601_17881                   -DQRLIPIYKYGISLVSMGFLI------EEGQPVIWRGPMLNSIIRQFLYQVEWNNLDFL
637450610_PMM1581                       -DQRLIPINKYGISLVSMGFLI------EEGQPVIWRGPMLNSIIKQFLYQVEWNNLDFL
640081464_P9515_17681                   -DSRLIPINKFGISLVSMGFLI------EEGQPVIWRGPMLNSIIKQFLYQVEWSNLDFL
646569576_Ava_4074                      -EKFL-PLEVQGIKLMSVGLLA------EENRPLAWRGPVLHKIITQFINDVEWGELDYL
2505803290_Cal7507_4972                 -DKFL-PLEVQGIKLMSVGLLV------EENRPLAWRGPVLHKIITQFLQDVEWGELDYL
2517700475_SYN7509DRAFT_4594            -QKFL-PLEAYGIKVMSVGLLA------EPEHPLAWRGPVLHKIVTQFIHDVEWGELDYL
2506747473_Syn7336_2171                 -QRFQ-PLEAFGIEVMSVGLLA------EPEHPLAWRGPVLHKIITQFIHDVEWGELDYL
gi|1172092|gb|AAA86371.1|               -------------------------------PRSRLN-----------------------
gi|15597431|ref|NP_250925.1|            ---------------------------------------------------------EVV
637450246_PMM1225                       -----KSNINNYLDIISSGQ--------DLSNKMLLGSLRMKELIENLK--NNY---QYI
gi|53721457|ref|YP_110442.1|            --------------------------------AWTITFKRTRS-----------------
638961258_RS9917_04770                  --------ERGAVALIPVGN---------------LDPAALEAFSAELRRALGS---SRE
639888778_RS9916_38577                  ---------HNSIALITLGK---------------IDDAIVKNFSSHLQTYLGA---NKT
637444523_SYNW0419                      --------DAQSVALIPVGN---------------FPNNQIEQLQQALVAALG----NRK
640545089_SynRCC307_0169                ------------------------------------------------------------
640084152_NATL1_08771                   ---------KGSIAFLAVGE---------------IDEEELMHLSKLLRKFTN----SPE
647672433_P9202_965                     --------QSKKIKFLTTNK---------------IDKAIKTKLINILFDREDY---N--
637797888_PMT9312_1355                  --------------------------------------QDKFTLRESLQEIDKD---DLI
640944066_P9215_14561                   ----------KNINI----------------------EDKFTHLIQLLE---KY---KFK
gi|397405|emb|CAA52655.1|               ------------------------------------------------------------
gi|455023|gb|AAB51624.1|                ------------------------------------------------------------
gi|1107924|emb|CAA62141.1|              ------------------------------------------------------------
gi|53720415|ref|YP_109401.1|            ----------------------------------AMEPKRVRNIF---------------
gi|45297|emb|CAA38731.1|                ----------------------------------ALEPSRIYNII---------------

2505798636_Cal7507_0387                 IYDVPCLVGMVDARLLAPY-TDGMVLVVRLDKTD-KSGLTAAQDSLKLS----------P
2509783408_Mic7126DRAFT_4160            IYDVPCLVGIVDARLVAPH-TDGIVLVVRLDKTD-KSGLIEAQDSLRIS----------P
648052066_Aazo_4925                     IYDAPPMLGLVDARLLAPQ-TDGMLLVVRIDKTD-KSAMMQLQDSLINS----------P
2504133443_Anacy_3769                   IYDVPPMLGLVDARLLAPQ-TDGMLLVVRIDKTD-KSALMQLQDSLRNS----------P
2506494313_Ana7108_4449                 IYDAPPMLGLVDARLLAPQ-TDGMILVARIDKTD-KSALIQLQDTLRSS----------P
2512980750_Fis9431DRAFT_5079            IYDAPPLLGLADASLIAPH-TDGILLIVRMEKTD-SSVLKRALESLKTS----------R
2517062414_PCC9339DRAFT_03271           IYDAPPLLGLADASLIAPH-TDGILLIVRMEKTD-SSVLKRALESLKTS----------R
2505767894_FJSC11DRAFT_3537             IYDAPPLLGLADASLLAPH-TDGILLVVRMEKTD-SSVLKRALDSLKTS----------R
2516145585_FIS9605DRAFT_02588           IYDAPSLPGLADASLLAPH-TDGILLVVRIDKTD-SSVLKRALDSLQMS----------R
2504096080_Cal6303_3081                 IYDAPELVGLADANLLLPH-TNGLLLVARMGKTD-SSLLKRSLDNLKLS----------R
2507333046_Tol9009DRAFT_00030320        IYDAPVLVGLADASLIAPH-TNGILLVVRMDKTD-SSVLKRALDNLKLS----------R
2507476338_Cal7103DRAFT_00032380        IYDTPHLDELADASLLVPQ-TNGLLLAVRMDKTD-TSLLKRALDNLKLS----------R
2505802134_Cal7507_3852                 IYDVPPLLKLADANLLAPH-TDGILLVVRIDQTN-SSIVQRTLDNLTIS----------P
```

```
2509782674_Mic7126DRAFT_3425          IYDTPCLSGLADASFLASN-TDGILLVVRIDKTD-STIIERALDHLKIA----------S
2509771979_CylstDRAFT_5939            IYDAPPLVGLADASLLAPL-TDGMLMLVRIDKTD-SSVLKRALEHLKIS----------R
2503738747_Nos7107_0132               IYDVPPLVGLADVSLLAPN-TDGILIVVKIGKTQ-SLVLNRAIDNLKLS----------R
2509809495_Nos7524_1926               IYDAPPLVGLADASLIAPQ-TDGILMVVRMDKTE-RSVFERTLADLKIA----------P
640026730_N9414_07896                 IYDTPPASGLADATLIGSQ-TDGLLIVARIDKTD-RSVFERAVADLKLAP----------
2517239228_Mas10914DRAFT_0471         IYDAPPLLGLADASLLAPC-SDGIMLVARMHKTD-RSALKEAIDSLKIG----------R
2510087477_Riv7116_2331               IYDTPPLVGLADANLLAPY-TDGVVVVTRIHQTD-RSVMMQALDSLKMA----------R
2510087323_Riv7116_2177               IYDAPPLVGLADASLLAPH-TDGVVLVVRIDKTE-RSIVKQAVDSLKTS----------R
2507477177_Cal7103DRAFT_00040780      IYDAPPLLGLADATLLAPQ-TDGIVFVARLHKTN-TSVIKQALDRLKTS----------R
637235631_all5222                     IYDAPHFFGLSDVSVISPY-TDGVLMVVRIGKTD-ASVIEQALDYLKIL----------P
2506482271_LYNGBM3L_49700             IYDTPSVLGLADASLLTSH-TDGLILVSRIGKTD-RSALTQALENLKLS----------Q
641611510_SYNPCC7002_A1500            VYDTPPVLGLADASLLASH-TDGVILVVTLNKTT-RSAVSQAIATFKQA----------N
2509842234_Lepto7375DRAFT_2251        VYDTPPLVGLADATLIAAN-TDGLILVSRLNGCD-RNILQHAMDNISFT----------K
647107641_CRC_03354                   IYDTPPVMGIVDSRLVASQ-TDGLILVVKMHKTD-RSMIKQAQDALRQS----------S
647110196_CRD_02439                   IYDTPPLSGLVDAKLLASQ-TDGLLLVVAMHKTN-RPAIAEVKNSLEES----------S
2509804452_LepboDRAFT_3794            IFDAPPLLNFADSSLLANH-TDGMLVVTRLGKTD-REDLRQMLDTLQTA----------R
647567980_MC7420_2223                 IFDTPPMLGLADARLLAAH-TDGIVMVVGLGRTD-RTVLMQVLSGLKTS----------H
2509437593_Mic7113_5758               LFDTPPLLGLADARILAAH-TDGIALVVGLGKTD-RAVLTEVLYGLRTS----------R
2506479760_LYNGBM3L_56010             IFDTPPLLGLADARLLEPN-TDGILMVVGLGKAN-QVLFKEALSELRIA----------H
647567242_MC7420_5618                 IYDTPPVLGLADGRLLAAH-ADGVMMVVGLGKSD-RSALMQALDGMRIS----------N
2509436287_Mic7113_4452               IYDTPPVLGLADGQLLAAH-TNGVVMVGGLGKTD-RSMMMQSLDRLRIS----------N
2503796318_Glo7428_3737               IYDTPPMLGLADSSLLAPY-TAGIVLVVRMGKTD-RSLLAQALDRLKMS----------P
2517697153_SYN7509DRAFT_1270          IYDTPPILGLADSSIVATH-TAGIVLVVRMAKTD-RSVVRQALDGLKLS----------R
2503615076_Chro_4796                  IYDTPPLLGLADASLLAPL-TAGIAIVTRMGKTD-RALLMQTLDQLKLS----------R
2509422734_Oscil6304_3527             IFDTPPLGAISDAKFLAPH-TDGLVMVIGMGKSD-RAILREVFDGLKMS----------R
2506601543_Spi9445_4406               IFDTPPMLGLADAKFLAAQ-ADVMLMVVRLGWTD-RAIFKQVIDGLKVS----------Q
2506609271_Spi6313_1630               IFDTPPMLGLADAKFLAAH-SDVMLLVVRLGWTD-RTFLKQVLESLKVS----------H
640014711_L8106_15385                 IYDTPPCTDVVDSKILAPH-TDGLLMVVRLKKTD-KNLLNQALESLKLS----------R
2509507761_Osc10802DRAFT_1411         IYDTPPLLGLADGSLLAGH-TNGILLVVSMGETD-RSEVKQALEGLQMA----------G
2509420889_Oscil6304_1682             IYDTPPLLGVVDGRVLAAY-TDGIGLVVSMGQTE-KPAIQQAIAELQTA----------N
2503798162_Sta7437_0103               IYDTPPILGFADGRILAGR-TNGVVLVIRMNKTD-RSLLKQNIDNLKMS----------N
2505785430_Chr6712_1674               IYDTPPILGFADGRILASR-TNGVVLVVRIGKTD-RSLLKQNLDNLKVS----------H
2509711143_Pleur7313DRAFT_05131       IYDTPPILGFADGRILSTR-TNGVVIVVRIGKTD-RSLLKQNIDNIKMS----------N
2508648278_Xen7305DRAFT_00003760      IYDTPPILGFADGRILASS-TNGVVLVVRMGKTD-RSLLKQNLDNLKVS----------N
2508646941_GLO73106DRAFT_00031960     IYDTPPLLGFADARILAAK-TTGIILVAKIGKTD-RSAFKQSIEQLKMS----------H
2509573356_Ple7327_1473               IYDTPPVLGFADGRILAAL-TSGIVLVTRINKTD-RTALKNCVDQLRLA----------H
641537293_MAE_32940                   IYDLPPIAAFADAKILAAM-ATGLILVTKLGKTD-RFVLKNLLEDLRLS----------H
643482531_PCC7424_4310                IYDTPPLLNFADAKIVAAL-TQGIILVTKMGKTD-RKAFKHCVDELRGS----------Q
648185689_Cyan7822_0712               IYDTPPILGFPDAKILAAS-TEGLILVTKMGKTE-RQAFKDCLDELKMS----------A
2509502225_Pro9006DRAFT_3724          IYDTPPLLGLADASLLAAH-LDGILLAVGVGKTD-QSALKAVLSNLRVS----------N
643584098_Cyan7425_1087               IYDAPPVVGFADSVLLSSN-TDGCLLVVGLGQTE-KAALQQALDYLKVS----------N
2503742029_Nos7107_3348               IVDTPPLHGFADAKFLASQ-TNGLIMVIGLDKTE-KTAAKQVLNDLQIS----------H
2504097563_Cal6303_4545               IYDAPNILGRVDTNILTTH-TDGIVLVVGLGKTL-RPNLKKLVEELKAT----------R
2507478127_Cal7103DRAFT_00050290      IYDTPNMLGRLDTNALINH-SDGILLVVGLGKTR-RPNFKQVIEELKSG----------R
2510089327_Riv7116_4181               VYDTPHILGRLDTSILATH-ADGILMVVGLGKTV-RPSLSTALDELEAG----------R
2517239184_Mas10914DRAFT_0427         VYDAPHIMGLLDTNILASR-VDAVLMVAGLGKTI-RPSLQQALEELKTA----------R
2507335438_Tol9009DRAFT_00054260      VYDTPHLLGRLDTNVLLSQ-VDGIVLVSGLGKAL-RPTLKQALEELKSS----------R
2504086624_Osc7112_0134               IYDTAPLLGLADANLLAAH-TNGLMLVVGLHQTE-REALLLAFEDLKMA----------G
2506346341_MicvaDRAFT_2066            IYDTPPLLGLADANLLAAH-TNGLMLVVGLHQTE-REALLLAFEDLKMA----------G
648859632_OSCI_3640004                IYDTPPLLGLADANLLGAH-TNGLMLVVGLNQTE-REALLLALEDLKMA----------G
2508872900_Oscil6407DRAFT_00010170    IYDTPPLLGLADANLLGAH-TNGLMLVVGLNQTE-REALLLALEDLKMA----------G
643171775_AmaxDRAFT_4132              VYDTAPLLGLADANLLASH-TNGLMMVVGLGKTD-RSAFNLALRELEMA----------G
648386075_APCC8_010100004460          VYDTAPLLGLADANLLASH-TNGLMMVVGLGKTD-RSAFNLALRELEMA----------G
650384684_NIES39_C03520               VYDTAPLLGLADANLLASH-TNGLMMVVGLGKTD-RSAFGLAMRELEMA----------G
646131598_AplaP_010100019750          ------------------------------------------------------------
640018815_L8106_14065                 IYDTAPLLGLADANLLAPH-TNGLMMVVGVGKTD-RAAVDLALRELQMA----------G
638107046_Tery_1925                   ICDTAPLLGRADANILAAC-TNGLLLVVGLGKTE-REALSLALDDLSIA----------G
2506483316_LYNGBM3L_63940             IYDTPPVIGLADSRLLAAN-TDGMVFVGRLNKTD-RSVLMQALDELKTS----------R
648856139_OSCI_1010012                IYDTPPLVGLADGHILAAH-TDGTILVVALDRTD-RSLVTKALEGLKIS----------G
2508875360_Oscil6407DRAFT_00034810    IYDTPPLVGLADGHILAAH-TDGTILVVALDRTD-RSLVTKALEGLKIS----------G
2509510258_Osc10802DRAFT_3909         IYDTPPLAGLSDGNILAAH-TDGMVMVVGLDKTD-RSMVDKALDGLKIS----------G
2509438066_Mic7113_6231               IYDTPPLLGLADGNILAAN-TDGIVLVVGLQKTD-RSLLTKSLDGLKIS----------G
2504090796_Osc7112_4249               IYDTPPLAGLADTHLIAAH-TDATIMVVQIGQTD-RSQVRKVLEELKIS----------G
2506345448_MicvaDRAFT_4285            IYDTPPLTGLADTHLIAAH-TDATIMVVQIGRTD-RSQVRKVLEELKIS----------G
2504091687_Osc7112_5131               IYDTPPLMGLADGHLLASQ-TDGTVLIVKIEKTD-RGLVNKALDQLKVA----------D
647568459_MC7420_6195                 IYDTPPLLGLGDSNLVAAQ-VDGTILVVGMEKTD-RSLMMKALDRLKIA----------R
647572642_MC7420_919                  IYDTPPLLGLGDGNLIAAQ-ADGTLLVVAIEKTD-RSLITKAFDGLKIA----------G
640014779_L8106_06284                 IYDTPAVTGLADTSLIAAQ-TDGIILVVGIKKTN-RSTVLKSLEELNLS----------N
2509421881_Oscil6304_2674             IYDTPPLLGLADGSILAAQ-TDGFVMVVKIDKTE-RSVVVKALDGLKIS----------G
641253939_AM1_4861                    IYDCPPL-NLADPTIIAPQ-TDGLMVVAHLGSVP-RSLLKESLRMLEIA----------Q
2514735482_ACCM5_010100007602         IYDCPPL-NLADPTIIAPQ-TDGLMVVAHLGSVP-RSLLKESLRMLEIA----------Q
2510441931_Cha6605_5932               IYDTVPL-NFADSLLLIPQ-TDGLLMVTRLGKVH-REALRNALRTLEVS----------K
2503611061_Chro_0810                  IYDTSHLIGFADANLLARH-VDASILTVSMGKTN-CSALVKALEQLNFS----------A
2503613235_Chro_2969                  VYDTSPLLGLADSNLLSAH-IDASILVVGMGKAH-RSALAKALESLKIV----------G
2512979625_Fis9431DRAFT_3954          IYDTPPLVGFADATLLAAN-TNGMVLVAGLGKLK-RTVFQQALEELQIS----------G
2517059612_PCC9339DRAFT_00465         IYDTPPLVGFADATLLAAN-TNGMVLVAGLGKLK-RTVFQQALEELQIS----------G
2505768848_FJSC11DRAFT_1825           IYDTPPLVGFADATLLAAN-TNGMVLVAGLGKLK-RTVFQQALEELQIS----------G
2516149265_FIS9605DRAFT_06272         IYDTPPLVGFADATLLAAN-TNGMVLVAGLGKVK-RSVLQQALEELQIS----------G
2517241827_Mas10914DRAFT_3070         IYDTPPLVGFADANLLAAN-TNGLILVAGLGKVK-RTMFQQALEELQVS----------G
2507335136_Tol9009DRAFT_00051240      IYDTPPVLGFADAYLLAAN-TNGMVLVAGLGKLK-RTVLQQALEEIQVS----------G
2504095135_Cal6303_2139               IYDTPPVVGFADANLLAAN-TNGMVLVAGLGKLK-RTLFKEALDELQIS----------G
2507476798_Cal7103DRAFT_00036990      IYDTPPIVGFADANLLAAN-TNGMVLVAGLGKVK-RTVFQQALEELQVS----------G
2510085606_Riv7116_0460               IYDTTPIVGFADANLLAAN-TNGVMLVAGLGKLK-RTVFTQALEEIQIS----------G
2503739631_Nos7107_1000               IYDTPPLLGFADAYLLASN-TNGIVMVAGLGHLK-RTALKQVLEEIQIS----------G
2505801704_Cal7507_3428               IYDTPPLLGFADAYLLATN-THGIILVAGLGKLK-RTALQQVLEEIQIS----------G
2505804241_Cal7507_5918               IYDTPNLLSVADANLLAAH-TNGMILVTELGKLK-RTAFQQALEELQIA----------G
643168344_AmaxDRAFT_0719              IYDAPPMLGLADANLLASE-TSGMVLVASLGRIN-RSVLENAIAQLKEHP-------NMS
648390241_APCC8_010100025948          IYDAPPMLGLADANLLASE-TSGMVLVASLGRIN-RSVLENAIAQLKEHP-------NMS
```

```
646129996_AplaP_010100011596          IYDAPPMLGLADANLLASE-TNGMVLVASLGRIN-RSVLENAIAQLKEKP-------NMS
650383923_NIES39_A06230               IYDAPPMLGLADANLLASE-TNGMVLVASLGRIN-RSVLENAIAQLKEKP-------NMS
640017922_L8106_10512                 VYDTPPLTGLADAFLLAAE-TNGLILVTGLGQVK-RSVLESALDQLKDRS-------KFS
2510101197_Gei7105DRAFT_2452          VYDSPIVLGLSDTHLLAAR-TQGVVFVTGLGHLK-RPFLEQALEELK----------SSG
2509573281_Ple7327_1398               IYKASSLSEFADPYLLANH-TNGILLVAGLGKLK-RPVLEHALDELRIS----------G
2506597130_Spi9445_0041               IYDAPPVLGLADSHLVATH-TDGVILVAGLGQIK-RSILEQALYDLRVSG-------N--
2503636620_PCC7418_1894               IYDTTILRGFTDAHLLATG-TNGLILVTGIGRLK-RSLLERTVENLTLS----------R
2506607995_Spi6313_0371               IYDAPPLAGLADAHLLAAH-SDGLLLVAGLGTVK-RSALEQSLYELAVA----------N
643585148_Cyan7425_2153               IYDTPPACDLADASLLATK-TDGMALVIGLGNTD-RSAFISTLESLRLSP----------
2504681848_Pse7367_3384               IYDAPPL-SFADAYLLAPR-TEGILMVTSLGKQK-RSGLEEALEKLRIS----------G
647577962_S7335_3828                  IFDTPPLLGFADSLMVAQI-TQGLLLTVRLGEIK-SSQLQQALDRLYTA----------R
2509846600_Lepto7375DRAFT_6617        VFDAPSLLGQSDAYLVAEQ-ADGMLIVTNPGQIK-QNLINQALEQLRIA----------N
2509774530_Lepto7104DRAFT_1750        IYDSPPFAEYADAALLSAE-TSGLILVSHLGTVK-SIQLEQALEKLWIA----------K
2509804417_LepboDRAFT_3759            IYDAPPVLGFSDSLILSGQ-VDGSVLIVVSHLGTVK-RTALIESLRGLQMS----------G
2531850144_CWATWH0003_3517            IVDCPPLVGLPDAKLVSQN-TDGLLLVLRLDKT-MKDVVKEALGEIKLA----------Q
2509777916_Lepto7104DRAFT_5137        IFDTPPLAGMIDAKLTAAH-ADGLLLVVRLHRSE-RSEIQRVLADL--GN-------TAQ
648856194_OSCI_1040007                IYDTPPLIDAKDANFIGAR-TEGILMVVAVLKTK-NSVVKQVLSQLE----------SFG
2508875344_Oscil6407DRAFT_00034650    IYDTPPLIDAKDANFIGAR-TEGILMVVAVLKTK-NSVVKQVLSQLE----------SFG
2504087727_Osc7112_1201               IYDTPPLADSKDTNFLAAR-TDGILMVVAIQQVP-LSVVKKVFSQLE----------NFG
2506348042_MicvaDRAFT_3627            IYDTPPLADSKDANFLAAR-TDGILMVVALRQVP-FSLVQKVFSQLE----------NFG
2509438406_Mic7113_6573               VYDTAQFGSFMDTNFLAAH-TDGILMVVAVGKTK-RTLVMQVLDQLN----------TYR
647571294_MC7420_5066                 VYDTPEFPSCMDISYLARH-TDGLLMVTAIQKTK-NSLVKQTITQLD----------AFG
2506481927_LYNGBM3L_44990             IYDTPNLLNYTDANFLAAH-TDGILMVVGFRGTK-RSQFKQVLDQID----------RFG
640019302_L8106_03117                 IYDTPQLKDYTDGTFLTGH-LDGLIMVVGIAKTH-QSVTENVLAQLN----------KFH
2509707887_Pleur7313DRAFT_01875       IYNTSPILESMDSVFLATH-TDGILMTAAINKTK-KSLFTQALNQVN----------NFN
641251898_AM1_2808                    IF---------------------------------SEALKDFVDVQ----------TQA
2514737774_ACCM5_010100019113         IYDTPYLQGHADANFLSIN-ADGLLMVVGIGKTQ-QSSLLKMLKDLQ----------ASR
641251897_AM1_2807                    IYDTPYLQGHADANFLSIN-ADGLLMVVGIGKTQ-QSSLIKMLKDLQ----------ASR
2512634931_Chl7702DRAFT_3806          IYDTTHLLGFADASFIAEH-ADGILLVVGVGKTK-RSSITQALNRLQ----------MLH
2516146228_FIS9605DRAFT_03233         IYDTPHLLGFADSYFLAEH-TDGILMVVGVGKTK-RSLLTQALHRLR----------MLH
641675948_cce_1468                    IYDTPPFFLSSDIKFMAKQ-TDGIILVATIQKTS-QSLLKRAVKEIR----------ALN
2507501641_Cy51472_2500               IYDTPPFFLSSDIKFMAKQ-TDGIILVATIQKTS-QSLLKRAVKEIR----------ALN
640626055_CY0110_29394                IYDTPSFFLSSDVKFMAKQ-TDGILLVATIQKTP-QSLFKKAVKEIK----------KLN
2531850936_CWATWH0003_4939            IYDTSPFFLSSDIKFIAKQ-ADGIILVATIKKTS-QSLLTKAVKEIK----------SLN
643476430_PCC8801_3415                IYDVPHFFDNPDIKFLGSK-MDGILMVVTVQKTP-QSLAKKAIKDIE----------TLN
644980660_Cyan8802_2701               IYDVPHFFDNPDIKFLGSK-MDGILMVVTVQKTA-QSLAKKAIKDIE----------TLN
643478515_PCC7424_0240                IYDAPHFYESTDIGFLGAQ-TDGIVMVVGIGKTP-PYLVKQAVEEIN----------KLR
648186851_Cyan7822_1903               IYDAPHFYESTDIGFLGAQ-TDGILMVAGIGKTS-SSIFKQAMSEIE----------KLR
2509575480_Ple7327_3597               IYDAPKFLDFTDTSFLAAQ-TDGILMVAGVGKTS-QSLLKKAIARIN----------GFN
2508645044_GLO73106DRAFT_00012970     IYNSPLFLESPDVSFLANN-TDGILMVVRL-KSTSHSLVKQAFERI---N-------SFE
2509773763_Lepto7104DRAFT_0983        VYALPPLMGLADVSLVAAK-TDGVLLVTSLGRRHLADALNRTLDRMQVA----------R
2503745907_Cyan10605_1646             IYDSPSLLSFSDGKIIASQ-TNGLILVVKIGSTK-DYELRHVYEQLRVT----------N
2509874395_Syn6308DRAFT_1102          IYDSPSLLGFIDAKIIAPN-TNGLILVMRLGRSD-RNKLRIVTDQLKIS----------R
644979531_Cyan8802_1534               ------------------------------------------------------------
2503745344_Cyan10605_1086             IYDTPPLMGFSDGKILTPL-TDGLVFVVRMGKTR-RPNVQQVINDLQVS----------K
2509873750_Syn6308DRAFT_0457          IYNTPPLAEFSDGKIVTPL-TEGLVMIIGLGKTN-RNNLEKVMNDLQIS----------K
2505785711_Chr6712_1954               IYDVASIVDYADVGLLASK-TDGVILVTGLGKLQ-ALKLKEAMNQLNIS----------R
2509706287_Pleur7313DRAFT_00275       IYDLCAITGFADVNLLAAK-TDGIIVVTGLGKIQ-SMALTEALNQLRLC----------P
640027738_N9414_07903                 ------------------------------------------------------------
2506482929_LYNGBM3L_58920             IIDTPSLNVAADAPILGKM-TDGVLLVSRPGVVDSGSA--AFA--------------FW
637461200_glr3785                     IIDSPPMIGLADATILTKC-ADGLVFVIGLEVARRGGV--LSALERLRSA-------SLV
2503887239_Lepto7376_1629             ILDIPSMLGLADATLFSRN-TDALILVTSLHKTR-QTILQETLDELENK----------Q
638113739_sync_0150                   LFDTPPVLGLADASLVAEH-CDGLMLLVSLNRVD-RGLPQEAVNRIVSS----------G
638963744_RS9917_02426                VFDTPPVLGLADAALVAEH-CDGLMLLVSLDRVD-RGLPKESVARIRSS----------G
2507493170_Syn8016_1895               LFDTPPVLGLADAALVAEH-CDGLMLLVSLDRVD-RSLPKEAINRIRSS----------G
638960084_WH5701_13945                LYDTPPVLGLADASLLAEN-LDSILLVVGLNKVD-RQLPQQAIDRIRSA----------G
650128418_SCB01_010100004274          LFDTPPALDLVEPLLLGDH-LDGLLFVVSLGRID-RNLPSQSLKRIQAS----------N
650132168_SCB02_010100007823          LFDSPPALDLVDPLLLGDH-LDGLLFVVSLGRID-RSLPDQAIKRIQTS----------E
2508551611_Cyagr_1223                 IVDAPPCLMLADPILLGEK-LDGILFLVGLGKVS-RELAPQASRRIRAS----------G
640081072_P9515_13861                 VFDTPPVIGLADSLLVSEK-SDGLILLVSTNSVN-KSLPKESISRSIKS----------G
648050855_Aazo_3125                   LIDAPSVIGMVDAILTASS-CRSVVMVASIGKVT-RNNLAQATAMLS----------KLN
2504129904_Anacy_0284                 LIDSSSVLGLVDAMLTASS-CRSVVMVASIGRVT-RNNLAQATAMLS----------KLN
2506490031_Ana7108_0216               LIDAPPVLGLVDAMLAASS-CRSVIMVASIGKVT-RTQLTQATAMLS----------KLN
2509770736_CylstDRAFT_4696            LIDAPPVLGLVDAMLTASS-CRSVVMVASIGRVT-RTQITQATAMLS----------KLN
642600344_Npun_R1070                  LIDAPPVLGLVDAMLTASS-CRSVVMVASIGIVT-RSQLTQATAMLS----------KLN
2505799741_Cal7507_1481               LIDASPVIGLVDAMLTASS-CRSVLMVASIGRVT-RTQIAQATGMLS----------KLN
2509779661_Mic7126DRAFT_0411          LVDASSVIGVVDAMLTASS-CRSVLLVASIGRVT-RSQIAQATAMLS----------KLN
2503739922_Nos7107_1290               LIDAPPVLGLVDAMLTASS-CRSVVLVASMGVVT-RNQLAQATGMLR----------KLN
640027761_N9414_00965                 LIDGPPVLGLVDAMLTASS-CRSVVLAASMSKIT-RSQLAQATAMLS----------KLN
637233217_alr2833                     IIDAPPVIGLVDALLTASS-CRSVVMVASLGKVT-RNQIAQATAMLS----------KLN
646566603_Ava_1116                    IIDAPPVIGLVDALLTASS-CRSVVMVASLGKVT-RNQIAQATAMLS----------KLN
2509808002_Nos7524_0433               ILDAPPVLGLVDAMLTASS-CRSVVLVASMSRVT-RAQIAQATAMLS----------KLN
2517241466_Mas10914DRAFT_2709         LIDSTPVLGLVDAMLTASS-CRGVVMVASIGRVT-RTQLAQATAILS----------RLN
647105946_CRC_01573                   LIDAPSVLGMVDAIITGSS-CRSVVMVASIGKVT-HNHLAQATSMLS----------KLN
647109494_CRD_02102                   LIDAPSVLGMVDAIITGSS-CRSVVMVASIGKVT-HNHLAQATSMLS----------KLN
2507332201_Tol9009DRAFT_00021840      LIDASPVLGMVDAILTASS-CRSVVMVASIGKVT-RTQLTQATTMLS----------KLN
2512975951_Fis9431DRAFT_0279          IVDAPPVLGLVDALLTASA-CRSVIMVASIGRVT-RTQLAQATAMLS----------RLN
2517062640_PCC9339DRAFT_03498         IVDAPPVLGLVDALLTASA-CRSVIMVASIGRVT-RTQLAQATAMLS----------RLN
2505767181_FJSC11DRAFT_4188           IVDAPPVLGLVDALLTASA-CRSVIMVASIGRVT-RTQLAQATAMLS----------RLN
2516146836_FIS9605DRAFT_03842         IVDAPPVLGLVDALLTASS-CRSVVMVASIGKVT-KAQLAQATAMLN----------RLN
2512634275_Chl7702DRAFT_3149          IVNASPVLGLVDALLTASS-CRSVVMVASIGRIT-KNQLAQATAMLG----------RLN
2510089385_Riv7116_4239               ILDASAVLGLVDALLTASN-CRGAIMVASIGGVT-RTQLAQATAMLG----------KLN
2504094820_Cal6303_1825               LIDAPPVIGLVDALLAGSA-CRSVVMVASIGRVT-RTQITQATTMLN----------KLN
2507482515_Cal7103DRAFT_00094220      LVDAPPVLGLVDALLAGSA-CRSTVMVASIGRVT-RTQLTQATGMLN----------RLN
2503612562_Chro_2300                  LLDAPPLLGMVDAMIAASC-CTGVVLVSRVGMVK-KTELVQANAMLR----------KLN
2517695945_SYN7509DRAFT_0062          LVDAPPVLGMVDAILAASF-CSGVLLVSRMGKVT-RTELTQATAMLS----------KLN
2503608186_GEI7407_2184               VLDAPPVLGMVDAMLAGSF-CQGVMLVGRIGQVT-RTELGQAAHTLH----------QLN
2504685208_Cri9333_2761               LLVAPPVLGMVDALLIGSC-CQGVVLVTRLDRVT-KTALAEATAMLN----------KFN
```

```
2509431928_Mic7113_0093                      LVDAPPVLGLVDSILTASY-CGGVLLVARMGQIT-KTELTQATAMLS----------KLN
2506479159_LYNGBM3L_28310                    LLDTPPVLGMVDALQAASL-CQGVVMVGRLERVT-QSQLSQATAILR----------NLN
640017638_L8106_27951                        LLDTSPILGTVDVLETASF-CDGVVLVERIDQIT-QTELNQATTMLK----------KLN
2509806250_LepboDRAFT_5593                   ILDAPPVLGIVDALLASSC-SDGVLLVGRMGHVQ-KSEIAQAVGMLN----------RLN
2504583836_Pse7429DRAFT_1713                 IVDAPPILGTVDTIQIASY-CGGVVSVARIDRIT-RGEFSQAISVLQ----------KLN
641251010_AM1_1910                           LIDSPPVLGVVDTIPIGLG-CDGVVMVGRMKQVT-RLELSKAISAHK----------RLN
2514735759_ACCM5_010100008979                LIDSPPVLGVVDTIPIGLG-CDGVVMVGRMKQVT-RLELSKAISAHK----------KLN
2509847582_Lepto7375DRAFT_7599               IVDAPAISGLADGLQLASM-CNASIIVSRLDRIT-QADLTHTTTMLD----------QVN
648051793_Aazo_4516                          LIDASSVLDNVDARIIASV-CNGIVIVGRIGQLT-PQKLIQATEVLS----------QLN
2504134691_Anacy_5006                        LIDASSVLDSVDARIIASA-CNGIVIVGRIGQLT-PQELMQATEILS----------QLN
2506494605_Ana7108_4733                      LIDAPSVLDSIDARIIASV-CNGIVIVGRIGQLT-PQELMQATEILS----------QLN
647107726_CRC_03451                          IIDACSLLDGVEARIMASV-SNSIVMVGKIGQLT-PDQLIQAREILS----------DFN
642604187_Npun_R5250                         LIDAPPILGMVDARIVASF-CNAIVMVERMGKVT-RTELTQATEILS----------QLN
2509767727_CylstDRAFT_1687                   LIDAPAIFGTVDARIVASL-CNGIIMVGRIGQVS-PNELIQATEVLN----------QLN
2507335141_Tol9009DRAFT_00051290             LIDAPTILGTVDARILATY-CHQIVLVEKMGQVT-RTELIQATEILG----------KLN
640028130_N9414_07219                        IIDAPPILGTVDSRILASF-CDGIVIVGRIGQIT-PNELMQTTEILS----------NLN
637230414_all0059                            LIDAPPILGTVDGRIVASY-CHGIMMVGRIGWVT-QTELTQAVEILN----------SLN
646568157_Ava_2661                           LIDAPPILGTVDGRIVASY-CHGIMMVGRIGWVT-QTELTQAVEILN----------SLN
2509811005_Nos7524_3436                      LIDAPAILGTVDARIMASL-CNGIMMVGRIGWVT-QTEVTQAVEILN----------QLN
2503739635_Nos7107_1004                      LVDAPPILGTADARILAGY-CQEIVMVGRMGQLT-QSDLIQSQEILS----------QLN
2509781657_Mic7126DRAFT_2408                 LIDASAISDMVDARILAPL-CDGIIMVSRIGQVK-RNELIQAKEILS----------NLN
2517243237_Mas10914DRAFT_4480                LIDTSPILGTVDARIMASL-CNGIVMVGRMNRVT-RNSLIQATEILS----------NLN
2512634393_Chl7702DRAFT_3267                 LIDAPAILSMVDARILATF-CNSIVMVAHLGRVT-ATEVIQATSILS----------NLN
2505769181_FJSC11DRAFT_2156                  LIDAPAILKTVDARILATL-CDRIIMVSCLGKIT-QPDVIQVTDILK----------NLN
2512977368_Fis9431DRAFT_1696                 LIDAPAILKTVDARILATL-CDRIIMVSRLGKVT-QADLIQATDILK----------NLN
2517063601_PCC9339DRAFT_04460                LIDAPAILQTVDARILATL-CDRIIMISRIGKVS-QADLIQATDILK----------NLN
2516147062_FIS9605DRAFT_04068                LIDAPAILNTVDARILATF-CDRIVMVARIGKVS-LADLIQATEILS----------HLN
2507482923_Cal7103DRAFT_00098300             LVDAPPILNTVDARILATL-CNAIVIIARLGQIT-RAELTQTKEILE----------NLN
2510085595_Riv7116_0449                      LIDASSILDTVDARILASL-CSGIIMVGRMGKIT-HTELVQATQILS----------SLN
2503614917_Chro_4637                         LVDAPPILGLADASIVASL-CSGTVVVERLNRVT-RFDLNQSLEALS----------KLN
2504093446_Cal6303_0467                      LIDTPQILKTIDARILATL-SKGIIMVERMGQVT-RSELAEAIAILN----------QHN
647578236_S7335_5395                         ILDSPPVLGSADVLEIAAC-CGDLVLVAPLNQTT-EEELQASLAALS----------QLN
2503798844_Sta7437_0777                      LIDTPPVLGMVDAIKISGY-CGGTMIVSRLNQVK-ATELIEATNLLC----------NSN
2509708827_Pleur7313DRAFT_02815              LVDTPPAIGMVDAIKVASN-CDSAVLTMRLDKLK-ASELLEVEALFS----------KLN
637009749_sll0923                            LIDTPPILGLTDSRLIADQ-TDGLVVVVRLNKTR-KDSIKEAFRELALAD----------
2503800689_Sta7437_2597                      LIDTPPIGVMADAKSLANQ-VDSVLFVVGMERAT-RRAINNSLEVLRSS----------Q
2509710958_Pleur7313DRAFT_04946              IIDTPPIGVIADAKSLAKE-VDSILFVAGIEKAS-RKAISNSVDVLRHS----------R
2505786361_Chr6712_2601                      IIDTPPIGIMADAQSLIHQ-VDTVLLVAGIDRAS-QKSLAHTVEILRSN----------E
2508650693_Xen7305DRAFT_00027920             LIDTPPIGAMADAQSLVHQ-VDGVMLVTGINKVN-QKAIVNTLEVLQSS----------N
2506480412_LYNGBM3L_19610                    LIDTPAVVGLADTQSVGSY-VDGIIFVAGMECST-HDDITRSLEILRSN----------Q
2509512444_Osc10802DRAFT_6095                LVDTTPLLGVTDAQSLAPK-ADAVILVAALERST-RSSLAHTMEVLQRS----------R
643587900_Cyan7425_4938                      LVDTPPVLGVPDIQSIGCR-FDGVVMLVAMNGPT-TAALTRAVEILRGS----------R
2504582150_Pse7429DRAFT_3127                 LVDTPPVIGITDAQCLTSK-VDTFILVAAINRST-RSGISRALEVLASA----------R
2504679882_Pse7367_1451                      LIDTPPIVGITDAQCLAGK-VDSVVLVAAIERST-RSAIARAVEILGHS----------R
2507088905_Pse6802_3551                      LFDTPPIVGLADAQSLAAK-VDAVVMVAAIQKST-RTAIARTVDILIKR----------H
2508687870_Syn7502_01070                     LIDTPPIVGITDAQSLAPR-MDGVVLVTAIEKAT-KQAIFRAIEILKIN----------N
2506748948_Syn7336_3628                      LIDTPPIVGFADAQSVASI-VDGVVLVVSLEMAS-KASVLRATEILQAE----------H
643484053_PCC7424_5769                       LIDTPSLTELADAQSLTPK-VDEVILTVDMELIT-DSMLTETIEILRRN----------Q
648199484_Cyan7822_5718                      LFDTSSLTGLADAQTITSK-VDEVILVISQNRVE-ISLVTEALKILQRN----------N
2510436294_Cha6605_0295                      LFDTPPIVGVSNTRLLSSL-VDGLVYIVSLNVAQ-KQIIDRGIDII---S-------SGK
637314397_tll1767                            IVDTPPLAHFADGKLWSGL-VEQTLVVVNLGAPRQPVALALADYSL------------GS
637313820_tll1199                            IVDVPAILAAADTYRVLTA-LDRALLVVRLRKTR-EAALAEALKA---------CDLGLR
2512980079_Fis9431DRAFT_4408                 IFDAPPLAGTADAAVLGKL-ADGVLLVVRPEVVDFASA--NAAKEFLIQS-------G--
2517061743_PCC9339DRAFT_02599                IFDAPPLAGTADAAVLGKL-VDGILLVVRPEVVDFASA--NAAKEFLTQS-------G--
2505770929_FJSC11DRAFT_1293                  IFDAPPLAGTADAAVLGKL-ADGMLLVVRPEVVDYASA--NAAKEFLTQS-------G--
2516148164_FIS9605DRAFT_05171                IFDTPPLAGTADTAVLGKL-TDGILLVVRPGVVDWNSA--NAAKEFLTQS-------S--
2512634416_Chl7702DRAFT_3290                 IFDTPPLAGTADAAVLGKL-VDGLLLVVRPGVVNSASA--HAAKEFLIQS-------G--
2505800360_Cal7507_2092                      IFDTPPLTGIPDAAVLGKM-TDGILLVVRPGIVDSASA--SAAKEFLDQS-------G--
2509780096_Mic7126DRAFT_0847                 IFDTPPLAGTPDAAVLGKM-TNGVLLVVRPGIVDSASA--TTAREFLSQS-------G--
2505800523_Cal7507_2254                      IFDAPALAGTVDAAVLGKL-ADGILLVVRPGVVNSSSA--NAAKDFLKHS-------G--
2507478259_Cal7103DRAFT_00051610             IFDTPALAGTADAAVIGNL-TDGTLLVVRPGVIDYASA--NAAKGFLAQT-------S--
637234831_all4432                            IFDTPPLTGIADAAVLSTL-TDGILLVVRPGVVDFNSA--NAAKEFLTQS-------G--
646566875_Ava_1386                           IFDTPPLTGIADAAVLSTL-TDGILLVVRPGVVDFNSA--NAAKEFLTQS-------G--
642600743_Npun_R1496                         IFDTPPLSGIADAAVLSTL-TDGILLVVRPGVVDLNSA--NSAKEFLNQS-------G--
2507334816_Tol9009DRAFT_00048030             IFDTPALSGTADAAVLSNL-TDGILLVVRPGVVNLDSA--NAAKEFLTQS-------G--
2507476704_Cal7103DRAFT_00036050             IFDTPSMSGTADAGVLSKL-ADGMLLVVRPGVVDLDSA--NATKEFLTQS-------G--
2510090628_Riv7116_5482                      IFDTPAVVGTADAAILSDL-TDGILLVVRPGVVESDSA--NAARDYLNQS-------H--
2506483552_LYNGBM3L_64290                    ILDTPALAGMADAPILGKM-ADGILLVARPEKVDSASA--NAAKEILNSS-------G--
2517697529_SYN7509DRAFT_1646                 IIDTPALNIGADAAILGKI-ADGILLVVRPGVVNSASA--TTAKEFLEHS-------G--
2503608035_GEI7407_2035                      IIDTPPMVLMADALSLAKM-TDGILLVSRPGVIDTVTA--RAAKELLNQS-------G--
2509433692_Mic7113_1857                      ILDTPPLILAADALSLSKM-TDGVLLVARPGILDRVSA--TAAKQFLVQS-------G--
640015133_L8106_09871                        IVDTSPLLLVADALTLGKK-SDGILLVARPKVIDSVSA--IAAKDLLTKS-------G--
2503795055_Glo7428_2485                      IIDAPPLVLAADAVTLGKM-TDGVLLVARPGVLDSSSA--AAAKESLERS-------G--
2509506717_Osc10802DRAFT_0363                IVDAPPLVIAADALTLGKM-ADGVLLVVRPEVVDYNSA--AAAKEYLQRS-------G--
2503614965_Chro_4685                         IIDAPPVLVAADALMLGKI-TDGLLMVARPGVVSSNTA--TTAKDLLQSS-------G--
2510091904_Riv7116_6758                      IIDAPSLVMRADALALGKM-ADGVLLVARPGILTSADA--ATAKEALERS-------G--
2503612629_Chro_2367                         IIDAPPLVVAADALTLGKI-TDGILLVARLGVVDYTNA--AAAREALKCS-------C--
2503612419_Chro_2158                         IIDAPAFSHAADALTLSHM-SDGVLFVARPGLLNYVSA--DAAKELLERS-------T--
2506493946_Ana7108_4087                      IIDAPPLVIGVEALTLGKM-TDGLLLVARPGIIDINSA--TTVEKYLDQS-------K--
2509803997_LepboDRAFT_3339                   IVDAPPLIVAAEALTLGRI-VDGLILVARPGVVDHANA--TTAKELLNQS-------S--
2512979777_Fis9431DRAFT_4106                 IFDTPPLVGIADAAVLGKM-ADGILLVTRPGVLDSPSA--AAAKSLLERS-------E--
2517062202_PCC9339DRAFT_03059                IFDTPPLVGIADAAVLGKM-ADGILLVTRPGVLDSPSA--AAAKSLLERS-------E--
2505770300_FJSC11DRAFT_0671                  IFDTPPLAGIADAAVLGKM-ADGILLVTRPGVLDAPSA--AAAKSLLERS-------E--
2507334440_Tol9009DRAFT_00044270             VFDTPPLVNTVDAAVLGKM-VDGVLLVAQPGVLDLASA--KAAKSLLERS-------E--
2510088639_Riv7116_3493                      IFDTPPLAGTPDAAVLGKM-VDGVLLVARPRVVDSASA--TAAKSLLERS-------E--
2505803639_Cal7507_5320                      VFDTPSLVGTADAAVLGKM-AGGVLVVVRPGLVDSGSA--TAAKSLLARS-------E--
2509782388_Mic7126DRAFT_3139                 VFDTPSLVGAAEAAVLGKM-AGGVLVVVRPGHVDSASA--VAAKSLLRRS-------E--
2507335607_Tol9009DRAFT_00055950             IFDTSPLVGSAEAAVLGNM-VDGVLIVVRPGVVDSSTA--TAAKSLLARS-------E--
```

```
2510088989_Riv7116_3843                      IFDAPSLVGTAEAAVLSQM-VDGALVVVRPGKVNSASA--AAAKSLVERS-------E--
2504096650_Cal6303_3648                      IFDTPPLAGTADAAVLGKM-ADGVLMVVRPGIANSDSA--IAAKSLLARS-------E--
2507481772_Cal7103DRAFT_00086780             IFDTPPLAGTADAAVLGKM-ADGVLVVVRPGVADLKSA--LAAKALLMRS-------E--
2509803885_LepboDRAFT_3227                   LFDTPPLAGTADAAVLGKM-ADGVLITVRPGVADAGSI--SAAKSLLDRS-------E--
647579433_S7335_2031                         ILDSSPFVGCADPSILGKV-ADGVVLVVRPGVLNAKAA--NAAREHLMST-------E--
2509846113_Lepto7375DRAFT_6130               IIDSPPILGLVDAITIGRA-TDGVLLVMRPGMANAENI--RATKTMLAQS-------K--
2512978854_Fis9431DRAFT_3183                 IIDAPSLTVAADATILGQM-SDGVLLVVRPGIADFANA--TTAIEILEKS-------G--
2516143706_FIS9605DRAFT_00708                IIDAPSLTLAADAATLGQM-ADGVLLVVRPGVVDSVNA--AIAIELLEKS-------G--
642600641_Npun_F1381                         IIDAPSLNVAADAATLGQM-TDGVLLVVRPGVVDSVQA--AVACEILEKS-------G--
2509770219_CylstDRAFT_4179                   IIDAPSLNVAADAATLGQM-ADGVLLVVRPGVVDSVNA--AFAKEVLEKS-------G--
2504094091_Cal6303_1107                      IIDTPSISVAADAAALGQM-ADGVLFVVRPGVADIGSA--GFAKDLLKKS-------G--
2507479320_Cal7103DRAFT_00062230             IIDTPSLSIGADAAALGQM-ADGVLFVVRPGVVDVVNA--AFAKDLLEKS-------G--
2510085390_Riv7116_0244                      IIDAPSLNLAADAATLGQM-ADGVLLVVRPGVVDSVNA--AFACEMLEKS-------G--
642601572_Npun_F2453                         ------------------------------------------------------------
2503611448_Chro_1193                         IIDTPALNAAADAAILGKM-TDGVLLVVRPGVVDTAAA--IRAKEFLEKS-------G--
2503796978_Glo7428_4384                      IIDTPALNVDAIAPILGKM-ADGVLLVVRPGVVDSASA--TFAKEFLAQS-------G--
2504087786_Osc7112_1259                      IVDTPALNVAADALILGNK-ADGILLVVRPGVLHSGTV--AFAKELLKKS-------N--
643584136_Cyan7425_1127                      LLDSPALNVADDPRILSQL-ADGVLLVVRPGVVSAAQA--QASKELLSQT-------E--
647105865_CRC_01492                          IIDTPPLTAVADALIVGKL-VDGVLLVVRPGQVESSAV--KASNSLLAQS-------K--
647108284_CRD_00419                          IIDTPPLTAVADALVVSKL-VDGVLLVVRPGRVESSAV--SAANTLLTQA-------K--
2505800344_Cal7507_2076                      IIDAPPLTAVADAQILGKL-VDGLLLVVRPGVVDSAAA--SETKTLLEQS-------E--
2509781889_Mic7126DRAFT_2640                 IIDAPPLTAVADAQILGKL-VDGLLLVVRPGVVESAAA--GAAKALLEQS-------E--
2507481279_Cal7103DRAFT_00081850             IVDTPPVTAVTDALLVSKQ-TNGILLVARPGVVQTDAA--NAAKMQLEQS-------G--
637233240_alr2856                            IIDTPPLSVLADASIVSKM-ADGMLLVVRPGVVNSAAA--KTTKTLIEHS-------R--
646566532_Ava_1045                           IIDTPPLSVLADASIIGKM-ADGMLLVARPGVLNSAAA--KTTKTLIEHS-------R--
640028261_N9414_00005                        ------------------------------------------------------------
642603803_Npun_R4851                         IIDTPALSLFGDALMLGKM-ADGILLVVRLGVLDCAVA--KSTKMMLEQA-------R--
2509770699_CylstDRAFT_4659                   IIDTPALSLFSDALMLGKI-VDGILLVVRPGVLDCAVA--KSTKMMLEQA-------R--
2507478691_Cal7103DRAFT_00055940             IIDTPPVIYAADALVLGKL-VDGTLVVVRPGATNSAIS--QEMKTLLTQS-------T--
641249527_AM1_0407                           LIDSPPLAVAADALLLGKM-TDGVLLVTRPGLVDTGSA--QSAKDALERA-------G--
2514736728_ACCM5_010100013856                LIDSPPLAVAADALLLGKM-TDGILLVTRPGLVDTGSA--QSAKDALERA-------G--
643586770_Cyan7425_3796                      LIDTPPIGVAVDALVVSKV-ADGILVVVRPDVTDSNTA--NAAREALVKS-------N--
2509555626_Dacsa_3240                        LVDTPPLAVAADAVILGRL-GQGVLLVVRPSVVAGGNL--DSTREILQNS-------G--
2509775597_Lepto7104DRAFT_2817               FIDTPPLLGAADAAVIGRM-SDGVLLVLRPRKVDSANA--IAAKSFLERS-------Q--
2506492920_Ana7108_3067                      IIDTSPLTVAADATILGKL-VNGILFVVRPGVADSNSV--SLSKEMLEKA-------D--
2512631781_Chl7702DRAFT_0655                 VIDTPPLTVAADATVLGKL-ANGILFVVRPGFVNSNSA--TFAKELLEKA-------G--
2504685956_Cri9333_3503                      IIDSPPVSVAADTTILGRM-ANGLMFVVRPGVANSGNL--TYCKEILDQS-------S--
2510436704_Cha6605_0705                      IIDAPPLTVAADAAILGTQ-AGGLVFVLRPGVADKESV--EYAQEILNQS-------KL-
2507482822_Cal7103DRAFT_00097290             VFDAPPILMAADALILGKI-TDGILMVSRPGVIDSNSA--INTKGLLEQS-------G--
2510087184_Riv7116_2038                      VIDAPPILMAADALILGKL-ANGILMVSRPGVVDANSL--VKTKNLLEQS-------G--
2506745596_Syn7336_0315                      AIDTPPLAVAADALIVGHL-SDGILMVARPGSLDSSSA--IAAKTALSQS-------N--
2503796286_Glo7428_3705                      IIDTPPLSIAADASILSKV-VDGVILVTRPGIADISSS--RFAKEYLDQS-------G--
642604427_Npun_F5505                         IIDAPPLLLAADALTISQM-TDGILLVARPGVIDSNSA--SAAQEILERS-------S--
2503612394_Chro_2133                         IIDAPPLLLTADALTLSQM-TDGILLVARPGVINANSA--NAAQEMLERS-------G--
2512632720_Chl7702DRAFT_1594                 IFDAPPLLIAADALTLGQI-TDGILLVARPGVIDYDSA--NAVQEMLKRA-------N--
2509807906_Nos7524_0337                      IIDSPPLLLAADALTLSQM-SDGILLVARPGVIDNSSA--DTVQEMLERC-------G--
2509770373_CylstDRAFT_4333                   IIDSPPFLLAADALTLGQM-TDGILLVARPGVINYNSA--ATAQEMLERS-------G--
646570355_Ava_4846                           IIDAPPLLVAADSLTLSHM-TDGILLVSRPNVIDSGSA--WAAKDTLERS-------G--
2510438837_Cha6605_2838                      IIDVPPILVASDALTVGQI-ADGVLLVSRPGVINAKDA--KAVQEKFKMS-------Q--
2506477923_LYNGBM3L_10200                    IIDAPPLLLTADALCLSPM-TDGMLLVARPGVLDFANA--NVAKQMLDSS-------G--
2509574529_Ple7327_2646                      IIDTPSLNLAADAPILSRM-ADGMLLVVKPGVVERDSA--IFAKEILEQS-------G--
643170532_AmaxDRAFT_2895                     IIDTPPLLLAADALTLANM-ASGVLMVARPRILDRDSA--KAAKEILGRS-------G--
648389308_APCC8_010100021105                 IIDTPPLLLAADALTLANM-ASGVLMVARPRILDRDSA--KAAKEILGRS-------G--
646129623_AplaP_010100009700                 IIDTPPLVLAADALTLANM-ASGVLMVARPRILDRDSA--KSAKEILGRS-------G--
650384826_NIES39_C04940                      IIDTPPLVLAADALTLANM-ASGVLMVARPRILDRDSA--KSAKEILGRS-------G--
2503801641_Sta7437_3540                      LIDAPPLPVTADVLTLSKL-VDGIVFISRPGVVEHESA--ELAQETLANS-------N--
2509711085_Pleur7313DRAFT_05073              LIDAPPLPVTADVLTLSKL-VDGIVFVTRPGVVEHESA--ELAQETLANT-------R--
2503801821_Sta7437_3719                      LIDAPPLPITADVLTLSKL-VDGLLFVSRPGVVEQESA--AFAQEILVTI-------N--
2505786500_Chr6712_2739                      IIDAPPLPVTADVLTLSKL-VDGIVFVSRPGIVEKESA--ELATEALEST-------R--
2508650623_Xen7305DRAFT_00027220             LIDAPPLPVTADVLTLSKL-VDGIMFVSRPGVVEHESA--ELAQETLIST-------G--
2509706990_Pleur7313DRAFT_00978              VIDAPPLSVTADVLTLSKL-ADGILFVTRPGIVETESA--ELAQETITNA-------NLS
2505787175_Chr6712_3399                      LIDAPPLPVTADVLTLSKM-VDGILFISRLGVVEQESA--ELAQEALTSI-------E--
2508650743_Xen7305DRAFT_00028420             LIDAPPLPVTADILTLNKL-VDGILFVSRPGVIEHESA--ELAQEVLDSI-------Q--
2509711668_Pleur7313DRAFT_05656              IFDTPPIIGIADTKMIGRL-VDGFLFVVRPGVADYGSA--TAAKKMLDST-------G--
2509712372_Pleur7313DRAFT_06363              IFDTPPLVGLADSKILGKL-ADGLLLIVRPGVANYNSV--AAATELLGDR-------D--
2505785156_Chr6712_1404                      IFDTPPIVGLADTKIISKL-VDGLLFVVRPGVANYSSV--TAAKKILENK-------D--
2503799529_Sta7437_1457                      IFDTPPITGMADTRILGRM-VDGLLMVVRPGVANYGSV--SVAKKLIETT-------G--
2506749054_Syn7336_3731                      VLDSPPLLATADAGILASM-ADGALVVVRPGLLDVASA--SAANDYLVNS-------NI-
2503799932_Sta7437_1855                      IFDTPPLLLFADALTIGKL-ADGILMVVRPGVIEPSSA--NASKEMLEQS-------G--
2505786145_Chr6712_2386                      VFDTPPVLLFADALTIAGQ-TEGIVFIARPGITSPASA--ASSKELLDQS-------G--
641538163_MAE_41520                          IIDAPPLLLNDITLQISPK-TDGIIFVVNPEKLETTTA--IQVQEILKKY-------Q--
2507085801_Pse6802_0485                      ILDTPPLLPVADAMVLSKQ-ADGVLIVARPELLSSPDA--SVAKKLLDKS-------DI-
2507089189_Pse6802_3805                      IIDTPPLLAVADAMVLGNL-ANGILLVARPDLLNSNNA--SRVRATLEQS-------GM-
2508689585_Syn7502_02785                     ILDTPPVLAVADAIVLGRI-ADGVLIVARPEVLDSGAA--IRTRAILDQS-------EL-
2504679565_Pse7367_1141                      IVDTPPMAVAADASILGKI-ADGIVMLCRVGVADTNNL--TFSKELLEQS-------G--
2503802282_Sta7437_4176                      IIDTPALNFAPDASIIGRI-TDGILLVVKPGVVDQTNA--KFTKELLEQS-------G--
2509709361_Pleur7313DRAFT_03349              ILDTPALDFTADAPIIGRT-ADGVLLVVRPGSVEKGQA--RFTKEILDQS-------G--
2508651492_Xen7305DRAFT_00035910             IIDSPALNKAADALTLGMM-ADGVLLTLKPGQVNYSEV--KFAQEILEQS-------G--
2505784713_Chr6712_0967                      IIDSPSLDRAADAPILGRM-ADGILLVVKPEGINHSQA--NFAKETLKGS-------G--
2514739634_ACCM5_010100028418                IFDTSPLLDVVGTSALANM-ADGVLFVTNPTMTQSKEL--EIATEIINQ--------FE
637461727_glr4310                            IFDAPPLTAASDALVLGAL-GGGLVLVVRPGVTNKRVL--AKVRDSLKRP-------GI-
641253915_AM1_4837                           LIDAPSLNSAADVSILGGQ-ADGIVIVVKPGIANLPNI--AAAKELLMRS-------G--
2514739407_ACCM5_010100027286                LIDAPSLNSAADVSILGGR-ADGIVIVVKPGIANLPNI--AAAKELLMRS-------G--
2509712141_Pleur7313DRAFT_06132              IIDSPPLLEVPDAISLGKL-ADGMILVSRLGVLDYASA--QECQELLEST-------G--
2506482932_LYNGBM3L_58950                    ------------------------------------------------------------
2506608339_Spi6313_0712                      IVDSPALLVAPEMLSLSKL-TDGVLLVLRPGVLNKADA--ASAQETVRQT-------DL-
641249511_AM1_0391                           LIDAPPLVSVADPLIIGKV-VDGILLVARPGHLEREYA--QKAHELLIHS-------NLS
2514736711_ACCM5_010100013771                LIDSPPLVSVADPLVIGKI-ADGIVLVARPEHLERELA--QKANELLTQS-------NLI
```

2509500847_Pro9006DRAFT_2342	ILDTPPLTRVADGALLGQM-VDGALWVMRTQFVDPSLA--VVARSLLLKS-------R--
2504681110_Pse7367_2660	LLDTPPVTVAADTTVLGKK-TDGVVLLVRPGLTEIDSF--AAAKDTLTRS-------R--
2509874159_Syn6308DRAFT_0866	IIDAPPISVAADAQILGRM-SDGMLVVIRQGKINTSMLAGTKESLIQAE-----------
2509428796_Syn6312_0755	LIDSPPLNAAADALFLGKI-SDCILLVARPDILIRPSA--QRAKDMLIQS-------G--
2508653159_Xen7305DRAFT_00052600	LIDSPALDANADAITLGNI-ADGMLLVVQSGKLNRSQA--KFAKELLNKS-------G--
2512978631_Fis9431DRAFT_2960	IIDTSPLSACADAQTLGRQ-SDGILLVTRPDFTIKEVL--QRAVSELTHN-------QI-
2517061062_PCC9339DRAFT_01917	IIDTPPLGACADGQTLGRQ-SGGILLVTRPNFTIKEVL--QKAVSELTHN-------QI-
2505768802_FJSC11DRAFT_1780	VIDTPPLSACADAHTLGRQ-SDGILLVTRPGFTIKEVL--QRAVSELTHN-------QI-
2516146713_FIS9605DRAFT_03719	IVDTPPLSACADAHTLSRQ-SDGIVLVTRPSITIKEVL--QRAVSELTNN-------QI-
2507336787_Tol9009DRAFT_00067760	IIDTAPISACADAATLARQ-SDGIMLVTRPSITIKEVL--QRAVSELEHN-------QI-
2517242598_Mas10914DRAFT_3841	IIDTAPLSACADASTLARQ-SDGVMLVTRPGFTEKEVL--SRSVWELTQN-------RI-
2507476104_Cal7103DRAFT_00030040	IIDTPPLSACADTATLAKQ-CEGVLMVTRPNITVKEVL--QRAVSELNNN-------HI-
2510090822_Riv7116_5676	IIDTPPVSACADAATLGKQ-GDGVVLVTRPSFTNKEML--QKAVSELTSN-------HI-
2509766470_CylstDRAFT_0429	IIDTPPLGISSDAATLSQY-SDGIILITRPHFTLKEIL--RKAVAELSRN-------RI-
637233445_alr3059	IIDTPPLSACADASTLSQM-SDGVILTTRPGFTLKEVL--QRAVSELNQN-------RI-
646566337_Ava_0852	IIDTPPLSACADASTLSQM-SDGVILTTRPGFTLKEVL--QRAVSELNQN-------RI-
2509812587_Nos7524_5019	--
2507480867_Cal7103DRAFT_00077730	ILDTPPLSACADATTLGQL-SDGIMLVTRPRYTIKEVF--QKAVSDLTRD-------RI-
2504094392_Cal6303_1407	IIDTPPLTICADAITLGRI-TEGLLLVTRPKVTVKEML--QRTITELSRI-------QI-
2506481239_LYNGBM3L_38240	IVDTPPVSSFADAHILSRY-SDGLVIVTRPNFTQKDIL--LQTVSELKDS-------ST-
2503613794_Chro_3525	VIDTPPVTSCVDAVSLCGN-GEKLLLVARPSFTQRDIF--TQAVAELRAK-------RV-
2510437494_Cha6605_1495	IVDTPALASSIDAFEWSHH-SDGLVLVVCPGVTTRNGL--AQAWADLHKE-------KV-
2509773567_Lepto7104DRAFT_0787	FVDTPVVSLSADAVSLSRY-ADGLVLVVRPGYTRRREL--QQAVADLRNS-------GT-
641254791_AM1_5726	VIDTPPVMACSDALVLSQE-GRQTVVVARLD-VTPKQVLNRSIDILNSN----------N
2514737653_ACCM5_010100018486	VIDTPPVMACSDALVLSQE-GRQTIVVARLD-VTPKQVLNRSIDILNSN----------N
637459597_gll2202	IIDTPPMAGYAHGHSLAAR-SEGVLLVLRPGHADIEHL--KHLKQTLDRN-------RI-
gi|4512007|gb|AAD21564.1|	IIDTPPILAVTDAAIIGRY-AGTCLLVARFEKNTVKEI-----------DVSMKRFEQSG
gi|53987110|gb|AAV27324.1|	LVDTPPILAVTDAAIIGPH-VGTTLLVARFEKNTVKEI-----------DVAKNRLEHSG
637230853_all0493	ILDTSPLSVSNDPLLIQPY-SDGIVLVSRVNYTQ-DSMMAEAIDQLVEAE----------
646568404_Ava_2908	ILDTSPLSVSNDPLLIQPY-SDGIVLVSRVNYTQ-DSMMAEAIDQLVEAE----------
2509812367_Nos7524_4799	ILDTNPLSLSNDPLLIQPY-SDGIVLVARPNYTQ-ENMLGEAIDQLVEDE----------
642599777_Npun_F0459	ILDTSPLSISNDPLLIQPY-SDGIVLVTRPHYTQ-ENMLGEAVDQLVEAE----------
2509772030_CylstDRAFT_5990	ILDTNPLSYSNDPLLIQPY-SDGIVLVARPNYTQ-ENMLNEAIDQLVESE----------
2503740929_Nos7107_2283	ILDTSPLSYSNDPLLIQPY-SDGIVLVARPNYTQ-ESMLGEAIDQLQEAE----------
640027950_N9414_23213	ILDTNPLSSSNDALLIQPY-SDGIVLVARPNHTQ-ENMLGEAIDQLVESE----------
2505798270_Cal7507_0021	ILDTSPLSLSNDPLLIQPY-SDGIILVTRPIYTQ-ENMLGEAVDQLMESE----------
2509781264_Mic7126DRAFT_2015	ILDTSPLSLSNDPLLIQPH-SDGIVLVTRPSYTQ-ENMLSEAVDQLMESE----------
2507337082_Tol9009DRAFT_00070720	ILDTSTLSLSNDPLLIQPY-SDGMVLVARPNYTQ-ENMLGEAVDQLTESE----------
2517243673_Mas10914DRAFT_4916	ILDTSPLGLSNDALLIQPY-SDGIVLVTRPNYTQ-ENMLSEAIEQLSESD----------
2504133191_Anacy_3523	ILDTNPLSISNDPLLIQPY-SDGIVLVARPNYTQ-ENMLGEAIDQLVEAE----------
2506492757_Ana7108_2905	ILDTNPLSISNDPLLIQPY-SDGIILVARPNHTQ-ENMLGEAIDQLVEDE----------
648049213_Aazo_0828	ILDTNDLSTSNDALLMQPY-SDGIVLVARPNYTQ-ENMLTEAIDQLVEGE----------
2512978956_Fis9431DRAFT_3285	IIDTNSISLSNDALLIQPY-SDGIILVTRPHHTQ-ENMLGEVIDELVESE----------
2517059344_PCC9339DRAFT_00197	IIDTNSISLSNDALLIQPY-SDGIILVTRPHYTQ-ENMLGEVVDELVESE----------
2505768976_FJSC11DRAFT_1951	IIDTNPISLSNDALLIQPY-SDGIILVTRPHYTQ-ENMLGEVVDELVESE----------
2516145655_FIS9605DRAFT_02658	IIDTNPLSASNDALLIQPY-SDGIVLVTRPHYTQ-ENMLGEAIDGLVESE----------
2512633064_Chl7702DRAFT_1938	ILDTSSLQVSKDALLVQPY-SDGIVLVTRPNYTQ-ENILNEVIDELVESE----------
2510085904_Riv7116_0758	ILDTTPLGLSNDALLIHPY-SDGMIMVTRPNYTQ-ENILGEAIDELVESE----------
2504093110_Cal6303_0131	ILDANPLGLSNDALLIQPY-SDGIILVARPNYTQ-ESVFGEAIDQLVESE----------
2507480421_Cal7103DRAFT_00073250	ILDSNPLGLSNDALLIQPY-SDGMILVARPNFTQ-ENVFSEAIDQLVESE----------
2503609163_GEI7407_3152	VIDTPSLSRCNDALLLEPF-TDGIVLVTRPGYTQ-DSLMGAAIDEFVESD----------
2509421114_Oscil6304_1907	ILDSPALSVNNDAFLLEPF-SDGMILVTRPLYTQ-GGMLTEYVEPLTESET---------
2509805590_LepboDRAFT_4932	VLDSPCLSKCNDAFILEPY-TDGMILVTRPGVTQ-QSLLEEAIDQLTENAN---------
646129274_AplaP_010100007922	IVDVPAWSDHNDALTLEPL-TDGMVLVARPTCTM-SAQMNQLADQLTETDEEQTGPKKYR
650387413_NIES39_K02750	IVDVPAWSDHNDALTLEPL-TDGMVLVARPTCTM-SAQMNQLADQLTETDEEQTGPKKYR
643168837_AmaxDRAFT_1210	IVDVPAWSDHNDALTLEPL-TDGMVLVARANCTM-SAQMNQLADQLTETDEEQTGPKKYR
648390350_APCC8_010100026493	IVDVPAWSDHNDALTLEPL-TDGMVLVARANCTM-SAQMNQLADQLTETDEEQTGPKKYR
640015518_L8106_06195	IIDVPSLSANNDALTLEPL-TDGMILVARPSYTL-SGQLGEFAEQLTEIDEDEDSTK-YR
648859640_OSCI_3640012	VIDSPPLNEYSDALTLEPY-TDGIVLVTRQGHTN-SETLTEVSDRLTEF-DEEL--NKVG
2508873356_Oscil6407DRAFT_00014730	VIDSPPLNEYSDALTLEPY-TDGIVLVTRQGHTN-SETLTEVSDRLTEF-DEEL--NKVG
2504092741_Osc7112_6177	VVDSPALSECNDALTLEPY-TDGMILVARPGYTL-SSMLSEAADQLLESEEDES--HKSG
2506346135_MicvaDRAFT_1862	VVDSPALSECNDALTLEPY-TDGMILVARPGYTL-SSMLSEAADQLLESEDEES--NKSG
638107751_Tery_2688	ILDTSCLNQNNDAFILEPY-TDGMIIITRPYYTT-SGILTEIIEQLTNDEEDEESTS--G
2506480833_LYNGBM3L_31450	VVDSPSLSRCNDALLLQPF-TDGIVLVTRPGYTQ-GSILSQVIEELDENEV---------
647565613_MC7420_2957	VIDSPALSLSNDTLLLEPL-ADGMVLVTRPGYSE-KSILNLAAQELTETEPS--------
2510102323_Gei7105DRAFT_3578	VVDAPSLSQCNDALLLEPY-TDGMILVARPGVTE-GGLLAEYAEMLEEAED--------R
2509498590_Pro9006DRAFT_0077	IIDTPSFLNHNDALLLEPL-VDGLLLVLRPGYSR-PSQLKAVLAQM-ETE---------K
2509773034_Lepto7104DRAFT_0253	LIDAPPLARSNDALLLGAA-SDGLLLITRPGITD-KAVLEALLEQLLENED---------
2517693907_LEP6406DRAFT_3365	ILDAPHLTGSNDTMLLQGR-TDGIVLVTRPQLTE-KPVLQTVLEQFEESED---------
2509844778_Lepto7375DRAFT_4795	ILDTPSLSRVDDALLLEEQ-TDGMILVARPGVTE-RAVLNTALEELDLNED---------
641676481_cce_1994	IIDTPSLSRCNDALLLEPL-TDGLVLVTRPGTTR-SSLLNEAIDQLSDAE----------
2507502153_Cy51472_3013	IIDTPSLSRCNDALLLEPL-TDGLVLVTRPGTTR-SSLLNEAIDQLSDAE----------
640626984_CY0110_27525	IIDTPSLSRCNDALLLEPF-TDGLVLVTRPGVTR-SSLLNEAIDQLSDAE----------
2503335332_CWat_WH8501_draft2_00062360	IIDSPSLSRCNDALLLESL-TDGLVLITRPGFTR-SSLLNEAIDQLSDAE----------
2531849533_CWATWH0003_2441	IIDSPSLSRCNDALLLESL-TDGLVLITRPGFTR-SSLLNEAIDQLSDAE----------
643475556_PCC8801_2514	IIDSPSLSRCNDALLLEPL-TDGLVLVTRPGYTR-SSLLNETIDQFTEAE----------
644981523_Cyan8802_3592	IIDSPSLSRCNDALLLEPL-TDGLVLVTRPGYTR-SSLLNETIDQFTEAE----------
2509576278_Ple7327_4396	IVDTPSLSRCNDALLLEPL-TDGLILVTRPGLTR-SSLLNEALDQFTEAE----------
641537721_MAE_37160	IVDTPSLSSCNDALLLEEL-ADGIILVTRQAITR-SSLLSEATDQLIEAE----------
648188704_Cyan7822_3790	VIDTPSLSKCNDALLLEPL-TDGLILVTRPGITR-SSFLSEAIEQLAEAE----------
2503801406_Sta7437_3306	IIDSPSLSKYNDALLIESL-TDGIVLVTRPGIIQ-SSMLGETIDQFTETD----------
2505785267_Chr6712_1514	IIDTPSLSKCNDALLLESL-TDGVVLIARPGISR-GSMLGETIDQFTETD----------
2506598905_Spi9445_1800	IVDSPALTTCNDALLLQPQ-TDGMILVTGVGVSQ-AKILGAMLDEMTEAE----------
2506609325_Spi6313_1684	ILDTPPLDHCNDALLLQPL-TDGMVITTRPGVTQ-KKPLGMMLEQLLDAE----------
2503367585_Cyast_2162	VIDTPSLSSCNDALLLEPF-VDGIVLVTHPGKSL-RNLLGTTIDDFIEEE----------
gi|16124419|ref|NP_418983.1|	LLDTAPLLAIADTRILAPH-TDAVVMLVRWKKTPVKAV--QSALALLQGTRAF-------
gi|16264834|ref|NP_437626.1|	VVDLAALAPVVDAKAFAPL-ADGILFVVEWGRTPSR-----LVRDLLHSEPL------IN
2508652600_Xen7305DRAFT_00047000	LLDTSAVDLSTDILNISQF-TDGIILVGRIGFTNPHKLL-EAQELIKAS----------N

```
2506480821_LYNGBM3L_31350             LIDSAPVSMTSETALMAAV-IGNLLFVVRPE-ISKRDFVSDSLEQLA----------QHN
2509432410_Mic7113_0575               LIDSAPVSLTSETALMANV-VPNVLFVVRPG-TSYSNSVNESLDQLA----------QHQ
2507482189_Cal7103DRAFT_00090960      IVDSAPVSFTSETPLIANI-IRNVLFVVRPD-ISYRDSVNESFAQLT----------QHN
2509801296_LepboDRAFT_0638            LVDSAPVSSTSETALMAAI-VPYVLFVVRPG-ISARNVVNNSLEQLT----------QHH
2505802696_Cal7507_4407               LVDTAPLSNTTATALMTAQ-IPNVLFVVRQG-ISYSNCVRDSLEQLA----------QHQ
2507332342_Tol9009DRAFT_00023250      LVDTAPVSSTTATALMTAQ-IPNVLFVVRPG-ISHSSLVRDSLEQLI----------QHQ
2510087202_Riv7116_2056               LVDTAPVSATTATALMTAQ-IPNVLFVVKPG-MSFSSSVRDSLQQLM----------EHQ
2510089901_Riv7116_4755               LVDSSPVALTPEAALLARI-ISKVVFVVRST-SSHRNYFYDSVEQLT----------RHQ
2506745681_Syn7336_0399               LVDSAPVGLTSEATLMARA-IGNVLMVVRPG-TSNRDPFCNSIEQLQ----------RHG
637457826_glr0454                     LIDTAPEDLVAETKLIAAA-IRNVLFVVRSG-VSNRDSVNSSLYTLQ----------RFG
637459188_gll1796                     LIDTAPEDLVAETKLIAAA-IGNVLFVVRPG-VSNRDEVNASLAALQ----------RFG
637461130_gll3716                     LVDSPPISLTSEAALMIPA-VRNVLFVVRPG-VSGRDAVSESLDRLV----------RHN
2503610992_Chro_0741                  LVDSPPVGLASETNLMSAV-VCNVLFVLRSG-TSDRYPVMNSLEQLT----------RYN
2503793842_Glo7428_1287               LVDSPPVGLASETNLMSSV-IQNVLFVVRAG-KSDRFSVMDSFEQLV----------RHN
2505800320_Cal7507_2052               IVDSAPVGCTSEAALMASA-IANVLLVIQLG-VSDRHMVQESMEQLI----------RHH
643588131_Cyan7425_5174               LVDTAPITLTSETGLMTTS-VNNLLFVVRPG-TSSRYSVLDSFEELS----------LRK
643167924_AmaxDRAFT_0301              IVDMPPGTGDAQLTLAQAVPMSGAVIVTTPQEV-----------SLLDSRKGLKMFEQLG
648389440_APCC8_010100021805          IVDMPPGTGDAQLTLAQAVPMSGAVIVTTPQEV-----------SLLDSRKGLKMFEQLG
646130866_AplaP_010100015993          IVDMPPGTGDAQLTLAQAVPMSGAVIVTTPQEV-----------SLLDSRKGLKMFEQLG
650384994_NIES39_D01080               IVDMPPGTGDAQLTLAQAVPMSGAVIVTTPQEV-----------SLLDSRKGLKMFEQLG
640015631_L8106_17697                 IVDMPPGTGDAQLTLVQAVPMSGVVIVTTPQTV-----------SLLDSRKGLRMFQQLG
647567948_MC7420_2316                 IVDMPPGTGDAQLTLAQAVPMAGAIIVTTPQNV-----------ALLDSRRGLKMFQQLG
638106387_Tery_1165                   LVDLPPGTGDAQLTLAQAVPMSGVVIVTTPQTV-----------ALLDSRKGLKMFQQLG
648857672_OSCI_2870002                IVDMPPGTGDAQLTMAQAVPMAGAVIVTTPQTV-----------ALLDSRKGLKMFQQLG
2508875395_Oscil6407DRAFT_00035160    IVDMPPGTGDAQLTMAQAVPMAGAVIVTTPQTV-----------ALLDSRKGLKMFQQLG
2509801138_LepboDRAFT_0480            IVDMPPGTGDAQLTMAQAVPMAGAVIVTTPQTV-----------ALLDSRRGLKMFQQLN
2512979571_Fis9431DRAFT_3900          LVDMPPGTGDAQLTLTQAVPMAGAVIVTTPQTV-----------ALLDSRKGLRMFQQMN
2517061293_PCC9339DRAFT_02148         LVDMPPGTGDAQLTLTQAVPMAGAVIVTTPQTV-----------ALLDSRKGLRMFQQMN
2505768399_FJSC11DRAFT_3149           LVDMPPGTGDAQLTLTQAVPMAGAVIVTTPQTV-----------ALLDSRKGLRMFQQMN
2509782632_Mic7126DRAFT_3383          VVDMPPGTGDAQLTLTQAVPMAGAVIVTTPQTV-----------ALLDSRKGLRMFQQMN
2504683355_Cri9333_0934               IVDMPPGTGDAQLTLAQAVPMAGAVIVTTPQTV-----------ALLDSRKGLKMFQQLG
2503606101_GEI7407_0138               IVDLPPGTGDAQLTLAQAVPMAGAVIVTTPQTV-----------ALIDSRRGLKMFQQMG
2509502622_Pro9006DRAFT_4121          LVDLPPGTGDAQLTLAQAVPMAGAVIVTTPQTV-----------ALLDARKGLNMFRQLG
2509778293_Lepto7104DRAFT_5514        VVDLPPGTGDAQLTLAQAVPMAGAVIVSTPQSV-----------AISDARRGLKMFQQLQ
2517694920_LEP6406DRAFT_4378          VVDMPPGTGDAQLTLAQAVPMAGAVIVTTPQSV-----------ALSDARRGLRMFQQLQ
647577822_S7335_3227                  VVDMPPGTGDAQLTLAQAVPMAGAVIVTTPQDV-----------ALSDARRGLKMFEQLK
2503637469_PCC7418_2732               VVDLPPGTGDAQLTLAQAVPMSGAVIVTTPQDV-----------SLMDARRGLKMFEQLN
2509552467_Dacsa_0081                 VVDLPPGTGDAQLTLAQAVPMSGAVIVTTPQDV-----------SLMDARRGLKMFEQLG
2503367528_Cyast_2106                 IVDMPPGTGDAQLTLAQAVPLAGAVIVTTPQTV-----------SLQDARRGLKMFEQLG
2503887475_Lepto7376_1861             LVDMPPGTGDAQLTMAQAVPMAGAIIVTTPQTV-----------SLLDSRRGLKMFQQMD
637688026_PMN2A_1153                  IVDLPPGTGDAQLSLAQAVPMAGVIIVTTPQNV-----------SLQDSRRGLAMFKQMN
640085320_NATL1_20271                 IVDLPPGTGDAQLSLAQAVPMAGVIIVTTPQNV-----------SLQDSRRGLAMFKQMN
641286083_P9211_17011                 VVDMPPGTGDAQLSLAQAVPITGVLVVTTPQKV-----------SLQDARRGLAMFKQMD
640547039_SynRCC307_2084              VVDLPPGTGDAQLSLAQAVPMAGVVVVTTPQQV-----------ALQDARRGLAMFQQMG
637798215_PMT9312_1673                VIDLPPGTGDAQISLSQSVPISGAIVVTTPQQV-----------SLQDARRGLAMFKQLG
640161373_P9301_17721                 VIDLPPGTGDAQISLTQSVPISGAIVVTTPQQV-----------SLQDARRGLAMFKQLG
640944474_P9215_18531                 VIDLPPGTGDAQISLSQSVPISGAIVVTTPQQV-----------SLQDARRGLAMFKQLG
647672827_P9202_63                    VIDLPPGTGDAQISLSQSVPISGAIVVTTPQQV-----------SLQDARRGLAMFKQLG
640079518_A9601_17881                 VIDLPPGTGDAQISLSQSVPITGAIVVTTPQQV-----------SLQDARRGLAMFKQLG
637450610_PMM1581                     VIDLPPGTGDAQISLSQSVPISGAIVVTTPQKV-----------SLQDARRGLAMFKQLG
640081464_P9515_17681                 VIDLPPGTGDAQISLSQSVPISGAIVVTTPQQV-----------SLQDARRGLAMFKQLG
646569576_Ava_4074                    LIDLPPGTGDAQITIIQESPICGVILVTTPQQV-----------AVADVRRNIYMFRQVG
2505803290_Cal7507_4972               LIDLPPGTGDAQITIIQESPVCGVILVTTPQQV-----------AVADVRRNIYMFRQVG
2517700475_SYN7509DRAFT_4594          LIDLPPGTGDAQITIVQESPICGVLLVTTPQQV-----------AVSDVRRSIHMFRRVG
2506747473_Syn7336_2171               LIDLPPGTGDAQITIVQESPIAGAVLVTTPQQV-----------ALGDVRRSIHMFRNVG
gi|1172092|gb|AAA86371.1|             ------------------------------------------------------------
gi|15597431|ref|NP_250925.1|          LLDAPALLDSREAFIALRR-ADLIALVVEAQKSTV-PVVEHALTILTTAFG---------
637450246_PMM1225                     FINGSSINNSPQSVINGNL-SDLTTILISTNNLK-KSDLVKSVDKLSKAGSNIDAY----
gi|53721457|ref|YP_110442.1|          ------------------------------------------------------------
638961258_RS9917_04770                LVVSRDLLAT-------RA-CSTQLLLTAPGAAKREQLRQLREQLALQGS----------
639888778_RS9916_38577                IILNQNLLET-------RA-CSTQVIITALGAAKRDDIYRLREELILQGG----------
637444523_SYNW0419                    LLVSRDLLAS-------RT-CSTQLLVTAPGAPQRQQLQQLREQLALQGT----------
640545089_SynRCC307_0169              ------------------------------GRGRRGQ-----------------------
640084152_NATL1_08771                 IIITKDLREA-------VQ-CPNLIVITALGITRNQELIEARQKLLLQRT----------
647672433_P9202_965                   -FFENNLSNI-------DS-SEKIIFIAKLGELKTDEIYNLKERLIFTGK----------
637797888_PMT9312_1355                IFIT------------------SLKNIFKKDVIKIRNKI--TLNKL--------------
640944066_P9215_14561                 I----------ETSLEKSF-ESNEILVI----IRSESITHKMIEDFDENAYI------IN
gi|397405|emb|CAA52655.1|             ------------------------------------------------------------
gi|455023|gb|AAB51624.1|              ------------------------------------------------------------
gi|1107924|emb|CAA62141.1|            ------------------------------------------------------------
gi|53720415|ref|YP_109401.1|          ------------ACFILGLAAWGVLSMLVAGIREHQE-----------------------
gi|45297|emb|CAA38731.1|              ------------ATFIIGLMLYGVLNLLIASIREHKN-----------------------
```

References

1 Shih, P. M. *et al.* Improving the coverage of the cyanobacterial phylum using diversity-driven genome sequencing. *Proc. Natl. Acad. Sci. U. S. A.* **110**, 1053-1058, doi:10.1073/pnas.1217107110 (2013).

2 Dagan, T. *et al.* Genomes of Stigonematalean Cyanobacteria (Subsection V) and the Evolution of Oxygenic Photosynthesis from Prokaryotes to Plastids. *Genome Biol. Evol.* **5**, 31-44, doi:10.1093/gbe/evs117 (2013).

3 Calteau, A. *et al.* Phylum-wide comparative genomics unravel the diversity of secondary metabolism in Cyanobacteria. *BMC Genomics* **15**, 977, doi:10.1186/1471-2164-15-977 (2014).

4 Cuthbertson, L., Mainprize, I. L., Naismith, J. H. & Whitfield, C. Pivotal Roles of the Outer Membrane Polysaccharide Export and Polysaccharide Copolymerase Protein Families in Export of Extracellular Polysaccharides in Gram-Negative Bacteria. *Microbiol. Mol. Biol. Rev.* **73**, 155-177, doi:10.1128/MMBR.00024-08 (2009).

5 Morona, R., Van Den Bosch, L. & Daniels, C. Evaluation of Wzz/MPA1/MPA2 proteins based on the presence of coiled-coil regions. *Microbiology* **146**, 1-4 (2000).

6 Reid, A. N. & Whitfield, C. Functional analysis of conserved gene products involved in assembly of *Escherichia coli* capsules and exopolysaccharides: evidence for molecular recognition between Wza and Wzc for colanic acid biosynthesis. *J. Bacteriol.* **187**, 5470-5481, doi:10.1128/JB.187.15.5470-5481.2005 (2005).

7 Soulat, D. *et al.* Tyrosine-kinase Wzc from *Escherichia coli* possesses an ATPase activity regulated by autophosphorylation. *FEMS Microbiol. Lett.* **274**, 252-259, doi:10.1111/j.1574-6968.2007.00841.x (2007).

8 Schneider, E. & Hunke, S. ATP-binding-cassette (ABC) transport systems: functional and structural aspects of the ATP-hydrolyzing subunits/domains. *FEMS Microbiol. Rev.* **22**, 1-20, doi:S0168-6445(98)00002-3 (1998).

9 Ferreira, A. S. *et al.* Functional analysis of *Burkholderia cepacia* genes *bceD* and *bceF*, encoding a phosphotyrosine phosphatase and a tyrosine autokinase, respectively: role in exopolysaccharide biosynthesis and biofilm formation. *Appl. Environ. Microbiol.* **73**, 524-534, doi:10.1128/AEM.01450-06 (2007).

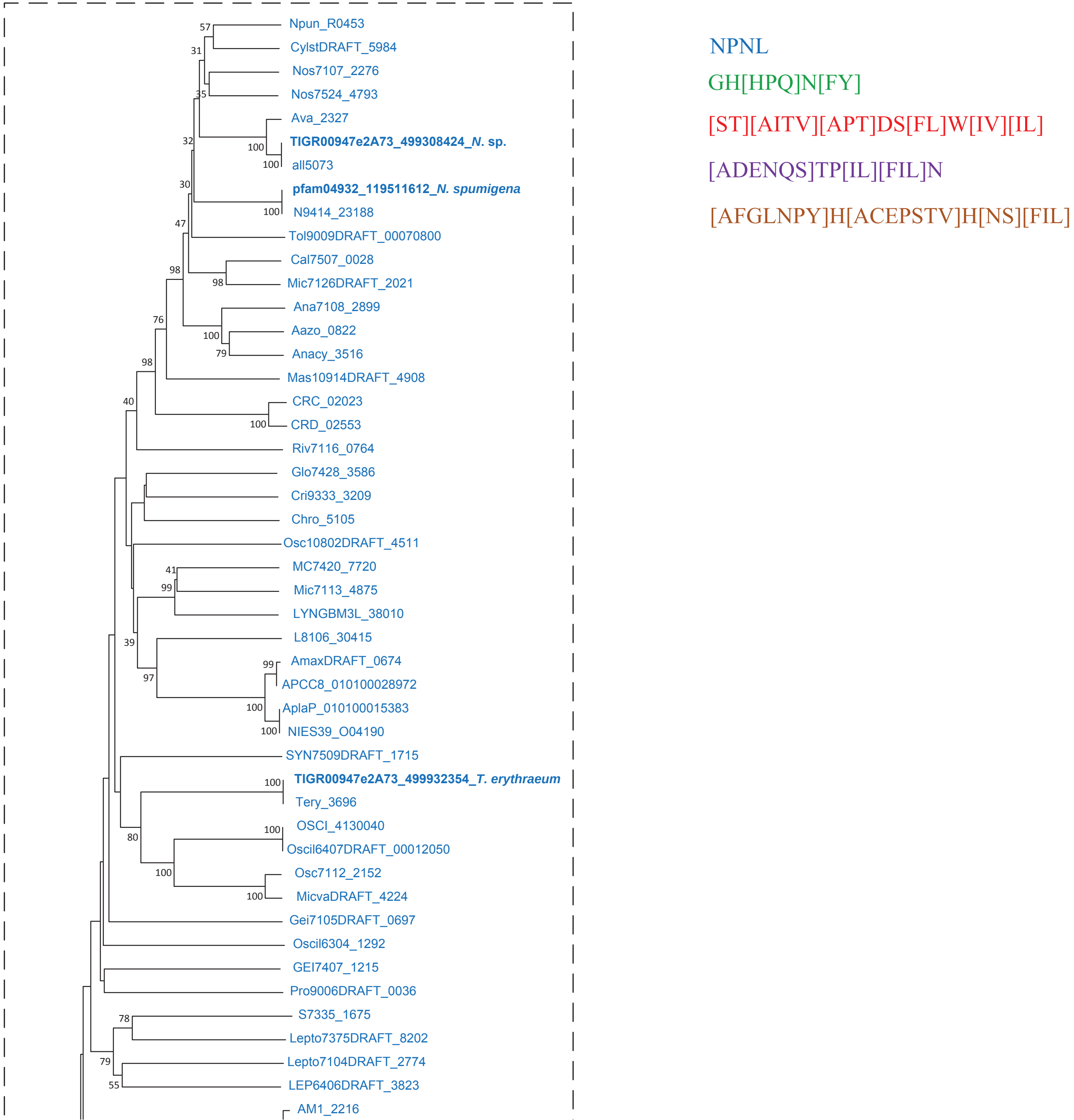
Npun_R0453
CylstDRAFT_5984
Nos7107_2276
Nos7524_4793
Ava_2327
TIGR00947e2A73_499308424_N. sp.
all5073
pfam04932_119511612_N. spumigena
N9414_23188
Tol9009DRAFT_00070800
Cal7507_0028
Mic7126DRAFT_2021
Ana7108_2899
Aazo_0822
Anacy_3516
Mas10914DRAFT_4908
CRC_02023
CRD_02553
Riv7116_0764
Glo7428_3586
Cri9333_3209
Chro_5105
Osc10802DRAFT_4511
MC7420_7720
Mic7113_4875
LYNGBM3L_38010
L8106_30415
AmaxDRAFT_0674
APCC8_010100028972
AplaP_010100015383
NIES39_O04190
SYN7509DRAFT_1715
TIGR00947e2A73_499932354_T. erythraeum
Tery_3696
OSCI_4130040
Oscil6407DRAFT_00012050
Osc7112_2152
MicvaDRAFT_4224
Gei7105DRAFT_0697
Oscil6304_1292
GEI7407_1215
Pro9006DRAFT_0036
S7335_1675
Lepto7375DRAFT_8202
Lepto7104DRAFT_2774
LEP6406DRAFT_3823
AM1_2216
NPNL
GH[HPQ]N[FY]
[ST][AITV][APT]DS[FL]W[IV][IL]
[ADENQS]TP[IL][FIL]N
[AFGLNPY]H[ACEPSTV]H[NS][FIL]

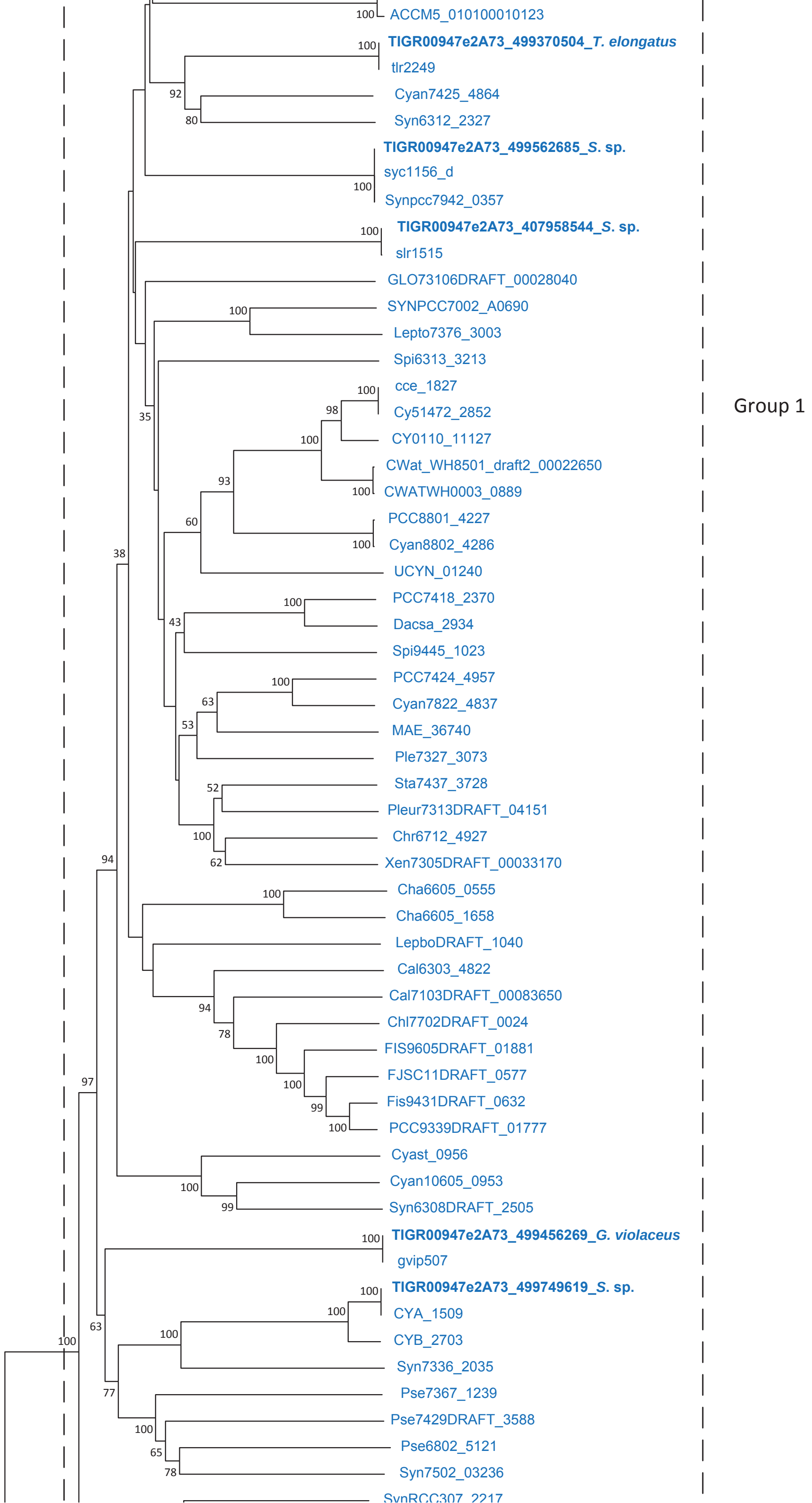
ACCM5_010100010123
TIGR00947e2A73_499370504_T. elongatus
tlr2249
Cyan7425_4864
Syn6312_2327
TIGR00947e2A73_499562685_S. sp.
syc1156_d
Synpcc7942_0357
TIGR00947e2A73_407958544_S. sp.
slr1515
GLO73106DRAFT_00028040
SYNPCC7002_A0690
Lepto7376_3003
Spi6313_3213
cce_1827
Cy51472_2852
CY0110_11127
CWat_WH8501_draft2_00022650
CWATWH0003_0889
PCC8801_4227
Cyan8802_4286
UCYN_01240
PCC7418_2370
Dacsa_2934
Spi9445_1023
PCC7424_4957
Cyan7822_4837
MAE_36740
Ple7327_3073
Sta7437_3728
Pleur7313DRAFT_04151
Chr6712_4927
Xen7305DRAFT_00033170
Cha6605_0555
Cha6605_1658
LepboDRAFT_1040
Cal6303_4822
Cal7103DRAFT_00083650
Chl7702DRAFT_0024
FIS9605DRAFT_01881
FJSC11DRAFT_0577
Fis9431DRAFT_0632
PCC9339DRAFT_01777
Cyast_0956
Cyan10605_0953
Syn6308DRAFT_2505
TIGR00947e2A73_499456269_G. violaceus
gvip507
TIGR00947e2A73_497749619_S. sp.
CYA_1509
CYB_2703
Syn7336_2035
Pse7367_1239
Pse7429DRAFT_3588
Pse6802_5121
Syn7502_03236
SynRCC307_2217
Group 1

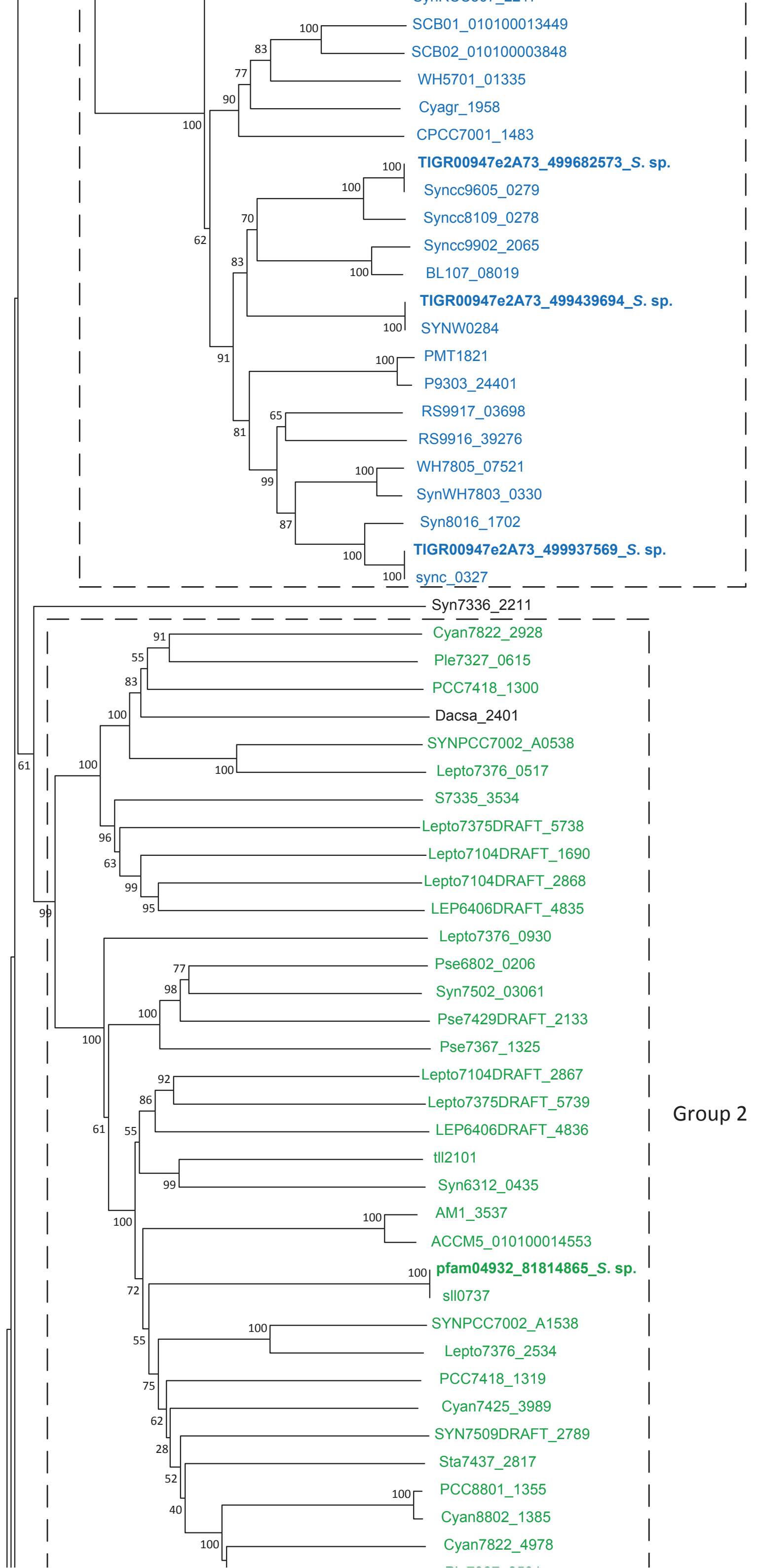
SCB01_010100013449
SCB02_010100003848
WH5701_01335
Cyagr_1958
CPCC7001_1483
TIGR00947e2A73_499682573_S. sp.
Syncc9605_0279
Syncc8109_0278
Syncc9902_2065
BL107_08019
TIGR00947e2A73_499439694_S. sp.
SYNW0284
PMT1821
P9303_24401
RS9917_03698
RS9916_39276
WH7805_07521
SynWH7803_0330
Syn8016_1702
TIGR00947e2A73_499937569_S. sp.
sync_0327
Syn7336_2211
Cyan7822_2928
Ple7327_0615
PCC7418_1300
Dacsa_2401
SYNPCC7002_A0538
Lepto7376_0517
S7335_3534
Lepto7375DRAFT_5738
Lepto7104DRAFT_1690
Lepto7104DRAFT_2868
LEP6406DRAFT_4835
Lepto7376_0930
Pse6802_0206
Syn7502_03061
Pse7429DRAFT_2133
Pse7367_1325
Lepto7104DRAFT_2867
Lepto7375DRAFT_5739
LEP6406DRAFT_4836
tll2101
Syn6312_0435
AM1_3537
ACCM5_010100014553
pfam04932_81814865_S. sp.
sll0737
SYNPCC7002_A1538
Lepto7376_2534
PCC7418_1319
Cyan7425_3989
SYN7509DRAFT_2789
Sta7437_2817
PCC8801_1355
Cyan8802_1385
Cyan7822_4978
Group 2

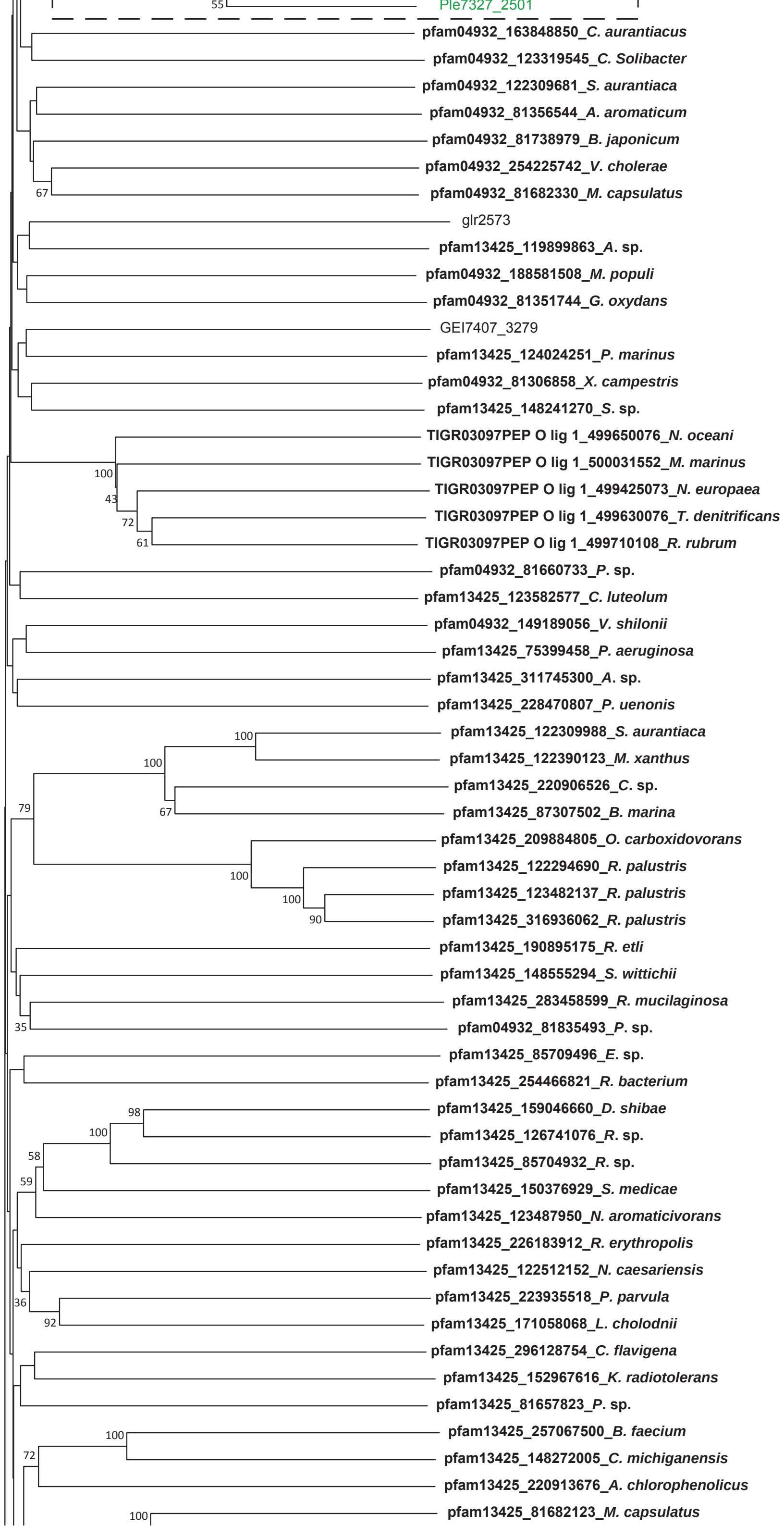
Ple7327_2501
pfam04932_163848850_C. aurantiacus
pfam04932_123319545_C. Solibacter
pfam04932_122309681_S. aurantiaca
pfam04932_81356544_A. aromaticum
pfam04932_81738979_B. japonicum
pfam04932_254225742_V. cholerae
pfam04932_81682330_M. capsulatus
glr2573
pfam13425_119899863_A. sp.
pfam04932_188581508_M. populi
pfam04932_81351744_G. oxydans
GEI7407_3279
pfam13425_124024251_P. marinus
pfam04932_81306858_X. campestris
pfam13425_148241270_S. sp.
TIGR03097PEP O lig 1_499650076_N. oceani
TIGR03097PEP O lig 1_500031552_M. marinus
TIGR03097PEP O lig 1_499425073_N. europaea
TIGR03097PEP O lig 1_499630076_T. denitrificans
TIGR03097PEP O lig 1_499710108_R. rubrum
pfam04932_81660733_P. sp.
pfam13425_123582577_C. luteolum
pfam04932_149189056_V. shilonii
pfam13425_75399458_P. aeruginosa
pfam13425_311745300_A. sp.
pfam13425_228470807_P. uenonis
pfam13425_122309988_S. aurantiaca
pfam13425_122390123_M. xanthus
pfam13425_220906526_C. sp.
pfam13425_87307502_B. marina
pfam13425_209884805_O. carboxidovorans
pfam13425_122294690_R. palustris
pfam13425_123482137_R. palustris
pfam13425_316936062_R. palustris
pfam13425_190895175_R. etli
pfam13425_148555294_S. wittichii
pfam13425_283458599_R. mucilaginosa
pfam04932_81835493_P. sp.
pfam13425_85709496_E. sp.
pfam13425_254466821_R. bacterium
pfam13425_159046660_D. shibae
pfam13425_126741076_R. sp.
pfam13425_85704932_R. sp.
pfam13425_150376929_S. medicae
pfam13425_123487950_N. aromaticivorans
pfam13425_226183912_R. erythropolis
pfam13425_122512152_N. caesariensis
pfam13425_223935518_P. parvula
pfam13425_171058068_L. cholodnii
pfam13425_296128754_C. flavigena
pfam13425_152967616_K. radiotolerans
pfam13425_81657823_P. sp.
pfam13425_257067500_B. faecium
pfam13425_148272005_C. michiganensis
pfam13425_220913676_A. chlorophenolicus
pfam13425_81682123_M. capsulatus

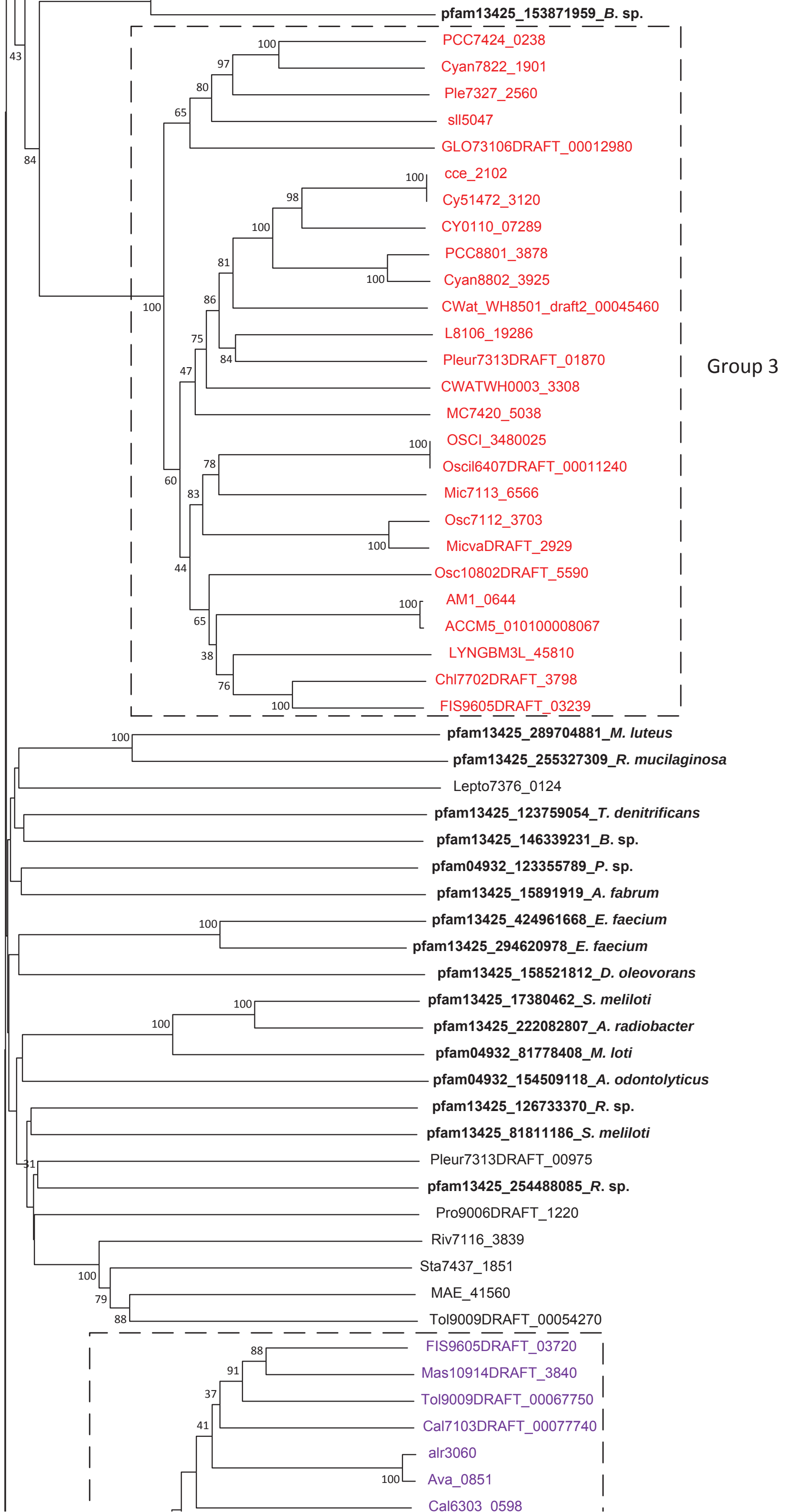

pfam13425_153871959_B. sp.
PCC7424_0238
Cyan7822_1901
Ple7327_2560
sll5047
GLO73106DRAFT_00012980
cce_2102
Cy51472_3120
CY0110_07289
PCC8801_3878
Cyan8802_3925
CWat_WH8501_draft2_00045460
L8106_19286
Pleur7313DRAFT_01870
CWATWH0003_3308
MC7420_5038
OSCI_3480025
Oscil6407DRAFT_00011240
Mic7113_6566
Osc7112_3703
MicvaDRAFT_2929
Osc10802DRAFT_5590
AM1_0644
ACCM5_010100008067
LYNGBM3L_45810
Chl7702DRAFT_3798
FIS9605DRAFT_03239
Group 3
pfam13425_289704881_M. luteus
pfam13425_255327309_R. mucilaginosa
Lepto7376_0124
pfam13425_123759054_T. denitrificans
pfam13425_146339231_B. sp.
pfam04932_123355789_P. sp.
pfam13425_15891919_A. fabrum
pfam13425_424961668_E. faecium
pfam13425_294620978_E. faecium
pfam13425_158521812_D. oleovorans
pfam13425_17380462_S. meliloti
pfam13425_222082807_A. radiobacter
pfam04932_81778408_M. loti
pfam04932_154509118_A. odontolyticus
pfam13425_126733370_R. sp.
pfam13425_81811186_S. meliloti
Pleur7313DRAFT_00975
pfam13425_254488085_R. sp.
Pro9006DRAFT_1220
Riv7116_3839
Sta7437_1851
MAE_41560
Tol9009DRAFT_00054270
FIS9605DRAFT_03720
Mas10914DRAFT_3840
Tol9009DRAFT_00067750
Cal7103DRAFT_00077740
alr3060
Ava_0851
Cal6303_0598

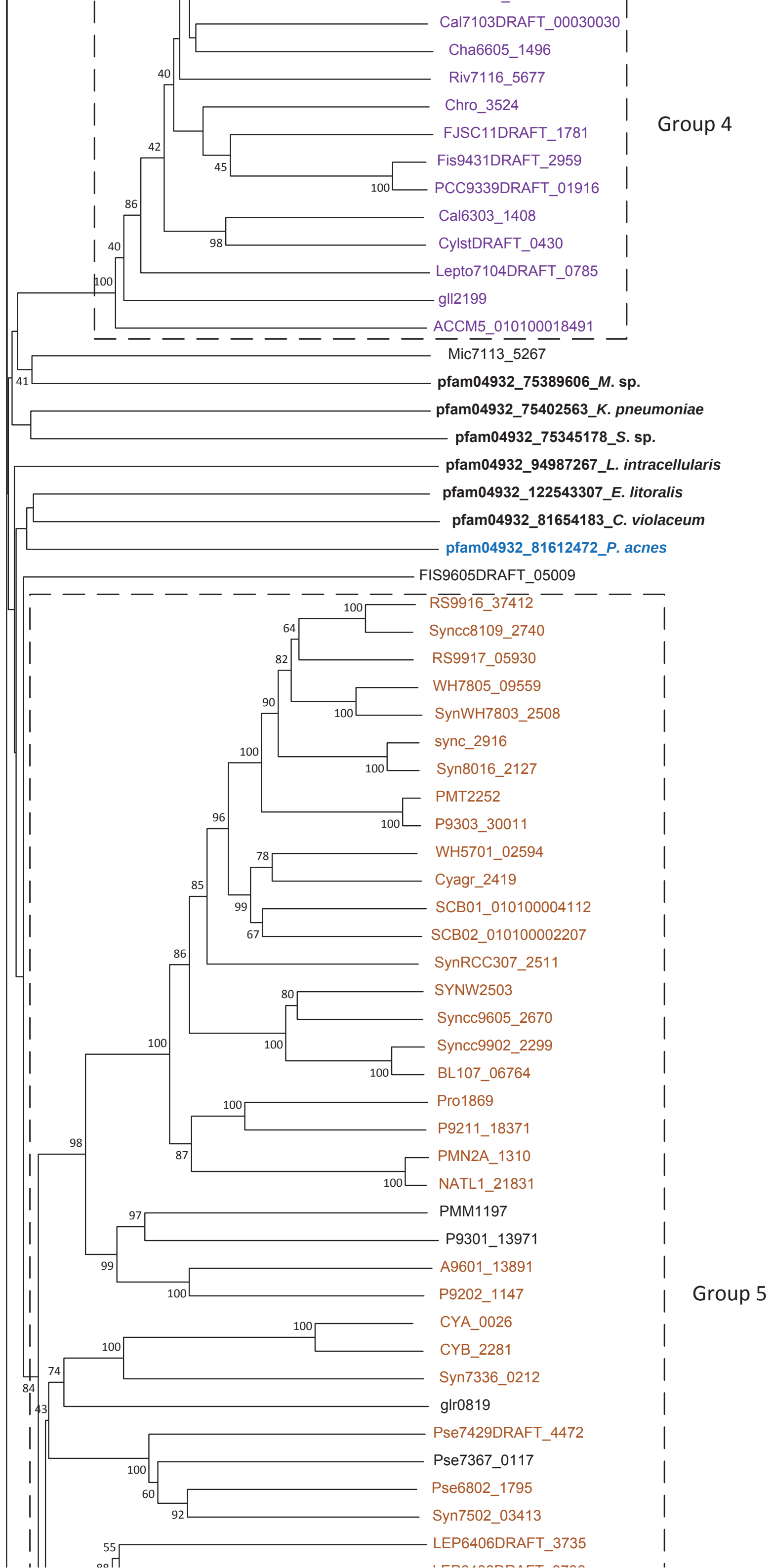

Cal7103DRAFT_00030030
Cha6605_1496
Riv7116_5677
Chro_3524
FJSC11DRAFT_1781
Fis9431DRAFT_2959
PCC9339DRAFT_01916
Cal6303_1408
CylstDRAFT_0430
Lepto7104DRAFT_0785
gll2199
ACCM5_010100018491
Group 4
Mic7113_5267
pfam04932_75389606_M. sp.
pfam04932_75402563_K. pneumoniae
pfam04932_75345178_S. sp.
pfam04932_94987267_L. intracellularis
pfam04932_122543307_E. litoralis
pfam04932_81654183_C. violaceum
pfam04932_81612472_P. acnes
FIS9605DRAFT_05009
RS9916_37412
Syncc8109_2740
RS9917_05930
WH7805_09559
SynWH7803_2508
sync_2916
Syn8016_2127
PMT2252
P9303_30011
WH5701_02594
Cyagr_2419
SCB01_010100004112
SCB02_010100002207
SynRCC307_2511
SYNW2503
Syncc9605_2670
Syncc9902_2299
BL107_06764
Pro1869
P9211_18371
PMN2A_1310
NATL1_21831
PMM1197
P9301_13971
A9601_13891
P9202_1147
Group 5
CYA_0026
CYB_2281
Syn7336_0212
glr0819
Pse7429DRAFT_4472
Pse7367_0117
Pse6802_1795
Syn7502_03413
LEP6406DRAFT_3735

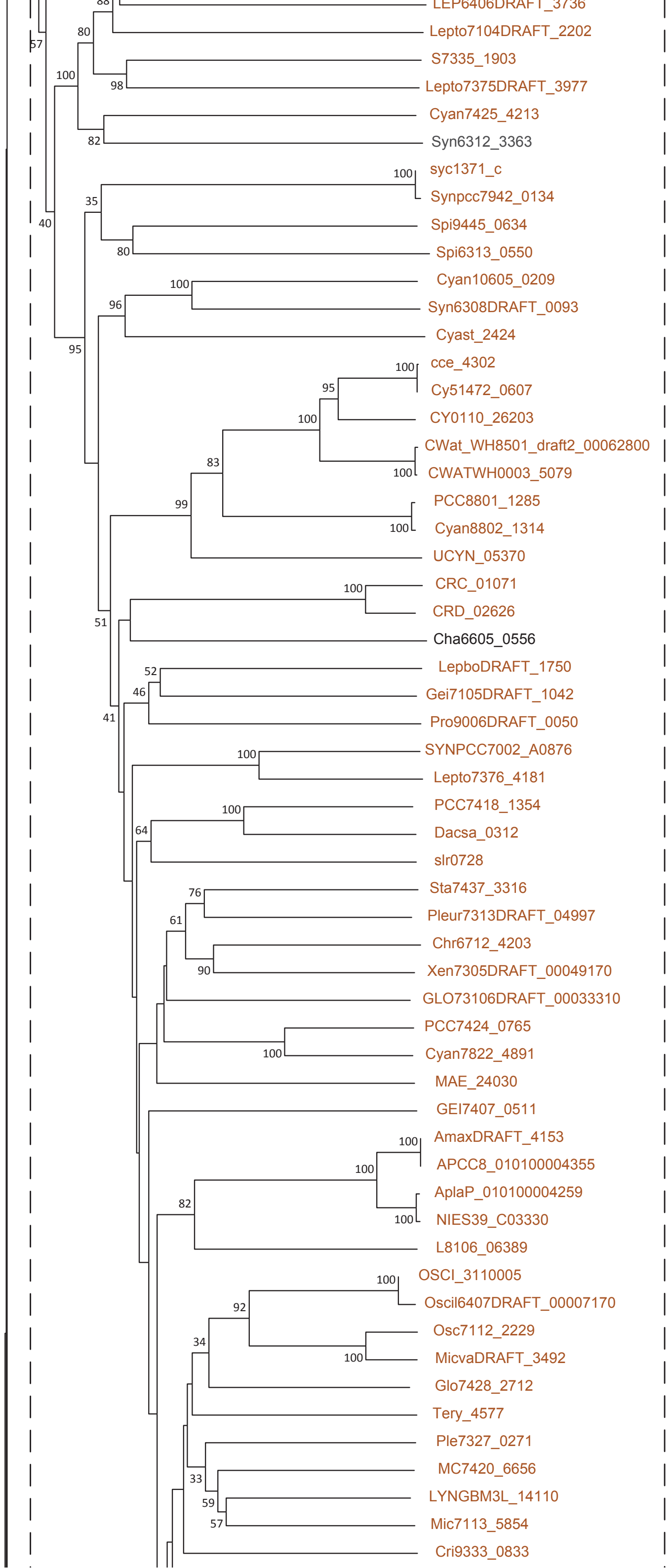

LEP6406DRAFT_3736
Lepto7104DRAFT_2202
S7335_1903
Lepto7375DRAFT_3977
Cyan7425_4213
Syn6312_3363
syc1371_c
Synpcc7942_0134
Spi9445_0634
Spi6313_0550
Cyan10605_0209
Syn6308DRAFT_0093
Cyast_2424
cce_4302
Cy51472_0607
CY0110_26203
CWat_WH8501_draft2_00062800
CWATWH0003_5079
PCC8801_1285
Cyan8802_1314
UCYN_05370
CRC_01071
CRD_02626
Cha6605_0556
LepboDRAFT_1750
Gei7105DRAFT_1042
Pro9006DRAFT_0050
SYNPCC7002_A0876
Lepto7376_4181
PCC7418_1354
Dacsa_0312
slr0728
Sta7437_3316
Pleur7313DRAFT_04997
Chr6712_4203
Xen7305DRAFT_00049170
GLO73106DRAFT_00033310
PCC7424_0765
Cyan7822_4891
MAE_24030
GEI7407_0511
AmaxDRAFT_4153
APCC8_010100004355
AplaP_010100004259
NIES39_C03330
L8106_06389
OSCI_3110005
Oscil6407DRAFT_00007170
Osc7112_2229
MicvaDRAFT_3492
Glo7428_2712
Tery_4577
Ple7327_0271
MC7420_6656
LYNGBM3L_14110
Mic7113_5854
Cri9333_0833

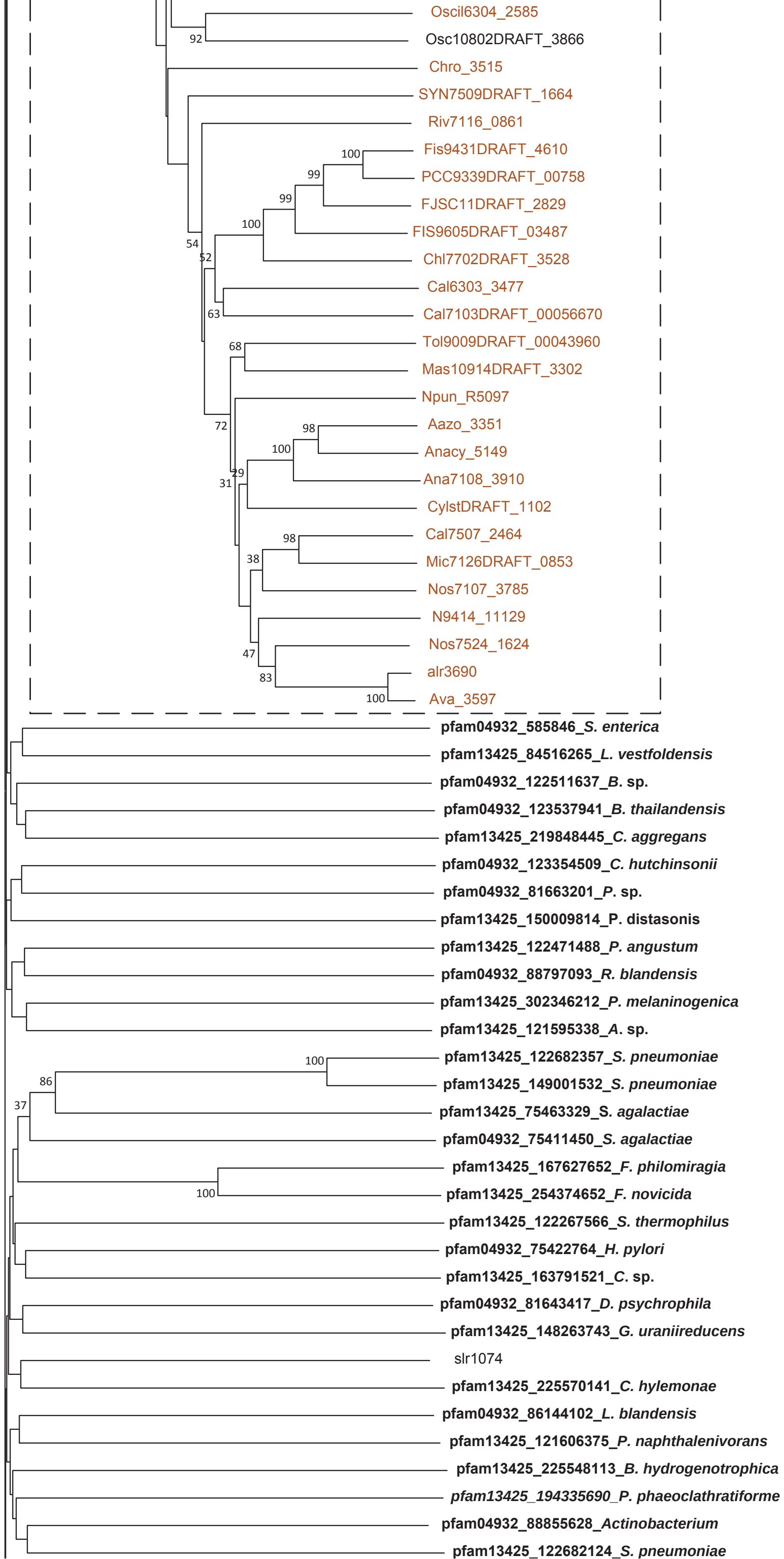

Oscil6304_2585
Osc10802DRAFT_3866
Chro_3515
SYN7509DRAFT_1664
Riv7116_0861
Fis9431DRAFT_4610
PCC9339DRAFT_00758
FJSC11DRAFT_2829
FIS9605DRAFT_03487
Chl7702DRAFT_3528
Cal6303_3477
Cal7103DRAFT_00056670
Tol9009DRAFT_00043960
Mas10914DRAFT_3302
Npun_R5097
Aazo_3351
Anacy_5149
Ana7108_3910
CylstDRAFT_1102
Cal7507_2464
Mic7126DRAFT_0853
Nos7107_3785
N9414_11129
Nos7524_1624
alr3690
Ava_3597
pfam04932_585846_S. enterica
pfam13425_84516265_L. vestfoldensis
pfam04932_122511637_B. sp.
pfam04932_123537941_B. thailandensis
pfam13425_219848445_C. aggregans
pfam04932_123354509_C. hutchinsonii
pfam04932_81663201_P. sp.
pfam13425_150009814_P. distasonis
pfam13425_122471488_P. angustum
pfam04932_88797093_R. blandensis
pfam13425_302346212_P. melaninogenica
pfam13425_121595338_A. sp.
pfam13425_122682357_S. pneumoniae
pfam13425_149001532_S. pneumoniae
pfam13425_75463329_S. agalactiae
pfam04932_75411450_S. agalactiae
pfam13425_167627652_F. philomiragia
pfam13425_254374652_F. novicida
pfam13425_122267566_S. thermophilus
pfam04932_75422764_H. pylori
pfam13425_163791521_C. sp.
pfam04932_81643417_D. psychrophila
pfam13425_148263743_G. uraniireducens
slr1074
pfam13425_225570141_C. hylemonae
pfam04932_86144102_L. blandensis
pfam13425_121606375_P. naphthalenivorans
pfam13425_225548113_B. hydrogenotrophica
pfam13425_194335690_P. phaeoclathratiforme
pfam04932_88855628_Actinobacterium
pfam13425_122682124_S. pneumoniae

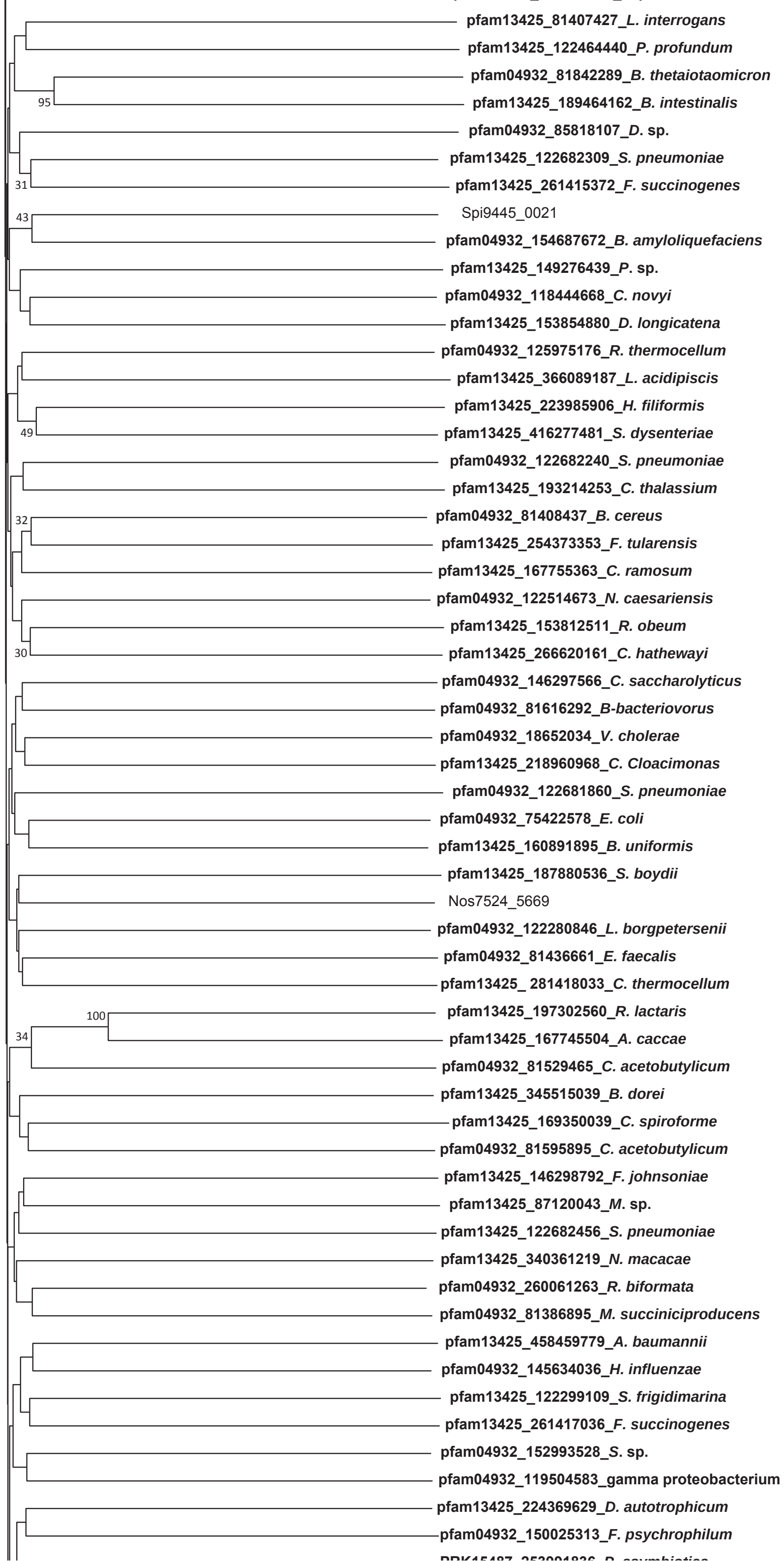
pfam13425_81407427_L. interrogans
pfam13425_122464440_P. profundum
pfam04932_81842289_B. thetaiotaomicron
95
pfam13425_189464162_B. intestinalis
pfam04932_85818107_D. sp.
pfam13425_122682309_S. pneumoniae
31
pfam13425_261415372_F. succinogenes
43
Spi9445_0021
pfam04932_154687672_B. amyloliquefaciens
pfam13425_149276439_P. sp.
pfam04932_118444668_C. novyi
pfam13425_153854880_D. longicatena
pfam04932_125975176_R. thermocellum
pfam13425_366089187_L. acidipiscis
pfam13425_223985906_H. filiformis
49
pfam13425_416277481_S. dysenteriae
pfam04932_122682240_S. pneumoniae
pfam13425_193214253_C. thalassium
32
pfam04932_81408437_B. cereus
pfam13425_254373353_F. tularensis
pfam13425_167755363_C. ramosum
pfam04932_122514673_N. caesariensis
pfam13425_153812511_R. obeum
30
pfam13425_266620161_C. hathewayi
pfam04932_146297566_C. saccharolyticus
pfam04932_81616292_B-bacteriovorus
pfam04932_18652034_V. cholerae
pfam13425_218960968_C. Cloacimonas
pfam04932_122681860_S. pneumoniae
pfam04932_75422578_E. coli
pfam13425_160891895_B. uniformis
pfam13425_187880536_S. boydii
Nos7524_5669
pfam04932_122280846_L. borgpetersenii
pfam04932_81436661_E. faecalis
pfam13425_ 281418033_C. thermocellum
100
pfam13425_197302560_R. lactaris
34
pfam13425_167745504_A. caccae
pfam04932_81529465_C. acetobutylicum
pfam13425_345515039_B. dorei
pfam13425_169350039_C. spiroforme
pfam04932_81595895_C. acetobutylicum
pfam13425_146298792_F. johnsoniae
pfam13425_87120043_M. sp.
pfam13425_122682456_S. pneumoniae
pfam13425_340361219_N. macacae
pfam04932_260061263_R. biformata
pfam04932_81386895_M. succiniciproducens
pfam13425_458459779_A. baumannii
pfam04932_145634036_H. influenzae
pfam13425_122299109_S. frigidimarina
pfam13425_261417036_F. succinogenes
pfam04932_152993528_S. sp.
pfam04932_119504583_gamma proteobacterium
pfam13425_224369629_D. autotrophicum
pfam04932_150025313_F. psychrophilum

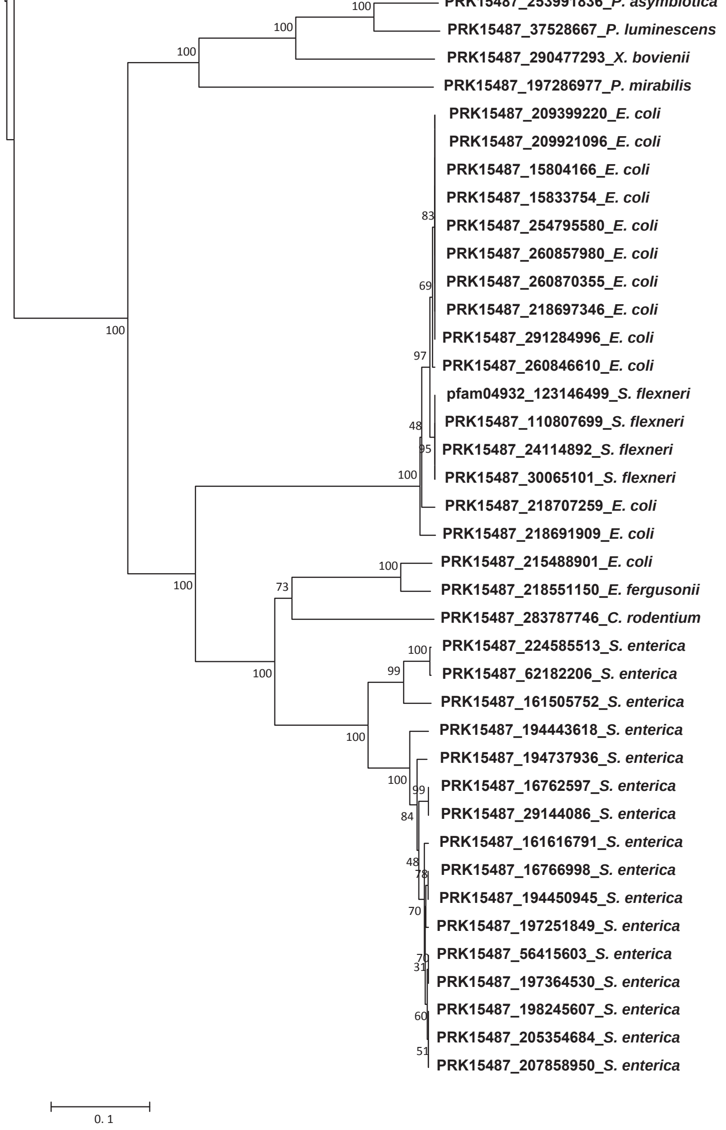

FIG S2 NJ phylogenetic tree of putative Wzy/WaaL. Amino acid patterns are shown in different colors (upper right side). Sequences possessing a given pattern are indicated in the same color as the pattern. Dashed boxes indicate the groups of cyanobacterial sequences (label: locus tag) used to define each pattern. Reference sequences retrieved from the Conserved Domain Database (NCBI; label: conserved domain designation_GI number_ abbreviated species name) are indicated in bold.

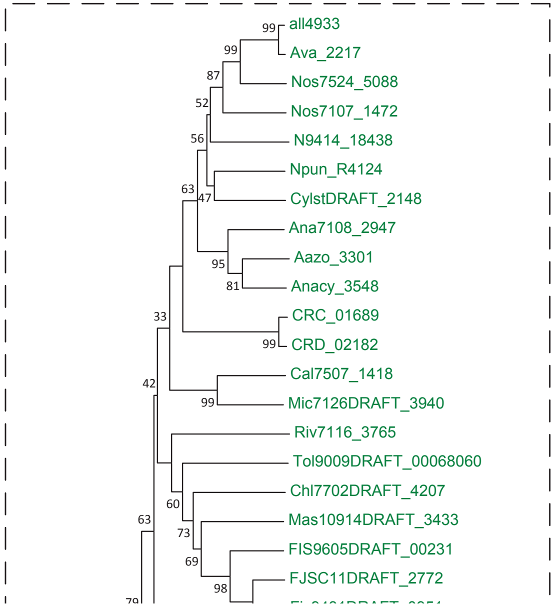

TDDLD
ADNC
[LY][IV][AMT]IFDADF
KAGN
[FGL][IMTV]DAD[ITV]
[DE][AS]D[FIL]C

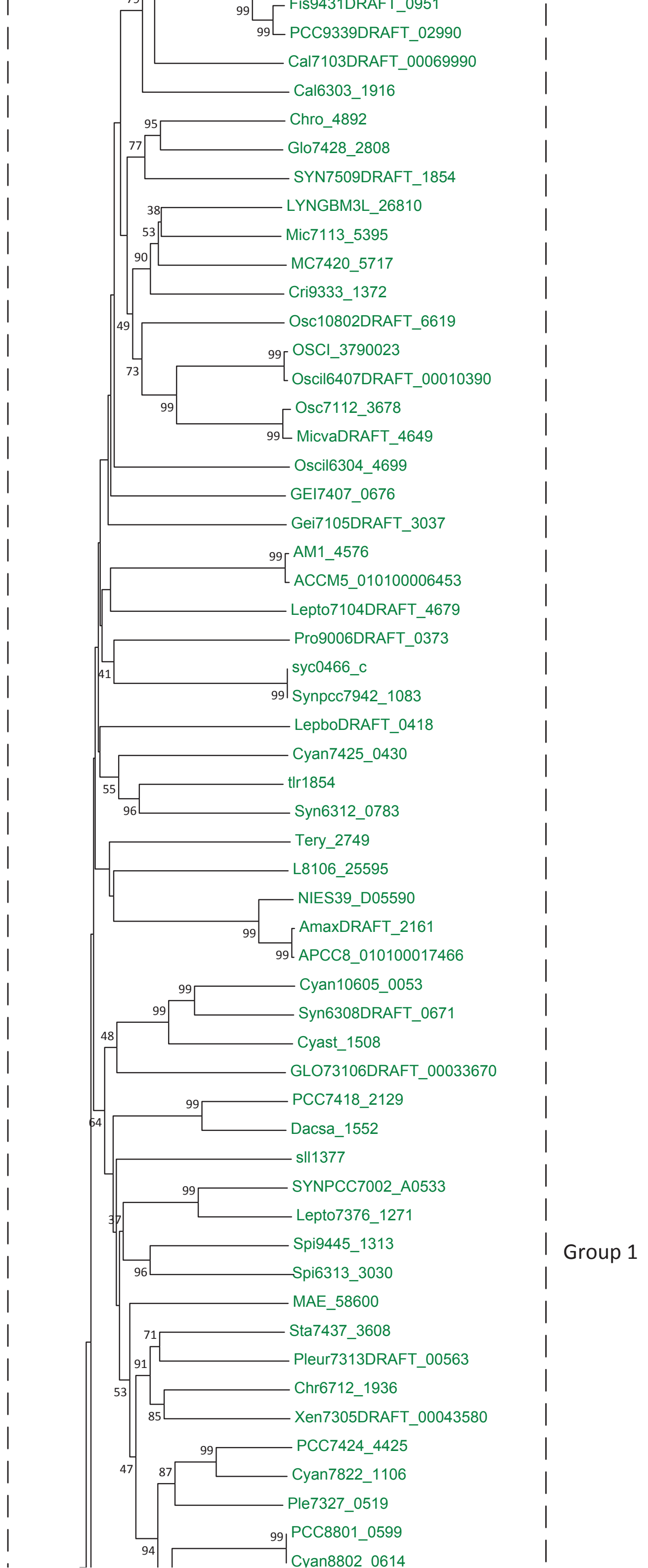
Fis9431DRAFT_0951
PCC9339DRAFT_02990
Cal7103DRAFT_00069990
Cal6303_1916
Chro_4892
Glo7428_2808
SYN7509DRAFT_1854
LYNGBM3L_26810
Mic7113_5395
MC7420_5717
Cri9333_1372
Osc10802DRAFT_6619
OSCI_3790023
Oscil6407DRAFT_00010390
Osc7112_3678
MicvaDRAFT_4649
Oscil6304_4699
GEI7407_0676
Gei7105DRAFT_3037
AM1_4576
ACCM5_010100006453
Lepto7104DRAFT_4679
Pro9006DRAFT_0373
syc0466_c
Synpcc7942_1083
LepboDRAFT_0418
Cyan7425_0430
tlr1854
Syn6312_0783
Tery_2749
L8106_25595
NIES39_D05590
AmaxDRAFT_2161
APCC8_010100017466
Cyan10605_0053
Syn6308DRAFT_0671
Cyast_1508
GLO73106DRAFT_00033670
PCC7418_2129
Dacsa_1552
sll1377
SYNPCC7002_A0533
Lepto7376_1271
Spi9445_1313
Spi6313_3030
MAE_58600
Sta7437_3608
Pleur7313DRAFT_00563
Chr6712_1936
Xen7305DRAFT_00043580
PCC7424_4425
Cyan7822_1106
Ple7327_0519
PCC8801_0599
Cyan8802_0614
Group 1

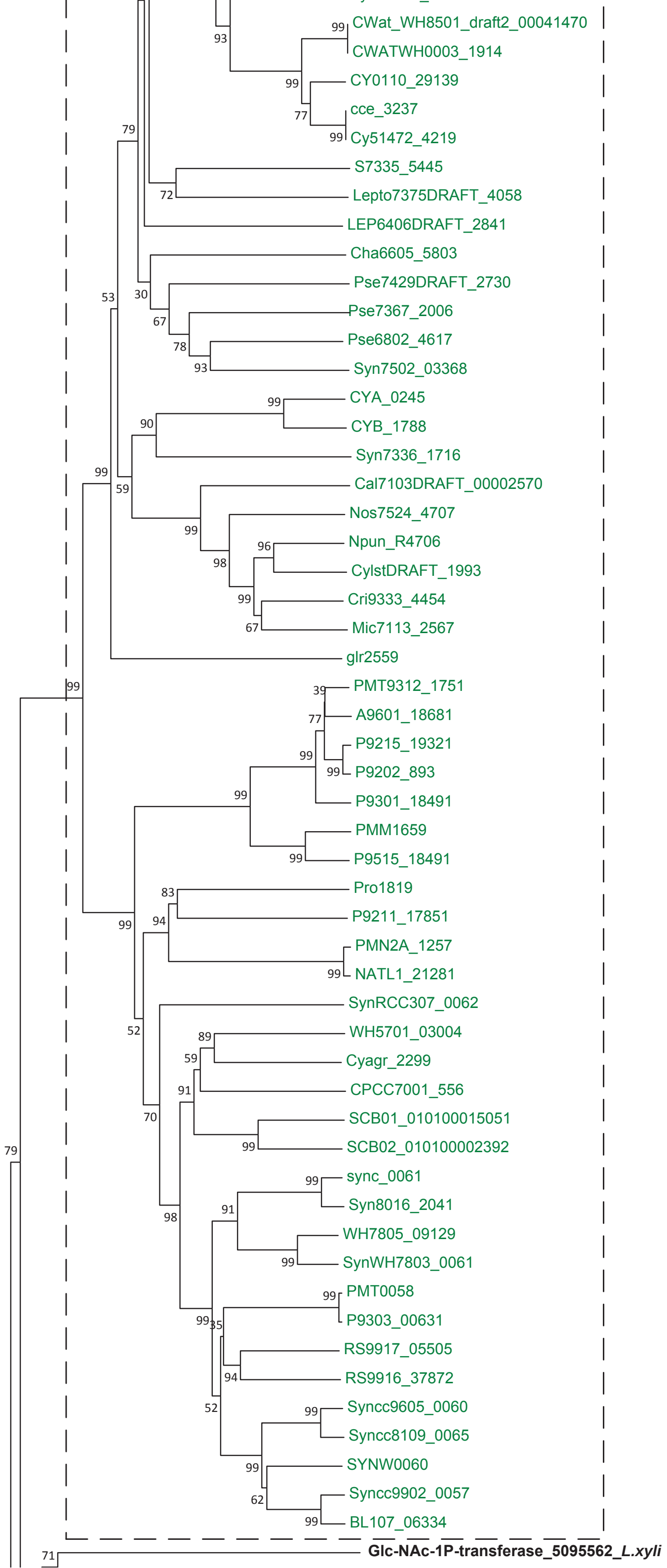

CWat_WH8501_draft2_00041470
CWATWH0003_1914
CY0110_29139
cce_3237
Cy51472_4219
S7335_5445
Lepto7375DRAFT_4058
LEP6406DRAFT_2841
Cha6605_5803
Pse7429DRAFT_2730
Pse7367_2006
Pse6802_4617
Syn7502_03368
CYA_0245
CYB_1788
Syn7336_1716
Cal7103DRAFT_00002570
Nos7524_4707
Npun_R4706
CylstDRAFT_1993
Cri9333_4454
Mic7113_2567
glr2559
PMT9312_1751
A9601_18681
P9215_19321
P9202_893
P9301_18491
PMM1659
P9515_18491
Pro1819
P9211_17851
PMN2A_1257
NATL1_21281
SynRCC307_0062
WH5701_03004
Cyagr_2299
CPCC7001_556
SCB01_010100015051
SCB02_010100002392
sync_0061
Syn8016_2041
WH7805_09129
SynWH7803_0061
PMT0058
P9303_00631
RS9917_05505
RS9916_37872
Syncc9605_0060
Syncc8109_0065
SYNW0060
Syncc9902_0057
BL107_06334
Glc-NAc-1P-transferase_5095562_L.xyli

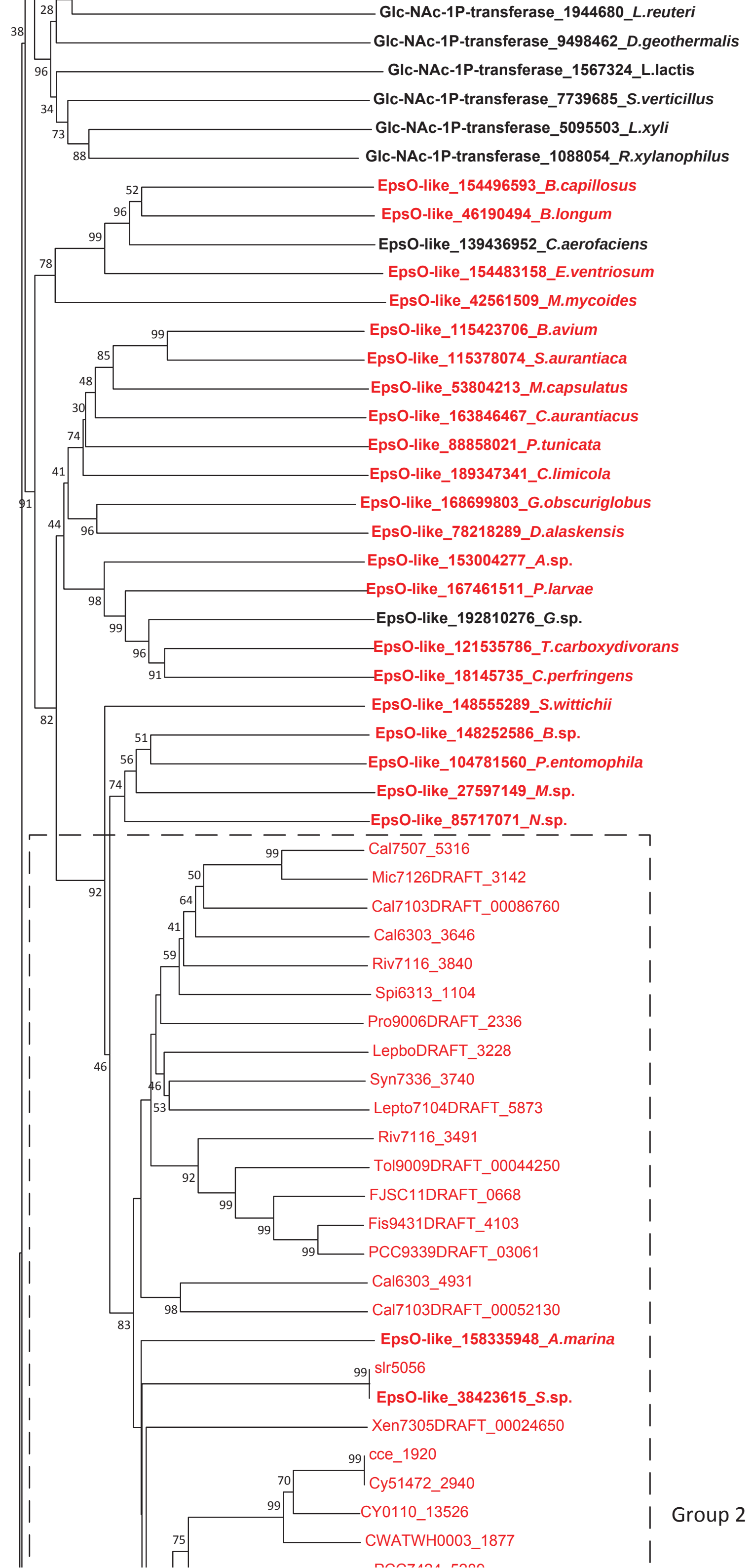
Glc-NAc-1P-transferase_1944680_L.reuteri
Glc-NAc-1P-transferase_9498462_D.geothermalis
Glc-NAc-1P-transferase_1567324_L.lactis
Glc-NAc-1P-transferase_7739685_S.verticillus
Glc-NAc-1P-transferase_5095503_L.xyli
Glc-NAc-1P-transferase_1088054_R.xylanophilus
EpsO-like_154496593_B.capillosus
EpsO-like_46190494_B.longum
EpsO-like_139436952_C.aerofaciens
EpsO-like_154483158_E.ventriosum
EpsO-like_42561509_M.mycoides
EpsO-like_115423706_B.avium
EpsO-like_115378074_S.aurantiaca
EpsO-like_53804213_M.capsulatus
EpsO-like_163846467_C.aurantiacus
EpsO-like_88858021_P.tunicata
EpsO-like_189347341_C.limicola
EpsO-like_168699803_G.obscuriglobus
EpsO-like_78218289_D.alaskensis
EpsO-like_153004277_A.sp.
EpsO-like_167461511_P.larvae
EpsO-like_192810276_G.sp.
EpsO-like_121535786_T.carboxydivorans
EpsO-like_18145735_C.perfringens
EpsO-like_148555289_S.wittichii
EpsO-like_148252586_B.sp.
EpsO-like_104781560_P.entomophila
EpsO-like_27597149_M.sp.
EpsO-like_85717071_N.sp.
Cal7507_5316
Mic7126DRAFT_3142
Cal7103DRAFT_00086760
Cal6303_3646
Riv7116_3840
Spi6313_1104
Pro9006DRAFT_2336
LepboDRAFT_3228
Syn7336_3740
Lepto7104DRAFT_5873
Riv7116_3491
Tol9009DRAFT_00044250
FJSC11DRAFT_0668
Fis9431DRAFT_4103
PCC9339DRAFT_03061
Cal6303_4931
Cal7103DRAFT_00052130
EpsO-like_158335948_A.marina
slr5056
EpsO-like_38423615_S.sp.
Xen7305DRAFT_00024650
cce_1920
Cy51472_2940
CY0110_13526
CWATWH0003_1877
Group 2

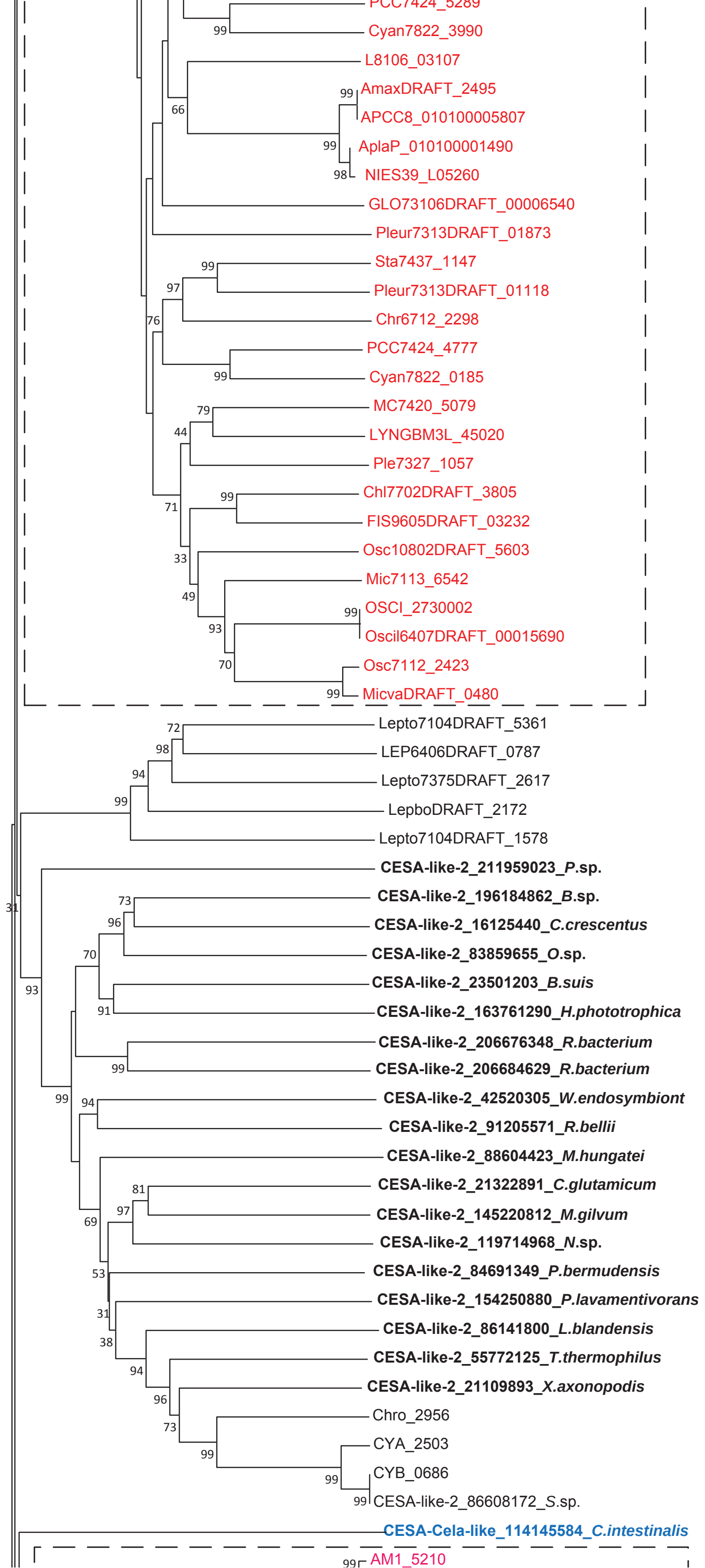
Cyan7822_3990
L8106_03107
AmaxDRAFT_2495
APCC8_010100005807
AplaP_010100001490
NIES39_L05260
GLO73106DRAFT_00006540
Pleur7313DRAFT_01873
Sta7437_1147
Pleur7313DRAFT_01118
Chr6712_2298
PCC7424_4777
Cyan7822_0185
MC7420_5079
LYNGBM3L_45020
Ple7327_1057
Chl7702DRAFT_3805
FIS9605DRAFT_03232
Osc10802DRAFT_5603
Mic7113_6542
OSCI_2730002
Oscil6407DRAFT_00015690
Osc7112_2423
MicvaDRAFT_0480
Lepto7104DRAFT_5361
LEP6406DRAFT_0787
Lepto7375DRAFT_2617
LepboDRAFT_2172
Lepto7104DRAFT_1578
CESA-like-2_211959023_P.sp.
CESA-like-2_196184862_B.sp.
CESA-like-2_16125440_C.crescentus
CESA-like-2_83859655_O.sp.
CESA-like-2_23501203_B.suis
CESA-like-2_163761290_H.phototrophica
CESA-like-2_206676348_R.bacterium
CESA-like-2_206684629_R.bacterium
CESA-like-2_42520305_W.endosymbiont
CESA-like-2_91205571_R.bellii
CESA-like-2_88604423_M.hungatei
CESA-like-2_21322891_C.glutamicum
CESA-like-2_145220812_M.gilvum
CESA-like-2_119714968_N.sp.
CESA-like-2_84691349_P.bermudensis
CESA-like-2_154250880_P.lavamentivorans
CESA-like-2_86141800_L.blandensis
CESA-like-2_55772125_T.thermophilus
CESA-like-2_21109893_X.axonopodis
Chro_2956
CYA_2503
CYB_0686
CESA-like-2_86608172_S.sp.
CESA-Cela-like_114145584_C.intestinalis
AM1_5210

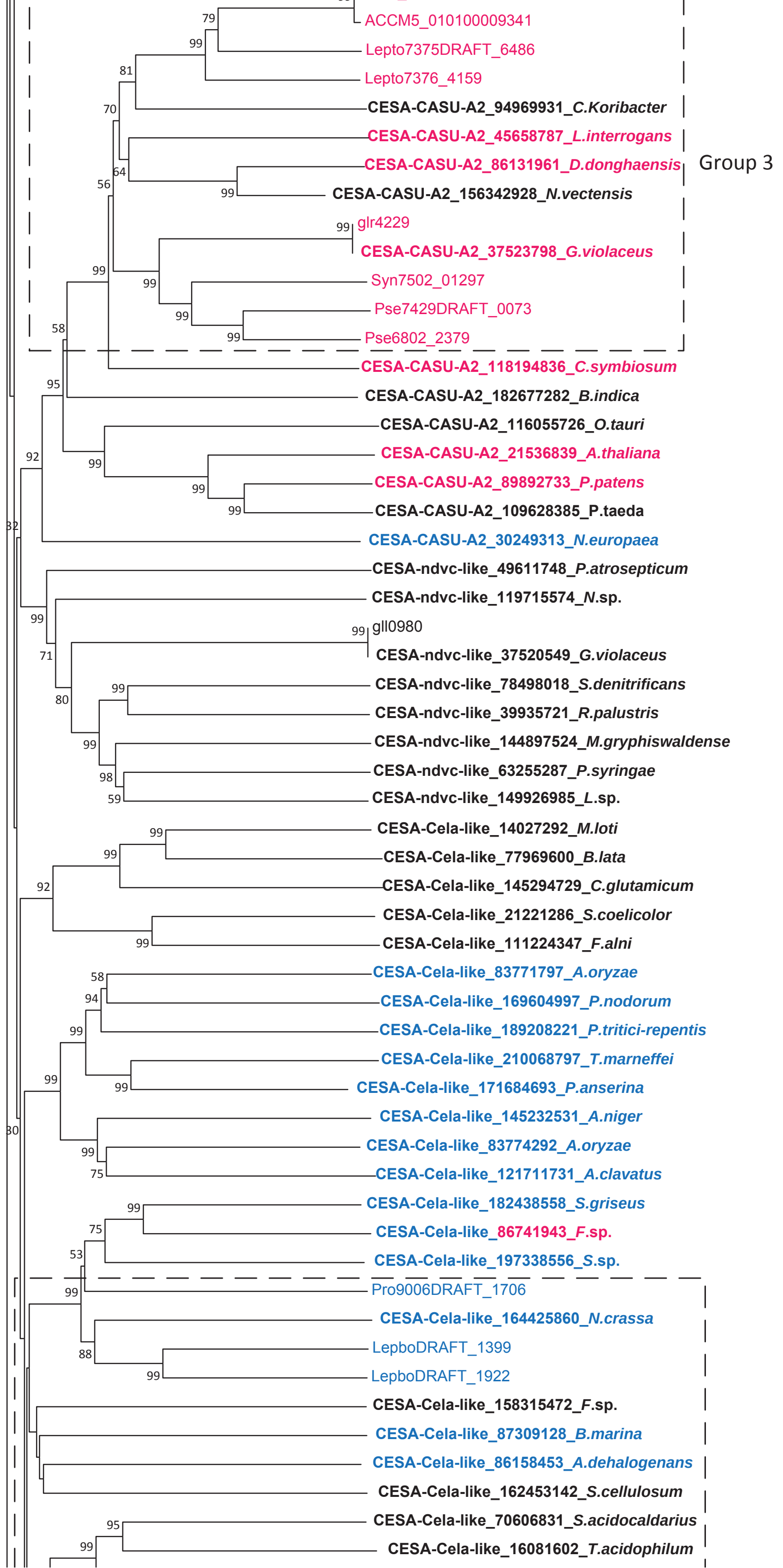
ACCM5_010100009341
Lepto7375DRAFT_6486
Lepto7376_4159
CESA-CASU-A2_94969931_C.Koribacter
CESA-CASU-A2_45658787_L.interrogans
CESA-CASU-A2_86131961_D.donghaensis
Group 3
CESA-CASU-A2_156342928_N.vectensis
glr4229
CESA-CASU-A2_37523798_G.violaceus
Syn7502_01297
Pse7429DRAFT_0073
Pse6802_2379
CESA-CASU-A2_118194836_C.symbiosum
CESA-CASU-A2_182677282_B.indica
CESA-CASU-A2_116055726_O.tauri
CESA-CASU-A2_21536839_A.thaliana
CESA-CASU-A2_89892733_P.patens
CESA-CASU-A2_109628385_P.taeda
CESA-CASU-A2_30249313_N.europaea
CESA-ndvc-like_49611748_P.atrosepticum
CESA-ndvc-like_119715574_N.sp.
gll0980
CESA-ndvc-like_37520549_G.violaceus
CESA-ndvc-like_78498018_S.denitrificans
CESA-ndvc-like_39935721_R.palustris
CESA-ndvc-like_144897524_M.gryphiswaldense
CESA-ndvc-like_63255287_P.syringae
CESA-ndvc-like_149926985_L.sp.
CESA-Cela-like_14027292_M.loti
CESA-Cela-like_77969600_B.lata
CESA-Cela-like_145294729_C.glutamicum
CESA-Cela-like_21221286_S.coelicolor
CESA-Cela-like_111224347_F.alni
CESA-Cela-like_83771797_A.oryzae
CESA-Cela-like_169604997_P.nodorum
CESA-Cela-like_189208221_P.tritici-repentis
CESA-Cela-like_210068797_T.marneffei
CESA-Cela-like_171684693_P.anserina
CESA-Cela-like_145232531_A.niger
CESA-Cela-like_83774292_A.oryzae
CESA-Cela-like_121711731_A.clavatus
CESA-Cela-like_182438558_S.griseus
CESA-Cela-like_86741943_F.sp.
CESA-Cela-like_197338556_S.sp.
Pro9006DRAFT_1706
CESA-Cela-like_164425860_N.crassa
LepboDRAFT_1399
LepboDRAFT_1922
CESA-Cela-like_158315472_F.sp.
CESA-Cela-like_87309128_B.marina
CESA-Cela-like_86158453_A.dehalogenans
CESA-Cela-like_162453142_S.cellulosum
CESA-Cela-like_70606831_S.acidocaldarius
CESA-Cela-like_16081602_T.acidophilum

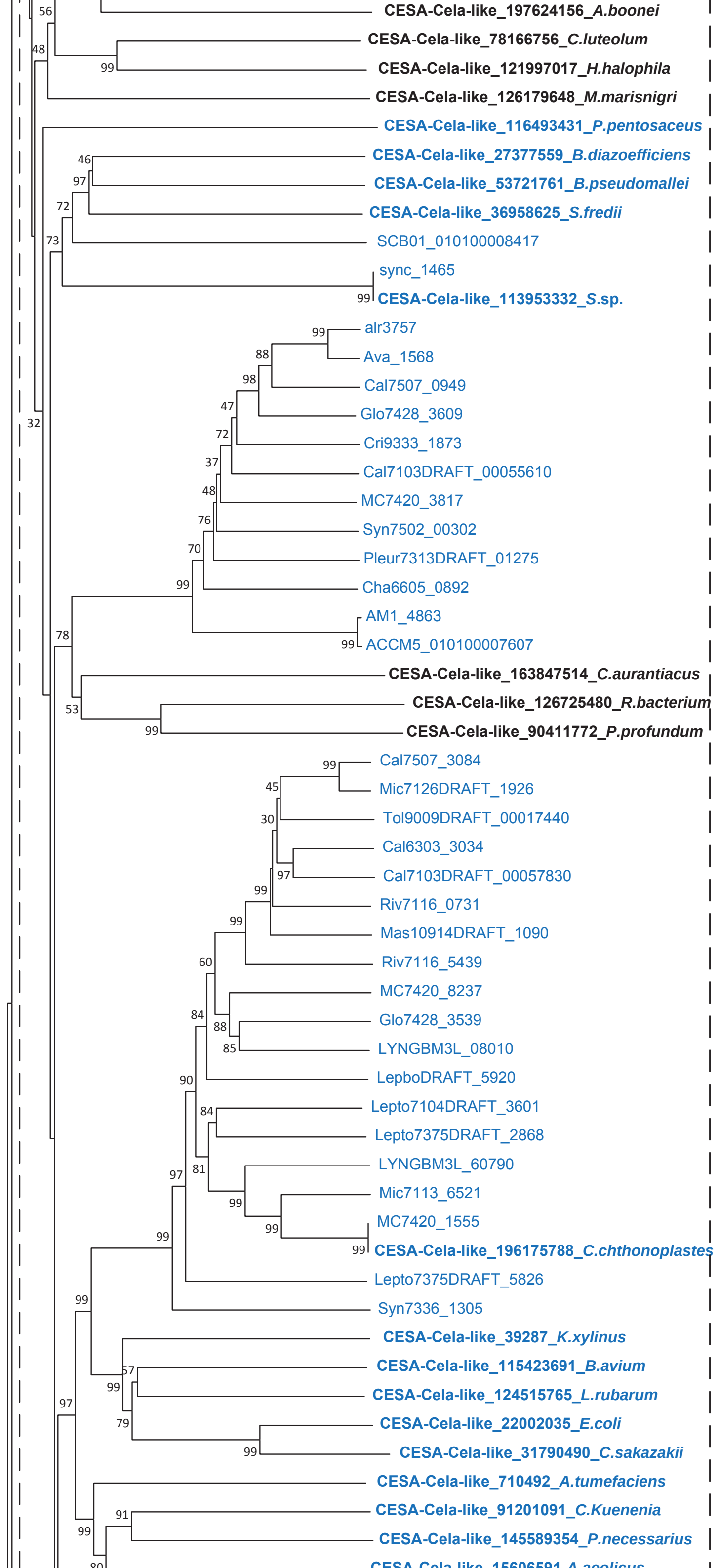
CESA-Cela-like_197624156_A.boonei
CESA-Cela-like_78166756_C.luteolum
CESA-Cela-like_121997017_H.halophila
CESA-Cela-like_126179648_M.marisnigri
CESA-Cela-like_116493431_P.pentosaceus
CESA-Cela-like_27377559_B.diazoefficiens
CESA-Cela-like_53721761_B.pseudomallei
CESA-Cela-like_36958625_S.fredii
SCB01_010100008417
sync_1465
CESA-Cela-like_113953332_S.sp.
alr3757
Ava_1568
Cal7507_0949
Glo7428_3609
Cri9333_1873
Cal7103DRAFT_00055610
MC7420_3817
Syn7502_00302
Pleur7313DRAFT_01275
Cha6605_0892
AM1_4863
ACCM5_010100007607
CESA-Cela-like_163847514_C.aurantiacus
CESA-Cela-like_126725480_R.bacterium
CESA-Cela-like_90411772_P.profundum
Cal7507_3084
Mic7126DRAFT_1926
Tol9009DRAFT_00017440
Cal6303_3034
Cal7103DRAFT_00057830
Riv7116_0731
Mas10914DRAFT_1090
Riv7116_5439
MC7420_8237
Glo7428_3539
LYNGBM3L_08010
LepboDRAFT_5920
Lepto7104DRAFT_3601
Lepto7375DRAFT_2868
LYNGBM3L_60790
Mic7113_6521
MC7420_1555
CESA-Cela-like_196175788_C.chthonoplastes
Lepto7375DRAFT_5826
Syn7336_1305
CESA-Cela-like_39287_K.xylinus
CESA-Cela-like_115423691_B.avium
CESA-Cela-like_124515765_L.rubarum
CESA-Cela-like_22002035_E.coli
CESA-Cela-like_31790490_C.sakazakii
CESA-Cela-like_710492_A.tumefaciens
CESA-Cela-like_91201091_C.Kuenenia
CESA-Cela-like_145589354_P.necessarius

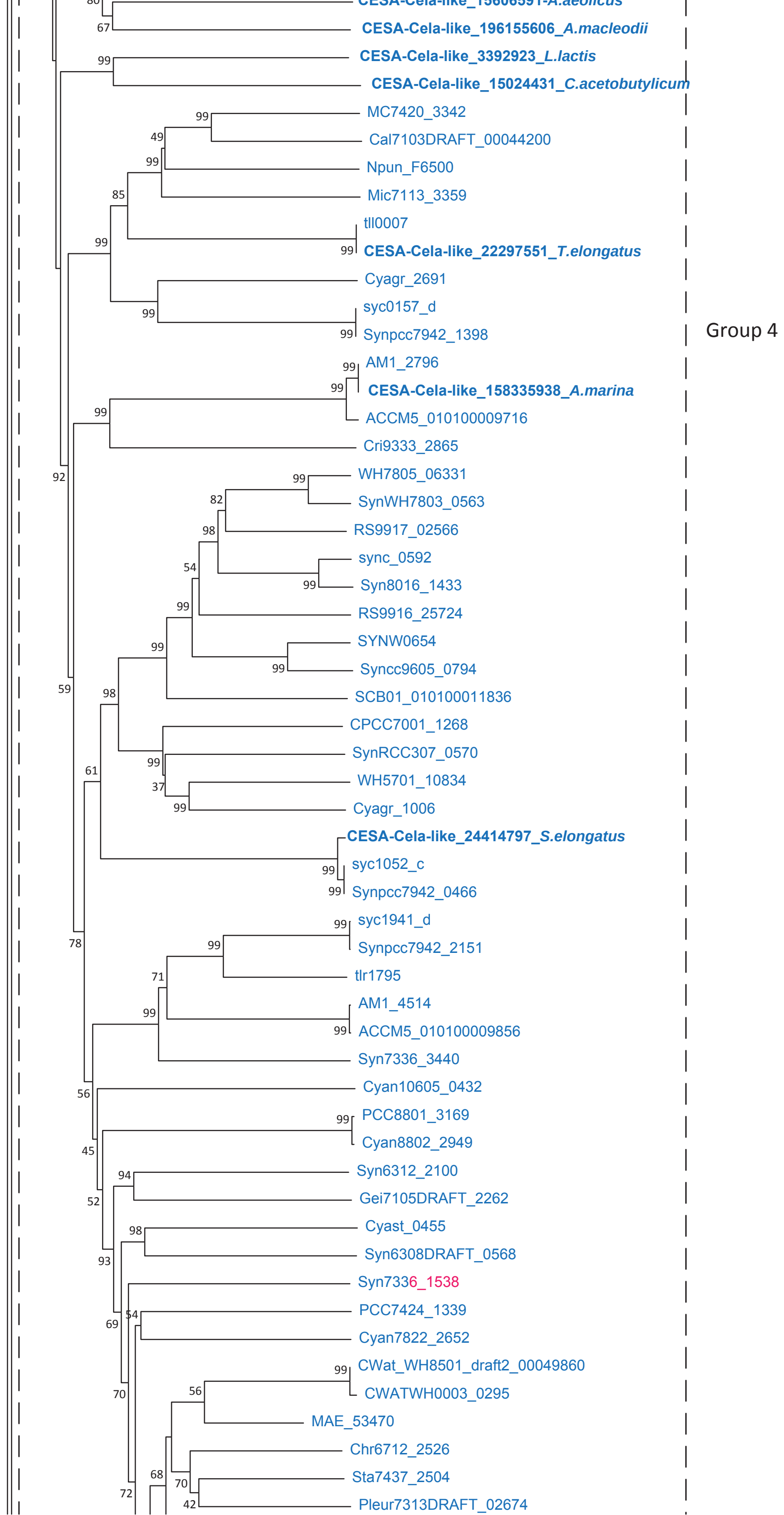
CESA-Cela-like_196155606_A.macleodii
CESA-Cela-like_3392923_L.lactis
CESA-Cela-like_15024431_C.acetobutylicum
MC7420_3342
Cal7103DRAFT_00044200
Npun_F6500
Mic7113_3359
tll0007
CESA-Cela-like_22297551_T.elongatus
Cyagr_2691
syc0157_d
Synpcc7942_1398
AM1_2796
CESA-Cela-like_158335938_A.marina
ACCM5_010100009716
Cri9333_2865
WH7805_06331
SynWH7803_0563
RS9917_02566
sync_0592
Syn8016_1433
RS9916_25724
SYNW0654
Syncc9605_0794
SCB01_010100011836
CPCC7001_1268
SynRCC307_0570
WH5701_10834
Cyagr_1006
CESA-Cela-like_24414797_S.elongatus
syc1052_c
Synpcc7942_0466
syc1941_d
Synpcc7942_2151
tlr1795
AM1_4514
ACCM5_010100009856
Syn7336_3440
Cyan10605_0432
PCC8801_3169
Cyan8802_2949
Syn6312_2100
Gei7105DRAFT_2262
Cyast_0455
Syn6308DRAFT_0568
Syn7336_1538
PCC7424_1339
Cyan7822_2652
CWat_WH8501_draft2_00049860
CWATWH0003_0295
MAE_53470
Chr6712_2526
Sta7437_2504
Pleur7313DRAFT_02674
Group 4

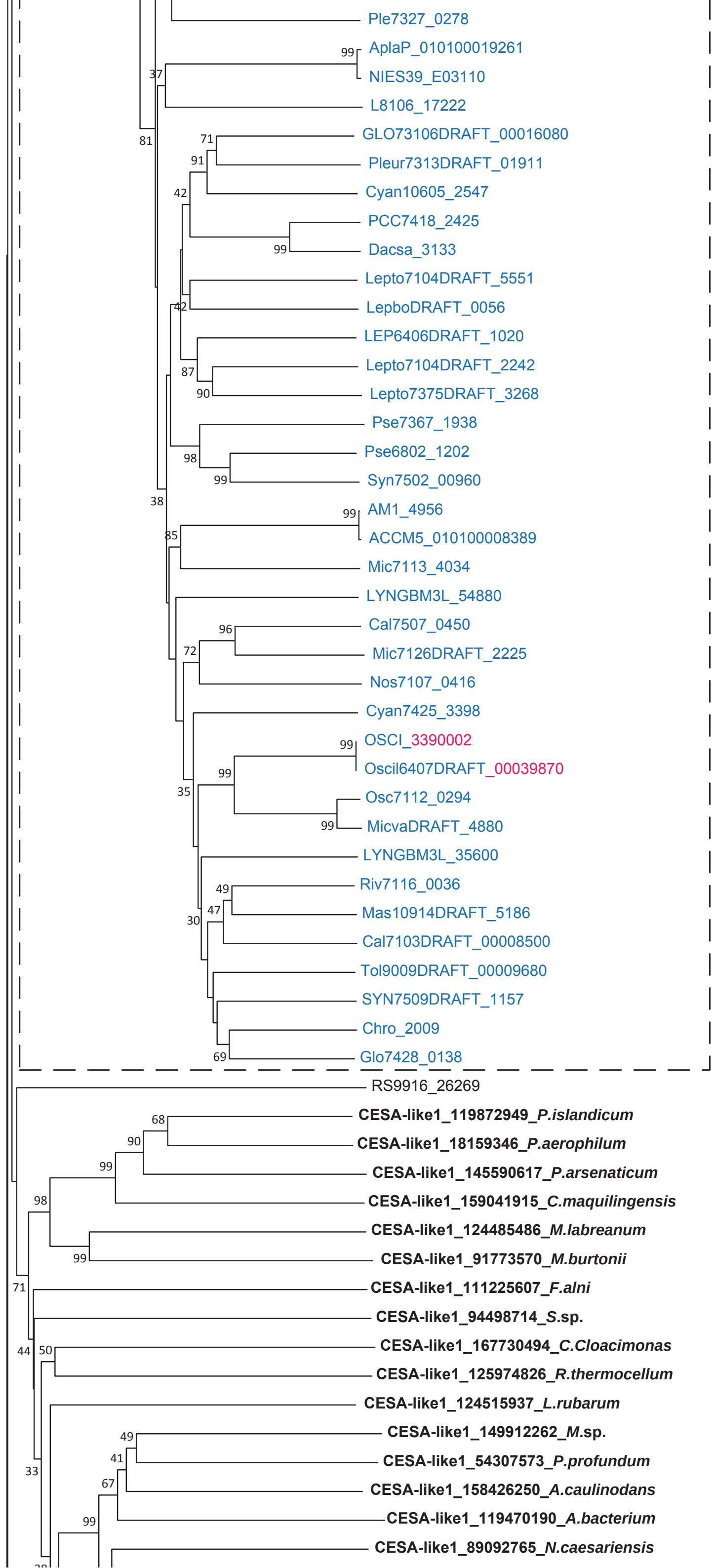
Ple7327_0278
AplaP_010100019261
NIES39_E03110
L8106_17222
GLO73106DRAFT_00016080
Pleur7313DRAFT_01911
Cyan10605_2547
PCC7418_2425
Dacsa_3133
Lepto7104DRAFT_5551
LepboDRAFT_0056
LEP6406DRAFT_1020
Lepto7104DRAFT_2242
Lepto7375DRAFT_3268
Pse7367_1938
Pse6802_1202
Syn7502_00960
AM1_4956
ACCM5_010100008389
Mic7113_4034
LYNGBM3L_54880
Cal7507_0450
Mic7126DRAFT_2225
Nos7107_0416
Cyan7425_3398
OSCI_3390002
Oscil6407DRAFT_00039870
Osc7112_0294
MicvaDRAFT_4880
LYNGBM3L_35600
Riv7116_0036
Mas10914DRAFT_5186
Cal7103DRAFT_00008500
Tol9009DRAFT_00009680
SYN7509DRAFT_1157
Chro_2009
Glo7428_0138
RS9916_26269
CESA-like1_119872949_P.islandicum
CESA-like1_18159346_P.aerophilum
CESA-like1_145590617_P.arsenaticum
CESA-like1_159041915_C.maquilingensis
CESA-like1_124485486_M.labreanum
CESA-like1_91773570_M.burtonii
CESA-like1_111225607_F.alni
CESA-like1_94498714_S.sp.
CESA-like1_167730494_C.Cloacimonas
CESA-like1_125974826_R.thermocellum
CESA-like1_124515937_L.rubarum
CESA-like1_149912262_M.sp.
CESA-like1_54307573_P.profundum
CESA-like1_158426250_A.caulinodans
CESA-like1_119470190_A.bacterium
CESA-like1_89092765_N.caesariensis

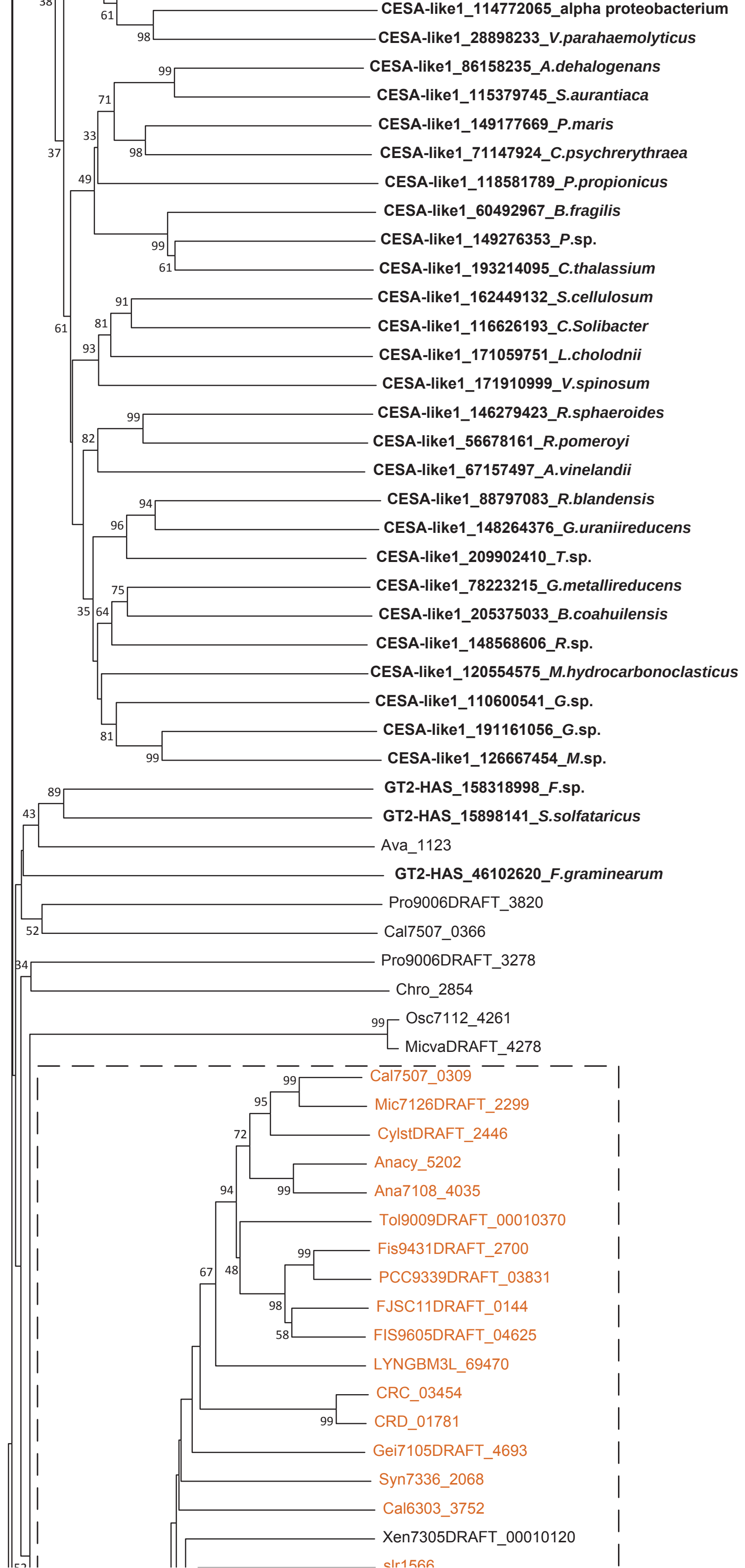
CESA-like1_114772065_alpha proteobacterium
CESA-like1_28898233_V.parahaemolyticus
CESA-like1_86158235_A.dehalogenans
CESA-like1_115379745_S.aurantiaca
CESA-like1_149177669_P.maris
CESA-like1_71147924_C.psychrerythraea
CESA-like1_118581789_P.propionicus
CESA-like1_60492967_B.fragilis
CESA-like1_149276353_P.sp.
CESA-like1_193214095_C.thalassium
CESA-like1_162449132_S.cellulosum
CESA-like1_116626193_C.Solibacter
CESA-like1_171059751_L.cholodnii
CESA-like1_171910999_V.spinosum
CESA-like1_146279423_R.sphaeroides
CESA-like1_56678161_R.pomeroyi
CESA-like1_67157497_A.vinelandii
CESA-like1_88797083_R.blandensis
CESA-like1_148264376_G.uraniireducens
CESA-like1_209902410_T.sp.
CESA-like1_78223215_G.metallireducens
CESA-like1_205375033_B.coahuilensis
CESA-like1_148568606_R.sp.
CESA-like1_120554575_M.hydrocarbonoclasticus
CESA-like1_110600541_G.sp.
CESA-like1_191161056_G.sp.
CESA-like1_126667454_M.sp.
GT2-HAS_158318998_F.sp.
GT2-HAS_15898141_S.solfataricus
Ava_1123
GT2-HAS_46102620_F.graminearum
Pro9006DRAFT_3820
Cal7507_0366
Pro9006DRAFT_3278
Chro_2854
Osc7112_4261
MicvaDRAFT_4278
Cal7507_0309
Mic7126DRAFT_2299
CylstDRAFT_2446
Anacy_5202
Ana7108_4035
Tol9009DRAFT_00010370
Fis9431DRAFT_2700
PCC9339DRAFT_03831
FJSC11DRAFT_0144
FIS9605DRAFT_04625
LYNGBM3L_69470
CRC_03454
CRD_01781
Gei7105DRAFT_4693
Syn7336_2068
Cal6303_3752
Xen7305DRAFT_00010120
slr1566

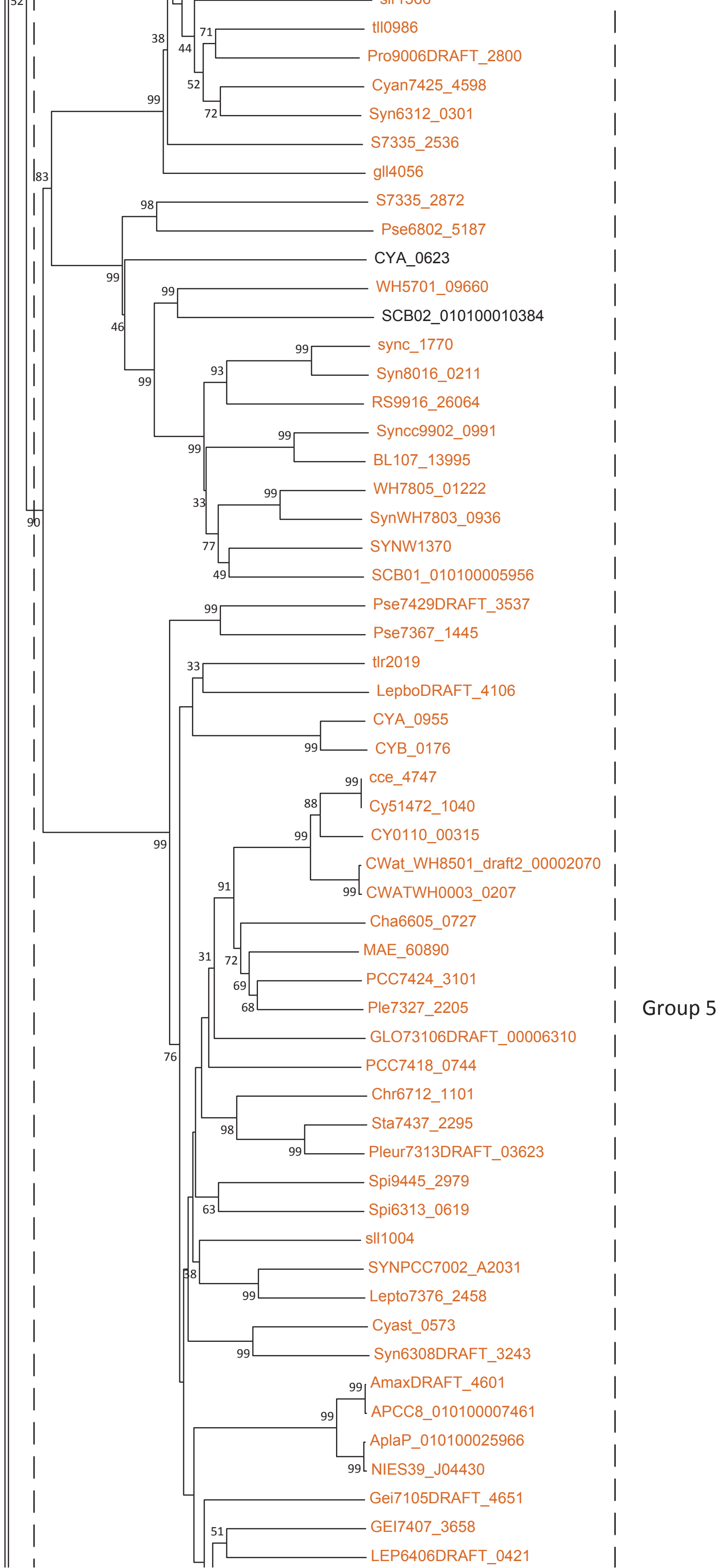
tll0986
Pro9006DRAFT_2800
Cyan7425_4598
Syn6312_0301
S7335_2536
gll4056
S7335_2872
Pse6802_5187
CYA_0623
WH5701_09660
SCB02_010100010384
sync_1770
Syn8016_0211
RS9916_26064
Syncc9902_0991
BL107_13995
WH7805_01222
SynWH7803_0936
SYNW1370
SCB01_010100005956
Pse7429DRAFT_3537
Pse7367_1445
tlr2019
LepboDRAFT_4106
CYA_0955
CYB_0176
cce_4747
Cy51472_1040
CY0110_00315
CWat_WH8501_draft2_00002070
CWATWH0003_0207
Cha6605_0727
MAE_60890
PCC7424_3101
Ple7327_2205
GLO73106DRAFT_00006310
PCC7418_0744
Chr6712_1101
Sta7437_2295
Pleur7313DRAFT_03623
Spi9445_2979
Spi6313_0619
sll1004
SYNPCC7002_A2031
Lepto7376_2458
Cyast_0573
Syn6308DRAFT_3243
AmaxDRAFT_4601
APCC8_010100007461
AplaP_010100025966
NIES39_J04430
Gei7105DRAFT_4651
GEI7407_3658
LEP6406DRAFT_0421
Group 5

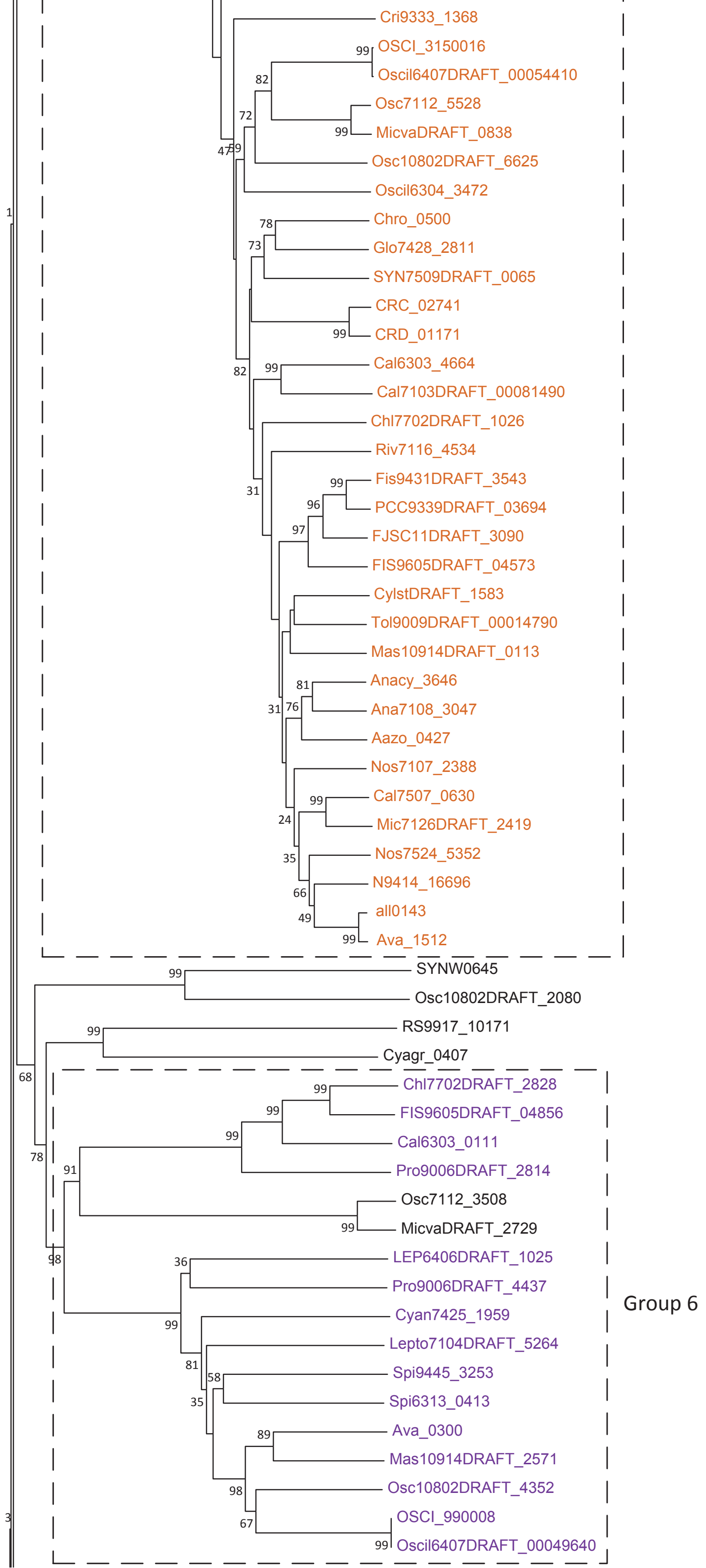
Cri9333_1368
OSCI_3150016
Oscil6407DRAFT_00054410
Osc7112_5528
MicvaDRAFT_0838
Osc10802DRAFT_6625
Oscil6304_3472
Chro_0500
Glo7428_2811
SYN7509DRAFT_0065
CRC_02741
CRD_01171
Cal6303_4664
Cal7103DRAFT_00081490
Chl7702DRAFT_1026
Riv7116_4534
Fis9431DRAFT_3543
PCC9339DRAFT_03694
FJSC11DRAFT_3090
FIS9605DRAFT_04573
CylstDRAFT_1583
Tol9009DRAFT_00014790
Mas10914DRAFT_0113
Anacy_3646
Ana7108_3047
Aazo_0427
Nos7107_2388
Cal7507_0630
Mic7126DRAFT_2419
Nos7524_5352
N9414_16696
all0143
Ava_1512
SYNW0645
Osc10802DRAFT_2080
RS9917_10171
Cyagr_0407
Chl7702DRAFT_2828
FIS9605DRAFT_04856
Cal6303_0111
Pro9006DRAFT_2814
Osc7112_3508
MicvaDRAFT_2729
LEP6406DRAFT_1025
Pro9006DRAFT_4437
Cyan7425_1959
Lepto7104DRAFT_5264
Spi9445_3253
Spi6313_0413
Ava_0300
Mas10914DRAFT_2571
Osc10802DRAFT_4352
OSCI_990008
Oscil6407DRAFT_00049640
Group 6

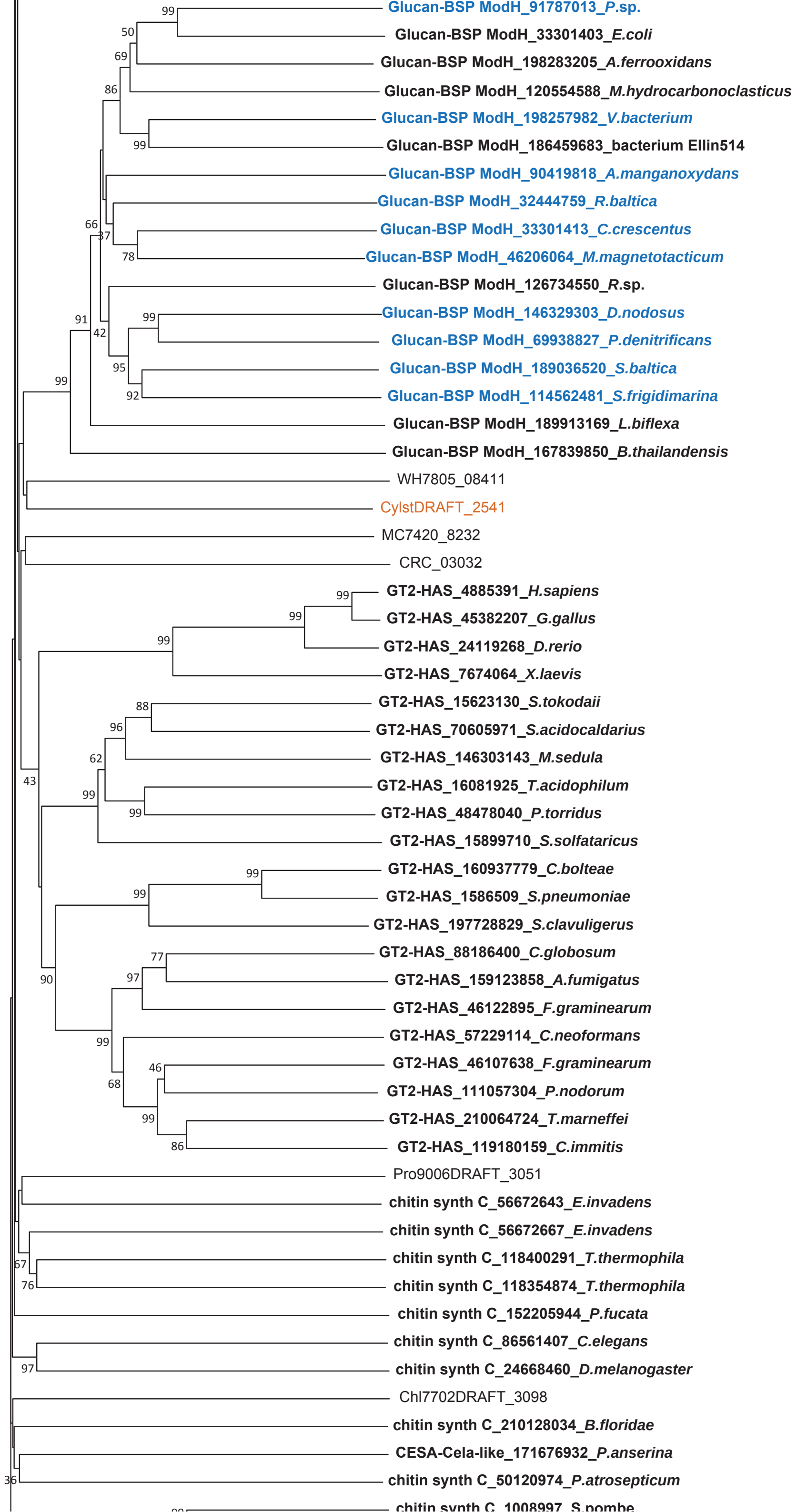
Glucan-BSP ModH_91787013_P.sp.
Glucan-BSP ModH_33301403_E.coli
Glucan-BSP ModH_198283205_A.ferrooxidans
Glucan-BSP ModH_120554588_M.hydrocarbonoclasticus
Glucan-BSP ModH_198257982_V.bacterium
Glucan-BSP ModH_186459683_bacterium Ellin514
Glucan-BSP ModH_90419818_A.manganoxydans
Glucan-BSP ModH_32444759_R.baltica
Glucan-BSP ModH_33301413_C.crescentus
Glucan-BSP ModH_46206064_M.magnetotacticum
Glucan-BSP ModH_126734550_R.sp.
Glucan-BSP ModH_146329303_D.nodosus
Glucan-BSP ModH_69938827_P.denitrificans
Glucan-BSP ModH_189036520_S.baltica
Glucan-BSP ModH_114562481_S.frigidimarina
Glucan-BSP ModH_189913169_L.biflexa
Glucan-BSP ModH_167839850_B.thailandensis
WH7805_08411
CylstDRAFT_2541
MC7420_8232
CRC_03032
GT2-HAS_4885391_H.sapiens
GT2-HAS_45382207_G.gallus
GT2-HAS_24119268_D.rerio
GT2-HAS_7674064_X.laevis
GT2-HAS_15623130_S.tokodaii
GT2-HAS_70605971_S.acidocaldarius
GT2-HAS_146303143_M.sedula
GT2-HAS_16081925_T.acidophilum
GT2-HAS_48478040_P.torridus
GT2-HAS_15899710_S.solfataricus
GT2-HAS_160937779_C.bolteae
GT2-HAS_1586509_S.pneumoniae
GT2-HAS_197728829_S.clavuligerus
GT2-HAS_88186400_C.globosum
GT2-HAS_159123858_A.fumigatus
GT2-HAS_46122895_F.graminearum
GT2-HAS_57229114_C.neoformans
GT2-HAS_46107638_F.graminearum
GT2-HAS_111057304_P.nodorum
GT2-HAS_210064724_T.marneffei
GT2-HAS_119180159_C.immitis
Pro9006DRAFT_3051
chitin synth C_56672643_E.invadens
chitin synth C_56672667_E.invadens
chitin synth C_118400291_T.thermophila
chitin synth C_118354874_T.thermophila
chitin synth C_152205944_P.fucata
chitin synth C_86561407_C.elegans
chitin synth C_24668460_D.melanogaster
Chl7702DRAFT_3098
chitin synth C_210128034_B.floridae
CESA-Cela-like_171676932_P.anserina
chitin synth C_50120974_P.atrosepticum
chitin synth C_1008997_S.pombe

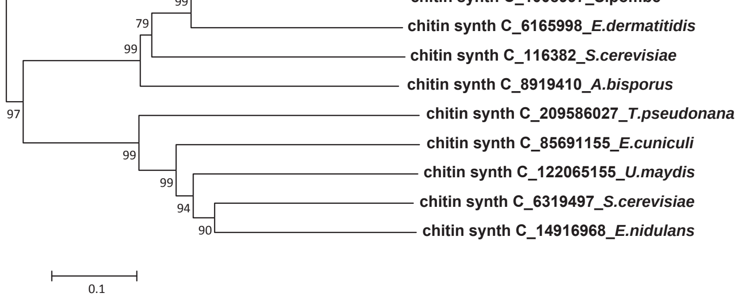

FIG S3 NJ phylogenetic tree of putative Alg8/BcsA. Amino acid patterns are shown in different colors (upper right side). Sequences possessing given pattern(s) are indicated in the same color(s) as the pattern(s). Dashed boxes indicate the groups of cyanobacterial sequences (label: locus tag) used to define each pattern. Reference sequences retrieved from the Conserved Domain Database (NCBI; label: conserved domain designation_GI number_ abbreviated species name) are indicated in bold.
